# Genetic Evidence Implicates the Immune System and Cholesterol Metabolism in the Aetiology of Alzheimer's Disease

**DOI:** 10.1371/journal.pone.0013950

**Published:** 2010-11-15

**Authors:** Lesley Jones, Peter A. Holmans, Marian L. Hamshere, Denise Harold, Valentina Moskvina, Dobril Ivanov, Andrew Pocklington, Richard Abraham, Paul Hollingworth, Rebecca Sims, Amy Gerrish, Jaspreet Singh Pahwa, Nicola Jones, Alexandra Stretton, Angharad R. Morgan, Simon Lovestone, John Powell, Petroula Proitsi, Michelle K. Lupton, Carol Brayne, David C. Rubinsztein, Michael Gill, Brian Lawlor, Aoibhinn Lynch, Kevin Morgan, Kristelle S. Brown, Peter A. Passmore, David Craig, Bernadette McGuinness, Stephen Todd, Clive Holmes, David Mann, A. David Smith, Seth Love, Patrick G. Kehoe, Simon Mead, Nick Fox, Martin Rossor, John Collinge, Wolfgang Maier, Frank Jessen, Britta Schürmann, Hendrik van den Bussche, Isabella Heuser, Oliver Peters, Johannes Kornhuber, Jens Wiltfang, Martin Dichgans, Lutz Frölich, Harald Hampel, Michael Hüll, Dan Rujescu, Alison M. Goate, John S. K. Kauwe, Carlos Cruchaga, Petra Nowotny, John C. Morris, Kevin Mayo, Gill Livingston, Nicholas J. Bass, Hugh Gurling, Andrew McQuillin, Rhian Gwilliam, Panos Deloukas, Ammar Al-Chalabi, Christopher E. Shaw, Andrew B. Singleton, Rita Guerreiro, Thomas W. Mühleisen, Markus M. Nöthen, Susanne Moebus, Karl-Heinz Jöckel, Norman Klopp, H.-Erich Wichmann, Eckhard Rüther, Minerva M. Carrasquillo, V. Shane Pankratz, Steven G. Younkin, John Hardy, Michael C. O'Donovan, Michael J. Owen, Julie Williams

**Affiliations:** 1 Medical Research Council (MRC) Centre for Neuropsychiatric Genetics and Genomics, Department of Psychological Medicine and Neurology, School of Medicine, Cardiff University, Cardiff, United Kingdom; 2 National Institute for Health Research Biomedical Research Centre for Mental Health at the South London and Maudsley National Health Service Foundation Trust and Institute of Psychiatry, Kings College, London, United Kingdom; 3 Department of Neuroscience, Institute of Psychiatry, Kings College, London, United Kingdom; 4 Institute of Public Health, University of Cambridge, Cambridge, United Kingdom; 5 Cambridge Institute for Medical Research, University of Cambridge, Cambridge, United Kingdom; 6 Mercer's Institute for Research on Aging, St. James Hospital and Trinity College, Dublin, Ireland; 7 Institute of Genetics, Queen's Medical Centre, University of Nottingham, Nottingham, United Kingdom; 8 Ageing Group, Centre for Public Health, School of Medicine, Dentistry and Biomedical Sciences, Queen's University Belfast, Belfast, United Kingdom; 9 Division of Clinical Neurosciences, School of Medicine, University of Southampton, Southampton, United Kingdom; 10 Clinical Neuroscience Research Group, Greater Manchester Neurosciences Centre, University of Manchester, Salford, United Kingdom; 11 Oxford Project to Investigate Memory and Ageing, University of Oxford, John Radcliffe Hospital, Oxford, United Kingdom; 12 Dementia Research Group, University of Bristol Institute of Clinical Neurosciences, Frenchay Hospital, Bristol, United Kingdom; 13 MRC Prion Unit, Department of Neurodegenerative Diseases, UCL Institute of Neurology, London, United Kingdom; 14 Dementia Research Centre, Department of Neurodegenerative Diseases, UCL Institute of Neurology, London, United Kingdom; 15 Department of Psychiatry, University of Bonn, Bonn, Germany; 16 Institute of Primary Medical Care, University Medical Center Hamburg-Eppendorf, Germany; 17 Department of Psychiatry and Psychotherapy, University of Erlangen-Nuremberg, Erlangen, Germany; 18 Landschaftsverband Rheinland-Hospital Essen, Department of Psychiatry and Psychotherapy, University Duisburg-Essen, Essen, Germany; 19 Institute for Stroke and Dementia Research, Klinikum der Universität München, Munich, Germany; 20 Department of Neurology, Klinikum der Universität München, Munich, Germany; 21 Department of Geriatric Psychiatry, Central Institute of Mental Health, Medical Faculty Mannheim, University of Heidelberg, Mannheim, Germany; 22 Discipline of Psychiatry, School of Medicine and Trinity College Institute of Neuroscience, Laboratory of Neuroimaging and Biomarker Research, Trinity College, University of Dublin, Dublin, Ireland; 23 Alzheimer Memorial Center and Geriatric Psychiatry Branch, Department of Psychiatry, Ludwig-Maximilian University, Munich, Germany; 24 Centre for Geriatric Medicine and Section of Gerontopsychiatry and Neuropsychology, Medical School, University of Freiburg, Freiburg, Germany; 25 Departments of Psychiatry, Neurology and Genetics, Washington University School of Medicine, St. Louis, Missouri, United States of America; 26 Department of Biology, Brigham Young University, Provo, Utah, United States of America; 27 Department of Mental Health Sciences, UCL, London, United Kingdom; 28 The Wellcome Trust Sanger Institute, Wellcome Trust Genome Campus, Hinxton, United Kingdom; 29 MRC Centre for Neurodegeneration Research, Department of Clinical Neuroscience, King's College London, Institute of Psychiatry, London, United Kingdom; 30 Laboratory of Neurogenetics, National Institute on Aging, National Institutes of Health, Bethesda, Maryland, United States of America; 31 Department of Genomics, Life and Brain Center, University of Bonn, Bonn, Germany; 32 Institute of Human Genetics, University of Bonn, Bonn, Germany; 33 Institute for Medical Informatics, Biometry and Epidemiology, University Hospital of Essen, University Duisburg-Essen, Essen, Germany; 34 Institute of Epidemiology, Helmholtz Zentrum München, German Research Center for Environmental Health, Neuherberg, Germany; 35 Institute of Medical Informatics, Biometry and Epidemiology, Ludwig-Maximilians-Universität, Munich, Germany; 36 Klinikum Grosshadern, Munich, Germany; 37 Department of Psychiatry, University of Gottingen, Gottingen, Germany; 38 Department of Neuroscience, Mayo Clinic College of Medicine, Jacksonville, Florida, United States of America; 39 Division of Biomedical Statistics and Informatics, Mayo Clinic and Mayo Foundation, Rochester, Minnesota, United States of America; 40 Department of Molecular Neuroscience and Reta Lilla Weston Laboratories, Institute of Neurology, London, United Kingdom; Massachusetts General Hospital and Harvard Medical School, United States of America

## Abstract

**Background:**

Late Onset Alzheimer's disease (LOAD) is the leading cause of dementia. Recent large genome-wide association studies (GWAS) identified the first strongly supported LOAD susceptibility genes since the discovery of the involvement of *APOE* in the early 1990s. We have now exploited these GWAS datasets to uncover key LOAD pathophysiological processes.

**Methodology:**

We applied a recently developed tool for mining GWAS data for biologically meaningful information to a LOAD GWAS dataset. The principal findings were then tested in an independent GWAS dataset.

**Principal Findings:**

We found a significant overrepresentation of association signals in pathways related to cholesterol metabolism and the immune response in both of the two largest genome-wide association studies for LOAD.

**Significance:**

Processes related to cholesterol metabolism and the innate immune response have previously been implicated by pathological and epidemiological studies of Alzheimer's disease, but it has been unclear whether those findings reflected primary aetiological events or consequences of the disease process. Our independent evidence from two large studies now demonstrates that these processes are aetiologically relevant, and suggests that they may be suitable targets for novel and existing therapeutic approaches.

## Introduction

Alzheimer's disease (AD) is the leading cause of dementia [1], [2] with a heritability of 56–79% [3]. It causes great social, emotional, and financial burdens to sufferers, their families and carers and there are no effective treatments that can slow or halt disease progression [4].

Genetic studies have been successful in identifying a number of causal loci (*APP*, *PSEN1* and *PSEN2*) for familial early onset forms of AD and in doing so have supported the amyloid cascade hypothesis [5]. Identical amyloid pathology to that observed in early onset disease is seen in the more common late onset form of AD (LOAD), thus implying the relevance of the amyloid cascade in both forms of disease. However, genetic variation at the early onset loci has not been reliably associated with LOAD. Indeed until recently, *APOE* was the only genetic locus with robust support in LOAD [6]. However, the publication of two genome-wide association studies (GWAS) and replications have recently established three novel LOAD susceptibility loci: *CLU*, *PICALM* and *CR1* [7], [8], [9], [10].

Genome-wide significant SNPs in complex traits generally explain only a proportion of the heritability of that disorder [11]. Much of the residual heritability underlying common traits appears to lie in SNPs that do not achieve genome-wide significance, meaning that a substantial proportion of the associated genetic signal in current GWAS is hidden below the genome-wide significance threshold. We know that SNPs that are robustly associated with particular common disorders are not randomly distributed across all genes. Instead, the implicated genes show biologically relevant relationships between each other [12], [13], [14], [15]. This is also true for SNPs in genes for which there is weaker individual evidence for association that falls short of stringent levels of genome-wide significance and statistical approaches have recently been developed to identify sets of functionally related genes containing genetic variants that collectively show evidence for association [14], [16]. We used the ALIGATOR algorithm [16] to examine SNPs in two AD GWAS [7], [8] for enrichment in related categories of genes. We also confirmed the results using gene set enrichment [15] and set-based analyses [17] to uncover sets of functionally related genes showing evidence for association with disease. The identification of such patterns in association datasets is likely to be crucial in moving beyond the genetic data to an understanding of function.

## Materials and Methods

### Data summary

The GWA studies were performed as described in Harold and colleagues [7] and Lambert and colleagues [8]. We have obtained approval to perform a genome-wide association study including 19,000 participants (Multi-centre Research Ethics Committee for Wales MREC 04/09/030; Amendment 2 and 4; approved 27^th^ July 2007). All individuals included in these analyses have provided informed written consent to take part in genetic association studies.

### Statistical analysis

#### Excess of SNPs passing significance thresholds

The number *N* of independent SNPs in the whole genome (excluding *APOE*, *CLU* and *PICALM*) was estimated by the method of Moskvina & Schmidt [18], as were the observed number of independent SNPs significant at each p-value criterion. In the absence of excess association, the expected number of independent SNPs significant at significance level α is distributed as a binomial (N,α).

#### Pathway analyses

ALIGATOR analysis was carried out essentially as in Holmans and colleagues [16] using gene ontology (GO) and KEGG defined functional categories [19], [20]. ALIGATOR converts a list of significant SNPs into a list of significant genes, and tests this list for enrichment within functional categories. Unlike methods designed for gene-expression data (where there is typically only one measurement per gene), ALIGATOR corrects for variable numbers of SNPs per gene. Each gene is counted once regardless of how many significant SNPs it contains, thus eliminating the influence of LD between SNPs within genes. Replicate gene lists of the same length as the original are generated by randomly sampling SNPs (thus correcting for variable gene size). The lists are used to obtain p-values for enrichment for each category and to correct these for testing multiple non-independent categories, and to test whether the number of significantly enriched categories is higher than expected. The present analysis was restricted to categories containing at least three genes: 6723 GO and 194 KEGG categories. Categories required at least two signals to be counted as enriched to remove the possibility of a small category being deemed significantly enriched based on one signal. SNPs that mapped to within 20kb of a gene (genome build 36_3) were assigned to that gene: if SNPs mapped within 20kb of more than one gene all such genes were included. Based upon the linkage disequilibrium (LD) structure of the region, 33 genes near *APOE* (chromosome 19: 49.6–50.6 Mb) were removed from the analysis. This was to remove the effects of genes whose evidence for association was merely a consequence of LD with the very strong *APOE* signal. *APOE* itself was included in the analysis since it is likely to be the AD susceptibility gene in this region. Any one SNP was not allowed to add more than one gene to any category to prevent the analysis being biased by SNPs located in multiple overlapping genes that are functionally related.

As independent validation of the results obtained from the analysis of GO categories, we also utilised the Mouse Genome Informatics (MGI) database [21]. This contains a comprehensive catalogue of behavioural, physiological and anatomical phenotypes observed in mutant mice. Extracting phenotype data for single gene studies (excluding all transgenes), we converted mouse genes to their human orthologs using the MGI's mouse/human orthology assignment. We were able to map 5671 different phenotypic annotation terms to 6297 human genes, and the gene sets corresponding to each annotation were tested for enrichment in the Harold et al. data using ALIGATOR, as described previously.

#### Set-based analyses on genes and gene sets

Two gene-wide analyses were carried out using PLINK [17]. The first was based on the most significant single-SNP p-value and the second, ‘set-based’, analysis was based on the average chi-squared statistic of all SNPs in the gene, calculated under an allelic association model. The former analysis will detect significant association in genes with a single strong signal, while the latter analysis will highlight genes with several independent signals, even if each of these is of modest significance individually. The analyses are thus complementary. Significance in each case was obtained by comparing the test statistic in the observed data to that obtained when disease status was randomly permuted among individuals, thereby accounting for inter-SNP LD. 1000 permutations were performed (10000 for genes with a gene-wide p-value<0.01). Genes without at least one SNPs p<0.05 were not analysed.

As a validation of the ALIGATOR results, set-based analysis was also performed on the set of SNPs within each of the GO processes that were significantly enriched in both GWAS datasets. 1000 permutations were used for each process. Set-based analysis is robust to LD between and within genes, as well as SNPs being in several genes.

#### Gene-set-enrichment (GSEA) analysis

As a further validation of the ALIGATOR results, gene-set enrichment analysis (GSEA) was performed using the method described in Wang et al. [15]. Rather than defining a list of significant genes, GSEA ranks all genes in order of a gene-wide association statistic, and tests whether the genes in a particular gene set have higher rank overall than would be expected by chance. Following Wang et al., in order to allow for varying numbers of SNPs per gene, the gene-wide statistic used was the Simes-corrected single-SNP p-value [22]. Since apparently significant GSEA enrichments can result from a single gene that is strongly associated with disease [23], we removed the APOE region before performing the analysis.

## Results

In the GWAS study of Harold et al. [7] involving approximately 12,000 AD cases and controls, we observed a considerable excess of SNPs surpassing different thresholds of significance when compared with those expected by chance (Table 1), suggesting the existence of many LOAD susceptibility loci that were not detected at genome-wide significance. To exploit any signal arising from the excess of nominally significant SNPs in the GWAS, we used ALIGATOR [16], to identify functional categories that were enriched for association signals.

**Table 1:** More significant SNPs are seen than expected.

Significance α	# SNPs in original data	Estimated # independent SNPs	# SNPs expected	p-value	Ratio: Est/Exp
0.000001	1	1	0.408	0.177	2.45
0.00001	16	12.6	4.0	7.5×10^−6^	3.17
0.0001	75	65.53	38.3	5.3×10^−6^	1.71
0.001	706	601.22	362.2	3.3×10^−36^	1.66
0.01	6064	4837.72	3294.6	8.7×10^−171^	1.47
0.05	29122	22064.52	14571.4	<10^−200^	1.51

The total number of SNPs considered was 528488, the whole genome without *APOE*, *PICALM* or *CLU* SNPs. The estimated number of independent SNPs at each significance level takes linkage disequilibrium into account. The ratio Est/Exp is the ratio of the estimated number of significant SNPs for any α divided by the expected number of independent SNPs seen at that α.

We found that in the real data, significantly more GO categories were enriched for genes containing at least one SNP surpassing varying thresholds of nominal significance compared with the simulated data (Table 2). The most significant excess in enriched GO categories was based upon a list of 589 autosomal genes defined by having at least 1 SNP with p<0.001. In that analysis, there was a significant excess of categories regardless of the threshold (p<0.05, p<0.01, p<0.001) for defining a category containing a significant excess of associated genes. This list was used to define enriched GO categories for further study [16]. However, we note that significant excesses of enriched categories were also observed for gene lists defined by other SNP association criteria and that the categories themselves were similar, suggesting the conclusions of this study are not highly sensitive to the threshold used to define nominal SNP association.

**Table 2:** Significantly more GO pathways are identified than expected.

SNP list criterion	#genes	p<0.05		p<0.01		p<0.001	
		#cat	p	#cat	p	#cat	p
p<1e-4	72	115	0.009	50	0.006	16	0.008
p<1e-3*	**589**	**254**	**0.005**	**127**	**<0.001**	**57**	**<0.001**
p<0.005	2212	291	0.006	76	0.006	18	<0.001
p<0.01	3703	282	0.023	64	0.031	8	0.110
p<0.05	10709	228	0.078	44	0.096	4	0.295

The analysis used only autosomes and was restricted to GO categories with at least two hits. SNPs that mapped to within 20kb of a gene were assigned to that gene: if SNPs mapped within 20kb of more than one gene all such genes were included. SNPs in the APOE region (49.6–50.6 Mb on chromosome 19, 34 genes) were removed from the analysis. Only the most significant of any GO categories containing the same list of significant genes was permitted and any one SNP was not allowed to add more than one gene to any GO category. P-values were generated using 5000 permutations of the data except for * 50,000 permutations.

From the most significantly enriched categories in the Harold GWAS [7] (Table 3, Table S1), two main themes emerged: sterol and lipid metabolism and the immune response. Many of the top 20 categories relate to these processes and aspects of these processes are detected throughout the significant GO categories. Note that several categories show significant enrichment even after correcting for the multiple GO categories tested (study-wide p<0.05). A similar analysis was performed on the GWAS data from Lambert and colleagues, in which the same SNP threshold of p<0.001 defined a list of 423 autosomal genes. Sterol and lipid metabolism and the immune system again emerge as clear themes in the list of significantly enriched categories derived from the Lambert data (Table S2). None of the categories relating to β-amyloid (Aβ) and its processing were significant in this analysis either in the Harold (Table S1) or Lambert (Table S2) data.

**Table 3:** The most significantly overrepresented gene ontology processes.

GO process	category total	# genes on list	p-value	Study-wide p-value	Function
GO:0032488	4	3	<1.00E-05	0.042	Cdc42 protein signal transduction
GO:0033700	8	4	<1.00E-05	0.042	phospholipid efflux
GO:0043691	14	7	<1.00E-05	0.042	reverse cholesterol transport
GO:0030301	34	8	<1.00E-05	0.042	cholesterol transport
GO:0015918	34	8	<1.00E-05	0.042	sterol transport
GO:0034369	18	6	<1.00E-05	0.042	plasma lipoprotein particle remodeling
GO:0034368	18	6	<1.00E-05	0.042	protein-lipid complex remodeling
GO:0034367	18	6	<1.00E-05	0.042	macromolecular complex remodeling
GO:0034375	11	5	<1.00E-05	0.042	high-density lipoprotein particle remodeling
GO:0034382	3	3	<1.00E-05	0.042	chylomicron remnant clearance
GO:0016125	87	11	2.00E-05	0.066	sterol metabolic process
GO:0022411	55	8	2.00E-05	0.066	cellular component disassembly
GO:0006958	28	6	2.00E-05	0.066	complement activation, classical pathway
GO:0002455	28	6	2.00E-05	0.066	humoral immune response mediated by circulating immunoglobulin
GO:0042632	33	7	4.00E-05	0.093	cholesterol homeostasis
GO:0055092	33	7	4.00E-05	0.093	sterol homeostasis
GO:0006956	37	6	4.00E-05	0.093	complement activation
GO:0002541	38	6	4.00E-05	0.093	activation of plasma proteins involved in acute inflammatory response
GO:0045087	120	11	6.00E-05	0.113	innate immune response
GO:0008203	78	10	8.00E-05	0.129	cholesterol metabolic process

The 589 genes identified as having GWAS SNP signals p<0.001 were used: APOE was included in the gene list. In this analysis one SNP was not allowed to add more than one gene to any gene ontology category. “Study-wide p-value” is the probability of obtaining by chance *at least one* GO category with a category-specific enrichment p-value at least as significant as that observed. . There are genes in the pathways that are in close proximity and that are both included because of the same significant SNP in both genes, as genes were associated with a SNP if it mapped within 20kb of a given gene: details of these genes are in Tables 5 and 6. If CR2, IL18RAP and IL18R1 are removed (effectively counting CR1/CR2 as one signal and IL1RL1/IL18RAP/IL18R1 as one signal) the GO analysis yields GO:0006958 and GO:0002455: 27 genes, 5 significant (0.60 expected) p = 0.0002, GO:0006956: 36 genes, 5 significant (0.79 expected) p = 0.0004, GO:0002541: 37 genes, 5 significant (0.81 expected) p = 0.0004 and GO:0045087: 117 genes, 8 significant (2.58 expected) p = 0.0044. Only processes are presented. The full data are available in Table S1.

In order to investigate whether we could replicate this signal we restricted enrichment analysis of the Lambert data [24] to the 173 GO processes with enrichment p<0.05 in the Harold data [7]. Of the 173 categories, twenty-five processes were also enriched for genes containing a SNP with p<0.05 in the replication dataset, a number that is significantly greater than expected (p = 0.0045). This provides evidence for a common underlying genetic association between the studies. Note that the significance of this overlap is not due to the biological areas in question being relatively well annotated since the same set of processes was tested in both the real and simulated gene lists (see Methods). Table 4 shows that these processes relate to the immune system and complement pathways and to cholesterol and lipid metabolism with one exception: cholinergic synaptic transmission. For the majority of these processes, their joint enrichment (defined as the product of the enrichment p-values in the two studies) is significant even after correction for testing multiple GO categories, thus providing strong evidence for their involvement in disease susceptibility.

**Table 4:** List of GO categories significantly (p<0.05) enriched in both GWAS.

GO category	ALIGATOR p (Harold)	ALIGATOR p (Lambert)	Joint p	Empirical GSEA p (Harold)	Empirical GSEA p (Lambert)	Set-based p (Harold)	Function
GO:0015918	<0.00001	0.0012	0.0079	<0.0001	0.0072	0.003	sterol transport
GO:0030301	<0.00001	0.0012	0.0079	<0.0001	0.0072	0.003	cholesterol transport
GO:0043691	<0.00001	0.0086	0.0079	<0.0001	0.0876	0.002	reverse cholesterol transport
GO:0033700	<0.00001	0.0278	0.0079	0.0018	0.5852	0.008	phospholipid efflux
GO:0034375	<0.00001	0.0348	0.0079	0.0014	0.7218	0.006	high-density lipoprotein particle remodeling
GO:0006958	0.00002	0.0108	0.0082	0.0004	0.0040	0.002	complement activation, classical pathway
GO:0002455	0.00002	0.0108	0.0082	0.0004	0.0040	0.002	humoral immune response mediated by circulating immunoglobulin
GO:0042632	0.00004	0.0092	0.0086	0.0000	0.3888	0.003	cholesterol homeostasis
GO:0055092	0.00004	0.0092	0.0086	0.0000	0.3888	0.003	sterol homeostasis
GO:0006956	0.00004	0.0226	0.0099	0.0012	0.0018	0.004	complement activation
GO:0002541	0.00004	0.0228	0.0099	0.0016	0.0010	0.004	activation of plasma proteins involved in acute inflammatory response
GO:0002504	0.00232	0.0008	0.0122	0.0360	0.0506	0.033	antigen processing and presentation of peptide or polysaccharide antigen via MHC class II
GO:0055088	0.00026	0.0170	0.0181	0.0004	0.5816	0.010	lipid homeostasis
GO:0006869	0.00028	0.0332	0.0306	0.0000	0.0046	0.003	lipid transport
GO:0016064	0.00048	0.0412	0.0519	0.0020	0.0044	0.004	immunoglobulin mediated immune response
GO:0010876	0.00048	0.0426	0.0531	0.0000	0.0032	0.003	lipid localization
GO:0010872	0.00120	0.0198	0.0592	0.0188	0.3844	0.007	regulation of cholesterol esterification
GO:0019724	0.00058	0.0450	0.0633	0.0014	0.0042	0.008	B cell mediated immunity
GO:0006955	0.00126	0.0214	0.0647	0.0030	0.0010	0.003	immune response
GO:0034377	0.00318	0.0250	0.1379	0.0068	0.6926	0.027	plasma lipoprotein particle assembly
GO:0065005	0.00318	0.0250	0.1379	0.0068	0.6926	0.027	protein-lipid complex assembly
GO:0002443	0.00410	0.0320	0.1872	0.0028	0.0018	0.022	leukocyte mediated immunity
GO:0007271	0.01574	0.0090	0.1955	0.1226	0.1780	0.005	synaptic transmission, cholinergic
GO:0033344	0.02684	0.0108	0.2972	0.0002	0.2094	0.020	cholesterol efflux
GO:0045940	0.00750	0.0464	0.3305	0.0262	0.6062	0.008	positive regulation of steroid metabolic process

“Joint p” is the probability of observing by chance at least one category among the entire set of categories tested with joint enrichment (defined as the product of enrichment p-values from the two GWAS) at least as extreme as that observed in the real data. This corrects for the multiple non-independent GO categories being tested. GSEA is gene set enrichment analysis.

ALIGATOR enrichment analysis was also performed on 194 KEGG [20] human pathways. Six KEGG pathways were significantly enriched (p<0.05) in both the Harold and Lambert datasets [7], [8]. This is higher than would be expected by chance (p = 1.16×10^−3^). These pathways, and their enrichment p-values, are listed in Table S3. The genes contained in the pathways, together with the p-values of the most significant SNP are listed in Table S4. Inspection of Table S4 reveals that, in addition to *CR1* and *CR2* (members of pathway hsa4640: hematopoietic cell lineage), there are several genes in the HLA region contributing to the enrichment signal in both datasets. These genes may reflect the same association signal due to LD, and were therefore collapsed into one signal: when the enrichment analysis was repeated, no pathway was significantly enriched (p<0.05) in both datasets. The enrichment significance for each of the MGI mouse phenotype annotations is shown in Table S5. It can be seen that several of the most significantly enriched annotations relate to lipids, cholesterol and innate immunity, similar to the top-ranking GO categories in Tables 3 and 4.

To investigate which genes contribute to the association signals seen in the enriched GO processes identified by both GWAS, two further analyses were performed in the Harold data using PLINK [17]. First, a gene-wide correction was applied to the most significant single-SNP p-value in each gene. Second, a ‘set-based’ analysis was applied to each gene based on the average single-SNP chi-squared statistic of all SNPs in that gene. The latter analysis measures the overall association evidence across a gene, highlighting genes with multiple association signals. Results for all genes in the cholesterol-related processes listed in Table 4 are given in Table S6, and for all genes in the immune-related processes in Table S7. Gene-wide significance of genes with a SNP with p<0.001 in either study are shown for lipid-related genes in Table 5 and for immune-related genes in Table 6. As expected, most of the genes in Tables 5 and 6 show gene-wide significant association evidence (Tables S6 and S7), but other genes in these processes are also significant. Tables 5 and 6 also give the most significantly associated SNP from each gene for both studies and the r^2^ between them. Note that the immune-related genes include both *CLU*, which contains a SNP showing genome-wide significant association in both GWAS, and *CR1*, which contains a SNP that is genome-wide significant in one study [8] and has a p-value<1×10^−5^ in the other [7]. It was not possible to perform gene-wide analyses on the Lambert data since individual genotypes were not available. However, the most significant p-values from the genes of interest are shown in Tables 5, S6 and S7. Similar gene-wide analyses were performed on the genes in the enriched KEGG pathways (Table S4).

**Table 5:** Genes with a SNP with p<0.001 in cholesterol and lipid-related processes that are significantly enriched in both GWAS.

Gene Symbol	Chr location (Mb)	No. of SNPs (Harold)	Best p-value (Harold)	No. of SNPs (Lambert)	Best p-value (Lambert)	Best SNP (Harold)	Best SNP (Lambert)	r^2^ (Harold)
***APOE***	19 (50)	5	<1.00E-10	5	<1.00E-10	rs8106922	rs8106922	1
***APOC1***	19 (50)	3	<1.00E-10	3	<1.00E-10	rs8106922	rs8106922	1
***CLU***	8 (28)	15	1.40E-09	14	5.19E-08	rs11136000	rs11136000	1
**APOC2**	19 (50)	4	3.43E-08	4	2.78E-03	rs5167	rs3760627	0.373
**APOC4**	19 (50)	4	3.43E-08	4	2.78E-03	rs5167	rs3760627	0.373
**ABCA7**	19 (1)	19	1.56E-05	17	4.24E-03	rs3764650	rs3764650	1
**ABCA1**	9 (107)	164	5.31E-05	169	1.30E-02	rs12686004	rs12336969	0.006
**ABCA12**	2 (216)	60	7.88E-05	62	1.43E-01	rs2225064	rs10206315	0.0002
**LIPC**	15 (57)	69	1.39E-04	64	6.34E-03	rs17269348	rs1077834	0.001
**ATP8A1**	4 (42)	64	1.82E-04	61	1.25E-01	rs3811769	rs9291220	0.105
**ATP8B4**	15 (48)	91	1.89E-04	86	1.69E-03	rs8041340	rs2009833	0.105
**MALL**	2 (110)	6	2.46E-04	3	4.37E-01	rs12998618	rs11240790	0.725
*ATP8A2*	13 (25)	154	1.06E-03	153	2.46E-04	rs3117849	rs10492697	0.001
**OSBPL7**	17 (43)	19	2.85E-04	19	3.07E-02	rs11079797	rs11652164	0.047
**SCARB1**	12 (124)	24	3.00E-04	25	6.94E-02	rs4765622	rs6488950	0.042
**VPS4B**	18 (59)	16	3.30E-04	15	1.89E-03	rs8094406	rs8091623	0.144
*ABCG1*	21 (42)	99	1.51E-03	95	4.64E-04	rs4148084	rs1044317	0.015
**LIPG**	18 (43)	19	5.20E-04	19	8.97E-03	rs12604221	rs2000813	0.046
**OSBPL9**	1 (59)	13	5.66E-04	12	3.81E-01	rs856614	rs1770791	0.005
*PCTP*	17 (51)	18	1.60E-02	19	6.01E-04	rs2960060	rs8079126	0.000
*SLC27A4*	9 (130)	7	8.19E-02	7	6.20E-04	rs3003600	rs7019382	0.028
*NPC1*	18 (19)	15	4.60E-02	15	6.25E-04	rs1808579	rs12970899	0.172
**APOA1**	11 (116)	6	7.62E-04	5	2.22E-01	rs12718464	rs509712	0.0002
**APOC3**	11 (116)	4	7.62E-04	3	2.68E-01	rs12718464	rs10047459	0.335
**APOA4**	11 (116)	4	7.62E-04	3	3.00E-01	rs12718464	rs1263167	0.0001
**AGTR1**	3 (149)	51	8.83E-04	50	1.32E-02	rs7647223	rs4681444	0.006
*SOAT1*	1 (177)	25	2.79E-02	25	9.94E-04	rs2492778	rs4652366	0.015

Genes included are those that have a SNP with p<0.001 in the Harold GWAS, and are in the lipid-related processes significantly enriched in both GWAS (Table 4). *APOC1*, *APOC2* and *APOC4* are not included in the enrichment analysis (Tables 3 and 4) since they are in LD with *APOE*. *APOA1* and *APOA4* share the same best SNP and are therefore counted as the same gene in the enrichment analyses. Two genes, *CLU* and *APOA4*, are found in both cholesterol and immune-related GO processes. The category-wide set-based analysis allows for such dependence between genes. Genes contributing to the enrichment signal from Harold et al. are in **bold**, genes contributing to the signal from Lambert et al. are in *italic* and genes contributing to the signal in both are in ***bold italic***.

**Table 6:** Genes with a SNP with p<0.001 in immune-related processes that are significantly enriched in both GWAS.

Gene Symbol	Chr location (Mb)	No. of SNPs (Harold)	Best p-value (Harold)	No. of SNPs (Lambert)	Best p-value (Lambert)	Best SNP (Harold)	Best SNP (Lambert)	r^2^ (Harold)
***BCL3***	19 (50)	6	<1.00E-10	6	1.90E-09	rs2927438	rs2965101	0.136
***CLU***	8 (28)	15	1.40E-09	14	5.19E-08	rs11136000	rs11136000	1
***CR1***	1 (206)	29	8.32E-06	29	1.03E-06	rs1408077	rs3818361	0.978
**IL1RAP**	3 (192)	50	1.26E-05	49	9.41E-03	rs4571225	rs6800609	0.004
**MS4A2**	11 (60)	11	5.74E-05	10	4.52E-02	rs540170	rs543695	0.447
*DEFB118*	20 (29)	5	2.73E-01	5	5.85E-05	rs6058963	rs17248462	0.021
*LILRA2*	19 (60)	8	2.81E-02	9	8.13E-05	rs11672845	rs2555691	0.003
*LILRA1*	19 (60)	8	1.14E-01	9	8.13E-05	rs10411879	rs2555691	0.026
*CHUK*	10 (102)	8	6.46E-03	7	9.00E-05	rs3818411	rs10883452	0.153
***HLA-DRB1***	6 (33)	18	1.55E-04	12	1.29E-04	rs660895	rs9269329	0.075
***CR2***	1 (206)	21	5.22E-04	21	2.10E-04	rs4317805	rs4310446	0.259
**CLNK**	4 (10)	62	2.72E-04	55	8.72E-02	rs2041216	rs10488945	0.193
*LILRB4*	19 (60)	22	1.87E-02	21	2.82E-04	rs1654668	rs1925241	0.050
*CHST4*	16 (70)	13	4.70E-02	12	3.02E-04	rs4149498	rs310334	0.185
**BTLA**	3 (113)	12	3.67E-04	11	6.85E-02	rs2171513	rs2705534	0.259
***HLA-DRA***	6 (33)	50	3.92E-04	45	4.63E-04	rs2395175	rs3135344	0.097
**IL18RAP**	2 (102)	15	3.94E-04	15	1.61E-02	rs2141781	rs2272128	0.275
*CPLX2*	5 (175)	48	3.18E-02	45	4.39E-04	rs17762082	rs2218891	0.149
**SERPINB4**	18 (59)	5	5.05E-04	5	6.44E-01	rs645623	rs3853683	0.028
**IL18R1**	2 (102)	16	5.42E-04	16	1.74E-02	rs4851004	rs13015714	0.629
**P2RY14**	3 (152)	14	5.47E-04	13	1.16E-01	rs10513391	rs9289834	0.080
*IL17A*	6 (52)	21	1.32E-02	21	5.55E-04	rs16882154	rs9395766	0.116
**TAP2**	6 (33)	97	5.64E-04	83	6.50E-03	rs1894406	rs4148870	0.001
**HLA-DOB**	6 (33)	75	5.64E-04	64	2.03E-03	rs1894406	rs7767167	0.002
**CFI**	4 (111)	18	5.85E-04	18	1.01E-01	rs2346841	rs4610335	0.011
**EXO1**	1 (240)	16	6.52E-04	19	9.65E-02	rs1776161	rs1776148	0.001
*HLA-DPA1*	6 (33)	45	6.03E-02	41	6.57E-04	rs11965964	rs2105929	0.008
*PAG1*	8 (82)	57	2.41E-03	56	7.20E-04	rs1445558	rs11778741	0.011
**CD300A**	17 (70)	14	7.23E-04	13	2.20E-01	rs4788839	rs1048367	0.106
**CXCL12**	10 (44)	18	7.41E-04	17	7.51E-03	rs2861442	rs2861442	1
**C9**	5 (39)	28	7.53E-04	27	3.38E-02	rs3776519	rs3733801	0.006
**GALNT2**	1 (228)	83	7.60E-04	79	1.02E-02	rs11122300	rs1474925	0.001
**APOA4**	11 (116)	4	7.62E-04	3	3.00E-01	rs12718464	rs1263167	0.001
**ICOSLG**	21 (44)	21	8.36E-04	19	2.21E-01	rs7278004	rs7283760	0.387
*IRF8*	16 (84)	39	7.22E-03	38	8.94E-04	rs11117425	rs419030	0.171
**IL1RL1**	2 (102)	22	9.02E-04	22	1.74E-02	rs10192157	rs13015714	0.181
**HLA-DQA1**	6 (33)	24	9.32E-04	14	6.62E-03	rs17533090	rs9272105	0.187
*HLA-DOA*	6 (33)	68	5.18E-03	67	9.73E-04	rs189984	rs9277015	0.027
**C1S**	12 (7)	6	9.73E-04	6	4.55E-03	rs7311672	rs11064498	0.652

Genes included are those that have a SNP with p<0.001 in the Harold GWAS, and are in the immune-related processes significantly enriched in both GWAS (Table 4). *BCL3* is not included in the enrichment analysis (Tables 3 and 4) since it is in LD with *APOE*. Two genes, *CLU* and *APOA4*, are found in both cholesterol and immune-related GO processes. *CR1* and *CR2* are at the same locus, as are *IL18RAP*, *IL18R1* and *IL1RL1* (see Table 3). Although they do not share the same best SNP, they may be tagging the same signal. The same applies to *HLA-DRB1*, *HLA-DRA*, *HLA-DOB*, *TAP2* and *HLA-DQA1*, which are all in the MHC region. The category-wide set-based analysis allows for such dependence between genes. Genes contributing to the enrichment signal from Harold et al. are in **bold**, genes contributing to the signal from Lambert et al. are in *italic* and genes contributing to the signal in both are in ***bold italic***.

Set-based and GSEA analysis was applied to each of the 25 GO processes with ALIGATOR p<0.05 in both the GWAS datasets (Table 4). GSEA analysis was applied in both Harold [7] and Lambert [8] datasets, while the set-based analysis was applied in the Harold dataset only (with the APOE region removed) since individual genotypes were not available in the Lambert dataset. Set-based analyses were also applied to the complete set of cholesterol-related genes in Table S6, and the complete set of immune-related genes in Table S7. The cholesterol-related genes gave a set-based p = 0.005, and the immune-related genes p = 0.005. After removing the SNPs giving rise to the GO signal (i.e. the most significant SNPs from the genes in Tables 5 and 6), the p-values are p = 0.009 and p = 0.007, respectively. This shows that the association signal in these genes is not restricted to a few highly-significant SNPs. GSEA analysis in the Harold dataset was significant for all of the processes except for GO:0007271 (synaptic transmission, cholinergic), with p-values very similar to that of the ALIGATOR analysis. In the Lambert dataset, all the immune-related pathways gave significant GSEA p-values, as did some of the lipid/cholesterol-related pathways. A pathway giving significant results in ALIGATOR but not in GSEA is likely due to the genes containing SNPs with p<0.001 being large (and thus subject to a stringent Simes correction), and the remaining genes showing little association evidence. In general, the set-based and GSEA analyses gave similar results to the ALIGATOR analyses, giving confidence that the results obtained by the latter reflect underlying biology.

## Discussion

Our analysis of two large independent GWAS of LOAD strongly implicates genetic variation in the functions of the immune system and in lipid metabolism as causes of LOAD susceptibility. A previous analysis of the Lambert et al. data [8], [24] highlighted similar biological processes despite not showing an overall excess of enriched GO categories. It highlights potential mechanisms related to these processes that should be the subject of further detailed genetic and functional investigations. This study has implications for the interpretation of GWAS of complex disease as it demonstrates that useful biological insights may be gained from association signals below the threshold for genome-wide significance, as previously shown for the WTCCC study [16], [25] where pathways known to be related to the diseases studied were highlighted by ALIGATOR. These analyses potentially highlight non-genome-wide significant SNPs that could explain some disease heritability which current GWAS do not have the power to detect.

The power of genetic data lies in their ability to highlight primary susceptibilities to disease, that is, they illuminate aetiology. This does not mean that all genes with a nominally significant SNP in an enriched GO category are true susceptibility genes for the phenotype under consideration, rather that that category itself is likely to be relevant to aetiology since it contains an excess of nominally associated SNPs. In this context, while the Harold [7] and Lambert [8] GWAS show a remarkable overlap in processes identified by ALIGATOR [16], the signal within each category did not necessarily reflect the same set of SNPs or genes. Tables 5 and 6 show that linkage disequilibrium between the most significant SNPs from each gene in the two GWAS varies from 1 (the same SNP) to none. In a pathway analysis this is perhaps unsurprising as there are several explanations for this observation. First, although we observe an excess of associated SNPs at all significance levels (Table 1), not all SNPs that surpass nominal significance can be expected to represent true associations. Second, in a set of genes that influence disease aetiology through a common biological pathway, it is likely that a number of SNPs will be associated with disease risk and affected individuals need not have the same combination of risk alleles. Individuals may have susceptibility alleles in different genes in a pathway or multiple rare susceptibility alleles may occur in a single gene; the latter will tend to be poorly tagged in GWAS. As a consequence even fairly large studies will have modest power to detect (or replicate between studies) *any one* signal, as compared with the power of tests based on the whole pathway. It is therefore noteworthy that the only non-immune and non-lipid related process detected in both studies was cholinergic synaptic transmission (Table 4); boosting cholinergic transmission is the target of one of the few available therapies for AD [26].

This analysis has limitations. We used categories curated in GO and KEGG databases and phenotypes annotated in the MGI database and will not have detected signal in functional processes not represented or well annotated by those systems. We chose to use GO and KEGG to define pathways since they are publicly available in a format that enables systematic testing of all pathways simultaneously in a statistically rigorous manner. The large number of GO categories increases the chance of alignment with the unknown disease biology underlying the GWAS results and the smaller number of results provided by the KEGG analysis supports this conclusion. The power to detect enrichment is highest for well-defined processes, and is greatly reduced if biologically important gene products are incorrectly or incompletely classified, or omitted. The quality of annotation in GO is variable, since some of it is inferred electronically, although there is some evidence that the majority of such annotations are correct [27]. However, enrichment analysis of an independent set of experimentally determined annotations, the MGI mouse phenotypes, highlighted the same biological processes, thus validating the GO results. The same analysis method applied to other diseases [16] found relevant biological pathways which were different to those presented here. Thus, the significance of these results is not simply due to the immune system and lipid metabolism being relatively well annotated. Furthermore, the ALIGATOR results were validated by applying GSEA and set-based analyses to the most significantly enriched pathways. These analyses produced similar results to ALIGATOR, giving confidence that the results obtained by ALIGATOR are genuine. This is supported by a direct analysis of SNPs in lipid-pathway genes in AD [28] which showed that more SNPs in lipid pathway genes than expected showed association with AD.

There are relatively few pathways highlighted by the KEGG analysis and this is likely due to the KEGG pathways including a more restricted range of biological processes than GO: while there are KEGG pathways relating to cholesterol and bile acid biosynthesis there are no pathways relating directly to lipid efflux from and transport between cells. Lambert et al. [24] detected an enrichment with the Alzheimer's disease KEGG pathway in a GSEA analysis. However, this enrichment is likely to have driven by the strong APOE association. We found significant enrichment of this pathway in the Lambert data when APOE was included, but not when it was removed. The KEGG pathways also tend to be large and the KEGG database does not have the hierarchical structure of the GO database that allows more specific functions to be defined. KEGG pathways with apparently similar names do not always contain similar genes to their corresponding GO categories. For example, KEGG pathway hsa4610 (complement and coagulation cascades) and GO:0006958 (complement activation, classical pathway) both relate to the complement cascade. However, hsa4610 also contains several genes that are not part of the complement cascade, making it larger than GO:0006958 (67 genes to 28) and reducing its significance in the enrichment analysis, since none of the extra genes have a SNP with p<0.001.

Cholesterol metabolism and innate immune processes have previously been implicated in AD pathogenesis [29], [30]. Epidemiological studies show that high cholesterol levels in mid-life are correlated with later dementia, and statins, which lower cholesterol levels, may have a protective effect against the development of dementia [31]. There have been trials and epidemiological surveys of the effects of anti-inflammatory treatment in AD which indicate that, although non-steroidal anti-inflammatories may have an effect on disease susceptibility, the drugs investigated so far are not a treatment for manifest disease [32]. Better targeted drugs to the parts of the immune system involved in AD susceptibility may offer new therapeutic avenues for research.

Although *APOE* was identified as a susceptibility factor for AD over 15 years ago [33], it is still not clear how the ε4 variant contributes to disease risk. The brain requires *de novo* cholesterol synthesis. This occurs in astrocytes and microglia, the cholesterol then being loaded into APOE lipoprotein particles and transported to the main cholesterol users, neurons and oligodendrocytes [34]. So while the impact of APOE is clearly of importance in AD, our data indicate that other participants in sterol metabolic processes also impact upon susceptibility. It is notable that some of these genes are not expressed in the brain, for instance *LIPC*, *APOA1*, *SCARB1* and *LIPG*, but are important in the systemic control of sterol metabolism in the liver and blood. Some of these gene products may well be useful in providing clues for possible systemic biomarkers of disease progress.

APOE has been implicated in Aβ clearance. The lipidation state of APOE is critical to its ability to transport Aβ across the BBB, APOE4 being associated with the least efficient transport [35]. Aβ in the blood is transported in cholesterol-rich HDL particles, which have ApoA1 or ApoE as associated lipoproteins, before elimination by the liver [36]. Our data suggest that the role of APOE in cholesterol metabolism is important in AD, and may implicate the systemic clearance of Aβ-HDL through the liver, in which APOE is certainly involved, as a primary modulator of AD susceptibility [36], [37]. *CLU*, encoding APOJ, is associated with cholesterol transport and has been demonstrated to promote export of Aβ over the BBB [38] and thus may modulate Aβ clearance from the brain in concert with APOE.

Apart from the *APOE* locus, *CLU*, which encodes the complement activation inhibitor clusterin and *CR1* which encodes complement receptor 1 both contain genome-wide significant signals and are involved in the innate immune response [7], [8]. The set of immune-related genes remained significantly associated (set-based p-value 0.006) after the removal of *CLU*. Complement components have been detected in AD amyloid plaques [37] and fibrillar APP activates complement pathways. The phagocytotic action of both microglia and blood-derived macrophages has been implicated in Aβ clearance [38]. However, until now, these observations have been considered to be consequences of disease pathology because activation of microglia, the resident immune cells of the brain, can result from neurodegeneration [39].

Our data suggest that the primary causes of LOAD include genetic variation in cholesterol metabolism and the innate immune system. They also indicate that common variation in genes directly related to Aβ metabolism does not underlie individual differences in susceptibility to LOAD. Nevertheless these findings do not exclude a central role for the amyloid cascade [5] in pathogenesis, and indeed, both processes highlighted by our analysis have been implicated in Aβ clearance in the brain [40] though further work is required to determine whether the risk these processes confer is mediated solely or in part through Aβ and whether they impact on risk via other mechanisms. Importantly both processes represent modifiable risk factors that might be addressed by drugs already in our armoury.

## Supporting Information

Table S1 Gene ontology categories identified by ALIGATOR analysis of the AD GWA data of Harold and colleagues (7). The 589 genes identified as having GWAS SNP signals p<0.001 were used: APOE was included in the gene list. In this analysis one SNP was not allowed to add more than one gene to any gene ontology category. “Study-wide p-value” is the probability of obtaining by chance at least one GO category with a category-specific enrichment p-value at least as significant as that observed.
(0.11 MB PDF)

Table S2 Gene ontology categories identified by ALIGATOR analysis of the AD GWA data of Lambert and colleagues. The 423 genes identified as having GWAS SNP signals p<0.001 from Lambert et al. (8)were used: APOE was included in the gene list. In this analysis one SNP was not allowed to add more than one gene to any gene ontology category. “Study-wide p-value” is the probability of obtaining by chance at least one GO category with a category-specific enrichment p-value at least as significant as that observed.
(0.08 MB PDF)

Table S3 List of KEGG categories significantly (p<0.05) enriched in both GWAS. “Joint p” is the probability of observing by chance at least one category among the entire set of categories tested with joint enrichment (defined as the product of enrichment p-values from the two GWAS) at least as extreme as that observed in the real data. This corrects for the multiple non-independent GO categories being tested.
(0.00 MB PDF)

Table S4 All genes in the KEGG immune-related categories in Table S3. “Best p (corrected)” is the significance of the best single-SNP p-value corrected for testing multiple SNPs in a gene (allowing for LD between SNPs). “Set based p” refers to a test of whether the average single-SNP chi-squared (allelic) association statistic is significantly high (again allowing for LD between SNPs).
(0.01 MB PDF)

Table S5 MGI mouse phenotypes identified by ALIGATOR analysis of the AD GWA data of Harold and colleagues. The 589 genes identified as having GWAS SNP signals p<0·001 were used: APOE was included in the gene list. In this analysis one SNP was not allowed to add more than one gene to any phenotype. “Study-wide p-value” is the probability of obtaining by chance at least one mouse phenotype with a phenotype-specific enrichment p-value at least as significant as that observed.
(0.08 MB PDF)

Table S6 All genes in the cholesterol and lipid categories in Table 5. “Best p (corrected)” is the significance of the best single-SNP p-value corrected for testing multiple SNPs in a gene (allowing for LD between SNPs). “Set based p” refers to a test of whether the average single-SNP chi-squared (allelic) association statistic is significantly high (again allowing for LD between SNPs).
(0.08 MB PDF)

Table S7 All genes in the immune-related categories in Table 6. “Best p (corrected)” is the significance of the best single-SNP p-value corrected for testing multiple SNPs in a gene (allowing for LD between SNPs). “Set based p” refers to a test of whether the average single-SNP chi-squared (allelic) association statistic is significantly high (again allowing for LD between SNPs).
(0.04 MB PDF)
