# Correction: Genetic Evidence Implicates the Immune System and Cholesterol Metabolism in the Aetiology of Alzheimer's Disease

**DOI:** 10.1371/annotation/a0bb886d-d345-4a20-a82e-adce9b047798

**Published:** 2011-02-11

**Authors:** Lesley Jones, Peter A. Holmans, Marian L. Hamshere, Denise Harold, Valentina Moskvina, Dobril Ivanov, Andrew Pocklington, Richard Abraham, Paul Hollingworth, Rebecca Sims, Amy Gerrish, Jaspreet Singh Pahwa, Nicola Jones, Alexandra Stretton, Angharad R. Morgan, Simon Lovestone, John Powell, Petroula Proitsi, Michelle K. Lupton, Carol Brayne, David C. Rubinsztein, Michael Gill, Brian Lawlor, Aoibhinn Lynch, Kevin Morgan, Kristelle S. Brown, Peter A. Passmore, David Craig, Bernadette McGuinness, Stephen Todd, Clive Holmes, David Mann, A. David Smith, Seth Love, Patrick G. Kehoe, Simon Mead, Nick Fox, Martin Rossor, John Collinge, Wolfgang Maier, Frank Jessen, Britta Schürmann, Hendrik van den Bussche, Isabella Heuser, Oliver Peters, Johannes Kornhuber, Jens Wiltfang, Martin Dichgans, Lutz Frölich, Harald Hampel, Michael Hüll, Dan Rujescu, Alison M. Goate, John S. K. Kauwe, Carlos Cruchaga, Petra Nowotny, John C. Morris, Kevin Mayo, Gill Livingston, Nicholas J. Bass, Hugh Gurling, Andrew McQuillin, Rhian Gwilliam, Panos Deloukas, Ammar Al-Chalabi, Christopher E. Shaw, Andrew B. Singleton, Rita Guerreiro, Thomas W. Mühleisen, Markus M. Nöthen, Susanne Moebus, Karl-Heinz Jöckel, Norman Klopp, H.-Erich Wichmann, Eckhard Rüther, Minerva M. Carrasquillo, V. Shane Pankratz, Steven G. Younkin, John Hardy, Michael C. O'Donovan, Michael J. Owen, Julie Williams

The authors would like to add Reinhard Heun and Heike Kölsch to the byline. The updated byline is: Lesley Jones, Peter A. Holmans, Marian L. Hamshere, Denise Harold, Valentina Moskvina, Dobril Ivanov, Andrew Pocklington, Richard Abraham, Paul Hollingworth, Rebecca Sims, Amy Gerrish, Jaspreet Singh Pahwa, Nicola Jones, Alexandra Stretton, Angharad R. Morgan, Simon Lovestone, John Powell, Petroula Proitsi, Michelle K. Lupton, Carol Brayne, David C. Rubinsztein, Michael Gill, Brian Lawlor, Aoibhinn Lynch, Kevin Morgan, Kristelle S. Brown, Peter A. Passmore, David Craig Bernadette McGuinness, Stephen Todd, Clive Holmes, David Mann, A. David Smith, Seth Love, Patrick G. Kehoe, Simon Mead, Nick Fox, Martin Rossor, John Collinge, Wolfgang Maier, Frank Jessen, Britta Schürmann, Reinhard Heun , Heike Kölsch, Hendrik van den Bussche, Isabella Heuser, Oliver Peters, Johannes Kornhuber, Jens Wiltfang, Martin Dichgans, Lutz Frölich, Harald Hampel, Michael Hüll, Dan Rujescu, Alison M. Goate, John S. K. Kauwe, Carlos Cruchaga, Petra Nowotny, John C. Morris, Kevin Mayo, Gill Livingston, Nicholas J. Bass, Hugh Gurling, Andrew McQuillin, Rhian Gwilliam, Panos Deloukas, Ammar Al-Chalabi Christopher E. Shaw, Andrew B. Singleton, Rita Guerreiro, Thomas W. Mühleisen, Markus M. Nöthen, Susanne Moebus, Karl-Heinz Jöckel, Norman Klopp, H.-Erich Wichmann, Eckhard Rüther, Minerva M. Carrasquillo, V. Shane Pankratz, Steven G. Younkin, John Hardy, Michael C. O'Donovan, Michael J. Owen, Julie Williams

