# Supplementary material for: Genetic Evidence Implicates the Immune System and Cholesterol Metabolism in the Aetiology of Alzheimer's Disease
Source: PLoS One. 2010 Nov 15;5(11):e13950. doi: 10.1371/journal.pone.0013950 (PMC2981526; doi:10.1371/journal.pone.0013950)
Supplement: Table S1 — Gene ontology categories identified by ALIGATOR analysis of the AD GWA data of Harold and colleagues (7). The 589 genes identified as having GWAS SNP signals p<0.001 were used: APOE was included in the gene list. In this analysis one SNP was not allowed to add more than one gene to any gene ontology category. “Study-wide p-value” is the probability of obtaining by chance at least one GO category with a category-specific enrichment p-value at least as significant as that observed. (0.11 MB PDF) [file pone.0013950.s001.pdf]

**Table S1 Gene ontology categories identified by ALIGATOR analysis of the AD GWA data of Harold and colleagues.[1]**

The 589 genes identified as having GWAS SNP signals p < 0.001 were used: APOE was included in the gene list. In this analysis one SNP was not allowed to add more than one gene to any gene ontology category. “Study-wide p-value” is the probability of obtaining by chance at least one GO category with a category-specific enrichment p-value at least as significant as that observed.

| GO process | Type | total genes in category | #genes on list | p-value | Study-wide corrected p-value | Function |
|---|---|---|---|---|---|---|
| GO:0032488 | PROCESS | 4 | 3 | <1.00E-05 | 0.042 | Cdc42 protein signal transduction |
| GO:0033700 | PROCESS | 8 | 4 | <1.00E-05 | 0.042 | phospholipid efflux |
| GO:0043691 | PROCESS | 14 | 7 | <1.00E-05 | 0.042 | reverse cholesterol transport |
| GO:0030301 | PROCESS | 34 | 8 | <1.00E-05 | 0.042 | cholesterol transport |
| GO:0015918 | PROCESS | 34 | 8 | <1.00E-05 | 0.042 | sterol transport |
| GO:0034369 | PROCESS | 18 | 6 | <1.00E-05 | 0.042 | plasma lipoprotein particle remodeling |
| GO:0034368 | PROCESS | 18 | 6 | <1.00E-05 | 0.042 | protein-lipid complex remodeling |
| GO:0034367 | PROCESS | 18 | 6 | <1.00E-05 | 0.042 | macromolecular complex remodeling |
| GO:0034375 | PROCESS | 11 | 5 | <1.00E-05 | 0.042 | high-density lipoprotein particle remodeling |
| GO:0034382 | PROCESS | 3 | 3 | <1.00E-05 | 0.042 | chylomicron remnant clearance |
| GO:0016125 | PROCESS | 87 | 11 | 2.00E-05 | 0.066 | sterol metabolic process |
| GO:0034364 | CELLULAR | 20 | 5 | 2.00E-05 | 0.066 | high-density lipoprotein particle |
| GO:0022411 | PROCESS | 55 | 8 | 2.00E-05 | 0.066 | cellular component disassembly |
| GO:0006958 | PROCESS | 28 | 6 | 2.00E-05 | 0.066 | complement activation, classical pathway |
| GO:0002455 | PROCESS | 28 | 6 | 2.00E-05 | 0.066 | humoral immune response mediated by circulating immunoglobulin |
| GO:0042632 | PROCESS | 33 | 7 | 4.00E-05 | 0.093 | cholesterol homeostasis |
| GO:0055092 | PROCESS | 33 | 7 | 4.00E-05 | 0.093 | sterol homeostasis |
| GO:0006956 | PROCESS | 37 | 6 | 4.00E-05 | 0.093 | complement activation |
| GO:0002541 | PROCESS | 38 | 6 | 4.00E-05 | 0.093 | activation of plasma proteins involved in acute inflammatory response |
| GO:0045087 | PROCESS | 120 | 11 | 6.00E-05 | 0.113 | innate immune response |
| GO:0008203 | PROCESS | 78 | 10 | 8.00E-05 | 0.129 | cholesterol metabolic process |
| GO:0034384 | PROCESS | 5 | 3 | 1.00E-04 | 0.146 | high-density lipoprotein particle clearance |
| GO:0032984 | PROCESS | 34 | 6 | 1.40E-04 | 0.192 | macromolecular complex disassembly |
| GO:0032376 | PROCESS | 7 | 3 | 1.80E-04 | 0.226 | positive regulation of cholesterol transport |
| GO:0032373 | PROCESS | 7 | 3 | 1.80E-04 | 0.226 | positive regulation of sterol transport |
| GO:0010873 | PROCESS | 6 | 3 | 2.00E-04 | 0.250 | positive regulation of cholesterol esterification |
| GO:0030001 | PROCESS | 410 | 36 | 2.20E-04 | 0.279 | metal ion transport |
| GO:0034372 | PROCESS | 6 | 3 | 2.20E-04 | 0.279 | very-low-density lipoprotein particle remodeling |
| GO:0008202 | PROCESS | 174 | 15 | 2.40E-04 | 0.294 | steroid metabolic process |
| GO:0055088 | PROCESS | 43 | 7 | 2.60E-04 | 0.316 | lipid homeostasis |
| GO:0006869 | PROCESS | 131 | 14 | 2.80E-04 | 0.336 | lipid transport |
| GO:0034366 | CELLULAR | 7 | 3 | 2.80E-04 | 0.336 | spherical high-density lipoprotein particle |
| GO:0034363 | CELLULAR | 3 | 2 | 2.80E-04 | 0.336 | intermediate-density lipoprotein particle |
| GO:0006812 | PROCESS | 494 | 38 | 3.60E-04 | 0.404 | cation transport |
| GO:0060228 | FUNCTION | 4 | 2 | 3.60E-04 | 0.404 | phosphatidylcholine-sterol O-acyltransferase activator activity |
| GO:0032370 | PROCESS | 9 | 3 | 3.60E-04 | 0.404 | positive regulation of lipid transport |
| GO:0032374 | PROCESS | 17 | 4 | 4.40E-04 | 0.455 | regulation of cholesterol transport |
| GO:0032371 | PROCESS | 17 | 4 | 4.40E-04 | 0.455 | regulation of sterol transport |
| GO:0010876 | PROCESS | 142 | 14 | 4.80E-04 | 0.487 | lipid localization |
| GO:0016485 | PROCESS | 95 | 10 | 4.80E-04 | 0.487 | protein processing |
| GO:0016064 | PROCESS | 44 | 6 | 4.80E-04 | 0.487 | immunoglobulin mediated immune response |
| GO:0034370 | PROCESS | 8 | 3 | 5.00E-04 | 0.505 | triglyceride-rich lipoprotein particle remodeling |
| GO:0019724 | PROCESS | 46 | 6 | 5.80E-04 | 0.549 | B cell mediated immunity |
| GO:0032395 | FUNCTION | 8 | 4 | 5.80E-04 | 0.549 | MHC class II receptor activity |
| GO:0043933 | PROCESS | 512 | 25 | 6.00E-04 | 0.563 | macromolecular complex subunit organization |
| GO:0015672 | PROCESS | 291 | 25 | 6.00E-04 | 0.563 | monovalent inorganic cation transport |
| GO:0034358 | CELLULAR | 30 | 5 | 6.40E-04 | 0.580 | plasma lipoprotein particle |
| GO:0032994 | CELLULAR | 30 | 5 | 6.40E-04 | 0.580 | protein-lipid complex |
| GO:0034381 | PROCESS | 14 | 4 | 6.40E-04 | 0.580 | lipoprotein particle clearance |
| GO:0070328 | PROCESS | 6 | 3 | 6.40E-04 | 0.580 | triglyceride homeostasis |
| GO:0051604 | PROCESS | 104 | 10 | 7.20E-04 | 0.618 | protein maturation |
| GO:0034380 | PROCESS | 5 | 3 | 7.60E-04 | 0.635 | high-density lipoprotein particle assembly |
| GO:0048584 | PROCESS | 167 | 13 | 8.00E-04 | 0.648 | positive regulation of response to stimulus |
| GO:0044242 | PROCESS | 80 | 8 | 8.60E-04 | 0.671 | cellular lipid catabolic process |
| GO:0050746 | PROCESS | 7 | 3 | 8.80E-04 | 0.675 | regulation of lipoprotein metabolic process |
| GO:0016042 | PROCESS | 155 | 12 | 9.00E-04 | 0.681 | lipid catabolic process |
| GO:0002526 | PROCESS | 74 | 7 | 9.40E-04 | 0.700 | acute inflammatory response |
| GO:0004875 | FUNCTION | 3 | 2 | 1.12E-03 | 0.757 | complement receptor activity |
| GO:0006707 | PROCESS | 9 | 3 | 1.14E-03 | 0.759 | cholesterol catabolic process |
| GO:0016127 | PROCESS | 9 | 3 | 1.14E-03 | 0.759 | sterol catabolic process |
| GO:0010872 | PROCESS | 7 | 3 | 1.20E-03 | 0.772 | regulation of cholesterol esterification |
| GO:0006955 | PROCESS | 576 | 25 | 1.26E-03 | 0.792 | immune response |
| GO:0006959 | PROCESS | 68 | 7 | 1.30E-03 | 0.804 | humoral immune response |
| GO:0002253 | PROCESS | 66 | 7 | 1.32E-03 | 0.809 | activation of immune response |
| GO:0042613 | CELLULAR | 12 | 4 | 1.88E-03 | 0.912 | MHC class II protein complex |
| GO:0004908 | FUNCTION | 6 | 3 | 1.88E-03 | 0.912 | interleukin-1 receptor activity |
| GO:0002449 | PROCESS | 56 | 6 | 2.00E-03 | 0.921 | lymphocyte mediated immunity |
| GO:0008324 | FUNCTION | 493 | 35 | 2.18E-03 | 0.937 | cation transmembrane transporter activity |
| GO:0002504 | PROCESS | 15 | 4 | 2.32E-03 | 0.952 | antigen processing and presentation of peptide or polysaccharide antigen via MHC class II |

| | | | | | | |
|---|---|---|---|---|---|---|
| GO:0002460 | PROCESS | 61 | 6 | 2.36E-03 | 0.953 | adaptive immune response based on somatic recombination of immune receptors built from immunoglobulin superfamily domains |
| GO:0002250 | PROCESS | 61 | 6 | 2.36E-03 | 0.953 | adaptive immune response |
| GO:0032368 | PROCESS | 25 | 4 | 2.46E-03 | 0.960 | regulation of lipid transport |
| GO:0006811 | PROCESS | 690 | 45 | 2.48E-03 | 0.961 | ion transport |
| GO:0001836 | PROCESS | 16 | 3 | 2.58E-03 | 0.963 | release of cytochrome c from mitochondria |
| GO:0004872 | FUNCTION | 1518 | 67 | 2.74E-03 | 0.971 | receptor activity |
| GO:0010984 | PROCESS | 4 | 2 | 2.78E-03 | 0.973 | regulation of lipoprotein particle clearance |
| GO:0051605 | PROCESS | 65 | 7 | 2.82E-03 | 0.974 | protein maturation by peptide bond cleavage |
| GO:0042592 | PROCESS | 545 | 29 | 3.02E-03 | 0.980 | homeostatic process |
| GO:0019966 | FUNCTION | 9 | 3 | 3.16E-03 | 0.981 | interleukin-1 binding |
| GO:0034377 | PROCESS | 11 | 3 | 3.18E-03 | 0.981 | plasma lipoprotein particle assembly |
| GO:0065005 | PROCESS | 11 | 3 | 3.18E-03 | 0.981 | protein-lipid complex assembly |
| GO:0010875 | PROCESS | 6 | 2 | 3.54E-03 | 0.984 | positive regulation of cholesterol efflux |
| GO:0048878 | PROCESS | 382 | 23 | 3.66E-03 | 0.986 | chemical homeostasis |
| GO:0002376 | PROCESS | 811 | 32 | 3.68E-03 | 0.986 | immune system process |
| GO:0004871 | FUNCTION | 1932 | 83 | 3.90E-03 | 0.991 | signal transducer activity |
| GO:0060089 | FUNCTION | 1932 | 83 | 3.90E-03 | 0.991 | molecular transducer activity |
| GO:0034443 | PROCESS | 3 | 2 | 3.92E-03 | 0.991 | negative regulation of lipoprotein oxidation |
| GO:0034442 | PROCESS | 3 | 2 | 3.92E-03 | 0.991 | regulation of lipoprotein oxidation |
| GO:0007266 | PROCESS | 32 | 5 | 3.98E-03 | 0.991 | Rho protein signal transduction |
| GO:0002443 | PROCESS | 66 | 6 | 4.10E-03 | 0.991 | leukocyte mediated immunity |
| GO:0031420 | FUNCTION | 222 | 20 | 4.40E-03 | 0.994 | alkali metal ion binding |
| GO:0042176 | PROCESS | 59 | 6 | 4.48E-03 | 0.994 | regulation of protein catabolic process |
| GO:0015075 | FUNCTION | 649 | 43 | 4.86E-03 | 0.996 | ion transmembrane transporter activity |
| GO:0022857 | FUNCTION | 818 | 50 | 4.92E-03 | 0.996 | transmembrane transporter activity |
| GO:0016126 | PROCESS | 30 | 4 | 5.26E-03 | 0.996 | sterol biosynthetic process |
| GO:0031402 | FUNCTION | 113 | 12 | 5.34E-03 | 0.996 | sodium ion binding |
| GO:0004806 | FUNCTION | 15 | 3 | 5.36E-03 | 0.996 | triglyceride lipase activity |
| GO:0005261 | FUNCTION | 252 | 23 | 5.52E-03 | 0.997 | cation channel activity |
| GO:0051000 | PROCESS | 7 | 2 | 5.60E-03 | 0.997 | positive regulation of nitric-oxide synthase activity |
| GO:0051702 | PROCESS | 7 | 2 | 5.72E-03 | 0.997 | interaction with symbiont |
| GO:0005215 | FUNCTION | 1074 | 57 | 6.40E-03 | 0.999 | transporter activity |
| GO:0005249 | FUNCTION | 94 | 10 | 6.94E-03 | 1.000 | voltage-gated potassium channel activity |
| GO:0002684 | PROCESS | 164 | 10 | 7.16E-03 | 1.000 | positive regulation of immune system process |
| GO:0006706 | PROCESS | 16 | 3 | 7.16E-03 | 1.000 | steroid catabolic process |
| GO:0006810 | PROCESS | 2330 | 96 | 7.26E-03 | 1.000 | transport |
| GO:0045940 | PROCESS | 11 | 3 | 7.50E-03 | 1.000 | positive regulation of steroid metabolic process |
| GO:0008637 | PROCESS | 24 | 3 | 7.56E-03 | 1.000 | apoptotic mitochondrial changes |
| GO:0000300 | CELLULAR | 3 | 2 | 7.70E-03 | 1.000 | peripheral to membrane of membrane fraction |
| GO:0008633 | PROCESS | 21 | 3 | 7.80E-03 | 1.000 | activation of pro-apoptotic gene products |
| GO:0048583 | PROCESS | 332 | 17 | 8.04E-03 | 1.000 | regulation of response to stimulus |
| GO:0034186 | FUNCTION | 3 | 2 | 8.06E-03 | 1.000 | apolipoprotein A-I binding |
| GO:0046873 | FUNCTION | 296 | 25 | 8.06E-03 | 1.000 | metal ion transmembrane transporter activity |
| GO:0006813 | PROCESS | 146 | 14 | 8.10E-03 | 1.000 | potassium ion transport |
| GO:0051055 | PROCESS | 10 | 2 | 8.12E-03 | 1.000 | negative regulation of lipid biosynthetic process |
| GO:0010874 | PROCESS | 7 | 2 | 8.18E-03 | 1.000 | regulation of cholesterol efflux |
| GO:0022836 | FUNCTION | 284 | 25 | 8.20E-03 | 1.000 | gated channel activity |
| GO:0006814 | PROCESS | 120 | 12 | 8.74E-03 | 1.000 | sodium ion transport |
| GO:0006694 | PROCESS | 71 | 6 | 8.92E-03 | 1.000 | steroid biosynthetic process |
| GO:0008076 | CELLULAR | 80 | 9 | 8.92E-03 | 1.000 | voltage-gated potassium channel complex |
| GO:0034705 | CELLULAR | 80 | 9 | 8.92E-03 | 1.000 | potassium channel complex |
| GO:0015914 | PROCESS | 28 | 6 | 8.96E-03 | 1.000 | phospholipid transport |
| GO:0006695 | PROCESS | 21 | 3 | 8.96E-03 | 1.000 | cholesterol biosynthetic process |
| GO:0009593 | PROCESS | 29 | 5 | 9.18E-03 | 1.000 | detection of chemical stimulus |
| GO:0002252 | PROCESS | 95 | 7 | 9.42E-03 | 1.000 | immune effector process |
| GO:0051234 | PROCESS | 2352 | 96 | 9.60E-03 | 1.000 | establishment of localization |
| GO:0030162 | PROCESS | 41 | 4 | 9.66E-03 | 1.000 | regulation of proteolysis |
| GO:0046470 | PROCESS | 16 | 3 | 9.66E-03 | 1.000 | phosphatidylcholine metabolic process |
| GO:0050778 | PROCESS | 93 | 7 | 1.00E-02 | 1.000 | positive regulation of immune response |
| GO:0042627 | CELLULAR | 10 | 2 | 1.01E-02 | 1.000 | chylomicron |
| GO:0005267 | FUNCTION | 124 | 12 | 1.01E-02 | 1.000 | potassium channel activity |
| GO:0009894 | PROCESS | 91 | 7 | 1.03E-02 | 1.000 | regulation of catabolic process |
| GO:0034703 | CELLULAR | 123 | 13 | 1.04E-02 | 1.000 | cation channel complex |
| GO:0001772 | CELLULAR | 7 | 2 | 1.07E-02 | 1.000 | immunological synapse |
| GO:0065003 | PROCESS | 465 | 19 | 1.11E-02 | 1.000 | macromolecular complex assembly |
| GO:0033017 | CELLULAR | 13 | 4 | 1.18E-02 | 1.000 | sarcoplasmic reticulum membrane |
| GO:0031232 | CELLULAR | 4 | 2 | 1.23E-02 | 1.000 | extrinsic to external side of plasma membrane |
| GO:0001848 | FUNCTION | 8 | 2 | 1.26E-02 | 1.000 | complement binding |
| GO:0034185 | FUNCTION | 10 | 3 | 1.26E-02 | 1.000 | apolipoprotein binding |
| GO:0009896 | PROCESS | 44 | 4 | 1.33E-02 | 1.000 | positive regulation of catabolic process |
| GO:0051044 | PROCESS | 9 | 2 | 1.36E-02 | 1.000 | positive regulation of membrane protein ectodomain proteolysis |
| GO:0017127 | FUNCTION | 12 | 3 | 1.42E-02 | 1.000 | cholesterol transporter activity |
| GO:0046661 | PROCESS | 45 | 4 | 1.42E-02 | 1.000 | male sex differentiation |
| GO:0015271 | FUNCTION | 4 | 2 | 1.45E-02 | 1.000 | outward rectifier potassium channel activity |
| GO:0022843 | FUNCTION | 134 | 13 | 1.47E-02 | 1.000 | voltage-gated cation channel activity |
| GO:0050748 | PROCESS | 6 | 2 | 1.51E-02 | 1.000 | negative regulation of lipoprotein metabolic process |
| GO:0022891 | FUNCTION | 751 | 44 | 1.57E-02 | 1.000 | substrate-specific transmembrane transporter activity |
| GO:0045732 | PROCESS | 25 | 3 | 1.57E-02 | 1.000 | positive regulation of protein catabolic process |

| | | | | | | |
|---|---|---|---|---|---|---|
| GO:0007271 | PROCESS | 13 | 2 | 1.57E-02 | 1.000 | synaptic transmission, cholinergic |
| GO:0005892 | CELLULAR | 13 | 2 | 1.58E-02 | 1.000 | nicotinic acetylcholine-gated receptor-channel complex |
| GO:0022892 | FUNCTION | 880 | 48 | 1.61E-02 | 1.000 | substrate-specific transporter activity |
| GO:0009056 | PROCESS | 1484 | 50 | 1.61E-02 | 1.000 | catabolic process |
| GO:0034702 | CELLULAR | 187 | 16 | 1.66E-02 | 1.000 | ion channel complex |
| GO:0015267 | FUNCTION | 373 | 28 | 1.66E-02 | 1.000 | channel activity |
| GO:0022803 | FUNCTION | 373 | 28 | 1.66E-02 | 1.000 | passive transmembrane transporter activity |
| GO:0019433 | PROCESS | 6 | 2 | 1.84E-02 | 1.000 | triglyceride catabolic process |
| GO:0004091 | FUNCTION | 91 | 6 | 1.90E-02 | 1.000 | carboxylesterase activity |
| GO:0016459 | CELLULAR | 62 | 7 | 1.93E-02 | 1.000 | myosin complex |
| GO:0060155 | PROCESS | 4 | 2 | 1.94E-02 | 1.000 | platelet dense granule organization |
| GO:0015248 | FUNCTION | 14 | 3 | 1.98E-02 | 1.000 | sterol transporter activity |
| GO:0051353 | PROCESS | 22 | 3 | 1.98E-02 | 1.000 | positive regulation of oxidoreductase activity |
| GO:0032770 | PROCESS | 11 | 2 | 2.00E-02 | 1.000 | positive regulation of monooxygenase activity |
| GO:0004620 | FUNCTION | 80 | 7 | 2.08E-02 | 1.000 | phospholipase activity |
| GO:0003950 | FUNCTION | 25 | 3 | 2.12E-02 | 1.000 | NAD+ ADP-ribosyltransferase activity |
| GO:0012502 | PROCESS | 228 | 12 | 2.20E-02 | 1.000 | induction of programmed cell death |
| GO:0006874 | PROCESS | 127 | 9 | 2.26E-02 | 1.000 | cellular calcium ion homeostasis |
| GO:0002682 | PROCESS | 280 | 13 | 2.30E-02 | 1.000 | regulation of immune system process |
| GO:0004889 | FUNCTION | 16 | 2 | 2.34E-02 | 1.000 | nicotinic acetylcholine-activated cation-selective channel activity |
| GO:0005216 | FUNCTION | 355 | 27 | 2.34E-02 | 1.000 | ion channel activity |
| GO:0046464 | PROCESS | 7 | 2 | 2.34E-02 | 1.000 | acylglycerol catabolic process |
| GO:0044269 | PROCESS | 7 | 2 | 2.34E-02 | 1.000 | glycerol ether catabolic process |
| GO:0046461 | PROCESS | 7 | 2 | 2.34E-02 | 1.000 | neutral lipid catabolic process |
| GO:0008238 | FUNCTION | 72 | 7 | 2.39E-02 | 1.000 | exopeptidase activity |
| GO:0055074 | PROCESS | 130 | 9 | 2.41E-02 | 1.000 | calcium ion homeostasis |
| GO:0030005 | PROCESS | 163 | 10 | 2.48E-02 | 1.000 | cellular di-, tri-valent inorganic cation homeostasis |
| GO:0009395 | PROCESS | 19 | 3 | 2.53E-02 | 1.000 | phospholipid catabolic process |
| GO:0042802 | FUNCTION | 517 | 25 | 2.61E-02 | 1.000 | identical protein binding |
| GO:0005543 | FUNCTION | 153 | 11 | 2.63E-02 | 1.000 | phospholipid binding |
| GO:0033344 | PROCESS | 15 | 3 | 2.68E-02 | 1.000 | cholesterol efflux |
| GO:0004181 | FUNCTION | 25 | 4 | 2.69E-02 | 1.000 | metallocarboxypeptidase activity |
| GO:0022838 | FUNCTION | 365 | 27 | 2.74E-02 | 1.000 | substrate-specific channel activity |
| GO:0005184 | FUNCTION | 19 | 2 | 2.77E-02 | 1.000 | neuropeptide hormone activity |
| GO:0005244 | FUNCTION | 177 | 15 | 2.77E-02 | 1.000 | voltage-gated ion channel activity |
| GO:0022832 | FUNCTION | 177 | 15 | 2.77E-02 | 1.000 | voltage-gated channel activity |
| GO:0006629 | PROCESS | 733 | 30 | 2.81E-02 | 1.000 | lipid metabolic process |
| GO:0007218 | PROCESS | 81 | 7 | 2.85E-02 | 1.000 | neuropeptide signaling pathway |
| GO:0016763 | FUNCTION | 45 | 4 | 2.91E-02 | 1.000 | transferase activity, transferring pentosyl groups |
| GO:0016455 | FUNCTION | 27 | 3 | 2.92E-02 | 1.000 | RNA polymerase II transcription mediator activity |
| GO:0055066 | PROCESS | 173 | 10 | 2.94E-02 | 1.000 | di-, tri-valent inorganic cation homeostasis |
| GO:0050801 | PROCESS | 301 | 17 | 2.96E-02 | 1.000 | ion homeostasis |
| GO:0031202 | FUNCTION | 24 | 2 | 2.97E-02 | 1.000 | RNA splicing factor activity, transesterification mechanism |
| GO:0050776 | PROCESS | 152 | 8 | 2.98E-02 | 1.000 | regulation of immune response |
| GO:0032312 | PROCESS | 27 | 3 | 3.01E-02 | 1.000 | regulation of ARF GTPase activity |
| GO:0007159 | PROCESS | 21 | 3 | 3.11E-02 | 1.000 | leukocyte adhesion |
| GO:0042611 | CELLULAR | 34 | 4 | 3.16E-02 | 1.000 | MHC protein complex |
| GO:0007548 | PROCESS | 98 | 6 | 3.18E-02 | 1.000 | sex differentiation |
| GO:0008060 | FUNCTION | 28 | 3 | 3.20E-02 | 1.000 | ARF GTPase activator activity |
| GO:0001942 | PROCESS | 26 | 3 | 3.21E-02 | 1.000 | hair follicle development |
| GO:0022405 | PROCESS | 26 | 3 | 3.21E-02 | 1.000 | hair cycle process |
| GO:0022404 | PROCESS | 26 | 3 | 3.21E-02 | 1.000 | molting cycle process |
| GO:0052548 | PROCESS | 68 | 5 | 3.28E-02 | 1.000 | regulation of endopeptidase activity |
| GO:0070325 | FUNCTION | 15 | 2 | 3.30E-02 | 1.000 | lipoprotein receptor binding |
| GO:0006919 | PROCESS | 45 | 4 | 3.34E-02 | 1.000 | activation of caspase activity |
| GO:0042633 | PROCESS | 27 | 3 | 3.42E-02 | 1.000 | hair cycle |
| GO:0042303 | PROCESS | 27 | 3 | 3.42E-02 | 1.000 | molting cycle |
| GO:0005545 | FUNCTION | 21 | 3 | 3.44E-02 | 1.000 | phosphatidylinositol binding |
| GO:0006461 | PROCESS | 325 | 15 | 3.51E-02 | 1.000 | protein complex assembly |
| GO:0070271 | PROCESS | 325 | 15 | 3.51E-02 | 1.000 | protein complex biogenesis |
| GO:0050999 | PROCESS | 12 | 2 | 3.52E-02 | 1.000 | regulation of nitric-oxide synthase activity |
| GO:0001530 | FUNCTION | 11 | 2 | 3.59E-02 | 1.000 | lipopolysaccharide binding |
| GO:0046503 | PROCESS | 8 | 2 | 3.61E-02 | 1.000 | glycerolipid catabolic process |
| GO:0048806 | PROCESS | 14 | 2 | 3.69E-02 | 1.000 | genitalia development |
| GO:0048070 | PROCESS | 8 | 2 | 3.73E-02 | 1.000 | regulation of pigmentation during development |
| GO:0034361 | CELLULAR | 16 | 2 | 3.78E-02 | 1.000 | very-low-density lipoprotein particle |
| GO:0034385 | CELLULAR | 16 | 2 | 3.78E-02 | 1.000 | triglyceride-rich lipoprotein particle |
| GO:0033363 | PROCESS | 7 | 2 | 3.80E-02 | 1.000 | secretory granule organization |
| GO:0008034 | FUNCTION | 32 | 5 | 3.82E-02 | 1.000 | lipoprotein binding |
| GO:0008305 | CELLULAR | 28 | 4 | 3.87E-02 | 1.000 | integrin complex |
| GO:0016298 | FUNCTION | 95 | 7 | 3.88E-02 | 1.000 | lipase activity |
| GO:0006917 | PROCESS | 227 | 11 | 3.89E-02 | 1.000 | induction of apoptosis |
| GO:0044248 | PROCESS | 951 | 30 | 3.92E-02 | 1.000 | cellular catabolic process |
| GO:0044255 | PROCESS | 598 | 25 | 3.94E-02 | 1.000 | cellular lipid metabolic process |
| GO:0052547 | PROCESS | 72 | 5 | 3.94E-02 | 1.000 | regulation of peptidase activity |
| GO:0042803 | FUNCTION | 261 | 15 | 3.95E-02 | 1.000 | protein homodimerization activity |
| GO:0051219 | FUNCTION | 14 | 2 | 3.99E-02 | 1.000 | phosphoprotein binding |
| GO:0051043 | PROCESS | 12 | 2 | 4.05E-02 | 1.000 | regulation of membrane protein ectodomain proteolysis |
| GO:0005513 | PROCESS | 9 | 3 | 4.05E-02 | 1.000 | detection of calcium ion |

| | | | | | | |
|---|---|---|---|---|---|---|
| GO:0008092 | FUNCTION | 433 | 25 | 4.08E-02 | 1.000 | cytoskeletal protein binding |
| GO:0004715 | FUNCTION | 38 | 4 | 4.25E-02 | 1.000 | non-membrane spanning protein tyrosine kinase activity |
| GO:0046466 | PROCESS | 15 | 2 | 4.26E-02 | 1.000 | membrane lipid catabolic process |
| GO:0030149 | PROCESS | 15 | 2 | 4.26E-02 | 1.000 | sphingolipid catabolic process |
| GO:0045646 | PROCESS | 16 | 2 | 4.35E-02 | 1.000 | regulation of erythrocyte differentiation |
| GO:0006508 | PROCESS | 926 | 32 | 4.36E-02 | 1.000 | proteolysis |
| GO:0042439 | PROCESS | 26 | 3 | 4.42E-02 | 1.000 | ethanolamine and derivative metabolic process |
| GO:0006873 | PROCESS | 274 | 15 | 4.44E-02 | 1.000 | cellular ion homeostasis |
| GO:0032012 | PROCESS | 42 | 4 | 4.45E-02 | 1.000 | regulation of ARF protein signal transduction |
| GO:0032367 | PROCESS | 4 | 2 | 4.47E-02 | 1.000 | intracellular cholesterol transport |
| GO:0032366 | PROCESS | 4 | 2 | 4.47E-02 | 1.000 | intracellular sterol transport |
| GO:0030804 | PROCESS | 9 | 2 | 4.49E-02 | 1.000 | positive regulation of cyclic nucleotide biosynthetic process |
| GO:0030801 | PROCESS | 9 | 2 | 4.49E-02 | 1.000 | positive regulation of cyclic nucleotide metabolic process |
| GO:0030810 | PROCESS | 9 | 2 | 4.49E-02 | 1.000 | positive regulation of nucleotide biosynthetic process |
| GO:0045981 | PROCESS | 9 | 2 | 4.49E-02 | 1.000 | positive regulation of nucleotide metabolic process |
| GO:0050684 | PROCESS | 15 | 2 | 4.50E-02 | 1.000 | regulation of mRNA processing |
| GO:0030163 | PROCESS | 936 | 32 | 4.61E-02 | 1.000 | protein catabolic process |
| GO:0043280 | PROCESS | 51 | 4 | 4.73E-02 | 1.000 | positive regulation of caspase activity |
| GO:0010952 | PROCESS | 51 | 4 | 4.73E-02 | 1.000 | positive regulation of peptidase activity |
| GO:0045833 | PROCESS | 19 | 2 | 4.77E-02 | 1.000 | negative regulation of lipid metabolic process |
| GO:0009595 | PROCESS | 15 | 2 | 4.79E-02 | 1.000 | detection of biotic stimulus |
| GO:0005217 | FUNCTION | 13 | 4 | 4.85E-02 | 1.000 | intracellular ligand-gated ion channel activity |
| GO:0016021 | CELLULAR | 4844 | 168 | 4.85E-02 | 1.000 | integral to membrane |
| GO:0033036 | PROCESS | 946 | 36 | 4.86E-02 | 1.000 | macromolecule localization |
| GO:0006641 | PROCESS | 31 | 3 | 4.86E-02 | 1.000 | triglyceride metabolic process |
| GO:0042471 | PROCESS | 50 | 3 | 4.86E-02 | 1.000 | ear morphogenesis |
| GO:0005272 | FUNCTION | 31 | 4 | 4.88E-02 | 1.000 | sodium channel activity |
| GO:0006954 | PROCESS | 263 | 11 | 4.96E-02 | 1.000 | inflammatory response |
| GO:0008015 | PROCESS | 144 | 9 | 5.01E-02 | 1.000 | blood circulation |
| GO:0003013 | PROCESS | 144 | 9 | 5.01E-02 | 1.000 | circulatory system process |
| GO:0005319 | FUNCTION | 57 | 6 | 5.02E-02 | 1.000 | lipid transporter activity |
| GO:0055082 | PROCESS | 280 | 15 | 5.04E-02 | 1.000 | cellular chemical homeostasis |
| GO:0008235 | FUNCTION | 37 | 4 | 5.12E-02 | 1.000 | metalloexopeptidase activity |
| GO:0015297 | FUNCTION | 59 | 6 | 5.30E-02 | 1.000 | antiporter activity |
| GO:0045862 | PROCESS | 20 | 2 | 5.34E-02 | 1.000 | positive regulation of proteolysis |
| GO:0055080 | PROCESS | 210 | 12 | 5.41E-02 | 1.000 | cation homeostasis |
| GO:0010594 | PROCESS | 49 | 4 | 5.46E-02 | 1.000 | regulation of endothelial cell migration |
| GO:0046546 | PROCESS | 39 | 3 | 5.49E-02 | 1.000 | development of primary male sexual characteristics |
| GO:0015020 | FUNCTION | 28 | 3 | 5.52E-02 | 1.000 | glucuronosyltransferase activity |
| GO:0032365 | PROCESS | 7 | 2 | 5.56E-02 | 1.000 | intracellular lipid transport |
| GO:0007265 | PROCESS | 92 | 6 | 5.61E-02 | 1.000 | Ras protein signal transduction |
| GO:0015276 | FUNCTION | 113 | 11 | 5.61E-02 | 1.000 | ligand-gated ion channel activity |
| GO:0022834 | FUNCTION | 113 | 11 | 5.61E-02 | 1.000 | ligand-gated channel activity |
| GO:0032844 | PROCESS | 80 | 5 | 5.68E-02 | 1.000 | regulation of homeostatic process |
| GO:0055091 | PROCESS | 5 | 2 | 5.71E-02 | 1.000 | phospholipid homeostasis |
| GO:0043167 | FUNCTION | 3926 | 138 | 5.81E-02 | 1.000 | ion binding |
| GO:0006921 | PROCESS | 20 | 2 | 5.89E-02 | 1.000 | cell structure disassembly during apoptosis |
| GO:0003006 | PROCESS | 108 | 6 | 5.93E-02 | 1.000 | reproductive developmental process |
| GO:0043583 | PROCESS | 68 | 5 | 5.93E-02 | 1.000 | ear development |
| GO:0016192 | PROCESS | 484 | 22 | 5.93E-02 | 1.000 | vesicle-mediated transport |
| GO:0005769 | CELLULAR | 64 | 4 | 5.93E-02 | 1.000 | early endosome |
| GO:0051224 | PROCESS | 34 | 3 | 5.98E-02 | 1.000 | negative regulation of protein transport |
| GO:0030139 | CELLULAR | 52 | 5 | 5.99E-02 | 1.000 | endocytic vesicle |
| GO:0045834 | PROCESS | 30 | 3 | 6.03E-02 | 1.000 | positive regulation of lipid metabolic process |
| GO:0007005 | PROCESS | 104 | 5 | 6.11E-02 | 1.000 | mitochondrion organization |
| GO:0005819 | CELLULAR | 112 | 5 | 6.14E-02 | 1.000 | spindle |
| GO:0015291 | FUNCTION | 186 | 12 | 6.20E-02 | 1.000 | secondary active transmembrane transporter activity |
| GO:0051179 | PROCESS | 2739 | 106 | 6.36E-02 | 1.000 | localization |
| GO:0004180 | FUNCTION | 36 | 4 | 6.37E-02 | 1.000 | carboxypeptidase activity |
| GO:0006897 | PROCESS | 181 | 11 | 6.38E-02 | 1.000 | endocytosis |
| GO:0010324 | PROCESS | 181 | 11 | 6.38E-02 | 1.000 | membrane invagination |
| GO:0006915 | PROCESS | 446 | 17 | 6.38E-02 | 1.000 | apoptosis |
| GO:0051341 | PROCESS | 33 | 3 | 6.39E-02 | 1.000 | regulation of oxidoreductase activity |
| GO:0003779 | FUNCTION | 290 | 19 | 6.45E-02 | 1.000 | actin binding |
| GO:0051336 | PROCESS | 289 | 13 | 6.49E-02 | 1.000 | regulation of hydrolase activity |
| GO:0030201 | PROCESS | 13 | 3 | 6.50E-02 | 1.000 | heparan sulfate proteoglycan metabolic process |
| GO:0043279 | PROCESS | 22 | 3 | 6.52E-02 | 1.000 | response to alkaloid |
| GO:0015464 | FUNCTION | 18 | 2 | 6.55E-02 | 1.000 | acetylcholine receptor activity |
| GO:0043624 | PROCESS | 13 | 2 | 6.55E-02 | 1.000 | cellular protein complex disassembly |
| GO:0043241 | PROCESS | 13 | 2 | 6.55E-02 | 1.000 | protein complex disassembly |
| GO:0042312 | PROCESS | 14 | 2 | 6.71E-02 | 1.000 | regulation of vasodilation |
| GO:0006875 | PROCESS | 136 | 9 | 6.88E-02 | 1.000 | cellular metal ion homeostasis |
| GO:0007274 | PROCESS | 15 | 2 | 6.89E-02 | 1.000 | neuromuscular synaptic transmission |
| GO:0051606 | PROCESS | 98 | 7 | 6.94E-02 | 1.000 | detection of stimulus |
| GO:0042166 | FUNCTION | 20 | 2 | 7.00E-02 | 1.000 | acetylcholine binding |
| GO:0009636 | PROCESS | 36 | 3 | 7.01E-02 | 1.000 | response to toxin |
| GO:0012501 | PROCESS | 453 | 17 | 7.01E-02 | 1.000 | programmed cell death |
| GO:0051437 | PROCESS | 63 | 3 | 7.03E-02 | 1.000 | positive regulation of ubiquitin-protein ligase activity during mitotic cell cycle |

| | | | | | | |
|---|---|---|---|---|---|---|
| GO:0032409 | PROCESS | 27 | 3 | 7.12E-02 | 1.000 | regulation of transporter activity |
| GO:0043169 | FUNCTION | 3876 | 135 | 7.13E-02 | 1.000 | cation binding |
| GO:0046928 | PROCESS | 12 | 2 | 7.30E-02 | 1.000 | regulation of neurotransmitter secretion |
| GO:0016251 | FUNCTION | 47 | 3 | 7.46E-02 | 1.000 | general RNA polymerase II transcription factor activity |
| GO:0043535 | PROCESS | 40 | 3 | 7.47E-02 | 1.000 | regulation of blood vessel endothelial cell migration |
| GO:0015300 | FUNCTION | 48 | 5 | 7.50E-02 | 1.000 | solute:solute antiporter activity |
| GO:0043285 | PROCESS | 1074 | 34 | 7.50E-02 | 1.000 | biopolymer catabolic process |
| GO:0051443 | PROCESS | 65 | 3 | 7.51E-02 | 1.000 | positive regulation of ubiquitin-protein ligase activity |
| GO:0005876 | CELLULAR | 24 | 2 | 7.53E-02 | 1.000 | spindle microtubule |
| GO:0048518 | PROCESS | 1531 | 53 | 7.59E-02 | 1.000 | positive regulation of biological process |
| GO:0001816 | PROCESS | 31 | 3 | 7.60E-02 | 1.000 | cytokine production |
| GO:0016755 | FUNCTION | 18 | 2 | 7.65E-02 | 1.000 | transferase activity, transferring amino-acyl groups |
| GO:0018210 | PROCESS | 14 | 2 | 7.66E-02 | 1.000 | peptidyl-threonine modification |
| GO:0009057 | PROCESS | 1130 | 35 | 7.66E-02 | 1.000 | macromolecule catabolic process |
| GO:0007528 | PROCESS | 10 | 2 | 7.67E-02 | 1.000 | neuromuscular junction development |
| GO:0051439 | PROCESS | 66 | 3 | 7.68E-02 | 1.000 | regulation of ubiquitin-protein ligase activity during mitotic cell cycle |
| GO:0030100 | PROCESS | 50 | 4 | 7.71E-02 | 1.000 | regulation of endocytosis |
| GO:0001950 | CELLULAR | 5 | 2 | 7.75E-02 | 1.000 | plasma membrane enriched fraction |
| GO:0048286 | PROCESS | 13 | 2 | 7.76E-02 | 1.000 | lung alveolus development |
| GO:0031331 | PROCESS | 23 | 2 | 7.90E-02 | 1.000 | positive regulation of cellular catabolic process |
| GO:0030276 | FUNCTION | 8 | 2 | 7.93E-02 | 1.000 | clathrin binding |
| GO:0019882 | PROCESS | 54 | 4 | 7.99E-02 | 1.000 | antigen processing and presentation |
| GO:0006639 | PROCESS | 35 | 3 | 8.05E-02 | 1.000 | acylglycerol metabolic process |
| GO:0006638 | PROCESS | 35 | 3 | 8.05E-02 | 1.000 | neutral lipid metabolic process |
| GO:0016411 | FUNCTION | 15 | 2 | 8.06E-02 | 1.000 | acylglycerol O-acyltransferase activity |
| GO:0030003 | PROCESS | 183 | 10 | 8.24E-02 | 1.000 | cellular cation homeostasis |
| GO:0015385 | FUNCTION | 7 | 2 | 8.29E-02 | 1.000 | sodium:hydrogen antiporter activity |
| GO:0005451 | FUNCTION | 7 | 2 | 8.29E-02 | 1.000 | monovalent cation:hydrogen antiporter activity |
| GO:0034374 | PROCESS | 9 | 2 | 8.30E-02 | 1.000 | low-density lipoprotein particle remodeling |
| GO:0015078 | FUNCTION | 77 | 4 | 8.34E-02 | 1.000 | hydrogen ion transmembrane transporter activity |
| GO:0005764 | CELLULAR | 189 | 8 | 8.36E-02 | 1.000 | lysosome |
| GO:0000323 | CELLULAR | 189 | 8 | 8.36E-02 | 1.000 | lytic vacuole |
| GO:0044403 | PROCESS | 35 | 3 | 8.36E-02 | 1.000 | symbiosis, encompassing mutualism through parasitism |
| GO:0001540 | FUNCTION | 12 | 2 | 8.37E-02 | 1.000 | beta-amyloid binding |
| GO:0015629 | CELLULAR | 224 | 14 | 8.37E-02 | 1.000 | actin cytoskeleton |
| GO:0055065 | PROCESS | 143 | 9 | 8.40E-02 | 1.000 | metal ion homeostasis |
| GO:0005741 | CELLULAR | 79 | 4 | 8.59E-02 | 1.000 | mitochondrial outer membrane |
| GO:0031526 | CELLULAR | 15 | 2 | 8.64E-02 | 1.000 | brush border membrane |
| GO:0015077 | FUNCTION | 88 | 5 | 8.68E-02 | 1.000 | monovalent inorganic cation transmembrane transporter activity |
| GO:0006805 | PROCESS | 20 | 2 | 8.76E-02 | 1.000 | xenobiotic metabolic process |
| GO:0006778 | PROCESS | 25 | 2 | 8.82E-02 | 1.000 | porphyrin metabolic process |
| GO:0033013 | PROCESS | 25 | 2 | 8.82E-02 | 1.000 | tetrapyrrole metabolic process |
| GO:0006662 | PROCESS | 37 | 3 | 8.84E-02 | 1.000 | glycerol ether metabolic process |
| GO:0030955 | FUNCTION | 124 | 10 | 8.92E-02 | 1.000 | potassium ion binding |
| GO:0048741 | PROCESS | 41 | 4 | 8.93E-02 | 1.000 | skeletal muscle fiber development |
| GO:0006509 | PROCESS | 13 | 2 | 8.94E-02 | 1.000 | membrane protein ectodomain proteolysis |
| GO:0016363 | CELLULAR | 48 | 3 | 8.99E-02 | 1.000 | nuclear matrix |
| GO:0051351 | PROCESS | 68 | 3 | 9.00E-02 | 1.000 | positive regulation of ligase activity |
| GO:0046872 | FUNCTION | 3842 | 133 | 9.03E-02 | 1.000 | metal ion binding |
| GO:0005231 | FUNCTION | 46 | 5 | 9.10E-02 | 1.000 | excitatory extracellular ligand-gated ion channel activity |
| GO:0030594 | FUNCTION | 92 | 6 | 9.10E-02 | 1.000 | neurotransmitter receptor activity |
| GO:0016891 | FUNCTION | 24 | 2 | 9.12E-02 | 1.000 | endoribonuclease activity, producing 5'-phosphomonoesters |
| GO:0018904 | PROCESS | 38 | 3 | 9.14E-02 | 1.000 | organic ether metabolic process |
| GO:0030169 | FUNCTION | 21 | 4 | 9.15E-02 | 1.000 | low-density lipoprotein binding |
| GO:0007165 | PROCESS | 3016 | 106 | 9.18E-02 | 1.000 | signal transduction |
| GO:0006891 | PROCESS | 18 | 2 | 9.19E-02 | 1.000 | intra-Golgi vesicle-mediated transport |
| GO:0051049 | PROCESS | 317 | 15 | 9.19E-02 | 1.000 | regulation of transport |
| GO:0022890 | FUNCTION | 127 | 7 | 9.21E-02 | 1.000 | inorganic cation transmembrane transporter activity |
| GO:0042165 | FUNCTION | 93 | 6 | 9.21E-02 | 1.000 | neurotransmitter binding |
| GO:0000041 | PROCESS | 63 | 4 | 9.27E-02 | 1.000 | transition metal ion transport |
| GO:0047555 | FUNCTION | 7 | 2 | 9.28E-02 | 1.000 | 3',5'-cyclic-GMP phosphodiesterase activity |
| GO:0009986 | CELLULAR | 248 | 16 | 9.34E-02 | 1.000 | cell surface |
| GO:0043281 | PROCESS | 65 | 4 | 9.36E-02 | 1.000 | regulation of caspase activity |
| GO:0034623 | PROCESS | 20 | 2 | 9.36E-02 | 1.000 | cellular macromolecular complex disassembly |
| GO:0044093 | PROCESS | 481 | 19 | 9.41E-02 | 1.000 | positive regulation of molecular function |
| GO:0015674 | PROCESS | 141 | 12 | 9.43E-02 | 1.000 | di-, tri-valent inorganic cation transport |
| GO:0043473 | PROCESS | 37 | 3 | 9.51E-02 | 1.000 | pigmentation |
| GO:0016567 | PROCESS | 96 | 5 | 9.57E-02 | 1.000 | protein ubiquitination |
| GO:0046578 | PROCESS | 187 | 12 | 9.67E-02 | 1.000 | regulation of Ras protein signal transduction |
| GO:0006996 | PROCESS | 1117 | 35 | 9.70E-02 | 1.000 | organelle organization |
| GO:0051438 | PROCESS | 73 | 3 | 9.76E-02 | 1.000 | regulation of ubiquitin-protein ligase activity |
| GO:0048168 | PROCESS | 16 | 2 | 9.91E-02 | 1.000 | regulation of neuronal synaptic plasticity |
| GO:0006826 | PROCESS | 24 | 2 | 1.00E-01 | 1.000 | iron ion transport |
| GO:0042113 | PROCESS | 53 | 4 | 1.00E-01 | 1.000 | B cell activation |
| GO:0032103 | PROCESS | 48 | 4 | 1.01E-01 | 1.000 | positive regulation of response to external stimulus |
| GO:0006471 | PROCESS | 21 | 2 | 1.01E-01 | 1.000 | protein amino acid ADP-ribosylation |
| GO:0022607 | PROCESS | 639 | 22 | 1.02E-01 | 1.000 | cellular component assembly |
| GO:0004709 | FUNCTION | 17 | 2 | 1.02E-01 | 1.000 | MAP kinase kinase kinase activity |
| GO:0003008 | PROCESS | 1267 | 45 | 1.02E-01 | 1.000 | system process |

| | | | | | | |
|---|---|---|---|---|---|---|
| GO:0034399 | CELLULAR | 52 | 3 | 1.03E-01 | 1.000 | nuclear periphery |
| GO:0019835 | PROCESS | 18 | 2 | 1.03E-01 | 1.000 | cytolysis |
| GO:0015012 | PROCESS | 10 | 2 | 1.04E-01 | 1.000 | heparan sulfate proteoglycan biosynthetic process |
| GO:0030552 | FUNCTION | 12 | 2 | 1.05E-01 | 1.000 | cAMP binding |
| GO:0019218 | PROCESS | 27 | 3 | 1.05E-01 | 1.000 | regulation of steroid metabolic process |
| GO:0060401 | PROCESS | 15 | 3 | 1.06E-01 | 1.000 | cytosolic calcium ion transport |
| GO:0009410 | PROCESS | 23 | 2 | 1.07E-01 | 1.000 | response to xenobiotic stimulus |
| GO:0043235 | CELLULAR | 100 | 7 | 1.08E-01 | 1.000 | receptor complex |
| GO:0005886 | CELLULAR | 3374 | 132 | 1.08E-01 | 1.000 | plasma membrane |
| GO:0006898 | PROCESS | 44 | 4 | 1.09E-01 | 1.000 | receptor-mediated endocytosis |
| GO:0030193 | PROCESS | 25 | 2 | 1.10E-01 | 1.000 | regulation of blood coagulation |
| GO:0032768 | PROCESS | 21 | 2 | 1.10E-01 | 1.000 | regulation of monooxygenase activity |
| GO:0009611 | PROCESS | 399 | 15 | 1.10E-01 | 1.000 | response to wounding |
| GO:0005539 | FUNCTION | 122 | 7 | 1.10E-01 | 1.000 | glycosaminoglycan binding |
| GO:0035091 | FUNCTION | 92 | 6 | 1.11E-01 | 1.000 | phosphoinositide binding |
| GO:0015380 | FUNCTION | 17 | 2 | 1.12E-01 | 1.000 | anion exchanger activity |
| GO:0001653 | FUNCTION | 102 | 6 | 1.12E-01 | 1.000 | peptide receptor activity |
| GO:0003774 | FUNCTION | 135 | 9 | 1.12E-01 | 1.000 | motor activity |
| GO:0010885 | PROCESS | 10 | 2 | 1.13E-01 | 1.000 | regulation of cholesterol storage |
| GO:0051340 | PROCESS | 76 | 3 | 1.14E-01 | 1.000 | regulation of ligase activity |
| GO:0007154 | PROCESS | 3319 | 117 | 1.14E-01 | 1.000 | cell communication |
| GO:0046983 | FUNCTION | 420 | 19 | 1.14E-01 | 1.000 | protein dimerization activity |
| GO:0007204 | PROCESS | 76 | 5 | 1.15E-01 | 1.000 | elevation of cytosolic calcium ion concentration |
| GO:0030425 | CELLULAR | 77 | 6 | 1.16E-01 | 1.000 | dendrite |
| GO:0000049 | FUNCTION | 21 | 2 | 1.17E-01 | 1.000 | tRNA binding |
| GO:0000776 | CELLULAR | 66 | 3 | 1.17E-01 | 1.000 | kinetochore |
| GO:0019838 | FUNCTION | 89 | 6 | 1.17E-01 | 1.000 | growth factor binding |
| GO:0015485 | FUNCTION | 15 | 2 | 1.18E-01 | 1.000 | cholesterol binding |
| GO:0019725 | PROCESS | 356 | 16 | 1.18E-01 | 1.000 | cellular homeostasis |
| GO:0004842 | FUNCTION | 127 | 6 | 1.18E-01 | 1.000 | ubiquitin-protein ligase activity |
| GO:0000151 | CELLULAR | 79 | 4 | 1.18E-01 | 1.000 | ubiquitin ligase complex |
| GO:0004114 | FUNCTION | 24 | 4 | 1.19E-01 | 1.000 | 3',5'-cyclic-nucleotide phosphodiesterase activity |
| GO:0015278 | FUNCTION | 9 | 3 | 1.19E-01 | 1.000 | calcium-release channel activity |
| GO:0004112 | FUNCTION | 25 | 4 | 1.20E-01 | 1.000 | cyclic-nucleotide phosphodiesterase activity |
| GO:0032446 | PROCESS | 107 | 5 | 1.20E-01 | 1.000 | protein modification by small protein conjugation |
| GO:0033619 | PROCESS | 18 | 2 | 1.21E-01 | 1.000 | membrane protein proteolysis |
| GO:0019897 | CELLULAR | 44 | 4 | 1.22E-01 | 1.000 | extrinsic to plasma membrane |
| GO:0007588 | PROCESS | 46 | 4 | 1.22E-01 | 1.000 | excretion |
| GO:0043068 | PROCESS | 305 | 13 | 1.23E-01 | 1.000 | positive regulation of programmed cell death |
| GO:0051588 | PROCESS | 17 | 2 | 1.23E-01 | 1.000 | regulation of neurotransmitter transport |
| GO:0004888 | FUNCTION | 1115 | 41 | 1.23E-01 | 1.000 | transmembrane receptor activity |
| GO:0005887 | CELLULAR | 1099 | 50 | 1.24E-01 | 1.000 | integral to plasma membrane |
| GO:0050818 | PROCESS | 27 | 2 | 1.24E-01 | 1.000 | regulation of coagulation |
| GO:0048863 | PROCESS | 22 | 2 | 1.25E-01 | 1.000 | stem cell differentiation |
| GO:0008415 | FUNCTION | 178 | 7 | 1.25E-01 | 1.000 | acyltransferase activity |
| GO:0016893 | FUNCTION | 30 | 2 | 1.25E-01 | 1.000 | endonuclease activity, active with either ribo- or deoxyribonucleic acids and producing 5'-phosphomonoesters |
| GO:0051480 | PROCESS | 80 | 5 | 1.25E-01 | 1.000 | cytosolic calcium ion homeostasis |
| GO:0006066 | PROCESS | 340 | 13 | 1.25E-01 | 1.000 | alcohol metabolic process |
| GO:0045137 | PROCESS | 80 | 4 | 1.28E-01 | 1.000 | development of primary sexual characteristics |
| GO:0004702 | FUNCTION | 57 | 4 | 1.28E-01 | 1.000 | receptor signaling protein serine/threonine kinase activity |
| GO:0048747 | PROCESS | 47 | 4 | 1.29E-01 | 1.000 | muscle fiber development |
| GO:0010942 | PROCESS | 308 | 13 | 1.29E-01 | 1.000 | positive regulation of cell death |
| GO:0004623 | FUNCTION | 25 | 2 | 1.29E-01 | 1.000 | phospholipase A2 activity |
| GO:0051180 | PROCESS | 21 | 2 | 1.30E-01 | 1.000 | vitamin transport |
| GO:0044459 | CELLULAR | 1964 | 89 | 1.30E-01 | 1.000 | plasma membrane part |
| GO:0044085 | PROCESS | 742 | 23 | 1.31E-01 | 1.000 | cellular component biogenesis |
| GO:0046903 | PROCESS | 216 | 12 | 1.31E-01 | 1.000 | secretion |
| GO:0019935 | PROCESS | 110 | 6 | 1.32E-01 | 1.000 | cyclic-nucleotide-mediated signaling |
| GO:0019439 | PROCESS | 17 | 2 | 1.33E-01 | 1.000 | aromatic compound catabolic process |
| GO:0016747 | FUNCTION | 180 | 7 | 1.35E-01 | 1.000 | transferase activity, transferring acyl groups other than amino-acyl groups |
| GO:0005540 | FUNCTION | 19 | 2 | 1.35E-01 | 1.000 | hyaluronic acid binding |
| GO:0015301 | FUNCTION | 19 | 2 | 1.35E-01 | 1.000 | anion:anion antiporter activity |
| GO:0032879 | PROCESS | 486 | 21 | 1.36E-01 | 1.000 | regulation of localization |
| GO:0016208 | FUNCTION | 16 | 2 | 1.36E-01 | 1.000 | AMP binding |
| GO:0030247 | FUNCTION | 130 | 7 | 1.36E-01 | 1.000 | polysaccharide binding |
| GO:0001871 | FUNCTION | 130 | 7 | 1.36E-01 | 1.000 | pattern binding |
| GO:0016529 | CELLULAR | 32 | 4 | 1.36E-01 | 1.000 | sarcoplasmic reticulum |
| GO:0030183 | PROCESS | 33 | 3 | 1.37E-01 | 1.000 | B cell differentiation |
| GO:0015491 | FUNCTION | 15 | 3 | 1.37E-01 | 1.000 | cation:cation antiporter activity |
| GO:0005792 | CELLULAR | 174 | 7 | 1.39E-01 | 1.000 | microsome |
| GO:0016050 | PROCESS | 48 | 3 | 1.41E-01 | 1.000 | vesicle organization |
| GO:0031625 | FUNCTION | 30 | 2 | 1.41E-01 | 1.000 | ubiquitin protein ligase binding |
| GO:0022403 | PROCESS | 366 | 11 | 1.41E-01 | 1.000 | cell cycle phase |
| GO:0005219 | FUNCTION | 5 | 2 | 1.41E-01 | 1.000 | ryanodine-sensitive calcium-release channel activity |
| GO:0005218 | FUNCTION | 5 | 2 | 1.41E-01 | 1.000 | intracellular ligand-gated calcium channel activity |
| GO:0051345 | PROCESS | 154 | 7 | 1.41E-01 | 1.000 | positive regulation of hydrolase activity |
| GO:0006644 | PROCESS | 164 | 7 | 1.42E-01 | 1.000 | phospholipid metabolic process |

| | | | | | | |
|---|---|---|---|---|---|---|
| GO:0065008 | PROCESS | 1109 | 41 | 1.42E-01 | 1.000 | regulation of biological quality |
| GO:0005902 | CELLULAR | 25 | 3 | 1.42E-01 | 1.000 | microvillus |
| GO:0001664 | FUNCTION | 82 | 3 | 1.43E-01 | 1.000 | G-protein-coupled receptor binding |
| GO:0005905 | CELLULAR | 41 | 3 | 1.44E-01 | 1.000 | coated pit |
| GO:0016746 | FUNCTION | 188 | 7 | 1.45E-01 | 1.000 | transferase activity, transferring acyl groups |
| GO:0034621 | PROCESS | 302 | 8 | 1.45E-01 | 1.000 | cellular macromolecular complex subunit organization |
| GO:0043122 | PROCESS | 97 | 4 | 1.46E-01 | 1.000 | regulation of I-kappaB kinase/NF-kappaB cascade |
| GO:0019787 | FUNCTION | 141 | 6 | 1.46E-01 | 1.000 | small conjugating protein ligase activity |
| GO:0008194 | FUNCTION | 112 | 7 | 1.46E-01 | 1.000 | UDP-glycosyltransferase activity |
| GO:0016528 | CELLULAR | 33 | 4 | 1.46E-01 | 1.000 | sarcoplasm |
| GO:0006941 | PROCESS | 30 | 3 | 1.49E-01 | 1.000 | striated muscle contraction |
| GO:0005777 | CELLULAR | 97 | 4 | 1.49E-01 | 1.000 | peroxisome |
| GO:0042579 | CELLULAR | 97 | 4 | 1.49E-01 | 1.000 | microbody |
| GO:0031410 | CELLULAR | 543 | 21 | 1.50E-01 | 1.000 | cytoplasmic vesicle |
| GO:0000287 | FUNCTION | 420 | 19 | 1.50E-01 | 1.000 | magnesium ion binding |
| GO:0001525 | PROCESS | 109 | 7 | 1.51E-01 | 1.000 | angiogenesis |
| GO:0010926 | PROCESS | 839 | 29 | 1.51E-01 | 1.000 | anatomical structure formation |
| GO:0043623 | PROCESS | 136 | 5 | 1.52E-01 | 1.000 | cellular protein complex assembly |
| GO:0016023 | CELLULAR | 459 | 18 | 1.52E-01 | 1.000 | cytoplasmic membrane-bounded vesicle |
| GO:0009066 | PROCESS | 22 | 2 | 1.52E-01 | 1.000 | aspartate family amino acid metabolic process |
| GO:0005548 | FUNCTION | 28 | 4 | 1.53E-01 | 1.000 | phospholipid transporter activity |
| GO:0044257 | PROCESS | 523 | 15 | 1.54E-01 | 1.000 | cellular protein catabolic process |
| GO:0008584 | PROCESS | 29 | 2 | 1.55E-01 | 1.000 | male gonad development |
| GO:0019637 | PROCESS | 170 | 7 | 1.56E-01 | 1.000 | organophosphate metabolic process |
| GO:0032991 | CELLULAR | 2674 | 73 | 1.58E-01 | 1.000 | macromolecular complex |
| GO:0008080 | FUNCTION | 65 | 3 | 1.58E-01 | 1.000 | N-acetyltransferase activity |
| GO:0008239 | FUNCTION | 10 | 2 | 1.60E-01 | 1.000 | dipeptidyl-peptidase activity |
| GO:0019229 | PROCESS | 18 | 2 | 1.60E-01 | 1.000 | regulation of vasoconstriction |
| GO:0042472 | PROCESS | 41 | 2 | 1.60E-01 | 1.000 | inner ear morphogenesis |
| GO:0043085 | PROCESS | 432 | 16 | 1.61E-01 | 1.000 | positive regulation of catalytic activity |
| GO:0005251 | FUNCTION | 12 | 2 | 1.61E-01 | 1.000 | delayed rectifier potassium channel activity |
| GO:0006790 | PROCESS | 94 | 6 | 1.61E-01 | 1.000 | sulfur metabolic process |
| GO:0031968 | CELLULAR | 91 | 4 | 1.62E-01 | 1.000 | organelle outer membrane |
| GO:0005615 | CELLULAR | 585 | 20 | 1.62E-01 | 1.000 | extracellular space |
| GO:0015277 | FUNCTION | 7 | 2 | 1.63E-01 | 1.000 | kainate selective glutamate receptor activity |
| GO:0031226 | CELLULAR | 1123 | 50 | 1.64E-01 | 1.000 | intrinsic to plasma membrane |
| GO:0042598 | CELLULAR | 179 | 7 | 1.64E-01 | 1.000 | vesicular fraction |
| GO:0007519 | PROCESS | 59 | 4 | 1.65E-01 | 1.000 | skeletal muscle tissue development |
| GO:0060538 | PROCESS | 59 | 4 | 1.65E-01 | 1.000 | skeletal muscle organ development |
| GO:0031988 | CELLULAR | 465 | 18 | 1.65E-01 | 1.000 | membrane-bounded vesicle |
| GO:0001948 | FUNCTION | 20 | 3 | 1.66E-01 | 1.000 | glycoprotein binding |
| GO:0031982 | CELLULAR | 552 | 21 | 1.67E-01 | 1.000 | vesicle |
| GO:0007157 | PROCESS | 18 | 2 | 1.68E-01 | 1.000 | heterophilic cell adhesion |
| GO:0005773 | CELLULAR | 222 | 8 | 1.70E-01 | 1.000 | vacuole |
| GO:0043065 | PROCESS | 303 | 12 | 1.70E-01 | 1.000 | positive regulation of apoptosis |
| GO:0008610 | PROCESS | 290 | 11 | 1.72E-01 | 1.000 | lipid biosynthetic process |
| GO:0005024 | FUNCTION | 16 | 2 | 1.72E-01 | 1.000 | transforming growth factor beta receptor activity |
| GO:0004675 | FUNCTION | 16 | 2 | 1.72E-01 | 1.000 | transmembrane receptor protein serine/threonine kinase activity |
| GO:0008188 | FUNCTION | 37 | 3 | 1.72E-01 | 1.000 | neuropeptide receptor activity |
| GO:0046890 | PROCESS | 30 | 2 | 1.72E-01 | 1.000 | regulation of lipid biosynthetic process |
| GO:0004857 | FUNCTION | 248 | 9 | 1.74E-01 | 1.000 | enzyme inhibitor activity |
| GO:0044419 | PROCESS | 265 | 9 | 1.75E-01 | 1.000 | interspecies interaction between organisms |
| GO:0016043 | PROCESS | 1996 | 67 | 1.77E-01 | 1.000 | cellular component organization |
| GO:0004115 | FUNCTION | 7 | 2 | 1.77E-01 | 1.000 | 3',5'-cyclic-AMP phosphodiesterase activity |
| GO:0042923 | FUNCTION | 38 | 3 | 1.78E-01 | 1.000 | neuropeptide binding |
| GO:0032318 | PROCESS | 94 | 5 | 1.80E-01 | 1.000 | regulation of Ras GTPase activity |
| GO:0031091 | CELLULAR | 53 | 3 | 1.80E-01 | 1.000 | platelet alpha granule |
| GO:0019867 | CELLULAR | 94 | 4 | 1.81E-01 | 1.000 | outer membrane |
| GO:0043395 | FUNCTION | 5 | 2 | 1.82E-01 | 1.000 | heparan sulfate proteoglycan binding |
| GO:0015031 | PROCESS | 679 | 22 | 1.82E-01 | 1.000 | protein transport |
| GO:0010883 | PROCESS | 19 | 2 | 1.83E-01 | 1.000 | regulation of lipid storage |
| GO:0008643 | PROCESS | 57 | 3 | 1.83E-01 | 1.000 | carbohydrate transport |
| GO:0009306 | PROCESS | 20 | 2 | 1.86E-01 | 1.000 | protein secretion |
| GO:0006909 | PROCESS | 39 | 3 | 1.86E-01 | 1.000 | phagocytosis |
| GO:0007631 | PROCESS | 44 | 3 | 1.87E-01 | 1.000 | feeding behavior |
| GO:0051099 | PROCESS | 56 | 3 | 1.87E-01 | 1.000 | positive regulation of binding |
| GO:0032101 | PROCESS | 114 | 6 | 1.88E-01 | 1.000 | regulation of response to external stimulus |
| GO:0055037 | CELLULAR | 18 | 2 | 1.88E-01 | 1.000 | recycling endosome |
| GO:0045884 | PROCESS | 19 | 2 | 1.90E-01 | 1.000 | regulation of survival gene product expression |
| GO:0042102 | PROCESS | 30 | 2 | 1.90E-01 | 1.000 | positive regulation of T cell proliferation |
| GO:0070011 | FUNCTION | 514 | 19 | 1.90E-01 | 1.000 | peptidase activity, acting on L-amino acid peptides |
| GO:0032934 | FUNCTION | 22 | 2 | 1.90E-01 | 1.000 | sterol binding |
| GO:0008284 | PROCESS | 306 | 11 | 1.91E-01 | 1.000 | positive regulation of cell proliferation |
| GO:0080134 | PROCESS | 195 | 7 | 1.91E-01 | 1.000 | regulation of response to stress |
| GO:0022804 | FUNCTION | 319 | 17 | 1.91E-01 | 1.000 | active transmembrane transporter activity |
| GO:0005793 | CELLULAR | 39 | 2 | 1.91E-01 | 1.000 | ER-Golgi intermediate compartment |
| GO:0019538 | PROCESS | 2511 | 74 | 1.91E-01 | 1.000 | protein metabolic process |
| GO:0010467 | PROCESS | 1099 | 26 | 1.92E-01 | 1.000 | gene expression |
| GO:0004521 | FUNCTION | 43 | 2 | 1.92E-01 | 1.000 | endoribonuclease activity |

| | | | | | | |
|---|---|---|---|---|---|---|
| GO:0050808 | PROCESS | 36 | 3 | 1.92E-01 | 1.000 | synapse organization |
| GO:0007586 | PROCESS | 79 | 4 | 1.93E-01 | 1.000 | digestion |
| GO:0016573 | PROCESS | 45 | 2 | 1.93E-01 | 1.000 | histone acetylation |
| GO:0060627 | PROCESS | 73 | 4 | 1.95E-01 | 1.000 | regulation of vesicle-mediated transport |
| GO:0006816 | PROCESS | 112 | 10 | 1.95E-01 | 1.000 | calcium ion transport |
| GO:0043565 | FUNCTION | 524 | 17 | 1.95E-01 | 1.000 | sequence-specific DNA binding |
| GO:0005740 | CELLULAR | 347 | 9 | 1.95E-01 | 1.000 | mitochondrial envelope |
| GO:0070838 | PROCESS | 113 | 10 | 1.96E-01 | 1.000 | divalent metal ion transport |
| GO:0045184 | PROCESS | 685 | 22 | 1.97E-01 | 1.000 | establishment of protein localization |
| GO:0044057 | PROCESS | 190 | 11 | 1.99E-01 | 1.000 | regulation of system process |
| GO:0008289 | FUNCTION | 455 | 20 | 1.99E-01 | 1.000 | lipid binding |
| GO:0004402 | FUNCTION | 36 | 2 | 2.00E-01 | 1.000 | histone acetyltransferase activity |
| GO:0004468 | FUNCTION | 36 | 2 | 2.00E-01 | 1.000 | lysine N-acetyltransferase activity |
| GO:0007187 | PROCESS | 100 | 5 | 2.01E-01 | 1.000 | G-protein signaling, coupled to cyclic nucleotide second messenger |
| GO:0016757 | FUNCTION | 250 | 11 | 2.01E-01 | 1.000 | transferase activity, transferring glycosyl groups |
| GO:0065009 | PROCESS | 814 | 27 | 2.01E-01 | 1.000 | regulation of molecular function |
| GO:0050877 | PROCESS | 1010 | 34 | 2.01E-01 | 1.000 | neurological system process |
| GO:0051056 | PROCESS | 228 | 13 | 2.02E-01 | 1.000 | regulation of small GTPase mediated signal transduction |
| GO:0005179 | FUNCTION | 102 | 3 | 2.02E-01 | 1.000 | hormone activity |
| GO:0018130 | PROCESS | 41 | 2 | 2.03E-01 | 1.000 | heterocycle biosynthetic process |
| GO:0051701 | PROCESS | 27 | 2 | 2.03E-01 | 1.000 | interaction with host |
| GO:0015299 | FUNCTION | 19 | 2 | 2.04E-01 | 1.000 | solute:hydrogen antiporter activity |
| GO:0070647 | PROCESS | 132 | 5 | 2.04E-01 | 1.000 | protein modification by small protein conjugation or removal |
| GO:0006473 | PROCESS | 48 | 2 | 2.07E-01 | 1.000 | protein amino acid acetylation |
| GO:0051246 | PROCESS | 467 | 15 | 2.08E-01 | 1.000 | regulation of protein metabolic process |
| GO:0016197 | PROCESS | 47 | 3 | 2.08E-01 | 1.000 | endosome transport |
| GO:0016570 | PROCESS | 104 | 4 | 2.09E-01 | 1.000 | histone modification |
| GO:0006800 | PROCESS | 51 | 3 | 2.10E-01 | 1.000 | oxygen and reactive oxygen species metabolic process |
| GO:0035258 | FUNCTION | 36 | 2 | 2.10E-01 | 1.000 | steroid hormone receptor binding |
| GO:0008233 | FUNCTION | 530 | 19 | 2.10E-01 | 1.000 | peptidase activity |
| GO:0016758 | FUNCTION | 170 | 8 | 2.10E-01 | 1.000 | transferase activity, transferring hexosyl groups |
| GO:0031333 | PROCESS | 29 | 2 | 2.11E-01 | 1.000 | negative regulation of protein complex assembly |
| GO:0031267 | FUNCTION | 72 | 5 | 2.11E-01 | 1.000 | small GTPase binding |
| GO:0044272 | PROCESS | 45 | 3 | 2.11E-01 | 1.000 | sulfur compound biosynthetic process |
| GO:0008219 | PROCESS | 550 | 19 | 2.12E-01 | 1.000 | cell death |
| GO:0007229 | PROCESS | 55 | 4 | 2.12E-01 | 1.000 | integrin-mediated signaling pathway |
| GO:0008374 | FUNCTION | 36 | 2 | 2.12E-01 | 1.000 | O-acyltransferase activity |
| GO:0004896 | FUNCTION | 48 | 3 | 2.15E-01 | 1.000 | cytokine receptor activity |
| GO:0016569 | PROCESS | 106 | 4 | 2.16E-01 | 1.000 | covalent chromatin modification |
| GO:0004519 | FUNCTION | 90 | 3 | 2.17E-01 | 1.000 | endonuclease activity |
| GO:0030334 | PROCESS | 159 | 8 | 2.17E-01 | 1.000 | regulation of cell migration |
| GO:0001726 | CELLULAR | 56 | 3 | 2.17E-01 | 1.000 | ruffle |
| GO:0015298 | FUNCTION | 26 | 3 | 2.18E-01 | 1.000 | solute:cation antiporter activity |
| GO:0015293 | FUNCTION | 121 | 7 | 2.18E-01 | 1.000 | symporter activity |
| GO:0004653 | FUNCTION | 19 | 3 | 2.18E-01 | 1.000 | polypeptide N-acetylgalactosaminyltransferase activity |
| GO:0016265 | PROCESS | 554 | 19 | 2.19E-01 | 1.000 | death |
| GO:0042562 | FUNCTION | 29 | 3 | 2.20E-01 | 1.000 | hormone binding |
| GO:0000279 | PROCESS | 299 | 8 | 2.21E-01 | 1.000 | M phase |
| GO:0016486 | PROCESS | 13 | 2 | 2.21E-01 | 1.000 | peptide hormone processing |
| GO:0030516 | PROCESS | 12 | 2 | 2.21E-01 | 1.000 | regulation of axon extension |
| GO:0030246 | FUNCTION | 303 | 12 | 2.25E-01 | 1.000 | carbohydrate binding |
| GO:0050927 | PROCESS | 12 | 2 | 2.25E-01 | 1.000 | positive regulation of positive chemotaxis |
| GO:0050926 | PROCESS | 12 | 2 | 2.25E-01 | 1.000 | regulation of positive chemotaxis |
| GO:0008528 | FUNCTION | 97 | 5 | 2.26E-01 | 1.000 | peptide receptor activity, G-protein coupled |
| GO:0030175 | CELLULAR | 24 | 2 | 2.27E-01 | 1.000 | filopodium |
| GO:0003700 | FUNCTION | 880 | 26 | 2.28E-01 | 1.000 | transcription factor activity |
| GO:0048514 | PROCESS | 152 | 8 | 2.28E-01 | 1.000 | blood vessel morphogenesis |
| GO:0008047 | FUNCTION | 305 | 15 | 2.29E-01 | 1.000 | enzyme activator activity |
| GO:0016407 | FUNCTION | 78 | 3 | 2.29E-01 | 1.000 | acetyltransferase activity |
| GO:0030141 | CELLULAR | 139 | 6 | 2.30E-01 | 1.000 | secretory granule |
| GO:0051603 | PROCESS | 521 | 14 | 2.30E-01 | 1.000 | proteolysis involved in cellular protein catabolic process |
| GO:0007186 | PROCESS | 950 | 25 | 2.30E-01 | 1.000 | G-protein coupled receptor protein signaling pathway |
| GO:0016814 | FUNCTION | 30 | 2 | 2.30E-01 | 1.000 | hydrolase activity, acting on carbon-nitrogen (but not peptide) bonds, in cyclic amidines |
| GO:0008237 | FUNCTION | 172 | 9 | 2.34E-01 | 1.000 | metallopeptidase activity |
| GO:0008629 | PROCESS | 41 | 2 | 2.34E-01 | 1.000 | induction of apoptosis by intracellular signals |
| GO:0005057 | FUNCTION | 153 | 8 | 2.35E-01 | 1.000 | receptor signaling protein activity |
| GO:0030234 | FUNCTION | 759 | 31 | 2.35E-01 | 1.000 | enzyme regulator activity |
| GO:0050671 | PROCESS | 37 | 2 | 2.36E-01 | 1.000 | positive regulation of lymphocyte proliferation |
| GO:0008236 | FUNCTION | 174 | 7 | 2.36E-01 | 1.000 | serine-type peptidase activity |
| GO:0043087 | PROCESS | 106 | 5 | 2.38E-01 | 1.000 | regulation of GTPase activity |
| GO:0008081 | FUNCTION | 81 | 6 | 2.39E-01 | 1.000 | phosphoric diester hydrolase activity |
| GO:0043025 | CELLULAR | 73 | 4 | 2.39E-01 | 1.000 | cell soma |
| GO:0030228 | FUNCTION | 14 | 2 | 2.40E-01 | 1.000 | lipoprotein receptor activity |
| GO:0005230 | FUNCTION | 67 | 5 | 2.41E-01 | 1.000 | extracellular ligand-gated ion channel activity |
| GO:0000777 | CELLULAR | 48 | 2 | 2.41E-01 | 1.000 | condensed chromosome kinetochore |
| GO:0043394 | FUNCTION | 7 | 2 | 2.42E-01 | 1.000 | proteoglycan binding |
| GO:0042277 | FUNCTION | 169 | 8 | 2.43E-01 | 1.000 | peptide binding |
| GO:0017171 | FUNCTION | 176 | 7 | 2.43E-01 | 1.000 | serine hydrolase activity |

| | | | | | | |
|---|---|---|---|---|---|---|
| GO:0005626 | CELLULAR | 628 | 26 | 2.45E-01 | 1.000 | insoluble fraction |
| GO:0008406 | PROCESS | 68 | 3 | 2.45E-01 | 1.000 | gonad development |
| GO:0032946 | PROCESS | 38 | 2 | 2.46E-01 | 1.000 | positive regulation of mononuclear cell proliferation |
| GO:0070665 | PROCESS | 38 | 2 | 2.46E-01 | 1.000 | positive regulation of leukocyte proliferation |
| GO:0046486 | PROCESS | 127 | 5 | 2.46E-01 | 1.000 | glycerolipid metabolic process |
| GO:0009190 | PROCESS | 22 | 2 | 2.47E-01 | 1.000 | cyclic nucleotide biosynthetic process |
| GO:0016787 | FUNCTION | 2094 | 66 | 2.47E-01 | 1.000 | hydrolase activity |
| GO:0008624 | PROCESS | 59 | 3 | 2.47E-01 | 1.000 | induction of apoptosis by extracellular signals |
| GO:0060402 | PROCESS | 14 | 2 | 2.47E-01 | 1.000 | calcium ion transport into cytosol |
| GO:0051128 | PROCESS | 362 | 14 | 2.48E-01 | 1.000 | regulation of cellular component organization |
| GO:0030335 | PROCESS | 70 | 4 | 2.48E-01 | 1.000 | positive regulation of cell migration |
| GO:0005938 | CELLULAR | 104 | 5 | 2.49E-01 | 1.000 | cell cortex |
| GO:0003725 | FUNCTION | 30 | 2 | 2.50E-01 | 1.000 | double-stranded RNA binding |
| GO:0031329 | PROCESS | 39 | 2 | 2.50E-01 | 1.000 | regulation of cellular catabolic process |
| GO:0016410 | FUNCTION | 77 | 3 | 2.50E-01 | 1.000 | N-acyltransferase activity |
| GO:0016765 | FUNCTION | 47 | 2 | 2.50E-01 | 1.000 | transferase activity, transferring alkyl or aryl (other than methyl) groups |
| GO:0051651 | PROCESS | 41 | 2 | 2.51E-01 | 1.000 | maintenance of location in cell |
| GO:0044450 | CELLULAR | 44 | 2 | 2.52E-01 | 1.000 | microtubule organizing center part |
| GO:0007605 | PROCESS | 80 | 6 | 2.53E-01 | 1.000 | sensory perception of sound |
| GO:0008277 | PROCESS | 48 | 3 | 2.53E-01 | 1.000 | regulation of G-protein coupled receptor protein signaling pathway |
| GO:0001701 | PROCESS | 115 | 5 | 2.54E-01 | 1.000 | in utero embryonic development |
| GO:0006952 | PROCESS | 514 | 15 | 2.55E-01 | 1.000 | defense response |
| GO:0009897 | CELLULAR | 114 | 8 | 2.55E-01 | 1.000 | external side of plasma membrane |
| GO:0051705 | PROCESS | 17 | 2 | 2.56E-01 | 1.000 | behavioral interaction between organisms |
| GO:0044448 | CELLULAR | 60 | 3 | 2.56E-01 | 1.000 | cell cortex part |
| GO:0019898 | CELLULAR | 461 | 21 | 2.57E-01 | 1.000 | extrinsic to membrane |
| GO:0045471 | PROCESS | 23 | 2 | 2.58E-01 | 1.000 | response to ethanol |
| GO:0006887 | PROCESS | 86 | 5 | 2.60E-01 | 1.000 | exocytosis |
| GO:0000775 | CELLULAR | 106 | 3 | 2.61E-01 | 1.000 | chromosome, centromeric region |
| GO:0030667 | CELLULAR | 20 | 2 | 2.62E-01 | 1.000 | secretory granule membrane |
| GO:0022402 | PROCESS | 506 | 13 | 2.64E-01 | 1.000 | cell cycle process |
| GO:0016788 | FUNCTION | 638 | 22 | 2.64E-01 | 1.000 | hydrolase activity, acting on ester bonds |
| GO:0042129 | PROCESS | 43 | 2 | 2.64E-01 | 1.000 | regulation of T cell proliferation |
| GO:0019955 | FUNCTION | 97 | 4 | 2.64E-01 | 1.000 | cytokine binding |
| GO:0031966 | CELLULAR | 326 | 8 | 2.65E-01 | 1.000 | mitochondrial membrane |
| GO:0015103 | FUNCTION | 30 | 2 | 2.65E-01 | 1.000 | inorganic anion transmembrane transporter activity |
| GO:0034622 | PROCESS | 269 | 6 | 2.66E-01 | 1.000 | cellular macromolecular complex assembly |
| GO:0006164 | PROCESS | 130 | 6 | 2.66E-01 | 1.000 | purine nucleotide biosynthetic process |
| GO:0008270 | FUNCTION | 2124 | 60 | 2.67E-01 | 1.000 | zinc ion binding |
| GO:0006029 | PROCESS | 34 | 3 | 2.68E-01 | 1.000 | proteoglycan metabolic process |
| GO:0080135 | PROCESS | 80 | 3 | 2.68E-01 | 1.000 | regulation of cellular response to stress |
| GO:0008016 | PROCESS | 60 | 4 | 2.69E-01 | 1.000 | regulation of heart contraction |
| GO:0007268 | PROCESS | 235 | 14 | 2.69E-01 | 1.000 | synaptic transmission |
| GO:0043543 | PROCESS | 54 | 2 | 2.70E-01 | 1.000 | protein amino acid acylation |
| GO:0009895 | PROCESS | 35 | 2 | 2.71E-01 | 1.000 | negative regulation of catabolic process |
| GO:0010743 | PROCESS | 25 | 2 | 2.71E-01 | 1.000 | regulation of macrophage derived foam cell differentiation |
| GO:0017016 | FUNCTION | 64 | 4 | 2.72E-01 | 1.000 | Ras GTPase binding |
| GO:0030414 | FUNCTION | 141 | 5 | 2.74E-01 | 1.000 | peptidase inhibitor activity |
| GO:0016849 | FUNCTION | 20 | 2 | 2.76E-01 | 1.000 | phosphorus-oxygen lyase activity |
| GO:0007067 | PROCESS | 201 | 5 | 2.77E-01 | 1.000 | mitosis |
| GO:0000280 | PROCESS | 201 | 5 | 2.77E-01 | 1.000 | nuclear division |
| GO:0034962 | PROCESS | 635 | 16 | 2.77E-01 | 1.000 | cellular biopolymer catabolic process |
| GO:0050896 | PROCESS | 2795 | 74 | 2.77E-01 | 1.000 | response to stimulus |
| GO:0001709 | PROCESS | 29 | 2 | 2.78E-01 | 1.000 | cell fate determination |
| GO:0001894 | PROCESS | 33 | 2 | 2.78E-01 | 1.000 | tissue homeostasis |
| GO:0044433 | CELLULAR | 154 | 7 | 2.79E-01 | 1.000 | cytoplasmic vesicle part |
| GO:0048661 | PROCESS | 16 | 2 | 2.80E-01 | 1.000 | positive regulation of smooth muscle cell proliferation |
| GO:0051272 | PROCESS | 75 | 4 | 2.80E-01 | 1.000 | positive regulation of cell motion |
| GO:0043067 | PROCESS | 616 | 20 | 2.81E-01 | 1.000 | regulation of programmed cell death |
| GO:0043123 | PROCESS | 89 | 3 | 2.81E-01 | 1.000 | positive regulation of I-kappaB kinase/NF-kappaB cascade |
| GO:0017111 | FUNCTION | 657 | 23 | 2.82E-01 | 1.000 | nucleoside-triphosphatase activity |
| GO:0000779 | CELLULAR | 53 | 2 | 2.82E-01 | 1.000 | condensed chromosome, centromeric region |
| GO:0044265 | PROCESS | 690 | 17 | 2.82E-01 | 1.000 | cellular macromolecule catabolic process |
| GO:0017137 | FUNCTION | 20 | 2 | 2.82E-01 | 1.000 | Rab GTPase binding |
| GO:0006163 | PROCESS | 155 | 7 | 2.82E-01 | 1.000 | purine nucleotide metabolic process |
| GO:0006885 | PROCESS | 26 | 2 | 2.82E-01 | 1.000 | regulation of pH |
| GO:0048871 | PROCESS | 34 | 2 | 2.84E-01 | 1.000 | multicellular organismal homeostasis |
| GO:0046914 | FUNCTION | 2560 | 73 | 2.84E-01 | 1.000 | transition metal ion binding |
| GO:0010941 | PROCESS | 618 | 20 | 2.84E-01 | 1.000 | regulation of cell death |
| GO:0060193 | PROCESS | 61 | 3 | 2.85E-01 | 1.000 | positive regulation of lipase activity |
| GO:0006979 | PROCESS | 106 | 4 | 2.86E-01 | 1.000 | response to oxidative stress |
| GO:0005096 | FUNCTION | 204 | 11 | 2.86E-01 | 1.000 | GTPase activator activity |
| GO:0000087 | PROCESS | 203 | 5 | 2.87E-01 | 1.000 | M phase of mitotic cell cycle |
| GO:0006916 | PROCESS | 172 | 6 | 2.87E-01 | 1.000 | anti-apoptosis |
| GO:0007127 | PROCESS | 36 | 2 | 2.89E-01 | 1.000 | meiosis I |
| GO:0051301 | PROCESS | 266 | 7 | 2.89E-01 | 1.000 | cell division |
| GO:0009892 | PROCESS | 618 | 19 | 2.91E-01 | 1.000 | negative regulation of metabolic process |
| GO:0030802 | PROCESS | 88 | 5 | 2.92E-01 | 1.000 | regulation of cyclic nucleotide biosynthetic process |
| GO:0030808 | PROCESS | 88 | 5 | 2.92E-01 | 1.000 | regulation of nucleotide biosynthetic process |

| | | | | | | |
|---|---|---|---|---|---|---|
| GO:0045177 | CELLULAR | 107 | 6 | 2.92E-01 | 1.000 | apical part of cell |
| GO:0005903 | CELLULAR | 31 | 2 | 2.94E-01 | 1.000 | brush border |
| GO:0051248 | PROCESS | 132 | 5 | 2.94E-01 | 1.000 | negative regulation of protein metabolic process |
| GO:0043234 | CELLULAR | 2177 | 63 | 2.95E-01 | 1.000 | protein complex |
| GO:0008217 | PROCESS | 69 | 3 | 2.95E-01 | 1.000 | regulation of blood pressure |
| GO:0016836 | FUNCTION | 40 | 2 | 2.95E-01 | 1.000 | hydro-lyase activity |
| GO:0051020 | FUNCTION | 78 | 5 | 2.96E-01 | 1.000 | GTPase binding |
| GO:0048522 | PROCESS | 1406 | 44 | 2.96E-01 | 1.000 | positive regulation of cellular process |
| GO:0048839 | PROCESS | 55 | 3 | 2.96E-01 | 1.000 | inner ear development |
| GO:0003702 | FUNCTION | 216 | 7 | 2.97E-01 | 1.000 | RNA polymerase II transcription factor activity |
| GO:0051704 | PROCESS | 553 | 15 | 2.97E-01 | 1.000 | multi-organism process |
| GO:0048608 | PROCESS | 77 | 3 | 2.97E-01 | 1.000 | reproductive structure development |
| GO:0001568 | PROCESS | 171 | 8 | 2.98E-01 | 1.000 | blood vessel development |
| GO:0009975 | FUNCTION | 21 | 2 | 2.99E-01 | 1.000 | cyclase activity |
| GO:0007517 | PROCESS | 176 | 8 | 2.99E-01 | 1.000 | muscle organ development |
| GO:0005275 | FUNCTION | 66 | 4 | 2.99E-01 | 1.000 | amine transmembrane transporter activity |
| GO:0048285 | PROCESS | 208 | 5 | 3.00E-01 | 1.000 | organelle fission |
| GO:0000398 | PROCESS | 140 | 3 | 3.04E-01 | 1.000 | nuclear mRNA splicing, via spliceosome |
| GO:0000377 | PROCESS | 140 | 3 | 3.04E-01 | 1.000 | RNA splicing, via transesterification reactions with bulged adenosine as nucleophile |
| GO:0000375 | PROCESS | 140 | 3 | 3.04E-01 | 1.000 | RNA splicing, via transesterification reactions |
| GO:0030145 | FUNCTION | 146 | 7 | 3.04E-01 | 1.000 | manganese ion binding |
| GO:0008064 | PROCESS | 49 | 2 | 3.06E-01 | 1.000 | regulation of actin polymerization or depolymerization |
| GO:0006518 | PROCESS | 46 | 2 | 3.06E-01 | 1.000 | peptide metabolic process |
| GO:0040012 | PROCESS | 174 | 8 | 3.07E-01 | 1.000 | regulation of locomotion |
| GO:0000278 | PROCESS | 328 | 8 | 3.08E-01 | 1.000 | mitotic cell cycle |
| GO:0051252 | PROCESS | 1601 | 40 | 3.10E-01 | 1.000 | regulation of RNA metabolic process |
| GO:0003704 | FUNCTION | 34 | 2 | 3.11E-01 | 1.000 | specific RNA polymerase II transcription factor activity |
| GO:0016044 | PROCESS | 313 | 12 | 3.11E-01 | 1.000 | membrane organization |
| GO:0031093 | CELLULAR | 38 | 2 | 3.13E-01 | 1.000 | platelet alpha granule lumen |
| GO:0001944 | PROCESS | 176 | 8 | 3.15E-01 | 1.000 | vasculature development |
| GO:0007126 | PROCESS | 82 | 3 | 3.16E-01 | 1.000 | meiosis |
| GO:0051327 | PROCESS | 82 | 3 | 3.16E-01 | 1.000 | M phase of meiotic cell cycle |
| GO:0050954 | PROCESS | 84 | 6 | 3.17E-01 | 1.000 | sensory perception of mechanical stimulus |
| GO:0043542 | PROCESS | 16 | 2 | 3.17E-01 | 1.000 | endothelial cell migration |
| GO:0030799 | PROCESS | 91 | 5 | 3.17E-01 | 1.000 | regulation of cyclic nucleotide metabolic process |
| GO:0051321 | PROCESS | 84 | 3 | 3.19E-01 | 1.000 | meiotic cell cycle |
| GO:0006950 | PROCESS | 1334 | 36 | 3.20E-01 | 1.000 | response to stress |
| GO:0016881 | FUNCTION | 176 | 6 | 3.20E-01 | 1.000 | acid-amino acid ligase activity |
| GO:0004540 | FUNCTION | 62 | 2 | 3.20E-01 | 1.000 | ribonuclease activity |
| GO:0005624 | CELLULAR | 613 | 24 | 3.21E-01 | 1.000 | membrane fraction |
| GO:0050790 | PROCESS | 712 | 22 | 3.21E-01 | 1.000 | regulation of catalytic activity |
| GO:0000302 | PROCESS | 42 | 2 | 3.22E-01 | 1.000 | response to reactive oxygen species |
| GO:0015085 | FUNCTION | 12 | 2 | 3.22E-01 | 1.000 | calcium ion transmembrane transporter activity |
| GO:0051329 | PROCESS | 81 | 3 | 3.22E-01 | 1.000 | interphase of mitotic cell cycle |
| GO:0060191 | PROCESS | 70 | 3 | 3.23E-01 | 1.000 | regulation of lipase activity |
| GO:0016209 | FUNCTION | 44 | 2 | 3.24E-01 | 1.000 | antioxidant activity |
| GO:0030666 | CELLULAR | 31 | 2 | 3.25E-01 | 1.000 | endocytic vesicle membrane |
| GO:0035023 | PROCESS | 88 | 6 | 3.25E-01 | 1.000 | regulation of Rho protein signal transduction |
| GO:0019941 | PROCESS | 517 | 13 | 3.26E-01 | 1.000 | modification-dependent protein catabolic process |
| GO:0043632 | PROCESS | 517 | 13 | 3.26E-01 | 1.000 | modification-dependent macromolecule catabolic process |
| GO:0006352 | PROCESS | 73 | 2 | 3.27E-01 | 1.000 | transcription initiation |
| GO:0006140 | PROCESS | 94 | 5 | 3.29E-01 | 1.000 | regulation of nucleotide metabolic process |
| GO:0000082 | PROCESS | 46 | 2 | 3.29E-01 | 1.000 | G1/S transition of mitotic cell cycle |
| GO:0050670 | PROCESS | 52 | 2 | 3.30E-01 | 1.000 | regulation of lymphocyte proliferation |
| GO:0005581 | CELLULAR | 33 | 3 | 3.31E-01 | 1.000 | collagen |
| GO:0006631 | PROCESS | 179 | 6 | 3.32E-01 | 1.000 | fatty acid metabolic process |
| GO:0030863 | CELLULAR | 37 | 2 | 3.33E-01 | 1.000 | cortical cytoskeleton |
| GO:0006281 | PROCESS | 257 | 7 | 3.36E-01 | 1.000 | DNA repair |
| GO:0051270 | PROCESS | 175 | 8 | 3.37E-01 | 1.000 | regulation of cell motion |
| GO:0043170 | PROCESS | 3776 | 97 | 3.38E-01 | 1.000 | macromolecule metabolic process |
| GO:0032268 | PROCESS | 408 | 12 | 3.38E-01 | 1.000 | regulation of cellular protein metabolic process |
| GO:0008104 | PROCESS | 773 | 23 | 3.38E-01 | 1.000 | protein localization |
| GO:0046328 | PROCESS | 53 | 2 | 3.38E-01 | 1.000 | regulation of JNK cascade |
| GO:0043283 | PROCESS | 3670 | 95 | 3.39E-01 | 1.000 | biopolymer metabolic process |
| GO:0000267 | CELLULAR | 796 | 29 | 3.39E-01 | 1.000 | cell fraction |
| GO:0016810 | FUNCTION | 107 | 4 | 3.39E-01 | 1.000 | hydrolase activity, acting on carbon-nitrogen (but not peptide) bonds |
| GO:0042060 | PROCESS | 131 | 5 | 3.40E-01 | 1.000 | wound healing |
| GO:0032944 | PROCESS | 53 | 2 | 3.41E-01 | 1.000 | regulation of mononuclear cell proliferation |
| GO:0070663 | PROCESS | 53 | 2 | 3.41E-01 | 1.000 | regulation of leukocyte proliferation |
| GO:0060205 | CELLULAR | 40 | 2 | 3.42E-01 | 1.000 | cytoplasmic membrane-bounded vesicle lumen |
| GO:0031983 | CELLULAR | 40 | 2 | 3.42E-01 | 1.000 | vesicle lumen |
| GO:0030166 | PROCESS | 22 | 2 | 3.43E-01 | 1.000 | proteoglycan biosynthetic process |
| GO:0051235 | PROCESS | 53 | 2 | 3.44E-01 | 1.000 | maintenance of location |
| GO:0060255 | PROCESS | 2929 | 72 | 3.44E-01 | 1.000 | regulation of macromolecule metabolic process |
| GO:0032561 | FUNCTION | 343 | 10 | 3.44E-01 | 1.000 | guanyl ribonucleotide binding |
| GO:0019001 | FUNCTION | 343 | 10 | 3.44E-01 | 1.000 | guanyl nucleotide binding |
| GO:0033554 | PROCESS | 442 | 12 | 3.44E-01 | 1.000 | cellular response to stress |
| GO:0009411 | PROCESS | 49 | 2 | 3.45E-01 | 1.000 | response to UV |

| | | | | | | |
|---|---|---|---|---|---|---|
| GO:0051325 | PROCESS | 84 | 3 | 3.45E-01 | 1.000 | interphase |
| GO:0045121 | CELLULAR | 82 | 5 | 3.45E-01 | 1.000 | membrane raft |
| GO:0034984 | PROCESS | 299 | 8 | 3.46E-01 | 1.000 | cellular response to DNA damage stimulus |
| GO:0030551 | FUNCTION | 20 | 2 | 3.46E-01 | 1.000 | cyclic nucleotide binding |
| GO:0001508 | PROCESS | 49 | 3 | 3.48E-01 | 1.000 | regulation of action potential |
| GO:0034470 | PROCESS | 175 | 4 | 3.49E-01 | 1.000 | ncRNA processing |
| GO:0004867 | FUNCTION | 88 | 3 | 3.49E-01 | 1.000 | serine-type endopeptidase inhibitor activity |
| GO:0000793 | CELLULAR | 102 | 3 | 3.49E-01 | 1.000 | condensed chromosome |
| GO:0031072 | FUNCTION | 63 | 2 | 3.51E-01 | 1.000 | heat shock protein binding |
| GO:0042391 | PROCESS | 89 | 5 | 3.52E-01 | 1.000 | regulation of membrane potential |
| GO:0019226 | PROCESS | 276 | 15 | 3.52E-01 | 1.000 | transmission of nerve impulse |
| GO:0032940 | PROCESS | 147 | 7 | 3.53E-01 | 1.000 | secretion by cell |
| GO:0048872 | PROCESS | 61 | 2 | 3.53E-01 | 1.000 | homeostasis of number of cells |
| GO:0014070 | PROCESS | 29 | 2 | 3.53E-01 | 1.000 | response to organic cyclic substance |
| GO:0070302 | PROCESS | 54 | 2 | 3.53E-01 | 1.000 | regulation of stress-activated protein kinase signaling pathway |
| GO:0051046 | PROCESS | 133 | 5 | 3.57E-01 | 1.000 | regulation of secretion |
| GO:0060341 | PROCESS | 174 | 6 | 3.59E-01 | 1.000 | regulation of cellular localization |
| GO:0005525 | FUNCTION | 334 | 9 | 3.59E-01 | 1.000 | GTP binding |
| GO:0051899 | PROCESS | 19 | 2 | 3.59E-01 | 1.000 | membrane depolarization |
| GO:0008376 | FUNCTION | 31 | 3 | 3.60E-01 | 1.000 | acetylgalactosaminyltransferase activity |
| GO:0016776 | FUNCTION | 29 | 2 | 3.65E-01 | 1.000 | phosphotransferase activity, phosphate group as acceptor |
| GO:0008201 | FUNCTION | 88 | 4 | 3.65E-01 | 1.000 | heparin binding |
| GO:0031253 | CELLULAR | 40 | 2 | 3.65E-01 | 1.000 | cell projection membrane |
| GO:0002521 | PROCESS | 88 | 4 | 3.65E-01 | 1.000 | leukocyte differentiation |
| GO:0030324 | PROCESS | 67 | 3 | 3.65E-01 | 1.000 | lung development |
| GO:0044238 | PROCESS | 4857 | 124 | 3.65E-01 | 1.000 | primary metabolic process |
| GO:0030054 | CELLULAR | 461 | 26 | 3.65E-01 | 1.000 | cell junction |
| GO:0005834 | CELLULAR | 26 | 2 | 3.66E-01 | 1.000 | heterotrimeric G-protein complex |
| GO:0007596 | PROCESS | 82 | 3 | 3.67E-01 | 1.000 | blood coagulation |
| GO:0050817 | PROCESS | 82 | 3 | 3.67E-01 | 1.000 | coagulation |
| GO:0048660 | PROCESS | 25 | 2 | 3.68E-01 | 1.000 | regulation of smooth muscle cell proliferation |
| GO:0016835 | FUNCTION | 50 | 2 | 3.71E-01 | 1.000 | carbon-oxygen lyase activity |
| GO:0007189 | PROCESS | 33 | 2 | 3.71E-01 | 1.000 | activation of adenylate cyclase activity by G-protein signaling pathway |
| GO:0010579 | PROCESS | 33 | 2 | 3.71E-01 | 1.000 | positive regulation of adenylate cyclase activity by G-protein signaling pathway |
| GO:0010578 | PROCESS | 33 | 2 | 3.71E-01 | 1.000 | regulation of adenylate cyclase activity involved in G-protein signaling |
| GO:0034704 | CELLULAR | 26 | 3 | 3.72E-01 | 1.000 | calcium channel complex |
| GO:0051592 | PROCESS | 37 | 3 | 3.73E-01 | 1.000 | response to calcium ion |
| GO:0005768 | CELLULAR | 260 | 8 | 3.76E-01 | 1.000 | endosome |
| GO:0045165 | PROCESS | 107 | 4 | 3.78E-01 | 1.000 | cell fate commitment |
| GO:0044421 | CELLULAR | 842 | 29 | 3.78E-01 | 1.000 | extracellular region part |
| GO:0030832 | PROCESS | 51 | 2 | 3.78E-01 | 1.000 | regulation of actin filament length |
| GO:0007188 | PROCESS | 67 | 3 | 3.79E-01 | 1.000 | G-protein signaling, coupled to cAMP nucleotide second messenger |
| GO:0019992 | FUNCTION | 64 | 5 | 3.79E-01 | 1.000 | diacylglycerol binding |
| GO:0007600 | PROCESS | 703 | 18 | 3.82E-01 | 1.000 | sensory perception |
| GO:0030695 | FUNCTION | 364 | 18 | 3.83E-01 | 1.000 | GTPase regulator activity |
| GO:0045637 | PROCESS | 53 | 2 | 3.83E-01 | 1.000 | regulation of myeloid cell differentiation |
| GO:0051223 | PROCESS | 90 | 3 | 3.84E-01 | 1.000 | regulation of protein transport |
| GO:0010975 | PROCESS | 42 | 3 | 3.86E-01 | 1.000 | regulation of neuron projection development |
| GO:0050804 | PROCESS | 65 | 4 | 3.87E-01 | 1.000 | regulation of synaptic transmission |
| GO:0016462 | FUNCTION | 684 | 23 | 3.87E-01 | 1.000 | pyrophosphatase activity |
| GO:0009605 | PROCESS | 677 | 21 | 3.89E-01 | 1.000 | response to external stimulus |
| GO:0050727 | PROCESS | 53 | 2 | 3.90E-01 | 1.000 | regulation of inflammatory response |
| GO:0030659 | CELLULAR | 112 | 5 | 3.91E-01 | 1.000 | cytoplasmic vesicle membrane |
| GO:0003677 | FUNCTION | 2132 | 48 | 3.91E-01 | 1.000 | DNA binding |
| GO:0016811 | FUNCTION | 54 | 2 | 3.92E-01 | 1.000 | hydrolase activity, acting on carbon-nitrogen (but not peptide) bonds, in linear amides |
| GO:0016887 | FUNCTION | 299 | 12 | 3.93E-01 | 1.000 | ATPase activity |
| GO:0051094 | PROCESS | 504 | 17 | 3.94E-01 | 1.000 | positive regulation of developmental process |
| GO:0080090 | PROCESS | 2941 | 73 | 3.94E-01 | 1.000 | regulation of primary metabolic process |
| GO:0015837 | PROCESS | 97 | 4 | 3.95E-01 | 1.000 | amine transport |
| GO:0006355 | PROCESS | 1570 | 38 | 3.95E-01 | 1.000 | regulation of transcription, DNA-dependent |
| GO:0007254 | PROCESS | 47 | 2 | 3.97E-01 | 1.000 | JNK cascade |
| GO:0060589 | FUNCTION | 373 | 18 | 3.98E-01 | 1.000 | nucleoside-triphosphatase regulator activity |
| GO:0016818 | FUNCTION | 687 | 23 | 3.99E-01 | 1.000 | hydrolase activity, acting on acid anhydrides, in phosphorus-containing anhydrides |
| GO:0016798 | FUNCTION | 103 | 3 | 3.99E-01 | 1.000 | hydrolase activity, acting on glycosyl bonds |
| GO:0007599 | PROCESS | 86 | 3 | 3.99E-01 | 1.000 | hemostasis |
| GO:0050921 | PROCESS | 24 | 2 | 4.01E-01 | 1.000 | positive regulation of chemotaxis |
| GO:0040017 | PROCESS | 24 | 2 | 4.01E-01 | 1.000 | positive regulation of locomotion |
| GO:0004012 | FUNCTION | 14 | 2 | 4.02E-01 | 1.000 | phospholipid-translocating ATPase activity |
| GO:0015247 | FUNCTION | 14 | 2 | 4.02E-01 | 1.000 | aminophospholipid transporter activity |
| GO:0006397 | PROCESS | 296 | 7 | 4.02E-01 | 1.000 | mRNA processing |
| GO:0042157 | PROCESS | 68 | 2 | 4.03E-01 | 1.000 | lipoprotein metabolic process |
| GO:0042127 | PROCESS | 603 | 18 | 4.03E-01 | 1.000 | regulation of cell proliferation |
| GO:0003014 | PROCESS | 19 | 2 | 4.03E-01 | 1.000 | renal system process |
| GO:0005083 | FUNCTION | 235 | 12 | 4.04E-01 | 1.000 | small GTPase regulator activity |
| GO:0015630 | CELLULAR | 467 | 14 | 4.04E-01 | 1.000 | microtubule cytoskeleton |
| GO:0016817 | FUNCTION | 691 | 23 | 4.05E-01 | 1.000 | hydrolase activity, acting on acid anhydrides |

| | | | | | | |
|---|---|---|---|---|---|---|
| GO:0046982 | FUNCTION | 139 | 5 | 4.05E-01 | 1.000 | protein heterodimerization activity |
| GO:0043069 | PROCESS | 277 | 8 | 4.05E-01 | 1.000 | negative regulation of programmed cell death |
| GO:0060548 | PROCESS | 277 | 8 | 4.05E-01 | 1.000 | negative regulation of cell death |
| GO:0015698 | PROCESS | 73 | 3 | 4.06E-01 | 1.000 | inorganic anion transport |
| GO:0005739 | CELLULAR | 949 | 21 | 4.06E-01 | 1.000 | mitochondrion |
| GO:0044429 | CELLULAR | 517 | 11 | 4.07E-01 | 1.000 | mitochondrial part |
| GO:0005044 | FUNCTION | 39 | 2 | 4.09E-01 | 1.000 | scavenger receptor activity |
| GO:0005529 | FUNCTION | 172 | 6 | 4.09E-01 | 1.000 | sugar binding |
| GO:0010605 | PROCESS | 589 | 17 | 4.11E-01 | 1.000 | negative regulation of macromolecule metabolic process |
| GO:0042981 | PROCESS | 607 | 18 | 4.12E-01 | 1.000 | regulation of apoptosis |
| GO:0030323 | PROCESS | 69 | 3 | 4.14E-01 | 1.000 | respiratory tube development |
| GO:0070201 | PROCESS | 96 | 3 | 4.14E-01 | 1.000 | regulation of establishment of protein localization |
| GO:0009314 | PROCESS | 146 | 5 | 4.15E-01 | 1.000 | response to radiation |
| GO:0045017 | PROCESS | 69 | 2 | 4.16E-01 | 1.000 | glycerolipid biosynthetic process |
| GO:0009790 | PROCESS | 405 | 13 | 4.16E-01 | 1.000 | embryonic development |
| GO:0007200 | PROCESS | 43 | 2 | 4.17E-01 | 1.000 | activation of phospholipase C activity by G-protein coupled receptor protein signaling pathway coupled to IP3 second messenger |
| GO:0009205 | PROCESS | 100 | 4 | 4.17E-01 | 1.000 | purine ribonucleoside triphosphate metabolic process |
| GO:0048520 | PROCESS | 27 | 2 | 4.17E-01 | 1.000 | positive regulation of behavior |
| GO:0010769 | PROCESS | 49 | 3 | 4.18E-01 | 1.000 | regulation of cell morphogenesis involved in differentiation |
| GO:0009199 | PROCESS | 101 | 4 | 4.20E-01 | 1.000 | ribonucleoside triphosphate metabolic process |
| GO:0050920 | PROCESS | 27 | 2 | 4.20E-01 | 1.000 | regulation of chemotaxis |
| GO:0042221 | PROCESS | 868 | 27 | 4.21E-01 | 1.000 | response to chemical stimulus |
| GO:0004866 | FUNCTION | 134 | 4 | 4.21E-01 | 1.000 | endopeptidase inhibitor activity |
| GO:0006974 | PROCESS | 329 | 8 | 4.21E-01 | 1.000 | response to DNA damage stimulus |
| GO:0046649 | PROCESS | 135 | 5 | 4.21E-01 | 1.000 | lymphocyte activation |
| GO:0008033 | PROCESS | 72 | 2 | 4.21E-01 | 1.000 | tRNA processing |
| GO:0009144 | PROCESS | 101 | 4 | 4.21E-01 | 1.000 | purine nucleoside triphosphate metabolic process |
| GO:0051969 | PROCESS | 72 | 4 | 4.22E-01 | 1.000 | regulation of transmission of nerve impulse |
| GO:0060541 | PROCESS | 73 | 3 | 4.22E-01 | 1.000 | respiratory system development |
| GO:0031098 | PROCESS | 50 | 2 | 4.23E-01 | 1.000 | stress-activated protein kinase signaling pathway |
| GO:0016071 | PROCESS | 339 | 8 | 4.25E-01 | 1.000 | mRNA metabolic process |
| GO:0044430 | CELLULAR | 797 | 24 | 4.27E-01 | 1.000 | cytoskeletal part |
| GO:0004252 | FUNCTION | 150 | 4 | 4.28E-01 | 1.000 | serine-type endopeptidase activity |
| GO:0055085 | PROCESS | 204 | 6 | 4.28E-01 | 1.000 | transmembrane transport |
| GO:0007050 | PROCESS | 95 | 3 | 4.29E-01 | 1.000 | cell cycle arrest |
| GO:0030099 | PROCESS | 64 | 2 | 4.29E-01 | 1.000 | myeloid cell differentiation |
| GO:0007166 | PROCESS | 1577 | 46 | 4.30E-01 | 1.000 | cell surface receptor linked signal transduction |
| GO:0043176 | FUNCTION | 80 | 3 | 4.30E-01 | 1.000 | amine binding |
| GO:0032269 | PROCESS | 126 | 4 | 4.31E-01 | 1.000 | negative regulation of cellular protein metabolic process |
| GO:0019932 | PROCESS | 199 | 7 | 4.31E-01 | 1.000 | second-messenger-mediated signaling |
| GO:0031644 | PROCESS | 75 | 4 | 4.33E-01 | 1.000 | regulation of neurological system process |
| GO:0051028 | PROCESS | 75 | 2 | 4.33E-01 | 1.000 | mRNA transport |
| GO:0016568 | PROCESS | 246 | 7 | 4.33E-01 | 1.000 | chromatin modification |
| GO:0005743 | CELLULAR | 243 | 5 | 4.34E-01 | 1.000 | mitochondrial inner membrane |
| GO:0007155 | PROCESS | 647 | 34 | 4.35E-01 | 1.000 | cell adhesion |
| GO:0001936 | PROCESS | 29 | 2 | 4.35E-01 | 1.000 | regulation of endothelial cell proliferation |
| GO:0022610 | PROCESS | 648 | 34 | 4.36E-01 | 1.000 | biological adhesion |
| GO:0009755 | PROCESS | 48 | 2 | 4.39E-01 | 1.000 | hormone-mediated signaling |
| GO:0012506 | CELLULAR | 120 | 5 | 4.39E-01 | 1.000 | vesicle membrane |
| GO:0048754 | PROCESS | 42 | 2 | 4.43E-01 | 1.000 | branching morphogenesis of a tube |
| GO:0006396 | PROCESS | 509 | 11 | 4.43E-01 | 1.000 | RNA processing |
| GO:0022603 | PROCESS | 231 | 9 | 4.46E-01 | 1.000 | regulation of anatomical structure morphogenesis |
| GO:0005516 | FUNCTION | 130 | 7 | 4.47E-01 | 1.000 | calmodulin binding |
| GO:0006865 | PROCESS | 74 | 3 | 4.48E-01 | 1.000 | amino acid transport |
| GO:0033559 | PROCESS | 50 | 2 | 4.50E-01 | 1.000 | unsaturated fatty acid metabolic process |
| GO:0005795 | CELLULAR | 40 | 2 | 4.51E-01 | 1.000 | Golgi stack |
| GO:0046907 | PROCESS | 548 | 15 | 4.51E-01 | 1.000 | intracellular transport |
| GO:0004713 | FUNCTION | 147 | 7 | 4.52E-01 | 1.000 | protein tyrosine kinase activity |
| GO:0035239 | PROCESS | 70 | 3 | 4.52E-01 | 1.000 | tube morphogenesis |
| GO:0031344 | PROCESS | 54 | 3 | 4.52E-01 | 1.000 | regulation of cell projection organization |
| GO:0010646 | PROCESS | 801 | 28 | 4.53E-01 | 1.000 | regulation of cell communication |
| GO:0050795 | PROCESS | 33 | 2 | 4.53E-01 | 1.000 | regulation of behavior |
| GO:0016604 | CELLULAR | 150 | 4 | 4.53E-01 | 1.000 | nuclear body |
| GO:0009141 | PROCESS | 107 | 4 | 4.54E-01 | 1.000 | nucleoside triphosphate metabolic process |
| GO:0050870 | PROCESS | 57 | 2 | 4.55E-01 | 1.000 | positive regulation of T cell activation |
| GO:0019205 | FUNCTION | 39 | 2 | 4.56E-01 | 1.000 | nucleobase, nucleoside, nucleotide kinase activity |
| GO:0030528 | FUNCTION | 1359 | 36 | 4.57E-01 | 1.000 | transcription regulator activity |
| GO:0042578 | FUNCTION | 313 | 13 | 4.58E-01 | 1.000 | phosphoric ester hydrolase activity |
| GO:0032787 | PROCESS | 266 | 8 | 4.59E-01 | 1.000 | monocarboxylic acid metabolic process |
| GO:0006936 | PROCESS | 123 | 6 | 4.60E-01 | 1.000 | muscle contraction |
| GO:0046395 | PROCESS | 98 | 3 | 4.60E-01 | 1.000 | carboxylic acid catabolic process |
| GO:0016054 | PROCESS | 98 | 3 | 4.60E-01 | 1.000 | organic acid catabolic process |
| GO:0045211 | CELLULAR | 119 | 9 | 4.60E-01 | 1.000 | postsynaptic membrane |
| GO:0050900 | PROCESS | 48 | 2 | 4.61E-01 | 1.000 | leukocyte migration |
| GO:0022604 | PROCESS | 97 | 5 | 4.61E-01 | 1.000 | regulation of cell morphogenesis |
| GO:0019933 | PROCESS | 73 | 3 | 4.61E-01 | 1.000 | cAMP-mediated signaling |
| GO:0043254 | PROCESS | 62 | 2 | 4.62E-01 | 1.000 | regulation of protein complex assembly |
| GO:0015082 | FUNCTION | 31 | 2 | 4.62E-01 | 1.000 | di-, tri-valent inorganic cation transmembrane transporter activity |

| | | | | | | |
|---|---|---|---|---|---|---|
| GO:0019222 | PROCESS | 3218 | 79 | 4.64E-01 | 1.000 | regulation of metabolic process |
| GO:0030098 | PROCESS | 69 | 3 | 4.64E-01 | 1.000 | lymphocyte differentiation |
| GO:0030855 | PROCESS | 57 | 2 | 4.66E-01 | 1.000 | epithelial cell differentiation |
| GO:0006650 | PROCESS | 95 | 3 | 4.67E-01 | 1.000 | glycerophospholipid metabolic process |
| GO:0035295 | PROCESS | 130 | 5 | 4.67E-01 | 1.000 | tube development |
| GO:0007160 | PROCESS | 79 | 4 | 4.68E-01 | 1.000 | cell-matrix adhesion |
| GO:0048193 | PROCESS | 110 | 3 | 4.69E-01 | 1.000 | Golgi vesicle transport |
| GO:0035257 | FUNCTION | 69 | 2 | 4.70E-01 | 1.000 | nuclear hormone receptor binding |
| GO:0030817 | PROCESS | 83 | 4 | 4.72E-01 | 1.000 | regulation of cAMP biosynthetic process |
| GO:0051247 | PROCESS | 163 | 5 | 4.73E-01 | 1.000 | positive regulation of protein metabolic process |
| GO:0031667 | PROCESS | 102 | 3 | 4.76E-01 | 1.000 | response to nutrient levels |
| GO:0048534 | PROCESS | 183 | 6 | 4.77E-01 | 1.000 | hemopoietic or lymphoid organ development |
| GO:0032313 | PROCESS | 43 | 2 | 4.79E-01 | 1.000 | regulation of Rab GTPase activity |
| GO:0032483 | PROCESS | 43 | 2 | 4.79E-01 | 1.000 | regulation of Rab protein signal transduction |
| GO:0004175 | FUNCTION | 354 | 10 | 4.79E-01 | 1.000 | endopeptidase activity |
| GO:0045765 | PROCESS | 83 | 3 | 4.79E-01 | 1.000 | regulation of angiogenesis |
| GO:0003723 | FUNCTION | 655 | 15 | 4.80E-01 | 1.000 | RNA binding |
| GO:0006357 | PROCESS | 592 | 16 | 4.81E-01 | 1.000 | regulation of transcription from RNA polymerase II promoter |
| GO:0005097 | FUNCTION | 44 | 2 | 4.81E-01 | 1.000 | Rab GTPase activator activity |
| GO:0009165 | PROCESS | 165 | 6 | 4.82E-01 | 1.000 | nucleotide biosynthetic process |
| GO:0005262 | FUNCTION | 73 | 6 | 4.82E-01 | 1.000 | calcium channel activity |
| GO:0017038 | PROCESS | 108 | 3 | 4.82E-01 | 1.000 | protein import |
| GO:0016879 | FUNCTION | 203 | 6 | 4.83E-01 | 1.000 | ligase activity, forming carbon-nitrogen bonds |
| GO:0009150 | PROCESS | 118 | 4 | 4.84E-01 | 1.000 | purine ribonucleotide metabolic process |
| GO:0006937 | PROCESS | 54 | 3 | 4.84E-01 | 1.000 | regulation of muscle contraction |
| GO:0006511 | PROCESS | 207 | 5 | 4.85E-01 | 1.000 | ubiquitin-dependent protein catabolic process |
| GO:0003824 | FUNCTION | 4762 | 134 | 4.85E-01 | 1.000 | catalytic activity |
| GO:0046930 | CELLULAR | 86 | 2 | 4.85E-01 | 1.000 | pore complex |
| GO:0006576 | PROCESS | 81 | 3 | 4.85E-01 | 1.000 | biogenic amine metabolic process |
| GO:0009791 | PROCESS | 39 | 2 | 4.86E-01 | 1.000 | post-embryonic development |
| GO:0014706 | PROCESS | 95 | 4 | 4.87E-01 | 1.000 | striated muscle tissue development |
| GO:0001822 | PROCESS | 64 | 3 | 4.88E-01 | 1.000 | kidney development |
| GO:0051649 | PROCESS | 697 | 20 | 4.89E-01 | 1.000 | establishment of localization in cell |
| GO:0006940 | PROCESS | 27 | 2 | 4.89E-01 | 1.000 | regulation of smooth muscle contraction |
| GO:0006725 | PROCESS | 107 | 4 | 4.92E-01 | 1.000 | cellular aromatic compound metabolic process |
| GO:0019866 | CELLULAR | 259 | 5 | 4.92E-01 | 1.000 | organelle inner membrane |
| GO:0000075 | PROCESS | 74 | 2 | 4.93E-01 | 1.000 | cell cycle checkpoint |
| GO:0050793 | PROCESS | 1051 | 33 | 4.93E-01 | 1.000 | regulation of developmental process |
| GO:0032501 | PROCESS | 3427 | 108 | 4.95E-01 | 1.000 | multicellular organismal process |
| GO:0004518 | FUNCTION | 147 | 3 | 4.95E-01 | 1.000 | nuclease activity |
| GO:0007601 | PROCESS | 205 | 8 | 4.95E-01 | 1.000 | visual perception |
| GO:0050953 | PROCESS | 205 | 8 | 4.95E-01 | 1.000 | sensory perception of light stimulus |
| GO:0030529 | CELLULAR | 442 | 7 | 4.96E-01 | 1.000 | ribonucleoprotein complex |
| GO:0010468 | PROCESS | 2575 | 60 | 4.97E-01 | 1.000 | regulation of gene expression |
| GO:0050890 | PROCESS | 760 | 19 | 4.97E-01 | 1.000 | cognition |
| GO:0032880 | PROCESS | 107 | 3 | 4.97E-01 | 1.000 | regulation of protein localization |
| GO:0009187 | PROCESS | 31 | 2 | 4.98E-01 | 1.000 | cyclic nucleotide metabolic process |
| GO:0007049 | PROCESS | 696 | 15 | 4.98E-01 | 1.000 | cell cycle |
| GO:0060249 | PROCESS | 45 | 2 | 4.99E-01 | 1.000 | anatomical structure homeostasis |
| GO:0050880 | PROCESS | 41 | 2 | 5.00E-01 | 1.000 | regulation of blood vessel size |
| GO:0035150 | PROCESS | 41 | 2 | 5.00E-01 | 1.000 | regulation of tube size |
| GO:0005891 | CELLULAR | 21 | 2 | 5.00E-01 | 1.000 | voltage-gated calcium channel complex |
| GO:0030814 | PROCESS | 86 | 4 | 5.02E-01 | 1.000 | regulation of cAMP metabolic process |
| GO:0006606 | PROCESS | 71 | 2 | 5.02E-01 | 1.000 | protein import into nucleus |
| GO:0008509 | FUNCTION | 131 | 6 | 5.04E-01 | 1.000 | anion transmembrane transporter activity |
| GO:0007584 | PROCESS | 66 | 2 | 5.05E-01 | 1.000 | response to nutrient |
| GO:0005576 | CELLULAR | 1837 | 53 | 5.06E-01 | 1.000 | extracellular region |
| GO:0007242 | PROCESS | 1082 | 35 | 5.06E-01 | 1.000 | intracellular signaling cascade |
| GO:0051251 | PROCESS | 68 | 2 | 5.07E-01 | 1.000 | positive regulation of lymphocyte activation |
| GO:0009124 | PROCESS | 45 | 2 | 5.08E-01 | 1.000 | nucleoside monophosphate biosynthetic process |
| GO:0051170 | PROCESS | 73 | 2 | 5.08E-01 | 1.000 | nuclear import |
| GO:0046394 | PROCESS | 139 | 4 | 5.10E-01 | 1.000 | carboxylic acid biosynthetic process |
| GO:0016053 | PROCESS | 139 | 4 | 5.10E-01 | 1.000 | organic acid biosynthetic process |
| GO:0006259 | PROCESS | 444 | 10 | 5.11E-01 | 1.000 | DNA metabolic process |
| GO:0005925 | CELLULAR | 89 | 4 | 5.14E-01 | 1.000 | focal adhesion |
| GO:0000904 | PROCESS | 158 | 9 | 5.14E-01 | 1.000 | cell morphogenesis involved in differentiation |
| GO:0060537 | PROCESS | 100 | 4 | 5.15E-01 | 1.000 | muscle tissue development |
| GO:0051427 | FUNCTION | 75 | 2 | 5.15E-01 | 1.000 | hormone receptor binding |
| GO:0002520 | PROCESS | 194 | 6 | 5.20E-01 | 1.000 | immune system development |
| GO:0030336 | PROCESS | 49 | 2 | 5.22E-01 | 1.000 | negative regulation of cell migration |
| GO:0045202 | CELLULAR | 266 | 17 | 5.23E-01 | 1.000 | synapse |
| GO:0031967 | CELLULAR | 520 | 11 | 5.23E-01 | 1.000 | organelle envelope |
| GO:0008022 | FUNCTION | 114 | 4 | 5.23E-01 | 1.000 | protein C-terminus binding |
| GO:0043066 | PROCESS | 271 | 7 | 5.23E-01 | 1.000 | negative regulation of apoptosis |
| GO:0001763 | PROCESS | 48 | 2 | 5.25E-01 | 1.000 | morphogenesis of a branching structure |
| GO:0031328 | PROCESS | 536 | 16 | 5.25E-01 | 1.000 | positive regulation of cellular biosynthetic process |
| GO:0005102 | FUNCTION | 771 | 22 | 5.26E-01 | 1.000 | receptor binding |
| GO:0031975 | CELLULAR | 522 | 11 | 5.26E-01 | 1.000 | envelope |
| GO:0005788 | CELLULAR | 75 | 2 | 5.27E-01 | 1.000 | endoplasmic reticulum lumen |

| | | | | | | |
|---|---|---|---|---|---|---|
| GO:0032555 | FUNCTION | 1693 | 53 | 5.28E-01 | 1.000 | purine ribonucleotide binding |
| GO:0032553 | FUNCTION | 1693 | 53 | 5.28E-01 | 1.000 | ribonucleotide binding |
| GO:0019899 | FUNCTION | 405 | 14 | 5.29E-01 | 1.000 | enzyme binding |
| GO:0004725 | FUNCTION | 92 | 5 | 5.29E-01 | 1.000 | protein tyrosine phosphatase activity |
| GO:0030203 | PROCESS | 47 | 2 | 5.29E-01 | 1.000 | glycosaminoglycan metabolic process |
| GO:0001655 | PROCESS | 74 | 3 | 5.30E-01 | 1.000 | urogenital system development |
| GO:0006575 | PROCESS | 138 | 4 | 5.31E-01 | 1.000 | cellular amino acid derivative metabolic process |
| GO:0044444 | CELLULAR | 4282 | 107 | 5.33E-01 | 1.000 | cytoplasmic part |
| GO:0031323 | PROCESS | 3082 | 74 | 5.33E-01 | 1.000 | regulation of cellular metabolic process |
| GO:0009891 | PROCESS | 539 | 16 | 5.34E-01 | 1.000 | positive regulation of biosynthetic process |
| GO:0007283 | PROCESS | 229 | 5 | 5.34E-01 | 1.000 | spermatogenesis |
| GO:0048232 | PROCESS | 229 | 5 | 5.34E-01 | 1.000 | male gamete generation |
| GO:0003018 | PROCESS | 44 | 2 | 5.37E-01 | 1.000 | vascular process in circulatory system |
| GO:0007190 | PROCESS | 47 | 2 | 5.38E-01 | 1.000 | activation of adenylate cyclase activity |
| GO:0007202 | PROCESS | 55 | 2 | 5.39E-01 | 1.000 | activation of phospholipase C activity |
| GO:0010863 | PROCESS | 55 | 2 | 5.39E-01 | 1.000 | positive regulation of phospholipase C activity |
| GO:0051641 | PROCESS | 764 | 22 | 5.40E-01 | 1.000 | cellular localization |
| GO:0016324 | CELLULAR | 81 | 4 | 5.40E-01 | 1.000 | apical plasma membrane |
| GO:0033365 | PROCESS | 119 | 3 | 5.40E-01 | 1.000 | protein localization in organelle |
| GO:0010518 | PROCESS | 56 | 2 | 5.42E-01 | 1.000 | positive regulation of phospholipase activity |
| GO:0045762 | PROCESS | 48 | 2 | 5.45E-01 | 1.000 | positive regulation of adenylate cyclase activity |
| GO:0048167 | PROCESS | 29 | 2 | 5.45E-01 | 1.000 | regulation of synaptic plasticity |
| GO:0007264 | PROCESS | 248 | 7 | 5.45E-01 | 1.000 | small GTPase mediated signal transduction |
| GO:0045595 | PROCESS | 352 | 12 | 5.46E-01 | 1.000 | regulation of cell differentiation |
| GO:0031589 | PROCESS | 89 | 4 | 5.47E-01 | 1.000 | cell-substrate adhesion |
| GO:0032559 | FUNCTION | 1380 | 46 | 5.48E-01 | 1.000 | adenyl ribonucleotide binding |
| GO:0050839 | FUNCTION | 21 | 3 | 5.48E-01 | 1.000 | cell adhesion molecule binding |
| GO:0016477 | PROCESS | 196 | 8 | 5.48E-01 | 1.000 | cell migration |
| GO:0051186 | PROCESS | 169 | 4 | 5.49E-01 | 1.000 | cofactor metabolic process |
| GO:0031281 | PROCESS | 49 | 2 | 5.50E-01 | 1.000 | positive regulation of cyclase activity |
| GO:0051093 | PROCESS | 445 | 13 | 5.50E-01 | 1.000 | negative regulation of developmental process |
| GO:0008380 | PROCESS | 258 | 5 | 5.50E-01 | 1.000 | RNA splicing |
| GO:0004553 | FUNCTION | 81 | 2 | 5.51E-01 | 1.000 | hydrolase activity, hydrolyzing O-glycosyl compounds |
| GO:0010517 | PROCESS | 58 | 2 | 5.51E-01 | 1.000 | regulation of phospholipase activity |
| GO:0007010 | PROCESS | 355 | 12 | 5.52E-01 | 1.000 | cytoskeleton organization |
| GO:0032386 | PROCESS | 60 | 2 | 5.52E-01 | 1.000 | regulation of intracellular transport |
| GO:0046483 | PROCESS | 280 | 9 | 5.53E-01 | 1.000 | heterocycle metabolic process |
| GO:0019219 | PROCESS | 2514 | 59 | 5.53E-01 | 1.000 | regulation of nucleobase, nucleoside, nucleotide and nucleic acid metabolic process |
| GO:0006310 | PROCESS | 88 | 2 | 5.56E-01 | 1.000 | DNA recombination |
| GO:0004222 | FUNCTION | 98 | 4 | 5.57E-01 | 1.000 | metalloendopeptidase activity |
| GO:0005912 | CELLULAR | 130 | 7 | 5.58E-01 | 1.000 | adherens junction |
| GO:0016829 | FUNCTION | 139 | 4 | 5.58E-01 | 1.000 | lyase activity |
| GO:0034504 | PROCESS | 79 | 2 | 5.59E-01 | 1.000 | protein localization in nucleus |
| GO:0015294 | FUNCTION | 74 | 3 | 5.61E-01 | 1.000 | solute:cation symporter activity |
| GO:0051349 | PROCESS | 51 | 2 | 5.61E-01 | 1.000 | positive regulation of lyase activity |
| GO:0005178 | FUNCTION | 51 | 2 | 5.61E-01 | 1.000 | integrin binding |
| GO:0006886 | PROCESS | 323 | 8 | 5.61E-01 | 1.000 | intracellular protein transport |
| GO:0009206 | PROCESS | 87 | 3 | 5.61E-01 | 1.000 | purine ribonucleoside triphosphate biosynthetic process |
| GO:0009145 | PROCESS | 87 | 3 | 5.61E-01 | 1.000 | purine nucleoside triphosphate biosynthetic process |
| GO:0048598 | PROCESS | 220 | 6 | 5.61E-01 | 1.000 | embryonic morphogenesis |
| GO:0005924 | CELLULAR | 93 | 4 | 5.63E-01 | 1.000 | cell-substrate adherens junction |
| GO:0009201 | PROCESS | 88 | 3 | 5.64E-01 | 1.000 | ribonucleoside triphosphate biosynthetic process |
| GO:0009142 | PROCESS | 88 | 3 | 5.64E-01 | 1.000 | nucleoside triphosphate biosynthetic process |
| GO:0046700 | PROCESS | 56 | 2 | 5.65E-01 | 1.000 | heterocycle catabolic process |
| GO:0006820 | PROCESS | 116 | 4 | 5.66E-01 | 1.000 | anion transport |
| GO:0016791 | FUNCTION | 231 | 8 | 5.68E-01 | 1.000 | phosphatase activity |
| GO:0031324 | PROCESS | 567 | 15 | 5.68E-01 | 1.000 | negative regulation of cellular metabolic process |
| GO:0002696 | PROCESS | 76 | 2 | 5.68E-01 | 1.000 | positive regulation of leukocyte activation |
| GO:0051015 | FUNCTION | 38 | 2 | 5.70E-01 | 1.000 | actin filament binding |
| GO:0007565 | PROCESS | 76 | 2 | 5.71E-01 | 1.000 | female pregnancy |
| GO:0042623 | FUNCTION | 238 | 8 | 5.72E-01 | 1.000 | ATPase activity, coupled |
| GO:0051129 | PROCESS | 114 | 3 | 5.72E-01 | 1.000 | negative regulation of cellular component organization |
| GO:0051239 | PROCESS | 689 | 22 | 5.72E-01 | 1.000 | regulation of multicellular organismal process |
| GO:0007215 | PROCESS | 22 | 2 | 5.74E-01 | 1.000 | glutamate signaling pathway |
| GO:0005783 | CELLULAR | 867 | 23 | 5.74E-01 | 1.000 | endoplasmic reticulum |
| GO:0032956 | PROCESS | 66 | 2 | 5.75E-01 | 1.000 | regulation of actin cytoskeleton organization |
| GO:0051241 | PROCESS | 100 | 3 | 5.75E-01 | 1.000 | negative regulation of multicellular organismal process |
| GO:0009991 | PROCESS | 118 | 3 | 5.75E-01 | 1.000 | response to extracellular stimulus |
| GO:0048870 | PROCESS | 223 | 9 | 5.76E-01 | 1.000 | cell motility |
| GO:0045182 | FUNCTION | 106 | 2 | 5.79E-01 | 1.000 | translation regulator activity |
| GO:0030097 | PROCESS | 163 | 5 | 5.79E-01 | 1.000 | hemopoiesis |
| GO:0003012 | PROCESS | 153 | 6 | 5.80E-01 | 1.000 | muscle system process |
| GO:0030326 | PROCESS | 54 | 2 | 5.80E-01 | 1.000 | embryonic limb morphogenesis |
| GO:0035113 | PROCESS | 54 | 2 | 5.80E-01 | 1.000 | embryonic appendage morphogenesis |
| GO:0051171 | PROCESS | 2531 | 59 | 5.81E-01 | 1.000 | regulation of nitrogen compound metabolic process |
| GO:0009893 | PROCESS | 681 | 20 | 5.81E-01 | 1.000 | positive regulation of metabolic process |
| GO:0006519 | PROCESS | 316 | 8 | 5.82E-01 | 1.000 | cellular amino acid and derivative metabolic process |
| GO:0048646 | PROCESS | 244 | 9 | 5.82E-01 | 1.000 | anatomical structure formation involved in morphogenesis |

| | | | | | | |
|---|---|---|---|---|---|---|
| GO:0000166 | FUNCTION | 2046 | 61 | 5.83E-01 | 1.000 | nucleotide binding |
| GO:0017076 | FUNCTION | 1768 | 54 | 5.85E-01 | 1.000 | purine nucleotide binding |
| GO:0032970 | PROCESS | 69 | 2 | 5.85E-01 | 1.000 | regulation of actin filament-based process |
| GO:0051051 | PROCESS | 96 | 3 | 5.85E-01 | 1.000 | negative regulation of transport |
| GO:0005125 | FUNCTION | 191 | 3 | 5.85E-01 | 1.000 | cytokine activity |
| GO:0019216 | PROCESS | 86 | 3 | 5.86E-01 | 1.000 | regulation of lipid metabolic process |
| GO:0030055 | CELLULAR | 96 | 4 | 5.86E-01 | 1.000 | cell-substrate junction |
| GO:0030136 | CELLULAR | 101 | 4 | 5.86E-01 | 1.000 | clathrin-coated vesicle |
| GO:0009259 | PROCESS | 127 | 4 | 5.87E-01 | 1.000 | ribonucleotide metabolic process |
| GO:0046777 | PROCESS | 57 | 2 | 5.90E-01 | 1.000 | protein amino acid autophosphorylation |
| GO:0015631 | FUNCTION | 72 | 2 | 5.90E-01 | 1.000 | tubulin binding |
| GO:0009966 | PROCESS | 716 | 23 | 5.92E-01 | 1.000 | regulation of signal transduction |
| GO:0009581 | PROCESS | 60 | 2 | 5.93E-01 | 1.000 | detection of external stimulus |
| GO:0006022 | PROCESS | 57 | 2 | 5.94E-01 | 1.000 | aminoglycan metabolic process |
| GO:0051716 | PROCESS | 604 | 15 | 5.95E-01 | 1.000 | cellular response to stimulus |
| GO:0045321 | PROCESS | 168 | 5 | 5.96E-01 | 1.000 | leukocyte activation |
| GO:0050867 | PROCESS | 79 | 2 | 5.97E-01 | 1.000 | positive regulation of cell activation |
| GO:0004970 | FUNCTION | 17 | 2 | 5.98E-01 | 1.000 | ionotropic glutamate receptor activity |
| GO:0005234 | FUNCTION | 18 | 2 | 5.99E-01 | 1.000 | extracellular-glutamate-gated ion channel activity |
| GO:0003676 | FUNCTION | 2957 | 62 | 6.01E-01 | 1.000 | nucleic acid binding |
| GO:0045935 | PROCESS | 484 | 14 | 6.01E-01 | 1.000 | positive regulation of nucleobase, nucleoside, nucleotide and nucleic acid metabolic process |
| GO:0031327 | PROCESS | 459 | 12 | 6.01E-01 | 1.000 | negative regulation of cellular biosynthetic process |
| GO:0010033 | PROCESS | 417 | 12 | 6.01E-01 | 1.000 | response to organic substance |
| GO:0010038 | PROCESS | 67 | 3 | 6.02E-01 | 1.000 | response to metal ion |
| GO:0010647 | PROCESS | 253 | 7 | 6.02E-01 | 1.000 | positive regulation of cell communication |
| GO:0042493 | PROCESS | 84 | 3 | 6.03E-01 | 1.000 | response to drug |
| GO:0030554 | FUNCTION | 1453 | 47 | 6.04E-01 | 1.000 | adenyl nucleotide binding |
| GO:0006730 | PROCESS | 89 | 2 | 6.05E-01 | 1.000 | one-carbon metabolic process |
| GO:0030036 | PROCESS | 188 | 7 | 6.05E-01 | 1.000 | actin cytoskeleton organization |
| GO:0042445 | PROCESS | 87 | 3 | 6.06E-01 | 1.000 | hormone metabolic process |
| GO:0040011 | PROCESS | 333 | 11 | 6.07E-01 | 1.000 | locomotion |
| GO:0046034 | PROCESS | 89 | 3 | 6.08E-01 | 1.000 | ATP metabolic process |
| GO:0010629 | PROCESS | 420 | 11 | 6.08E-01 | 1.000 | negative regulation of gene expression |
| GO:0010740 | PROCESS | 145 | 3 | 6.10E-01 | 1.000 | positive regulation of protein kinase cascade |
| GO:0045944 | PROCESS | 273 | 8 | 6.11E-01 | 1.000 | positive regulation of transcription from RNA polymerase II promoter |
| GO:0032403 | FUNCTION | 126 | 4 | 6.12E-01 | 1.000 | protein complex binding |
| GO:0007346 | PROCESS | 138 | 3 | 6.12E-01 | 1.000 | regulation of mitotic cell cycle |
| GO:0008146 | FUNCTION | 45 | 2 | 6.12E-01 | 1.000 | sulfotransferase activity |
| GO:0006325 | PROCESS | 332 | 7 | 6.14E-01 | 1.000 | chromatin organization |
| GO:0009890 | PROCESS | 465 | 12 | 6.16E-01 | 1.000 | negative regulation of biosynthetic process |
| GO:0005089 | FUNCTION | 66 | 4 | 6.17E-01 | 1.000 | Rho guanyl-nucleotide exchange factor activity |
| GO:0045893 | PROCESS | 378 | 11 | 6.20E-01 | 1.000 | positive regulation of transcription, DNA-dependent |
| GO:0031981 | CELLULAR | 1297 | 26 | 6.21E-01 | 1.000 | nuclear lumen |
| GO:0010035 | PROCESS | 72 | 3 | 6.22E-01 | 1.000 | response to inorganic substance |
| GO:0031326 | PROCESS | 2641 | 61 | 6.24E-01 | 1.000 | regulation of cellular biosynthetic process |
| GO:0050863 | PROCESS | 84 | 2 | 6.25E-01 | 1.000 | regulation of T cell activation |
| GO:0050770 | PROCESS | 36 | 2 | 6.26E-01 | 1.000 | regulation of axonogenesis |
| GO:0051254 | PROCESS | 381 | 11 | 6.28E-01 | 1.000 | positive regulation of RNA metabolic process |
| GO:0001883 | FUNCTION | 1469 | 47 | 6.28E-01 | 1.000 | purine nucleoside binding |
| GO:0006928 | PROCESS | 361 | 15 | 6.29E-01 | 1.000 | cell motion |
| GO:0051674 | PROCESS | 361 | 15 | 6.29E-01 | 1.000 | localization of cell |
| GO:0010627 | PROCESS | 225 | 5 | 6.29E-01 | 1.000 | regulation of protein kinase cascade |
| GO:0005245 | FUNCTION | 24 | 2 | 6.30E-01 | 1.000 | voltage-gated calcium channel activity |
| GO:0048705 | PROCESS | 74 | 2 | 6.30E-01 | 1.000 | skeletal system morphogenesis |
| GO:0051173 | PROCESS | 495 | 14 | 6.31E-01 | 1.000 | positive regulation of nitrogen compound metabolic process |
| GO:0008654 | PROCESS | 91 | 2 | 6.31E-01 | 1.000 | phospholipid biosynthetic process |
| GO:0043168 | FUNCTION | 78 | 3 | 6.32E-01 | 1.000 | anion binding |
| GO:0005524 | FUNCTION | 1366 | 44 | 6.34E-01 | 1.000 | ATP binding |
| GO:0008360 | PROCESS | 48 | 2 | 6.34E-01 | 1.000 | regulation of cell shape |
| GO:0009889 | PROCESS | 2649 | 61 | 6.34E-01 | 1.000 | regulation of biosynthetic process |
| GO:0051098 | PROCESS | 118 | 3 | 6.35E-01 | 1.000 | regulation of binding |
| GO:0032147 | PROCESS | 83 | 3 | 6.35E-01 | 1.000 | activation of protein kinase activity |
| GO:0007411 | PROCESS | 71 | 5 | 6.36E-01 | 1.000 | axon guidance |
| GO:0006470 | PROCESS | 118 | 5 | 6.36E-01 | 1.000 | protein amino acid dephosphorylation |
| GO:0032270 | PROCESS | 158 | 4 | 6.37E-01 | 1.000 | positive regulation of cellular protein metabolic process |
| GO:0007219 | PROCESS | 43 | 2 | 6.39E-01 | 1.000 | Notch signaling pathway |
| GO:0050865 | PROCESS | 125 | 3 | 6.40E-01 | 1.000 | regulation of cell activation |
| GO:0016782 | FUNCTION | 54 | 2 | 6.40E-01 | 1.000 | transferase activity, transferring sulfur-containing groups |
| GO:0031252 | CELLULAR | 108 | 4 | 6.41E-01 | 1.000 | cell leading edge |
| GO:0000226 | PROCESS | 108 | 2 | 6.41E-01 | 1.000 | microtubule cytoskeleton organization |
| GO:0043687 | PROCESS | 1043 | 33 | 6.41E-01 | 1.000 | post-translational protein modification |
| GO:0051188 | PROCESS | 88 | 2 | 6.41E-01 | 1.000 | cofactor biosynthetic process |
| GO:0051052 | PROCESS | 101 | 2 | 6.41E-01 | 1.000 | regulation of DNA metabolic process |
| GO:0016607 | CELLULAR | 95 | 2 | 6.42E-01 | 1.000 | nuclear speck |
| GO:0015171 | FUNCTION | 52 | 2 | 6.42E-01 | 1.000 | amino acid transmembrane transporter activity |
| GO:0009967 | PROCESS | 241 | 6 | 6.43E-01 | 1.000 | positive regulation of signal transduction |
| GO:0007423 | PROCESS | 159 | 5 | 6.45E-01 | 1.000 | sensory organ development |
| GO:0051271 | PROCESS | 54 | 2 | 6.46E-01 | 1.000 | negative regulation of cell motion |

| | | | | | | |
|---|---|---|---|---|---|---|
| GO:0045449 | PROCESS | 2339 | 52 | 6.47E-01 | 1.000 | regulation of transcription |
| GO:0001882 | FUNCTION | 1478 | 47 | 6.47E-01 | 1.000 | nucleoside binding |
| GO:0009416 | PROCESS | 108 | 3 | 6.47E-01 | 1.000 | response to light stimulus |
| GO:0005911 | CELLULAR | 156 | 6 | 6.47E-01 | 1.000 | cell-cell junction |
| GO:0031325 | PROCESS | 658 | 18 | 6.49E-01 | 1.000 | positive regulation of cellular metabolic process |
| GO:0009308 | PROCESS | 351 | 9 | 6.52E-01 | 1.000 | amine metabolic process |
| GO:0006399 | PROCESS | 114 | 2 | 6.52E-01 | 1.000 | tRNA metabolic process |
| GO:0005509 | FUNCTION | 865 | 38 | 6.53E-01 | 1.000 | calcium ion binding |
| GO:0055067 | PROCESS | 32 | 2 | 6.53E-01 | 1.000 | monovalent inorganic cation homeostasis |
| GO:0048015 | PROCESS | 75 | 2 | 6.57E-01 | 1.000 | phosphoinositide-mediated signaling |
| GO:0009152 | PROCESS | 105 | 3 | 6.58E-01 | 1.000 | purine ribonucleotide biosynthetic process |
| GO:0015662 | FUNCTION | 44 | 2 | 6.59E-01 | 1.000 | ATPase activity, coupled to transmembrane movement of ions, phosphorylative mechanism |
| GO:0043009 | PROCESS | 229 | 6 | 6.59E-01 | 1.000 | chordate embryonic development |
| GO:0046906 | FUNCTION | 119 | 3 | 6.61E-01 | 1.000 | tetrapyrrole binding |
| GO:0030135 | CELLULAR | 126 | 4 | 6.62E-01 | 1.000 | coated vesicle |
| GO:0006633 | PROCESS | 74 | 2 | 6.62E-01 | 1.000 | fatty acid biosynthetic process |
| GO:0030029 | PROCESS | 204 | 7 | 6.63E-01 | 1.000 | actin filament-based process |
| GO:0016616 | FUNCTION | 96 | 2 | 6.64E-01 | 1.000 | oxidoreductase activity, acting on the CH-OH group of donors, NAD or NADP as acceptor |
| GO:0031974 | CELLULAR | 1668 | 33 | 6.64E-01 | 1.000 | membrane-enclosed lumen |
| GO:0030016 | CELLULAR | 91 | 3 | 6.65E-01 | 1.000 | myofibril |
| GO:0019752 | PROCESS | 497 | 12 | 6.65E-01 | 1.000 | carboxylic acid metabolic process |
| GO:0043436 | PROCESS | 497 | 12 | 6.65E-01 | 1.000 | oxoacid metabolic process |
| GO:0009792 | PROCESS | 231 | 6 | 6.66E-01 | 1.000 | embryonic development ending in birth or egg hatching |
| GO:0008544 | PROCESS | 153 | 3 | 6.66E-01 | 1.000 | epidermis development |
| GO:0043005 | CELLULAR | 171 | 9 | 6.67E-01 | 1.000 | neuron projection |
| GO:0051253 | PROCESS | 288 | 7 | 6.68E-01 | 1.000 | negative regulation of RNA metabolic process |
| GO:0006082 | PROCESS | 500 | 12 | 6.69E-01 | 1.000 | organic acid metabolic process |
| GO:0048469 | PROCESS | 53 | 2 | 6.69E-01 | 1.000 | cell maturation |
| GO:0048609 | PROCESS | 68 | 2 | 6.70E-01 | 1.000 | reproductive process in a multicellular organism |
| GO:0032504 | PROCESS | 68 | 2 | 6.70E-01 | 1.000 | multicellular organism reproduction |
| GO:0006665 | PROCESS | 67 | 2 | 6.72E-01 | 1.000 | sphingolipid metabolic process |
| GO:0009628 | PROCESS | 242 | 7 | 6.72E-01 | 1.000 | response to abiotic stimulus |
| GO:0034613 | PROCESS | 355 | 8 | 6.73E-01 | 1.000 | cellular protein localization |
| GO:0051259 | PROCESS | 106 | 3 | 6.73E-01 | 1.000 | protein oligomerization |
| GO:0005625 | CELLULAR | 199 | 5 | 6.75E-01 | 1.000 | soluble fraction |
| GO:0010604 | PROCESS | 646 | 18 | 6.75E-01 | 1.000 | positive regulation of macromolecule metabolic process |
| GO:0009101 | PROCESS | 144 | 5 | 6.77E-01 | 1.000 | glycoprotein biosynthetic process |
| GO:0031090 | CELLULAR | 931 | 23 | 6.78E-01 | 1.000 | organelle membrane |
| GO:0005856 | CELLULAR | 1187 | 37 | 6.79E-01 | 1.000 | cytoskeleton |
| GO:0043408 | PROCESS | 83 | 2 | 6.80E-01 | 1.000 | regulation of MAPKKK cascade |
| GO:0070161 | CELLULAR | 149 | 7 | 6.83E-01 | 1.000 | anchoring junction |
| GO:0042180 | PROCESS | 507 | 12 | 6.86E-01 | 1.000 | cellular ketone metabolic process |
| GO:0022613 | PROCESS | 164 | 2 | 6.86E-01 | 1.000 | ribonucleoprotein complex biogenesis |
| GO:0005794 | CELLULAR | 774 | 24 | 6.88E-01 | 1.000 | Golgi apparatus |
| GO:0070727 | PROCESS | 358 | 8 | 6.91E-01 | 1.000 | cellular macromolecule localization |
| GO:0043292 | CELLULAR | 99 | 3 | 6.92E-01 | 1.000 | contractile fiber |
| GO:0009968 | PROCESS | 195 | 5 | 6.94E-01 | 1.000 | negative regulation of signal transduction |
| GO:0005829 | CELLULAR | 1075 | 25 | 6.95E-01 | 1.000 | cytosol |
| GO:0051249 | PROCESS | 102 | 2 | 6.95E-01 | 1.000 | regulation of lymphocyte activation |
| GO:0009617 | PROCESS | 127 | 2 | 6.95E-01 | 1.000 | response to bacterium |
| GO:0031347 | PROCESS | 94 | 2 | 6.96E-01 | 1.000 | regulation of defense response |
| GO:0035108 | PROCESS | 63 | 2 | 6.99E-01 | 1.000 | limb morphogenesis |
| GO:0035107 | PROCESS | 63 | 2 | 6.99E-01 | 1.000 | appendage morphogenesis |
| GO:0043233 | CELLULAR | 1637 | 32 | 7.01E-01 | 1.000 | organelle lumen |
| GO:0050878 | PROCESS | 109 | 3 | 7.01E-01 | 1.000 | regulation of body fluid levels |
| GO:0043269 | PROCESS | 67 | 2 | 7.03E-01 | 1.000 | regulation of ion transport |
| GO:0016311 | PROCESS | 133 | 5 | 7.03E-01 | 1.000 | dephosphorylation |
| GO:0007169 | PROCESS | 186 | 7 | 7.04E-01 | 1.000 | transmembrane receptor protein tyrosine kinase signaling pathway |
| GO:0048812 | PROCESS | 131 | 7 | 7.04E-01 | 1.000 | neuron projection morphogenesis |
| GO:0001775 | PROCESS | 201 | 5 | 7.05E-01 | 1.000 | cell activation |
| GO:0009123 | PROCESS | 55 | 2 | 7.06E-01 | 1.000 | nucleoside monophosphate metabolic process |
| GO:0007178 | PROCESS | 83 | 2 | 7.06E-01 | 1.000 | transmembrane receptor protein serine/threonine kinase signaling pathway |
| GO:0005099 | FUNCTION | 80 | 3 | 7.06E-01 | 1.000 | Ras GTPase activator activity |
| GO:0006643 | PROCESS | 73 | 2 | 7.07E-01 | 1.000 | membrane lipid metabolic process |
| GO:0022414 | PROCESS | 547 | 12 | 7.07E-01 | 1.000 | reproductive process |
| GO:0050662 | FUNCTION | 154 | 4 | 7.08E-01 | 1.000 | coenzyme binding |
| GO:0051726 | PROCESS | 270 | 5 | 7.09E-01 | 1.000 | regulation of cell cycle |
| GO:0010817 | PROCESS | 111 | 3 | 7.10E-01 | 1.000 | regulation of hormone levels |
| GO:0000003 | PROCESS | 551 | 12 | 7.10E-01 | 1.000 | reproduction |
| GO:0016614 | FUNCTION | 108 | 2 | 7.13E-01 | 1.000 | oxidoreductase activity, acting on CH-OH group of donors |
| GO:0005923 | CELLULAR | 65 | 2 | 7.14E-01 | 1.000 | tight junction |
| GO:0070160 | CELLULAR | 65 | 2 | 7.14E-01 | 1.000 | occluding junction |
| GO:0009100 | PROCESS | 181 | 6 | 7.15E-01 | 1.000 | glycoprotein metabolic process |
| GO:0060173 | PROCESS | 65 | 2 | 7.15E-01 | 1.000 | limb development |
| GO:0048736 | PROCESS | 65 | 2 | 7.15E-01 | 1.000 | appendage development |
| GO:0007398 | PROCESS | 167 | 3 | 7.15E-01 | 1.000 | ectoderm development |

| | | | | | | |
|---|---|---|---|---|---|---|
| GO:0004930 | FUNCTION | 743 | 14 | 7.15E-01 | 1.000 | G-protein coupled receptor activity |
| GO:0005088 | FUNCTION | 78 | 4 | 7.16E-01 | 1.000 | Ras guanyl-nucleotide exchange factor activity |
| GO:0048667 | PROCESS | 133 | 7 | 7.17E-01 | 1.000 | cell morphogenesis involved in neuron differentiation |
| GO:0005200 | FUNCTION | 71 | 2 | 7.18E-01 | 1.000 | structural constituent of cytoskeleton |
| GO:0042625 | FUNCTION | 61 | 2 | 7.20E-01 | 1.000 | ATPase activity, coupled to transmembrane movement of ions |
| GO:0050658 | PROCESS | 84 | 2 | 7.20E-01 | 1.000 | RNA transport |
| GO:0050657 | PROCESS | 84 | 2 | 7.20E-01 | 1.000 | nucleic acid transport |
| GO:0051236 | PROCESS | 84 | 2 | 7.20E-01 | 1.000 | establishment of RNA localization |
| GO:0051276 | PROCESS | 423 | 8 | 7.21E-01 | 1.000 | chromosome organization |
| GO:0034660 | PROCESS | 217 | 3 | 7.21E-01 | 1.000 | ncRNA metabolic process |
| GO:0045597 | PROCESS | 169 | 5 | 7.21E-01 | 1.000 | positive regulation of cell differentiation |
| GO:0044432 | CELLULAR | 302 | 7 | 7.21E-01 | 1.000 | endoplasmic reticulum part |
| GO:0003924 | FUNCTION | 181 | 3 | 7.22E-01 | 1.000 | GTPase activity |
| GO:0019199 | FUNCTION | 80 | 4 | 7.23E-01 | 1.000 | transmembrane receptor protein kinase activity |
| GO:0014069 | CELLULAR | 43 | 3 | 7.23E-01 | 1.000 | postsynaptic density |
| GO:0004672 | FUNCTION | 559 | 19 | 7.26E-01 | 1.000 | protein kinase activity |
| GO:0010648 | PROCESS | 204 | 5 | 7.27E-01 | 1.000 | negative regulation of cell communication |
| GO:0009117 | PROCESS | 247 | 7 | 7.27E-01 | 1.000 | nucleotide metabolic process |
| GO:0006753 | PROCESS | 247 | 7 | 7.27E-01 | 1.000 | nucleoside phosphate metabolic process |
| GO:0048858 | PROCESS | 138 | 7 | 7.29E-01 | 1.000 | cell projection morphogenesis |
| GO:0042110 | PROCESS | 80 | 2 | 7.30E-01 | 1.000 | T cell activation |
| GO:0044267 | PROCESS | 2072 | 51 | 7.32E-01 | 1.000 | cellular protein metabolic process |
| GO:0016874 | FUNCTION | 355 | 8 | 7.32E-01 | 1.000 | ligase activity |
| GO:0019887 | FUNCTION | 73 | 2 | 7.36E-01 | 1.000 | protein kinase regulator activity |
| GO:0006457 | PROCESS | 153 | 2 | 7.36E-01 | 1.000 | protein folding |
| GO:0045934 | PROCESS | 420 | 10 | 7.38E-01 | 1.000 | negative regulation of nucleobase, nucleoside, nucleotide and nucleic acid metabolic process |
| GO:0007608 | PROCESS | 363 | 4 | 7.38E-01 | 1.000 | sensory perception of smell |
| GO:0048523 | PROCESS | 1309 | 33 | 7.38E-01 | 1.000 | negative regulation of cellular process |
| GO:0004721 | FUNCTION | 150 | 5 | 7.40E-01 | 1.000 | phosphoprotein phosphatase activity |
| GO:0051172 | PROCESS | 421 | 10 | 7.41E-01 | 1.000 | negative regulation of nitrogen compound metabolic process |
| GO:0009260 | PROCESS | 112 | 3 | 7.41E-01 | 1.000 | ribonucleotide biosynthetic process |
| GO:0016070 | PROCESS | 830 | 15 | 7.42E-01 | 1.000 | RNA metabolic process |
| GO:0031225 | CELLULAR | 208 | 8 | 7.42E-01 | 1.000 | anchored to membrane |
| GO:0021700 | PROCESS | 66 | 2 | 7.43E-01 | 1.000 | developmental maturation |
| GO:0004497 | FUNCTION | 88 | 2 | 7.44E-01 | 1.000 | monooxygenase activity |
| GO:0046942 | PROCESS | 115 | 3 | 7.45E-01 | 1.000 | carboxylic acid transport |
| GO:0032990 | PROCESS | 145 | 7 | 7.45E-01 | 1.000 | cell part morphogenesis |
| GO:0010608 | PROCESS | 184 | 3 | 7.45E-01 | 1.000 | posttranscriptional regulation of gene expression |
| GO:0015849 | PROCESS | 116 | 3 | 7.45E-01 | 1.000 | organic acid transport |
| GO:0045596 | PROCESS | 157 | 4 | 7.46E-01 | 1.000 | negative regulation of cell differentiation |
| GO:0007267 | PROCESS | 497 | 16 | 7.46E-01 | 1.000 | cell-cell signaling |
| GO:0048513 | PROCESS | 1319 | 40 | 7.48E-01 | 1.000 | organ development |
| GO:0051493 | PROCESS | 101 | 2 | 7.51E-01 | 1.000 | regulation of cytoskeleton organization |
| GO:0005901 | CELLULAR | 30 | 2 | 7.51E-01 | 1.000 | caveola |
| GO:0006403 | PROCESS | 86 | 2 | 7.52E-01 | 1.000 | RNA localization |
| GO:0030674 | FUNCTION | 84 | 2 | 7.54E-01 | 1.000 | protein binding, bridging |
| GO:0044249 | PROCESS | 1587 | 36 | 7.54E-01 | 1.000 | cellular biosynthetic process |
| GO:0040008 | PROCESS | 244 | 5 | 7.55E-01 | 1.000 | regulation of growth |
| GO:0007167 | PROCESS | 282 | 10 | 7.55E-01 | 1.000 | enzyme linked receptor protein signaling pathway |
| GO:0090066 | PROCESS | 222 | 5 | 7.55E-01 | 1.000 | regulation of anatomical structure size |
| GO:0044451 | CELLULAR | 470 | 9 | 7.56E-01 | 1.000 | nucleoplasm part |
| GO:0006351 | PROCESS | 226 | 4 | 7.57E-01 | 1.000 | transcription, DNA-dependent |
| GO:0045664 | PROCESS | 85 | 3 | 7.57E-01 | 1.000 | regulation of neuron differentiation |
| GO:0010556 | PROCESS | 2541 | 54 | 7.58E-01 | 1.000 | regulation of macromolecule biosynthetic process |
| GO:0005730 | CELLULAR | 628 | 11 | 7.59E-01 | 1.000 | nucleolus |
| GO:0016740 | FUNCTION | 1568 | 44 | 7.62E-01 | 1.000 | transferase activity |
| GO:0009887 | PROCESS | 521 | 15 | 7.63E-01 | 1.000 | organ morphogenesis |
| GO:0070013 | CELLULAR | 1601 | 30 | 7.66E-01 | 1.000 | intracellular organelle lumen |
| GO:0016564 | FUNCTION | 261 | 6 | 7.66E-01 | 1.000 | transcription repressor activity |
| GO:0016337 | PROCESS | 256 | 11 | 7.67E-01 | 1.000 | cell-cell adhesion |
| GO:0010628 | PROCESS | 455 | 12 | 7.67E-01 | 1.000 | positive regulation of gene expression |
| GO:0005085 | FUNCTION | 140 | 6 | 7.68E-01 | 1.000 | guanyl-nucleotide exchange factor activity |
| GO:0032774 | PROCESS | 230 | 4 | 7.70E-01 | 1.000 | RNA biosynthetic process |
| GO:0000902 | PROCESS | 218 | 9 | 7.71E-01 | 1.000 | cell morphogenesis |
| GO:0044270 | PROCESS | 85 | 2 | 7.72E-01 | 1.000 | nitrogen compound catabolic process |
| GO:0005001 | FUNCTION | 18 | 2 | 7.72E-01 | 1.000 | transmembrane receptor protein tyrosine phosphatase activity |
| GO:0019198 | FUNCTION | 18 | 2 | 7.72E-01 | 1.000 | transmembrane receptor protein phosphatase activity |
| GO:0044087 | PROCESS | 108 | 2 | 7.73E-01 | 1.000 | regulation of cellular component biogenesis |
| GO:0030031 | PROCESS | 60 | 2 | 7.73E-01 | 1.000 | cell projection assembly |
| GO:0044237 | PROCESS | 4589 | 106 | 7.73E-01 | 1.000 | cellular metabolic process |
| GO:0006754 | PROCESS | 78 | 2 | 7.74E-01 | 1.000 | ATP biosynthetic process |
| GO:0008283 | PROCESS | 302 | 7 | 7.74E-01 | 1.000 | cell proliferation |
| GO:0045941 | PROCESS | 440 | 11 | 7.75E-01 | 1.000 | positive regulation of transcription |
| GO:0055086 | PROCESS | 267 | 7 | 7.75E-01 | 1.000 | nucleobase, nucleoside and nucleotide metabolic process |
| GO:0006412 | PROCESS | 273 | 3 | 7.76E-01 | 1.000 | translation |
| GO:0019207 | FUNCTION | 83 | 2 | 7.77E-01 | 1.000 | kinase regulator activity |
| GO:0005813 | CELLULAR | 191 | 4 | 7.77E-01 | 1.000 | centrosome |
| GO:0003712 | FUNCTION | 325 | 7 | 7.77E-01 | 1.000 | transcription cofactor activity |

| | | | | | | |
|---|---|---|---|---|---|---|
| GO:0004674 | FUNCTION | 395 | 12 | 7.80E-01 | 1.000 | protein serine/threonine kinase activity |
| GO:0032870 | PROCESS | 96 | 2 | 7.80E-01 | 1.000 | cellular response to hormone stimulus |
| GO:0043062 | PROCESS | 104 | 3 | 7.81E-01 | 1.000 | extracellular structure organization |
| GO:0007409 | PROCESS | 123 | 6 | 7.81E-01 | 1.000 | axonogenesis |
| GO:0044456 | CELLULAR | 182 | 10 | 7.82E-01 | 1.000 | synapse part |
| GO:0033043 | PROCESS | 179 | 3 | 7.84E-01 | 1.000 | regulation of organelle organization |
| GO:0005789 | CELLULAR | 228 | 5 | 7.86E-01 | 1.000 | endoplasmic reticulum membrane |
| GO:0051707 | PROCESS | 219 | 3 | 7.89E-01 | 1.000 | response to other organism |
| GO:0008083 | FUNCTION | 151 | 3 | 7.90E-01 | 1.000 | growth factor activity |
| GO:0045892 | PROCESS | 283 | 6 | 7.91E-01 | 1.000 | negative regulation of transcription, DNA-dependent |
| GO:0008037 | PROCESS | 47 | 3 | 7.91E-01 | 1.000 | cell recognition |
| GO:0044446 | CELLULAR | 3686 | 79 | 7.95E-01 | 1.000 | intracellular organelle part |
| GO:0016323 | CELLULAR | 150 | 5 | 7.95E-01 | 1.000 | basolateral plasma membrane |
| GO:0031404 | FUNCTION | 66 | 2 | 7.96E-01 | 1.000 | chloride ion binding |
| GO:0005654 | CELLULAR | 781 | 14 | 7.98E-01 | 1.000 | nucleoplasm |
| GO:0006366 | PROCESS | 176 | 3 | 8.01E-01 | 1.000 | transcription from RNA polymerase II promoter |
| GO:0045296 | FUNCTION | 13 | 2 | 8.02E-01 | 1.000 | cadherin binding |
| GO:0030027 | CELLULAR | 57 | 2 | 8.02E-01 | 1.000 | lamellipodium |
| GO:0006520 | PROCESS | 213 | 4 | 8.02E-01 | 1.000 | cellular amino acid metabolic process |
| GO:0044106 | PROCESS | 213 | 4 | 8.02E-01 | 1.000 | cellular amine metabolic process |
| GO:0001817 | PROCESS | 130 | 2 | 8.03E-01 | 1.000 | regulation of cytokine production |
| GO:0044428 | CELLULAR | 1605 | 30 | 8.04E-01 | 1.000 | nuclear part |
| GO:0007606 | PROCESS | 405 | 4 | 8.06E-01 | 1.000 | sensory perception of chemical stimulus |
| GO:0009058 | PROCESS | 1657 | 37 | 8.07E-01 | 1.000 | biosynthetic process |
| GO:0015931 | PROCESS | 99 | 2 | 8.07E-01 | 1.000 | nucleobase, nucleoside, nucleotide and nucleic acid transport |
| GO:0030534 | PROCESS | 59 | 2 | 8.09E-01 | 1.000 | adult behavior |
| GO:0042995 | CELLULAR | 464 | 18 | 8.11E-01 | 1.000 | cell projection |
| GO:0012505 | CELLULAR | 680 | 18 | 8.12E-01 | 1.000 | endomembrane system |
| GO:0003777 | FUNCTION | 72 | 2 | 8.12E-01 | 1.000 | microtubule motor activity |
| GO:0005254 | FUNCTION | 63 | 2 | 8.13E-01 | 1.000 | chloride channel activity |
| GO:0030900 | PROCESS | 88 | 2 | 8.14E-01 | 1.000 | forebrain development |
| GO:0005976 | PROCESS | 97 | 2 | 8.14E-01 | 1.000 | polysaccharide metabolic process |
| GO:0020037 | FUNCTION | 111 | 2 | 8.14E-01 | 1.000 | heme binding |
| GO:0005634 | CELLULAR | 4571 | 94 | 8.15E-01 | 1.000 | nucleus |
| GO:0005253 | FUNCTION | 67 | 2 | 8.21E-01 | 1.000 | anion channel activity |
| GO:0007276 | PROCESS | 286 | 5 | 8.21E-01 | 1.000 | gamete generation |
| GO:0048519 | PROCESS | 1431 | 36 | 8.23E-01 | 1.000 | negative regulation of biological process |
| GO:0007017 | PROCESS | 207 | 4 | 8.23E-01 | 1.000 | microtubule-based process |
| GO:0051050 | PROCESS | 159 | 3 | 8.24E-01 | 1.000 | positive regulation of transport |
| GO:0006486 | PROCESS | 119 | 3 | 8.24E-01 | 1.000 | protein amino acid glycosylation |
| GO:0043413 | PROCESS | 119 | 3 | 8.24E-01 | 1.000 | biopolymer glycosylation |
| GO:0070085 | PROCESS | 119 | 3 | 8.24E-01 | 1.000 | glycosylation |
| GO:0006913 | PROCESS | 132 | 2 | 8.24E-01 | 1.000 | nucleocytoplasmic transport |
| GO:0005694 | CELLULAR | 386 | 5 | 8.25E-01 | 1.000 | chromosome |
| GO:0051240 | PROCESS | 147 | 3 | 8.26E-01 | 1.000 | positive regulation of multicellular organismal process |
| GO:0051169 | PROCESS | 134 | 2 | 8.28E-01 | 1.000 | nuclear transport |
| GO:0043405 | PROCESS | 115 | 2 | 8.29E-01 | 1.000 | regulation of MAP kinase activity |
| GO:0032989 | PROCESS | 246 | 9 | 8.29E-01 | 1.000 | cellular component morphogenesis |
| GO:0010557 | PROCESS | 513 | 12 | 8.29E-01 | 1.000 | positive regulation of macromolecule biosynthetic process |
| GO:0055114 | PROCESS | 570 | 12 | 8.30E-01 | 1.000 | oxidation reduction |
| GO:0002694 | PROCESS | 119 | 2 | 8.30E-01 | 1.000 | regulation of leukocyte activation |
| GO:0016301 | FUNCTION | 717 | 22 | 8.31E-01 | 1.000 | kinase activity |
| GO:0050767 | PROCESS | 110 | 3 | 8.31E-01 | 1.000 | regulation of neurogenesis |
| GO:0006807 | PROCESS | 1885 | 39 | 8.33E-01 | 1.000 | nitrogen compound metabolic process |
| GO:0006732 | PROCESS | 132 | 2 | 8.35E-01 | 1.000 | coenzyme metabolic process |
| GO:0042175 | CELLULAR | 241 | 5 | 8.35E-01 | 1.000 | nuclear envelope-endoplasmic reticulum network |
| GO:0044422 | CELLULAR | 3708 | 79 | 8.37E-01 | 1.000 | organelle part |
| GO:0003714 | FUNCTION | 124 | 2 | 8.37E-01 | 1.000 | transcription corepressor activity |
| GO:0044260 | PROCESS | 3304 | 73 | 8.38E-01 | 1.000 | cellular macromolecule metabolic process |
| GO:0044449 | CELLULAR | 91 | 2 | 8.38E-01 | 1.000 | contractile fiber part |
| GO:0005201 | FUNCTION | 76 | 2 | 8.40E-01 | 1.000 | extracellular matrix structural constituent |
| GO:0009607 | PROCESS | 304 | 4 | 8.41E-01 | 1.000 | response to biotic stimulus |
| GO:0044092 | PROCESS | 277 | 5 | 8.42E-01 | 1.000 | negative regulation of molecular function |
| GO:0016481 | PROCESS | 378 | 8 | 8.42E-01 | 1.000 | negative regulation of transcription |
| GO:0005815 | CELLULAR | 213 | 4 | 8.43E-01 | 1.000 | microtubule organizing center |
| GO:0000139 | CELLULAR | 172 | 4 | 8.43E-01 | 1.000 | Golgi membrane |
| GO:0030030 | PROCESS | 239 | 10 | 8.44E-01 | 1.000 | cell projection organization |
| GO:0031175 | PROCESS | 155 | 7 | 8.47E-01 | 1.000 | neuron projection development |
| GO:0005874 | CELLULAR | 240 | 5 | 8.48E-01 | 1.000 | microtubule |
| GO:0006605 | PROCESS | 180 | 3 | 8.49E-01 | 1.000 | protein targeting |
| GO:0070887 | PROCESS | 197 | 4 | 8.49E-01 | 1.000 | cellular response to chemical stimulus |
| GO:0006139 | PROCESS | 1548 | 30 | 8.51E-01 | 1.000 | nucleobase, nucleoside, nucleotide and nucleic acid metabolic process |
| GO:0032535 | PROCESS | 184 | 3 | 8.51E-01 | 1.000 | regulation of cellular component size |
| GO:0001666 | PROCESS | 72 | 2 | 8.52E-01 | 1.000 | response to hypoxia |
| GO:0006464 | PROCESS | 1290 | 36 | 8.52E-01 | 1.000 | protein modification process |
| GO:0043086 | PROCESS | 233 | 4 | 8.52E-01 | 1.000 | negative regulation of catalytic activity |
| GO:0006350 | PROCESS | 268 | 4 | 8.53E-01 | 1.000 | transcription |
| GO:0043412 | PROCESS | 1356 | 37 | 8.53E-01 | 1.000 | biopolymer modification |
| GO:0045761 | PROCESS | 79 | 2 | 8.53E-01 | 1.000 | regulation of adenylate cyclase activity |

| | | | | | | |
|---|---|---|---|---|---|---|
| GO:0034641 | PROCESS | 224 | 4 | 8.53E-01 | 1.000 | cellular nitrogen compound metabolic process |
| GO:0000122 | PROCESS | 205 | 4 | 8.54E-01 | 1.000 | negative regulation of transcription from RNA polymerase II promoter |
| GO:0060429 | PROCESS | 117 | 2 | 8.54E-01 | 1.000 | epithelium development |
| GO:0045860 | PROCESS | 172 | 4 | 8.54E-01 | 1.000 | positive regulation of protein kinase activity |
| GO:0016773 | FUNCTION | 667 | 20 | 8.54E-01 | 1.000 | phosphotransferase activity, alcohol group as acceptor |
| GO:0001501 | PROCESS | 243 | 5 | 8.55E-01 | 1.000 | skeletal system development |
| GO:0034960 | PROCESS | 3187 | 70 | 8.55E-01 | 1.000 | cellular biopolymer metabolic process |
| GO:0070482 | PROCESS | 74 | 2 | 8.55E-01 | 1.000 | response to oxygen levels |
| GO:0033674 | PROCESS | 173 | 4 | 8.56E-01 | 1.000 | positive regulation of kinase activity |
| GO:0046943 | FUNCTION | 80 | 2 | 8.58E-01 | 1.000 | carboxylic acid transmembrane transporter activity |
| GO:0005342 | FUNCTION | 81 | 2 | 8.59E-01 | 1.000 | organic acid transmembrane transporter activity |
| GO:0007610 | PROCESS | 349 | 9 | 8.59E-01 | 1.000 | behavior |
| GO:0031279 | PROCESS | 81 | 2 | 8.60E-01 | 1.000 | regulation of cyclase activity |
| GO:0005578 | CELLULAR | 290 | 10 | 8.61E-01 | 1.000 | proteinaceous extracellular matrix |
| GO:0048666 | PROCESS | 211 | 8 | 8.61E-01 | 1.000 | neuron development |
| GO:0051339 | PROCESS | 83 | 2 | 8.64E-01 | 1.000 | regulation of lyase activity |
| GO:0010558 | PROCESS | 450 | 9 | 8.64E-01 | 1.000 | negative regulation of macromolecule biosynthetic process |
| GO:0051960 | PROCESS | 125 | 3 | 8.66E-01 | 1.000 | regulation of nervous system development |
| GO:0017124 | FUNCTION | 83 | 2 | 8.71E-01 | 1.000 | SH3 domain binding |
| GO:0007018 | PROCESS | 98 | 2 | 8.71E-01 | 1.000 | microtubule-based movement |
| GO:0008066 | FUNCTION | 29 | 2 | 8.71E-01 | 1.000 | glutamate receptor activity |
| GO:0042626 | FUNCTION | 93 | 3 | 8.74E-01 | 1.000 | ATPase activity, coupled to transmembrane movement of substances |
| GO:0051347 | PROCESS | 180 | 4 | 8.75E-01 | 1.000 | positive regulation of transferase activity |
| GO:0044420 | CELLULAR | 97 | 3 | 8.77E-01 | 1.000 | extracellular matrix part |
| GO:0043492 | FUNCTION | 94 | 3 | 8.77E-01 | 1.000 | ATPase activity, coupled to movement of substances |
| GO:0044271 | PROCESS | 132 | 2 | 8.78E-01 | 1.000 | nitrogen compound biosynthetic process |
| GO:0016820 | FUNCTION | 95 | 3 | 8.78E-01 | 1.000 | hydrolase activity, acting on acid anhydrides, catalyzing transmembrane movement of substances |
| GO:0048037 | FUNCTION | 214 | 4 | 8.80E-01 | 1.000 | cofactor binding |
| GO:0048468 | PROCESS | 587 | 18 | 8.81E-01 | 1.000 | cell development |
| GO:0018193 | PROCESS | 122 | 2 | 8.82E-01 | 1.000 | peptidyl-amino acid modification |
| GO:0016772 | FUNCTION | 833 | 23 | 8.85E-01 | 1.000 | transferase activity, transferring phosphorus-containing groups |
| GO:0043296 | CELLULAR | 89 | 2 | 8.85E-01 | 1.000 | apical junction complex |
| GO:0015405 | FUNCTION | 104 | 3 | 8.85E-01 | 1.000 | P-P-bond-hydrolysis-driven transmembrane transporter activity |
| GO:0015399 | FUNCTION | 104 | 3 | 8.85E-01 | 1.000 | primary active transmembrane transporter activity |
| GO:0004984 | FUNCTION | 346 | 2 | 8.86E-01 | 1.000 | olfactory receptor activity |
| GO:0003713 | FUNCTION | 196 | 3 | 8.89E-01 | 1.000 | transcription coactivator activity |
| GO:0005496 | FUNCTION | 112 | 2 | 8.90E-01 | 1.000 | steroid binding |
| GO:0007275 | PROCESS | 2322 | 69 | 8.92E-01 | 1.000 | multicellular organismal development |
| GO:0031012 | CELLULAR | 308 | 10 | 8.94E-01 | 1.000 | extracellular matrix |
| GO:0048699 | PROCESS | 367 | 12 | 8.96E-01 | 1.000 | generation of neurons |
| GO:0009653 | PROCESS | 843 | 24 | 8.98E-01 | 1.000 | anatomical structure morphogenesis |
| GO:0043284 | PROCESS | 741 | 11 | 9.01E-01 | 1.000 | biopolymer biosynthetic process |
| GO:0019953 | PROCESS | 343 | 5 | 9.03E-01 | 1.000 | sexual reproduction |
| GO:0016327 | CELLULAR | 92 | 2 | 9.05E-01 | 1.000 | apicolateral plasma membrane |
| GO:0009059 | PROCESS | 930 | 16 | 9.05E-01 | 1.000 | macromolecule biosynthetic process |
| GO:0008285 | PROCESS | 288 | 6 | 9.07E-01 | 1.000 | negative regulation of cell proliferation |
| GO:0060284 | PROCESS | 138 | 3 | 9.08E-01 | 1.000 | regulation of cell development |
| GO:0044427 | CELLULAR | 314 | 3 | 9.08E-01 | 1.000 | chromosomal part |
| GO:0001558 | PROCESS | 134 | 2 | 9.10E-01 | 1.000 | regulation of cell growth |
| GO:0016563 | FUNCTION | 341 | 6 | 9.10E-01 | 1.000 | transcription activator activity |
| GO:0009719 | PROCESS | 200 | 4 | 9.10E-01 | 1.000 | response to endogenous stimulus |
| GO:0009725 | PROCESS | 186 | 3 | 9.10E-01 | 1.000 | response to hormone stimulus |
| GO:0045859 | PROCESS | 278 | 5 | 9.12E-01 | 1.000 | regulation of protein kinase activity |
| GO:0043232 | CELLULAR | 2236 | 50 | 9.13E-01 | 1.000 | intracellular non-membrane-bounded organelle |
| GO:0043228 | CELLULAR | 2236 | 50 | 9.13E-01 | 1.000 | non-membrane-bounded organelle |
| GO:0000165 | PROCESS | 142 | 2 | 9.13E-01 | 1.000 | MAPKKK cascade |
| GO:0043549 | PROCESS | 283 | 5 | 9.14E-01 | 1.000 | regulation of kinase activity |
| GO:0019900 | FUNCTION | 124 | 2 | 9.15E-01 | 1.000 | kinase binding |
| GO:0016491 | FUNCTION | 624 | 12 | 9.15E-01 | 1.000 | oxidoreductase activity |
| GO:0007507 | PROCESS | 158 | 3 | 9.16E-01 | 1.000 | heart development |
| GO:0009055 | FUNCTION | 198 | 3 | 9.16E-01 | 1.000 | electron carrier activity |
| GO:0006468 | PROCESS | 588 | 16 | 9.20E-01 | 1.000 | protein amino acid phosphorylation |
| GO:0030154 | PROCESS | 1245 | 35 | 9.20E-01 | 1.000 | cell differentiation |
| GO:0034645 | PROCESS | 912 | 15 | 9.22E-01 | 1.000 | cellular macromolecule biosynthetic process |
| GO:0022008 | PROCESS | 392 | 12 | 9.24E-01 | 1.000 | neurogenesis |
| GO:0009888 | PROCESS | 537 | 10 | 9.25E-01 | 1.000 | tissue development |
| GO:0008134 | FUNCTION | 450 | 8 | 9.27E-01 | 1.000 | transcription factor binding |
| GO:0048729 | PROCESS | 134 | 2 | 9.28E-01 | 1.000 | tissue morphogenesis |
| GO:0006796 | PROCESS | 848 | 23 | 9.29E-01 | 1.000 | phosphate metabolic process |
| GO:0006793 | PROCESS | 848 | 23 | 9.29E-01 | 1.000 | phosphorus metabolic process |
| GO:0004714 | FUNCTION | 64 | 2 | 9.29E-01 | 1.000 | transmembrane receptor protein tyrosine kinase activity |
| GO:0051338 | PROCESS | 294 | 5 | 9.30E-01 | 1.000 | regulation of transferase activity |
| GO:0009898 | CELLULAR | 292 | 6 | 9.31E-01 | 1.000 | internal side of plasma membrane |
| GO:0005759 | CELLULAR | 210 | 2 | 9.32E-01 | 1.000 | mitochondrial matrix |
| GO:0031980 | CELLULAR | 210 | 2 | 9.32E-01 | 1.000 | mitochondrial lumen |
| GO:0034961 | PROCESS | 731 | 10 | 9.34E-01 | 1.000 | cellular biopolymer biosynthetic process |
| GO:0051130 | PROCESS | 137 | 2 | 9.36E-01 | 1.000 | positive regulation of cellular component organization |
| GO:0048471 | CELLULAR | 223 | 4 | 9.36E-01 | 1.000 | perinuclear region of cytoplasm |

| | | | | | | |
|---|---|---|---|---|---|---|
| GO:0030182 | PROCESS | 283 | 9 | 9.36E-01 | 1.000 | neuron differentiation |
| GO:0048869 | PROCESS | 1286 | 35 | 9.45E-01 | 1.000 | cellular developmental process |
| GO:0044463 | CELLULAR | 134 | 3 | 9.46E-01 | 1.000 | cell projection part |
| GO:0016310 | PROCESS | 696 | 17 | 9.47E-01 | 1.000 | phosphorylation |
| GO:0005635 | CELLULAR | 176 | 2 | 9.49E-01 | 1.000 | nuclear envelope |
| GO:0044431 | CELLULAR | 257 | 5 | 9.56E-01 | 1.000 | Golgi apparatus part |
| GO:0007626 | PROCESS | 216 | 4 | 9.57E-01 | 1.000 | locomotory behavior |
| GO:0032502 | PROCESS | 2538 | 71 | 9.59E-01 | 1.000 | developmental process |
| GO:0042325 | PROCESS | 373 | 6 | 9.59E-01 | 1.000 | regulation of phosphorylation |
| GO:0005506 | FUNCTION | 278 | 4 | 9.59E-01 | 1.000 | iron ion binding |
| GO:0005198 | FUNCTION | 555 | 9 | 9.60E-01 | 1.000 | structural molecule activity |
| GO:0048731 | PROCESS | 1804 | 51 | 9.67E-01 | 1.000 | system development |
| GO:0007156 | PROCESS | 131 | 3 | 9.69E-01 | 1.000 | homophilic cell adhesion |
| GO:0005975 | PROCESS | 455 | 7 | 9.70E-01 | 1.000 | carbohydrate metabolic process |
| GO:0007420 | PROCESS | 184 | 4 | 9.72E-01 | 1.000 | brain development |
| GO:0019220 | PROCESS | 387 | 6 | 9.73E-01 | 1.000 | regulation of phosphate metabolic process |
| GO:0051174 | PROCESS | 387 | 6 | 9.73E-01 | 1.000 | regulation of phosphorus metabolic process |
| GO:0007417 | PROCESS | 293 | 8 | 9.78E-01 | 1.000 | central nervous system development |
| GO:0048856 | PROCESS | 1940 | 53 | 9.80E-01 | 1.000 | anatomical structure development |
| GO:0007243 | PROCESS | 292 | 4 | 9.81E-01 | 1.000 | protein kinase cascade |
| GO:0044262 | PROCESS | 257 | 3 | 9.83E-01 | 1.000 | cellular carbohydrate metabolic process |
| GO:0019904 | FUNCTION | 253 | 4 | 9.86E-01 | 1.000 | protein domain specific binding |
| GO:0007399 | PROCESS | 802 | 21 | 9.98E-01 | 1.000 | nervous system development |
