# Supplementary material for: Genetic Evidence Implicates the Immune System and Cholesterol Metabolism in the Aetiology of Alzheimer's Disease
Source: PLoS One. 2010 Nov 15;5(11):e13950. doi: 10.1371/journal.pone.0013950 (PMC2981526; doi:10.1371/journal.pone.0013950)
Supplement: Table S2 — Gene ontology categories identified by ALIGATOR analysis of the AD GWA data of Lambert and colleagues. The 423 genes identified as having GWAS SNP signals p<0.001 from Lambert et al. (8)were used: APOE was included in the gene list. In this analysis one SNP was not allowed to add more than one gene to any gene ontology category. “Study-wide p-value” is the probability of obtaining by chance at least one GO category with a category-specific enrichment p-value at least as significant as that observed. (0.08 MB PDF) [file pone.0013950.s002.pdf]

Table S2 Gene ontology categories identified by ALIGATOR analysis of the AD GWA data of Lambert and colleagues (8)

The 423 genes identified as having GWAS SNP signals p < 0.001 were used: APOE was included in the gene list. In this analysis one SNP was not allowed to add more than one gene to any gene ontology category. “Study-wide p-value” is the probability of obtaining by chance at least one GO category with a category-specific enrichment p-value at least as significant as that observed.

GO process	Type	total genes in category	#genes on list	p-value	Study-wide corrected p-value	Function
GO:0032395	FUNCTION	8	4	0.0002	0.478	MHC class II receptor activity
GO:0004875	FUNCTION	3	2	0.0004	0.597	complement receptor activity
GO:0042613	CELLULAR	12	4	0.0006	0.675	MHC class II protein complex
GO:0002504	PROCESS	15	4	0.0008	0.758	antigen processing and presentation of peptide or polysaccharide antigen via MHC class II
GO:0030301	PROCESS	34	5	0.0012	0.839	cholesterol transport
GO:0015918	PROCESS	34	5	0.0012	0.839	sterol transport
GO:0031490	FUNCTION	7	2	0.0032	0.976	chromatin DNA binding
GO:0019276	PROCESS	3	2	0.0034	0.979	UDP-N-acetylgalactosamine metabolic process
GO:0005319	FUNCTION	57	7	0.0038	0.985	lipid transporter activity
GO:0042611	CELLULAR	34	4	0.0050	0.996	MHC protein complex
GO:0001848	FUNCTION	8	2	0.0066	0.999	complement binding
GO:0045540	PROCESS	7	2	0.0070	1	regulation of cholesterol biosynthetic process
GO:0005543	FUNCTION	153	10	0.0084	1	phospholipid binding
GO:0043691	PROCESS	14	3	0.0086	1	reverse cholesterol transport
GO:0007271	PROCESS	13	2	0.0090	1	synaptic transmission, cholinergic
GO:0042632	PROCESS	33	4	0.0092	1	cholesterol homeostasis
GO:0055092	PROCESS	33	4	0.0092	1	sterol homeostasis
GO:0033344	PROCESS	15	3	0.0108	1	cholesterol efflux
GO:0006958	PROCESS	28	3	0.0108	1	complement activation, classical pathway
GO:0002455	PROCESS	28	3	0.0108	1	humoral immune response mediated by circulating immunoglobulin
GO:0015248	FUNCTION	14	3	0.0112	1	sterol transporter activity
GO:0051775	PROCESS	6	2	0.0124	1	response to redox state
GO:0008289	FUNCTION	454	20	0.0158	1	lipid binding
GO:0055088	PROCESS	43	4	0.0170	1	lipid homeostasis
GO:0016525	PROCESS	20	3	0.0174	1	negative regulation of angiogenesis
GO:0032526	PROCESS	14	2	0.0180	1	response to retinoic acid
GO:0003705	FUNCTION	29	3	0.0186	1	RNA polymerase II transcription factor activity, enhancer binding
GO:0010872	PROCESS	7	2	0.0198	1	regulation of cholesterol esterification
GO:0033189	PROCESS	15	2	0.0198	1	response to vitamin A
GO:0006040	PROCESS	26	3	0.0202	1	amino sugar metabolic process
GO:0006955	PROCESS	570	16	0.0214	1	immune response
GO:0031228	CELLULAR	46	5	0.0220	1	intrinsic to Golgi membrane
GO:0030195	PROCESS	15	2	0.0220	1	negative regulation of blood coagulation
GO:0051393	FUNCTION	3	2	0.0222	1	alpha-actinin binding
GO:0019882	PROCESS	54	4	0.0224	1	antigen processing and presentation
GO:0006956	PROCESS	37	3	0.0226	1	complement activation
GO:0016592	CELLULAR	19	2	0.0226	1	mediator complex
GO:0002541	PROCESS	38	3	0.0228	1	activation of plasma proteins involved in acute inflammatory response
GO:0007010	PROCESS	354	15	0.0232	1	cytoskeleton organization
GO:0005587	CELLULAR	4	2	0.0248	1	collagen type IV
GO:0034377	PROCESS	11	2	0.0250	1	plasma lipoprotein particle assembly
GO:0065005	PROCESS	11	2	0.0250	1	protein-lipid complex assembly
GO:0004364	FUNCTION	19	2	0.0252	1	glutathione transferase activity
GO:0006997	PROCESS	40	3	0.0258	1	nucleus organization
GO:0006297	PROCESS	17	2	0.0258	1	nucleotide-excision repair, DNA gap filling
GO:0050819	PROCESS	17	2	0.0276	1	negative regulation of coagulation
GO:0033700	PROCESS	8	2	0.0278	1	phospholipid efflux
GO:0019748	PROCESS	65	4	0.0308	1	secondary metabolic process
GO:0030935	CELLULAR	5	2	0.0318	1	sheet-forming collagen
GO:0002443	PROCESS	66	4	0.0320	1	leukocyte mediated immunity
GO:0042805	FUNCTION	4	2	0.0324	1	actinin binding
GO:0032420	CELLULAR	12	3	0.0328	1	stereocilium
GO:0006869	PROCESS	131	8	0.0332	1	lipid transport
GO:0034375	PROCESS	11	2	0.0348	1	high-density lipoprotein particle remodeling
GO:0033293	FUNCTION	42	3	0.0350	1	monocarboxylic acid binding
GO:0051592	PROCESS	37	4	0.0364	1	response to calcium ion
GO:0010038	PROCESS	67	5	0.0376	1	response to metal ion
GO:0015931	PROCESS	98	5	0.0394	1	nucleobase, nucleoside, nucleotide and nucleic acid transport

GO:0050810	PROCESS	17	2	0.0398	1	regulation of steroid biosynthetic process
GO:0030414	FUNCTION	141	6	0.0400	1	peptidase inhibitor activity
GO:0032421	CELLULAR	13	3	0.0400	1	stereocilium bundle
GO:0034364	CELLULAR	20	2	0.0406	1	high-density lipoprotein particle
GO:0010596	PROCESS	12	2	0.0408	1	negative regulation of endothelial cell migration
GO:0016064	PROCESS	44	3	0.0412	1	immunoglobulin mediated immune response
GO:0010035	PROCESS	72	5	0.0422	1	response to inorganic substance
GO:0010876	PROCESS	142	8	0.0426	1	lipid localization
GO:0019724	PROCESS	46	3	0.0450	1	B cell mediated immunity
GO:0045940	PROCESS	11	2	0.0464	1	positive regulation of steroid metabolic process
GO:0005634	CELLULAR	4560	86	0.0474	1	nucleus
GO:0051179	PROCESS	2732	80	0.0486	1	localization
GO:0044445	CELLULAR	128	4	0.0506	1	cytosolic part
GO:0048168	PROCESS	16	2	0.0510	1	regulation of neuronal synaptic plasticity
GO:0015914	PROCESS	28	4	0.0526	1	phospholipid transport
GO:0000139	CELLULAR	172	8	0.0530	1	Golgi membrane
GO:0008376	FUNCTION	31	4	0.0558	1	acetylgalactosaminyltransferase activity
GO:0046068	PROCESS	9	2	0.0558	1	cGMP metabolic process
GO:0030261	PROCESS	24	2	0.0564	1	chromosome condensation
GO:0051605	PROCESS	65	4	0.0566	1	protein maturation by peptide bond cleavage
GO:0009225	PROCESS	11	2	0.0572	1	nucleotide-sugar metabolic process
GO:0015485	FUNCTION	15	2	0.0576	1	cholesterol binding
GO:0017127	FUNCTION	12	2	0.0578	1	cholesterol transporter activity
GO:0012505	CELLULAR	681	22	0.0586	1	endomembrane system
GO:0051235	PROCESS	53	3	0.0590	1	maintenance of location
GO:0006952	PROCESS	510	14	0.0596	1	defense response
GO:0048738	PROCESS	28	3	0.0596	1	cardiac muscle tissue development
GO:0005548	FUNCTION	28	4	0.0598	1	phospholipid transporter activity
GO:0030374	FUNCTION	30	2	0.0602	1	ligand-dependent nuclear receptor transcription coactivator activity
GO:0001937	PROCESS	17	2	0.0608	1	negative regulation of endothelial cell proliferation
GO:0015294	FUNCTION	74	5	0.0608	1	solute:cation symporter activity
GO:0016485	PROCESS	95	5	0.0616	1	protein processing
GO:0015293	FUNCTION	121	7	0.0620	1	symporter activity
GO:0030193	PROCESS	25	2	0.0626	1	regulation of blood coagulation
GO:0005902	CELLULAR	25	3	0.0626	1	microvillus
GO:0001764	PROCESS	42	4	0.0634	1	neuron migration
GO:0004867	FUNCTION	88	4	0.0664	1	serine-type endopeptidase inhibitor activity
GO:0005504	FUNCTION	28	2	0.0668	1	fatty acid binding
GO:0001523	PROCESS	20	2	0.0672	1	retinoid metabolic process
GO:0016101	PROCESS	20	2	0.0672	1	diterpenoid metabolic process
GO:0033273	PROCESS	22	2	0.0674	1	response to vitamin
GO:0008375	FUNCTION	35	3	0.0684	1	acetylglucosaminyltransferase activity
GO:0031290	PROCESS	9	2	0.0688	1	retinal ganglion cell axon guidance
GO:0004690	FUNCTION	8	2	0.0692	1	cyclic nucleotide-dependent protein kinase activity
GO:0050658	PROCESS	83	4	0.0706	1	RNA transport
GO:0050657	PROCESS	83	4	0.0706	1	nucleic acid transport
GO:0051236	PROCESS	83	4	0.0706	1	establishment of RNA localization
GO:0006721	PROCESS	22	2	0.0720	1	terpenoid metabolic process
GO:0051604	PROCESS	104	5	0.0722	1	protein maturation
GO:0050818	PROCESS	27	2	0.0722	1	regulation of coagulation
GO:0034369	PROCESS	18	2	0.0740	1	plasma lipoprotein particle remodeling
GO:0034368	PROCESS	18	2	0.0740	1	protein-lipid complex remodeling
GO:0034367	PROCESS	18	2	0.0740	1	macromolecular complex remodeling
GO:0051234	PROCESS	2346	66	0.0742	1	establishment of localization
GO:0022603	PROCESS	231	10	0.0758	1	regulation of anatomical structure morphogenesis
GO:0007051	PROCESS	39	2	0.0758	1	spindle organization
GO:0015934	CELLULAR	58	2	0.0758	1	large ribosomal subunit
GO:0030173	CELLULAR	43	4	0.0760	1	integral to Golgi membrane
GO:0030099	PROCESS	64	3	0.0776	1	myeloid cell differentiation
GO:0006917	PROCESS	227	8	0.0780	1	induction of apoptosis
GO:0019538	PROCESS	2512	58	0.0784	1	protein metabolic process
GO:0006081	PROCESS	25	2	0.0786	1	cellular aldehyde metabolic process
GO:0009593	PROCESS	29	3	0.0802	1	detection of chemical stimulus
GO:0031098	PROCESS	50	3	0.0804	1	stress-activated protein kinase signaling pathway
GO:0002449	PROCESS	56	3	0.0808	1	lymphocyte mediated immunity
GO:0015291	FUNCTION	186	9	0.0810	1	secondary active transmembrane transporter activity
GO:0050896	PROCESS	2782	59	0.0812	1	response to stimulus
GO:0030518	PROCESS	53	3	0.0814	1	steroid hormone receptor signaling pathway
GO:0015301	FUNCTION	19	2	0.0818	1	anion:anion antiporter activity
GO:0006954	PROCESS	263	8	0.0824	1	inflammatory response

GO:0006044	PROCESS	20	2	0.0830	1	N-acetylglucosamine metabolic process
GO:0005769	CELLULAR	64	3	0.0832	1	early endosome
GO:0051028	PROCESS	74	3	0.0834	1	mRNA transport
GO:0006403	PROCESS	85	4	0.0846	1	RNA localization
GO:0004871	FUNCTION	1929	55	0.0850	1	signal transducer activity
GO:0060089	FUNCTION	1929	55	0.0850	1	molecular transducer activity
GO:0001843	PROCESS	17	2	0.0850	1	neural tube closure
GO:0060606	PROCESS	17	2	0.0850	1	tube closure
GO:0006041	PROCESS	21	2	0.0860	1	glucosamine metabolic process
GO:0006096	PROCESS	38	2	0.0862	1	glycolysis
GO:0002460	PROCESS	61	3	0.0874	1	adaptive immune response based on somatic recombination of immune receptors built from immunoglobulin superfamily domains
GO:0002250	PROCESS	61	3	0.0874	1	adaptive immune response
GO:0016055	PROCESS	104	4	0.0878	1	Wnt receptor signaling pathway
GO:0050767	PROCESS	110	6	0.0888	1	regulation of neurogenesis
GO:0008194	FUNCTION	112	6	0.0890	1	UDP-glycosyltransferase activity
GO:0012502	PROCESS	228	8	0.0892	1	induction of programmed cell death
GO:0031985	CELLULAR	18	2	0.0902	1	Golgi cisterna
GO:0004866	FUNCTION	134	5	0.0904	1	endopeptidase inhibitor activity
GO:0005328	FUNCTION	17	2	0.0906	1	neurotransmitter:sodium symporter activity
GO:0035148	PROCESS	18	2	0.0914	1	tube lumen formation
GO:0044238	PROCESS	4851	98	0.0918	1	primary metabolic process
GO:0014020	PROCESS	19	2	0.0940	1	primary neural tube formation
GO:0004674	FUNCTION	393	15	0.0942	1	protein serine/threonine kinase activity
GO:0031300	CELLULAR	135	6	0.0946	1	intrinsic to organelle membrane
GO:0050866	PROCESS	37	2	0.0948	1	negative regulation of cell activation
GO:0005086	FUNCTION	16	2	0.0960	1	ARF guanyl-nucleotide exchange factor activity
GO:0004722	FUNCTION	41	2	0.0984	1	protein serine/threonine phosphatase activity
GO:0016455	FUNCTION	27	2	0.0990	1	RNA polymerase II transcription mediator activity
GO:0034358	CELLULAR	30	2	0.0994	1	plasma lipoprotein particle
GO:0032994	CELLULAR	30	2	0.0994	1	protein-lipid complex
GO:0003702	FUNCTION	217	7	0.1004	1	RNA polymerase II transcription factor activity
GO:0032934	FUNCTION	22	2	0.1006	1	sterol binding
GO:0008015	PROCESS	143	6	0.1022	1	blood circulation
GO:0003013	PROCESS	143	6	0.1022	1	circulatory system process
GO:0007059	PROCESS	72	3	0.1026	1	chromosome segregation
GO:0043407	PROCESS	30	2	0.1046	1	negative regulation of MAP kinase activity
GO:0006996	PROCESS	1114	26	0.1048	1	organelle organization
GO:0046890	PROCESS	30	2	0.1048	1	regulation of lipid biosynthetic process
GO:0002376	PROCESS	805	19	0.1056	1	immune system process
GO:0015459	FUNCTION	22	2	0.1066	1	potassium channel regulator activity
GO:0051018	FUNCTION	9	2	0.1080	1	protein kinase A binding
GO:0002252	PROCESS	95	4	0.1088	1	immune effector process
GO:0006810	PROCESS	2324	64	0.1106	1	transport
GO:0005326	FUNCTION	20	2	0.1106	1	neurotransmitter transporter activity
GO:0055037	CELLULAR	18	2	0.1108	1	recycling endosome
GO:0002526	PROCESS	74	3	0.1118	1	acute inflammatory response
GO:0031984	CELLULAR	20	2	0.1128	1	organelle subcompartment
GO:0050768	PROCESS	30	2	0.1130	1	negative regulation of neurogenesis
GO:0006007	PROCESS	45	2	0.1136	1	glucose catabolic process
GO:0003007	PROCESS	59	3	0.1144	1	heart morphogenesis
GO:0051960	PROCESS	125	6	0.1154	1	regulation of nervous system development
GO:0000794	CELLULAR	36	2	0.1180	1	condensed nuclear chromosome
GO:0001841	PROCESS	24	2	0.1188	1	neural tube formation
GO:0006915	PROCESS	445	12	0.1190	1	apoptosis
GO:0055007	PROCESS	17	2	0.1204	1	cardiac muscle cell differentiation
GO:0006066	PROCESS	339	10	0.1212	1	alcohol metabolic process
GO:0030675	FUNCTION	8	2	0.1220	1	Rac GTPase activator activity
GO:0030336	PROCESS	49	3	0.1226	1	negative regulation of cell migration
GO:0030522	PROCESS	63	3	0.1228	1	intracellular receptor-mediated signaling pathway
GO:0005768	CELLULAR	260	8	0.1238	1	endosome
GO:0045087	PROCESS	120	4	0.1238	1	innate immune response
GO:0005513	PROCESS	9	2	0.1248	1	detection of calcium ion
GO:0006979	PROCESS	106	4	0.1254	1	response to oxidative stress
GO:0043170	PROCESS	3772	76	0.1258	1	macromolecule metabolic process
GO:0001838	PROCESS	25	2	0.1264	1	embryonic epithelial tube formation
GO:0005215	FUNCTION	1072	36	0.1268	1	transporter activity
GO:0012501	PROCESS	452	12	0.1270	1	programmed cell death
GO:0033036	PROCESS	943	25	0.1274	1	macromolecule localization
GO:0006469	PROCESS	72	3	0.1276	1	negative regulation of protein kinase activity
GO:0019239	FUNCTION	24	2	0.1282	1	deaminase activity

GO:0008624	PROCESS	59	3	0.1286	1	induction of apoptosis by extracellular signals
GO:0045664	PROCESS	85	5	0.1286	1	regulation of neuron differentiation
GO:0015297	FUNCTION	59	4	0.1292	1	antiporter activity
GO:0007242	PROCESS	1079	31	0.1294	1	intracellular signaling cascade
GO:0010721	PROCESS	32	2	0.1294	1	negative regulation of cell development
GO:0042596	PROCESS	14	2	0.1308	1	fear response
GO:0004672	FUNCTION	556	20	0.1318	1	protein kinase activity
GO:0016757	FUNCTION	250	9	0.1320	1	transferase activity, transferring glycosyl groups
GO:0004872	FUNCTION	1515	42	0.1324	1	receptor activity
GO:0007283	PROCESS	228	6	0.1330	1	spermatogenesis
GO:0048232	PROCESS	228	6	0.1330	1	male gamete generation
GO:0005227	FUNCTION	17	2	0.1330	1	calcium activated cation channel activity
GO:0002253	PROCESS	66	3	0.1332	1	activation of immune response
GO:0006829	PROCESS	23	2	0.1354	1	zinc ion transport
GO:0016125	PROCESS	87	4	0.1364	1	sterol metabolic process
GO:0042169	FUNCTION	21	2	0.1366	1	SH2 domain binding
GO:0033673	PROCESS	76	3	0.1376	1	negative regulation of kinase activity
GO:0005003	FUNCTION	17	2	0.1376	1	ephrin receptor activity
GO:0043086	PROCESS	233	7	0.1382	1	negative regulation of catalytic activity
GO:0006959	PROCESS	68	3	0.1388	1	humoral immune response
GO:0016740	FUNCTION	1562	41	0.1392	1	transferase activity
GO:0015370	FUNCTION	43	3	0.1394	1	solute:sodium symporter activity
GO:0000910	PROCESS	36	2	0.1396	1	cytokinesis
GO:0021575	PROCESS	16	2	0.1398	1	hindbrain morphogenesis
GO:0045786	PROCESS	65	3	0.1424	1	negative regulation of cell cycle
GO:0006950	PROCESS	1330	29	0.1430	1	response to stress
GO:0031329	PROCESS	40	2	0.1452	1	regulation of cellular catabolic process
GO:0045834	PROCESS	30	2	0.1484	1	positive regulation of lipid metabolic process
GO:0043623	PROCESS	134	4	0.1486	1	cellular protein complex assembly
GO:0030036	PROCESS	187	8	0.1488	1	actin cytoskeleton organization
GO:0022892	FUNCTION	878	31	0.1492	1	substrate-specific transporter activity
GO:0007265	PROCESS	92	4	0.1496	1	Ras protein signal transduction
GO:0035051	PROCESS	20	2	0.1516	1	cardiac cell differentiation
GO:0042054	FUNCTION	38	2	0.1532	1	histone methyltransferase activity
GO:0051348	PROCESS	81	3	0.1534	1	negative regulation of transferase activity
GO:0044267	PROCESS	2072	46	0.1542	1	cellular protein metabolic process
GO:0016765	FUNCTION	46	2	0.1552	1	transferase activity, transferring alkyl or aryl (other than methyl) groups
GO:0019897	CELLULAR	44	3	0.1566	1	extrinsic to plasma membrane
GO:0051651	PROCESS	41	2	0.1588	1	maintenance of location in cell
GO:0001654	PROCESS	91	4	0.1610	1	eye development
GO:0019218	PROCESS	27	2	0.1616	1	regulation of steroid metabolic process
GO:0006836	PROCESS	67	4	0.1616	1	neurotransmitter transport
GO:0006412	PROCESS	274	5	0.1618	1	translation
GO:0015833	PROCESS	34	2	0.1618	1	peptide transport
GO:0016773	FUNCTION	664	22	0.1626	1	phosphotransferase activity, alcohol group as acceptor
GO:0000165	PROCESS	142	5	0.1632	1	MAPKKK cascade
GO:0043065	PROCESS	303	9	0.1640	1	positive regulation of apoptosis
GO:0016251	FUNCTION	47	2	0.1652	1	general RNA polymerase II transcription factor activity
GO:0003777	FUNCTION	72	4	0.1656	1	microtubule motor activity
GO:0005996	PROCESS	165	5	0.1672	1	monosaccharide metabolic process
GO:0006289	PROCESS	51	2	0.1680	1	nucleotide-excision repair
GO:0019320	PROCESS	55	2	0.1684	1	hexose catabolic process
GO:0060284	PROCESS	138	6	0.1690	1	regulation of cell development
GO:0006928	PROCESS	360	15	0.1694	1	cell motion
GO:0051674	PROCESS	360	15	0.1694	1	localization of cell
GO:0055085	PROCESS	204	6	0.1718	1	transmembrane transport
GO:0016247	FUNCTION	46	3	0.1728	1	channel regulator activity
GO:0005581	CELLULAR	33	3	0.1740	1	collagen
GO:0050770	PROCESS	36	3	0.1750	1	regulation of axonogenesis
GO:0007165	PROCESS	3008	76	0.1752	1	signal transduction
GO:0050793	PROCESS	1051	28	0.1756	1	regulation of developmental process
GO:0005102	FUNCTION	771	20	0.1760	1	receptor binding
GO:0006629	PROCESS	733	19	0.1762	1	lipid metabolic process
GO:0005085	FUNCTION	140	8	0.1762	1	guanyl-nucleotide exchange factor activity
GO:0005125	FUNCTION	191	4	0.1762	1	cytokine activity
GO:0009055	FUNCTION	197	6	0.1770	1	electron carrier activity
GO:0005794	CELLULAR	771	23	0.1802	1	Golgi apparatus
GO:0016310	PROCESS	694	21	0.1804	1	phosphorylation
GO:0009059	PROCESS	929	19	0.1818	1	macromolecule biosynthetic process
GO:0044275	PROCESS	57	2	0.1824	1	cellular carbohydrate catabolic process

GO:0007411	PROCESS	70	6	0.1828	1	axon guidance
GO:0008373	FUNCTION	20	2	0.1832	1	sialyltransferase activity
GO:0048523	PROCESS	1310	31	0.1836	1	negative regulation of cellular process
GO:0016301	FUNCTION	713	23	0.1836	1	kinase activity
GO:0005975	PROCESS	454	12	0.1840	1	carbohydrate metabolic process
GO:0005789	CELLULAR	229	7	0.1850	1	endoplasmic reticulum membrane
GO:0043283	PROCESS	3666	73	0.1858	1	biopolymer metabolic process
GO:0006163	PROCESS	155	6	0.1858	1	purine nucleotide metabolic process
GO:0030029	PROCESS	203	8	0.1878	1	actin filament-based process
GO:0016763	FUNCTION	45	2	0.1886	1	transferase activity, transferring pentosyl groups
GO:0043068	PROCESS	305	9	0.1896	1	positive regulation of programmed cell death
GO:0005777	CELLULAR	97	3	0.1900	1	peroxisome
GO:0042579	CELLULAR	97	3	0.1900	1	microbody
GO:0043405	PROCESS	115	4	0.1910	1	regulation of MAP kinase activity
GO:0031327	PROCESS	459	12	0.1914	1	negative regulation of cellular biosynthetic process
GO:0051301	PROCESS	266	6	0.1914	1	cell division
GO:0015457	FUNCTION	50	3	0.1932	1	auxiliary transport protein activity
GO:0006720	PROCESS	41	2	0.1934	1	isoprenoid metabolic process
GO:0006818	PROCESS	55	2	0.1942	1	hydrogen transport
GO:0048878	PROCESS	382	12	0.1958	1	chemical homeostasis
GO:0008276	FUNCTION	47	2	0.1958	1	protein methyltransferase activity
GO:0006024	PROCESS	20	2	0.1960	1	glycosaminoglycan biosynthetic process
GO:0010942	PROCESS	308	9	0.1968	1	positive regulation of cell death
GO:0051271	PROCESS	54	3	0.1968	1	negative regulation of cell motion
GO:0045892	PROCESS	283	8	0.1968	1	negative regulation of transcription, DNA-dependent
GO:0043284	PROCESS	740	14	0.1970	1	biopolymer biosynthetic process
GO:0044431	CELLULAR	257	9	0.1986	1	Golgi apparatus part
GO:0009890	PROCESS	465	12	0.1986	1	negative regulation of biosynthetic process
GO:0000226	PROCESS	108	3	0.2016	1	microtubule cytoskeleton organization
GO:0006023	PROCESS	22	2	0.2016	1	aminoglycan biosynthetic process
GO:0000793	CELLULAR	102	3	0.2032	1	condensed chromosome
GO:0051253	PROCESS	288	8	0.2038	1	negative regulation of RNA metabolic process
GO:0016209	FUNCTION	44	2	0.2044	1	antioxidant activity
GO:0030145	FUNCTION	147	6	0.2050	1	manganese ion binding
GO:0002009	PROCESS	69	3	0.2054	1	morphogenesis of an epithelium
GO:0048592	PROCESS	54	3	0.2058	1	eye morphogenesis
GO:0006164	PROCESS	130	5	0.2064	1	purine nucleotide biosynthetic process
GO:0048519	PROCESS	1432	34	0.2072	1	negative regulation of biological process
GO:0043010	PROCESS	71	3	0.2076	1	camera-type eye development
GO:0009165	PROCESS	165	6	0.2084	1	nucleotide biosynthetic process
GO:0048609	PROCESS	68	3	0.2102	1	reproductive process in a multicellular organism
GO:0032504	PROCESS	68	3	0.2102	1	multicellular organism reproduction
GO:0050865	PROCESS	125	4	0.2114	1	regulation of cell activation
GO:0034645	PROCESS	911	18	0.2122	1	cellular macromolecule biosynthetic process
GO:0044260	PROCESS	3299	64	0.2124	1	cellular macromolecule metabolic process
GO:0009152	PROCESS	105	4	0.2124	1	purine ribonucleotide biosynthetic process
GO:0006323	PROCESS	98	2	0.2124	1	DNA packaging
GO:0010975	PROCESS	42	3	0.2126	1	regulation of neuron projection development
GO:0022604	PROCESS	97	5	0.2136	1	regulation of cell morphogenesis
GO:0060429	PROCESS	116	4	0.2138	1	epithelium development
GO:0033279	CELLULAR	112	2	0.2160	1	ribosomal subunit
GO:0051094	PROCESS	504	14	0.2162	1	positive regulation of developmental process
GO:0006897	PROCESS	181	7	0.2164	1	endocytosis
GO:0010324	PROCESS	181	7	0.2164	1	membrane invagination
GO:0032102	PROCESS	30	2	0.2166	1	negative regulation of response to external stimulus
GO:0046486	PROCESS	127	4	0.2166	1	glycerolipid metabolic process
GO:0004697	FUNCTION	14	2	0.2166	1	protein kinase C activity
GO:0008324	FUNCTION	492	19	0.2172	1	cation transmembrane transporter activity
GO:0007017	PROCESS	205	6	0.2172	1	microtubule-based process
GO:0000776	CELLULAR	66	2	0.2172	1	kinetochore
GO:0005764	CELLULAR	189	5	0.2194	1	lysosome
GO:0000323	CELLULAR	189	5	0.2194	1	lytic vacuole
GO:0030258	PROCESS	57	2	0.2196	1	lipid modification
GO:0007276	PROCESS	285	7	0.2196	1	gamete generation
GO:0048585	PROCESS	68	3	0.2204	1	negative regulation of response to stimulus
GO:0035091	FUNCTION	92	4	0.2204	1	phosphoinositide binding
GO:0046907	PROCESS	545	13	0.2206	1	intracellular transport
GO:0050880	PROCESS	41	2	0.2208	1	regulation of blood vessel size
GO:0035150	PROCESS	41	2	0.2208	1	regulation of tube size
GO:0031324	PROCESS	567	14	0.2212	1	negative regulation of cellular metabolic process
GO:0005643	CELLULAR	73	2	0.2218	1	nuclear pore

GO:0006766	PROCESS	82	3	0.2220	1	vitamin metabolic process
GO:0048015	PROCESS	75	3	0.2220	1	phosphoinositide-mediated signaling
GO:0042981	PROCESS	607	15	0.2222	1	regulation of apoptosis
GO:0046365	PROCESS	64	2	0.2236	1	monosaccharide catabolic process
GO:0005576	CELLULAR	1821	43	0.2248	1	extracellular region
GO:0016758	FUNCTION	170	6	0.2250	1	transferase activity, transferring hexosyl groups
GO:0006605	PROCESS	180	5	0.2250	1	protein targeting
GO:0043687	PROCESS	1041	29	0.2268	1	post-translational protein modification
GO:0016616	FUNCTION	96	3	0.2272	1	oxidoreductase activity, acting on the CH-OH group of donors, NAD or NADP as acceptor
GO:0030148	PROCESS	27	2	0.2274	1	sphingolipid biosynthetic process
GO:0008202	PROCESS	174	6	0.2282	1	steroid metabolic process
GO:0015300	FUNCTION	48	3	0.2296	1	solute:solute antiporter activity
GO:0051146	PROCESS	34	2	0.2302	1	striated muscle cell differentiation
GO:0051168	PROCESS	52	2	0.2304	1	nuclear export
GO:0007194	PROCESS	44	3	0.2310	1	negative regulation of adenylate cyclase activity
GO:0031280	PROCESS	44	3	0.2310	1	negative regulation of cyclase activity
GO:0051350	PROCESS	44	3	0.2310	1	negative regulation of lyase activity
GO:0007154	PROCESS	3311	83	0.2320	1	cell communication
GO:0021915	PROCESS	41	2	0.2320	1	neural tube development
GO:0016331	PROCESS	41	2	0.2324	1	morphogenesis of embryonic epithelium
GO:0042175	CELLULAR	242	7	0.2348	1	nuclear envelope-endoplasmic reticulum network
GO:0010769	PROCESS	49	3	0.2354	1	regulation of cell morphogenesis involved in differentiation
GO:0060090	FUNCTION	58	3	0.2356	1	molecular adaptor activity
GO:0009117	PROCESS	247	8	0.2362	1	nucleotide metabolic process
GO:0006753	PROCESS	247	8	0.2362	1	nucleoside phosphate metabolic process
GO:0007018	PROCESS	96	4	0.2370	1	microtubule-based movement
GO:0007169	PROCESS	187	8	0.2372	1	transmembrane receptor protein tyrosine kinase signaling pathway
GO:0007067	PROCESS	200	4	0.2372	1	mitosis
GO:0000280	PROCESS	200	4	0.2372	1	nuclear division
GO:0060562	PROCESS	37	2	0.2384	1	epithelial tube morphogenesis
GO:0007264	PROCESS	247	7	0.2396	1	small GTPase mediated signal transduction
GO:0045184	PROCESS	683	16	0.2406	1	establishment of protein localization
GO:0048568	PROCESS	36	2	0.2418	1	embryonic organ development
GO:0000287	FUNCTION	420	13	0.2430	1	magnesium ion binding
GO:0035023	PROCESS	88	5	0.2442	1	regulation of Rho protein signal transduction
GO:0009123	PROCESS	55	3	0.2442	1	nucleoside monophosphate metabolic process
GO:0003018	PROCESS	44	2	0.2444	1	vascular process in circulatory system
GO:0000087	PROCESS	202	4	0.2456	1	M phase of mitotic cell cycle
GO:0009058	PROCESS	1655	34	0.2462	1	biosynthetic process
GO:0007229	PROCESS	55	3	0.2462	1	integrin-mediated signaling pathway
GO:0003677	FUNCTION	2129	37	0.2466	1	DNA binding
GO:0016481	PROCESS	378	10	0.2480	1	negative regulation of transcription
GO:0009150	PROCESS	118	4	0.2494	1	purine ribonucleotide metabolic process
GO:0008305	CELLULAR	28	2	0.2496	1	integrin complex
GO:0043412	PROCESS	1354	35	0.2500	1	biopolymer modification
GO:0008170	FUNCTION	51	2	0.2514	1	N-methyltransferase activity
GO:0051649	PROCESS	694	17	0.2516	1	establishment of localization in cell
GO:0005524	FUNCTION	1360	37	0.2518	1	ATP binding
GO:0006796	PROCESS	847	25	0.2522	1	phosphate metabolic process
GO:0006793	PROCESS	847	25	0.2522	1	phosphorus metabolic process
GO:0030554	FUNCTION	1446	39	0.2524	1	adenyl nucleotide binding
GO:0006767	PROCESS	52	2	0.2526	1	water-soluble vitamin metabolic process
GO:0044265	PROCESS	690	13	0.2526	1	cellular macromolecule catabolic process
GO:0044092	PROCESS	277	7	0.2536	1	negative regulation of molecular function
GO:0009100	PROCESS	181	7	0.2548	1	glycoprotein metabolic process
GO:0009888	PROCESS	536	13	0.2552	1	tissue development
GO:0043025	CELLULAR	73	3	0.2552	1	cell soma
GO:0046467	PROCESS	30	2	0.2558	1	membrane lipid biosynthetic process
GO:0031090	CELLULAR	931	21	0.2588	1	organelle membrane
GO:0031344	PROCESS	54	3	0.2596	1	regulation of cell projection organization
GO:0008203	PROCESS	78	3	0.2598	1	cholesterol metabolic process
GO:0006464	PROCESS	1288	34	0.2604	1	protein modification process
GO:0002682	PROCESS	279	7	0.2606	1	regulation of immune system process
GO:0048285	PROCESS	207	4	0.2606	1	organelle fission
GO:0022414	PROCESS	544	12	0.2608	1	reproductive process
GO:0035239	PROCESS	70	3	0.2608	1	tube morphogenesis
GO:0022804	FUNCTION	319	12	0.2616	1	active transmembrane transporter activity
GO:0000003	PROCESS	548	12	0.2618	1	reproduction
GO:0010558	PROCESS	450	11	0.2618	1	negative regulation of macromolecule biosynthetic process

GO:0043067	PROCESS	616	15	0.2626	1	regulation of programmed cell death
GO:0014706	PROCESS	95	4	0.2626	1	striated muscle tissue development
GO:0010941	PROCESS	618	15	0.2636	1	regulation of cell death
GO:0048598	PROCESS	220	6	0.2640	1	embryonic morphogenesis
GO:0005100	FUNCTION	23	2	0.2646	1	Rho GTPase activator activity
GO:0044255	PROCESS	598	15	0.2654	1	cellular lipid metabolic process
GO:0007254	PROCESS	47	2	0.2658	1	JNK cascade
GO:0034961	PROCESS	730	13	0.2662	1	cellular biopolymer biosynthetic process
GO:0005802	CELLULAR	30	2	0.2668	1	trans-Golgi network
GO:0016614	FUNCTION	108	3	0.2672	1	oxidoreductase activity, acting on CH-OH group of donors
GO:0044420	CELLULAR	97	5	0.2684	1	extracellular matrix part
GO:0001883	FUNCTION	1462	39	0.2694	1	purine nucleoside binding
GO:0006650	PROCESS	95	3	0.2694	1	glycerophospholipid metabolic process
GO:0050727	PROCESS	53	2	0.2696	1	regulation of inflammatory response
GO:0031328	PROCESS	535	14	0.2700	1	positive regulation of cellular biosynthetic process
GO:0003823	FUNCTION	40	2	0.2702	1	antigen binding
GO:0030802	PROCESS	88	4	0.2704	1	regulation of cyclic nucleotide biosynthetic process
GO:0030808	PROCESS	88	4	0.2704	1	regulation of nucleotide biosynthetic process
GO:0044249	PROCESS	1585	32	0.2706	1	cellular biosynthetic process
GO:0003700	FUNCTION	879	19	0.2712	1	transcription factor activity
GO:0050778	PROCESS	93	3	0.2716	1	positive regulation of immune response
GO:0006941	PROCESS	30	2	0.2718	1	striated muscle contraction
GO:0009260	PROCESS	112	4	0.2752	1	ribonucleotide biosynthetic process
GO:0009891	PROCESS	538	14	0.2756	1	positive regulation of biosynthetic process
GO:0006260	PROCESS	163	4	0.2758	1	DNA replication
GO:0032501	PROCESS	3421	83	0.2762	1	multicellular organismal process
GO:0007399	PROCESS	801	28	0.2772	1	nervous system development
GO:0046164	PROCESS	72	2	0.2780	1	alcohol catabolic process
GO:0010648	PROCESS	205	6	0.2782	1	negative regulation of cell communication
GO:0044262	PROCESS	257	7	0.2790	1	cellular carbohydrate metabolic process
GO:0032012	PROCESS	41	2	0.2792	1	regulation of ARF protein signal transduction
GO:0048593	PROCESS	34	2	0.2802	1	camera-type eye morphogenesis
GO:0032559	FUNCTION	1374	37	0.2804	1	adenyl ribonucleotide binding
GO:0055086	PROCESS	267	8	0.2804	1	nucleobase, nucleoside and nucleotide metabolic process
GO:0030384	PROCESS	61	2	0.2820	1	phosphoinositide metabolic process
GO:0001882	FUNCTION	1471	39	0.2824	1	nucleoside binding
GO:0060537	PROCESS	100	4	0.2830	1	muscle tissue development
GO:0022411	PROCESS	54	2	0.2848	1	cellular component disassembly
GO:0051606	PROCESS	98	4	0.2864	1	detection of stimulus
GO:0032787	PROCESS	266	7	0.2876	1	monocarboxylic acid metabolic process
GO:0016702	FUNCTION	60	2	0.2892	1	oxidoreductase activity, acting on single donors with incorporation of molecular oxygen, incorporation of two atoms of oxygen
GO:0051213	FUNCTION	60	2	0.2892	1	dioxygenase activity
GO:0044444	CELLULAR	4277	82	0.2898	1	cytoplasmic part
GO:0033555	PROCESS	25	2	0.2898	1	multicellular organismal response to stress
GO:0016701	FUNCTION	61	2	0.2908	1	oxidoreductase activity, acting on single donors with incorporation of molecular oxygen
GO:0045765	PROCESS	83	3	0.2910	1	regulation of angiogenesis
GO:0005795	CELLULAR	40	2	0.2912	1	Golgi stack
GO:0006468	PROCESS	586	18	0.2914	1	protein amino acid phosphorylation
GO:0030799	PROCESS	91	4	0.2944	1	regulation of cyclic nucleotide metabolic process
GO:0007266	PROCESS	32	2	0.2950	1	Rho protein signal transduction
GO:0031091	CELLULAR	53	2	0.2978	1	platelet alpha granule
GO:0001936	PROCESS	29	2	0.2992	1	regulation of endothelial cell proliferation
GO:0044248	PROCESS	951	18	0.2994	1	cellular catabolic process
GO:0006487	PROCESS	41	2	0.2996	1	protein amino acid N-linked glycosylation
GO:0010594	PROCESS	49	2	0.3008	1	regulation of endothelial cell migration
GO:0022403	PROCESS	365	7	0.3024	1	cell cycle phase
GO:0000271	PROCESS	40	2	0.3036	1	polysaccharide biosynthetic process
GO:0006936	PROCESS	123	5	0.3048	1	muscle contraction
GO:0004857	FUNCTION	247	6	0.3054	1	enzyme inhibitor activity
GO:0007423	PROCESS	159	5	0.3056	1	sensory organ development
GO:0008016	PROCESS	60	3	0.3058	1	regulation of heart contraction
GO:0030674	FUNCTION	84	3	0.3062	1	protein binding, bridging
GO:0006140	PROCESS	94	4	0.3064	1	regulation of nucleotide metabolic process
GO:0000278	PROCESS	327	6	0.3066	1	mitotic cell cycle
GO:0008135	FUNCTION	88	2	0.3092	1	translation factor activity, nucleic acid binding
GO:0008643	PROCESS	57	2	0.3098	1	carbohydrate transport
GO:0010467	PROCESS	1095	18	0.3106	1	gene expression
GO:0030005	PROCESS	163	5	0.3122	1	cellular di-, tri-valent inorganic cation homeostasis

GO:0031301	CELLULAR	114	4	0.3130	1	integral to organelle membrane
GO:0009892	PROCESS	618	14	0.3148	1	negative regulation of metabolic process
GO:0022857	FUNCTION	816	28	0.3150	1	transmembrane transporter activity
GO:0015082	FUNCTION	31	2	0.3158	1	di-, tri-valent inorganic cation transmembrane transporter activity
GO:0016772	FUNCTION	829	23	0.3172	1	transferase activity, transferring phosphorus-containing groups
GO:0015031	PROCESS	677	15	0.3176	1	protein transport
GO:0046930	CELLULAR	86	2	0.3190	1	pore complex
GO:0035257	FUNCTION	69	2	0.3200	1	nuclear hormone receptor binding
GO:0031325	PROCESS	657	16	0.3212	1	positive regulation of cellular metabolic process
GO:0016651	FUNCTION	70	2	0.3218	1	oxidoreductase activity, acting on NADH or NADPH
GO:0006754	PROCESS	78	3	0.3220	1	ATP biosynthetic process
GO:0050801	PROCESS	301	9	0.3222	1	ion homeostasis
GO:0009259	PROCESS	127	4	0.3228	1	ribonucleotide metabolic process
GO:0003735	FUNCTION	146	2	0.3238	1	structural constituent of ribosome
GO:0030528	FUNCTION	1358	28	0.3264	1	transcription regulator activity
GO:0044421	CELLULAR	842	22	0.3274	1	extracellular region part
GO:0007584	PROCESS	66	2	0.3274	1	response to nutrient
GO:0044237	PROCESS	4582	85	0.3290	1	cellular metabolic process
GO:0019953	PROCESS	342	7	0.3294	1	sexual reproduction
GO:0009615	PROCESS	96	2	0.3312	1	response to virus
GO:0010629	PROCESS	420	10	0.3328	1	negative regulation of gene expression
GO:0055066	PROCESS	173	5	0.3340	1	di-, tri-valent inorganic cation homeostasis
GO:0051707	PROCESS	216	4	0.3348	1	response to other organism
GO:0022890	FUNCTION	126	4	0.3352	1	inorganic cation transmembrane transporter activity
GO:0042592	PROCESS	544	14	0.3356	1	homeostatic process
GO:0009991	PROCESS	118	3	0.3356	1	response to extracellular stimulus
GO:0042802	FUNCTION	517	14	0.3366	1	identical protein binding
GO:0007166	PROCESS	1573	35	0.3372	1	cell surface receptor linked signal transduction
GO:0005070	FUNCTION	42	2	0.3382	1	SH3/SH2 adaptor activity
GO:0009057	PROCESS	1131	22	0.3388	1	macromolecule catabolic process
GO:0004620	FUNCTION	80	3	0.3394	1	phospholipase activity
GO:0051093	PROCESS	445	11	0.3400	1	negative regulation of developmental process
GO:0004653	FUNCTION	19	2	0.3404	1	polypeptide N-acetylgalactosaminyltransferase activity
GO:0009124	PROCESS	45	2	0.3412	1	nucleoside monophosphate biosynthetic process
GO:0046982	FUNCTION	139	4	0.3420	1	protein heterodimerization activity
GO:0043235	CELLULAR	100	4	0.3424	1	receptor complex
GO:0007015	PROCESS	54	2	0.3430	1	actin filament organization
GO:0005773	CELLULAR	222	5	0.3432	1	vacuole
GO:0051129	PROCESS	113	3	0.3444	1	negative regulation of cellular component organization
GO:0032101	PROCESS	114	4	0.3456	1	regulation of response to external stimulus
GO:0009206	PROCESS	87	3	0.3460	1	purine ribonucleoside triphosphate biosynthetic process
GO:0009145	PROCESS	87	3	0.3460	1	purine nucleoside triphosphate biosynthetic process
GO:0007588	PROCESS	46	2	0.3468	1	excretion
GO:0005741	CELLULAR	79	2	0.3472	1	mitochondrial outer membrane
GO:0007606	PROCESS	402	5	0.3474	1	sensory perception of chemical stimulus
GO:0008219	PROCESS	548	13	0.3476	1	cell death
GO:0019992	FUNCTION	63	4	0.3480	1	diacylglycerol binding
GO:0016042	PROCESS	155	4	0.3486	1	lipid catabolic process
GO:0045934	PROCESS	421	10	0.3488	1	negative regulation of nucleobase, nucleoside, nucleotide and nucleic acid metabolic process
GO:0009201	PROCESS	88	3	0.3488	1	ribonucleoside triphosphate biosynthetic process
GO:0009142	PROCESS	88	3	0.3488	1	nucleoside triphosphate biosynthetic process
GO:0002684	PROCESS	164	4	0.3494	1	positive regulation of immune system process
GO:0007507	PROCESS	158	5	0.3504	1	heart development
GO:0009187	PROCESS	31	2	0.3508	1	cyclic nucleotide metabolic process
GO:0051172	PROCESS	422	10	0.3508	1	negative regulation of nitrogen compound metabolic process
GO:0016051	PROCESS	86	3	0.3522	1	carbohydrate biosynthetic process
GO:0016563	FUNCTION	340	8	0.3540	1	transcription activator activity
GO:0051427	FUNCTION	75	2	0.3542	1	hormone receptor binding
GO:0016265	PROCESS	552	13	0.3544	1	death
GO:0046578	PROCESS	185	7	0.3548	1	regulation of Ras protein signal transduction
GO:0050730	PROCESS	56	2	0.3556	1	regulation of peptidyl-tyrosine phosphorylation
GO:0048729	PROCESS	134	4	0.3558	1	tissue morphogenesis
GO:0009611	PROCESS	399	9	0.3560	1	response to wounding
GO:0042803	FUNCTION	262	8	0.3564	1	protein homodimerization activity
GO:0030203	PROCESS	47	2	0.3582	1	glycosaminoglycan metabolic process
GO:0031402	FUNCTION	113	5	0.3584	1	sodium ion binding
GO:0034960	PROCESS	3182	59	0.3592	1	cellular biopolymer metabolic process
GO:0030165	FUNCTION	31	2	0.3592	1	PDZ domain binding
GO:0015078	FUNCTION	77	2	0.3602	1	hydrogen ion transmembrane transporter activity

GO:0000775	CELLULAR	106	2	0.3626	1	chromosome, centromeric region
GO:0045859	PROCESS	277	7	0.3636	1	regulation of protein kinase activity
GO:0005882	CELLULAR	159	2	0.3642	1	intermediate filament
GO:0048699	PROCESS	366	13	0.3648	1	generation of neurons
GO:0005615	CELLULAR	585	13	0.3662	1	extracellular space
GO:0017076	FUNCTION	1758	42	0.3664	1	purine nucleotide binding
GO:0005089	FUNCTION	66	4	0.3672	1	Rho guanyl-nucleotide exchange factor activity
GO:0006644	PROCESS	164	4	0.3674	1	phospholipid metabolic process
GO:0008277	PROCESS	48	2	0.3682	1	regulation of G-protein coupled receptor protein signaling pathway
GO:0016052	PROCESS	92	2	0.3682	1	carbohydrate catabolic process
GO:0045111	CELLULAR	160	2	0.3682	1	intermediate filament cytoskeleton
GO:0045595	PROCESS	352	10	0.3696	1	regulation of cell differentiation
GO:0031406	FUNCTION	107	3	0.3698	1	carboxylic acid binding
GO:0043549	PROCESS	282	7	0.3700	1	regulation of kinase activity
GO:0031175	PROCESS	154	8	0.3700	1	neuron projection development
GO:0048812	PROCESS	130	7	0.3718	1	neuron projection morphogenesis
GO:0005694	CELLULAR	386	6	0.3718	1	chromosome
GO:0032270	PROCESS	158	4	0.3734	1	positive regulation of cellular protein metabolic process
GO:0048167	PROCESS	29	2	0.3736	1	regulation of synaptic plasticity
GO:0016874	FUNCTION	355	8	0.3750	1	ligase activity
GO:0044422	CELLULAR	3701	65	0.3756	1	organelle part
GO:0000041	PROCESS	63	2	0.3756	1	transition metal ion transport
GO:0008757	FUNCTION	79	2	0.3762	1	S-adenosylmethionine-dependent methyltransferase activity
GO:0045935	PROCESS	484	12	0.3764	1	positive regulation of nucleobase, nucleoside, nucleotide and nucleic acid metabolic process
GO:0044432	CELLULAR	303	7	0.3782	1	endoplasmic reticulum part
GO:0034622	PROCESS	265	4	0.3804	1	cellular macromolecular complex assembly
GO:0005829	CELLULAR	1072	21	0.3808	1	cytosol
GO:0007275	PROCESS	2320	59	0.3812	1	multicellular organismal development
GO:0022891	FUNCTION	749	25	0.3812	1	substrate-specific transmembrane transporter activity
GO:0005604	CELLULAR	59	3	0.3826	1	basement membrane
GO:0006139	PROCESS	1542	27	0.3836	1	nucleobase, nucleoside, nucleotide and nucleic acid metabolic process
GO:0006913	PROCESS	130	3	0.3838	1	nucleocytoplasmic transport
GO:0048667	PROCESS	132	7	0.3840	1	cell morphogenesis involved in neuron differentiation
GO:0019637	PROCESS	170	4	0.3846	1	organophosphate metabolic process
GO:0006486	PROCESS	119	4	0.3852	1	protein amino acid glycosylation
GO:0043413	PROCESS	119	4	0.3852	1	biopolymer glycosylation
GO:0070085	PROCESS	119	4	0.3852	1	glycosylation
GO:0032386	PROCESS	60	2	0.3868	1	regulation of intracellular transport
GO:0044428	CELLULAR	1600	26	0.3872	1	nuclear part
GO:0046034	PROCESS	89	3	0.3872	1	ATP metabolic process
GO:0009887	PROCESS	522	14	0.3880	1	organ morphogenesis
GO:0034962	PROCESS	635	11	0.3880	1	cellular biopolymer catabolic process
GO:0051169	PROCESS	132	3	0.3886	1	nuclear transport
GO:0050660	FUNCTION	64	2	0.3896	1	FAD binding
GO:0000279	PROCESS	298	5	0.3922	1	M phase
GO:0006874	PROCESS	127	4	0.3922	1	cellular calcium ion homeostasis
GO:0050769	PROCESS	39	2	0.3922	1	positive regulation of neurogenesis
GO:0006006	PROCESS	96	2	0.3942	1	glucose metabolic process
GO:0008217	PROCESS	69	2	0.3950	1	regulation of blood pressure
GO:0006694	PROCESS	71	2	0.3950	1	steroid biosynthetic process
GO:0009101	PROCESS	144	5	0.3966	1	glycoprotein biosynthetic process
GO:0007218	PROCESS	80	3	0.3968	1	neuropeptide signaling pathway
GO:0005083	FUNCTION	233	9	0.3970	1	small GTPase regulator activity
GO:0000187	PROCESS	67	2	0.3970	1	activation of MAPK activity
GO:0051247	PROCESS	163	4	0.3974	1	positive regulation of protein metabolic process
GO:0048858	PROCESS	137	7	0.3978	1	cell projection morphogenesis
GO:0032555	FUNCTION	1684	40	0.4010	1	purine ribonucleotide binding
GO:0032553	FUNCTION	1684	40	0.4010	1	ribonucleotide binding
GO:0002694	PROCESS	119	3	0.4014	1	regulation of leukocyte activation
GO:0055074	PROCESS	130	4	0.4014	1	calcium ion homeostasis
GO:0051173	PROCESS	495	12	0.4026	1	positive regulation of nitrogen compound metabolic process
GO:0030326	PROCESS	54	2	0.4032	1	embryonic limb morphogenesis
GO:0035113	PROCESS	54	2	0.4032	1	embryonic appendage morphogenesis
GO:0005874	CELLULAR	238	6	0.4040	1	microtubule
GO:0055080	PROCESS	210	6	0.4042	1	cation homeostasis
GO:0035295	PROCESS	130	4	0.4044	1	tube development
GO:0003012	PROCESS	153	5	0.4054	1	muscle system process
GO:0007215	PROCESS	22	2	0.4068	1	glutamate signaling pathway

GO:0016298	FUNCTION	95	3	0.4076	1	lipase activity
GO:0045182	FUNCTION	106	2	0.4106	1	translation regulator activity
GO:0044242	PROCESS	80	2	0.4106	1	cellular lipid catabolic process
GO:0051338	PROCESS	294	7	0.4110	1	regulation of transferase activity
GO:0007417	PROCESS	293	11	0.4128	1	central nervous system development
GO:0006814	PROCESS	120	5	0.4138	1	sodium ion transport
GO:0010557	PROCESS	513	12	0.4142	1	positive regulation of macromolecule biosynthetic process
GO:0043232	CELLULAR	2233	44	0.4166	1	intracellular non-membrane-bounded organelle
GO:0043228	CELLULAR	2233	44	0.4166	1	non-membrane-bounded organelle
GO:0048518	PROCESS	1530	33	0.4182	1	positive regulation of biological process
GO:0006022	PROCESS	57	2	0.4186	1	aminoglycan metabolic process
GO:0005635	CELLULAR	176	4	0.4192	1	nuclear envelope
GO:0009056	PROCESS	1485	28	0.4196	1	catabolic process
GO:0009205	PROCESS	100	3	0.4198	1	purine ribonucleoside triphosphate metabolic process
GO:0032990	PROCESS	144	7	0.4198	1	cell part morphogenesis
GO:0009968	PROCESS	196	5	0.4202	1	negative regulation of signal transduction
GO:0015631	FUNCTION	72	2	0.4202	1	tubulin binding
GO:0015672	PROCESS	291	10	0.4206	1	monovalent inorganic cation transport
GO:0051726	PROCESS	270	5	0.4210	1	regulation of cell cycle
GO:0032502	PROCESS	2536	63	0.4216	1	developmental process
GO:0022008	PROCESS	391	13	0.4224	1	neurogenesis
GO:0009199	PROCESS	101	3	0.4226	1	ribonucleoside triphosphate metabolic process
GO:0019941	PROCESS	517	9	0.4230	1	modification-dependent protein catabolic process
GO:0043632	PROCESS	517	9	0.4230	1	modification-dependent macromolecule catabolic process
GO:0048869	PROCESS	1285	33	0.4238	1	cellular developmental process
GO:0006898	PROCESS	44	2	0.4238	1	receptor-mediated endocytosis
GO:0009144	PROCESS	101	3	0.4240	1	purine nucleoside triphosphate metabolic process
GO:0045761	PROCESS	79	3	0.4248	1	regulation of adenylate cyclase activity
GO:0009893	PROCESS	680	16	0.4264	1	positive regulation of metabolic process
GO:0009653	PROCESS	843	23	0.4268	1	anatomical structure morphogenesis
GO:0048747	PROCESS	47	2	0.4268	1	muscle fiber development
GO:0051603	PROCESS	521	9	0.4272	1	proteolysis involved in cellular protein catabolic process
GO:0051329	PROCESS	81	2	0.4276	1	interphase of mitotic cell cycle
GO:0044257	PROCESS	523	9	0.4292	1	cellular protein catabolic process
GO:0006916	PROCESS	172	4	0.4302	1	anti-apoptosis
GO:0016043	PROCESS	1989	45	0.4326	1	cellular component organization
GO:0030154	PROCESS	1244	32	0.4332	1	cell differentiation
GO:0051716	PROCESS	603	12	0.4338	1	cellular response to stimulus
GO:0030003	PROCESS	183	5	0.4350	1	cellular cation homeostasis
GO:0042734	CELLULAR	16	2	0.4354	1	presynaptic membrane
GO:0005783	CELLULAR	866	18	0.4360	1	endoplasmic reticulum
GO:0031279	PROCESS	81	3	0.4368	1	regulation of cyclase activity
GO:0031982	CELLULAR	553	13	0.4378	1	vesicle
GO:0030695	FUNCTION	362	13	0.4384	1	GTPase regulator activity
GO:0051641	PROCESS	761	17	0.4400	1	cellular localization
GO:0008104	PROCESS	771	16	0.4400	1	protein localization
GO:0080134	PROCESS	194	4	0.4404	1	regulation of response to stress
GO:0051339	PROCESS	83	3	0.4436	1	regulation of lyase activity
GO:0000122	PROCESS	205	5	0.4436	1	negative regulation of transcription from RNA polymerase II promoter
GO:0005681	CELLULAR	123	2	0.4438	1	spliceosomal complex
GO:0016604	CELLULAR	150	3	0.4448	1	nuclear body
GO:0016607	CELLULAR	95	2	0.4452	1	nuclear speck
GO:0030425	CELLULAR	77	3	0.4464	1	dendrite
GO:0060589	FUNCTION	371	13	0.4482	1	nucleoside-triphosphatase regulator activity
GO:0009141	PROCESS	107	3	0.4492	1	nucleoside triphosphate metabolic process
GO:0008168	FUNCTION	159	3	0.4496	1	methyltransferase activity
GO:0015075	FUNCTION	647	22	0.4504	1	ion transmembrane transporter activity
GO:0007586	PROCESS	78	2	0.4506	1	digestion
GO:0010604	PROCESS	646	15	0.4516	1	positive regulation of macromolecule metabolic process
GO:0007612	PROCESS	35	2	0.4516	1	learning
GO:0008066	FUNCTION	29	3	0.4516	1	glutamate receptor activity
GO:0004888	FUNCTION	1113	26	0.4518	1	transmembrane receptor activity
GO:0051325	PROCESS	84	2	0.4520	1	interphase
GO:0043285	PROCESS	1075	20	0.4522	1	biopolymer catabolic process
GO:0050863	PROCESS	84	2	0.4524	1	regulation of T cell activation
GO:0008360	PROCESS	48	2	0.4532	1	regulation of cell shape
GO:0016741	FUNCTION	161	3	0.4542	1	transferase activity, transferring one-carbon groups
GO:0031968	CELLULAR	91	2	0.4574	1	organelle outer membrane
GO:0016192	PROCESS	483	12	0.4582	1	vesicle-mediated transport
GO:0007005	PROCESS	104	2	0.4596	1	mitochondrion organization

GO:0016782	FUNCTION	54	2	0.4598	1	transferase activity, transferring sulfur-containing groups
GO:0048468	PROCESS	586	17	0.4624	1	cell development
GO:0006399	PROCESS	114	2	0.4634	1	tRNA metabolic process
GO:0005088	FUNCTION	78	4	0.4634	1	Ras guanyl-nucleotide exchange factor activity
GO:0019219	PROCESS	2511	44	0.4638	1	regulation of nucleobase, nucleoside, nucleotide and nucleic acid metabolic process
GO:0010564	PROCESS	90	2	0.4646	1	regulation of cell cycle process
GO:0016529	CELLULAR	32	2	0.4650	1	sarcoplasmic reticulum
GO:0007243	PROCESS	292	7	0.4652	1	protein kinase cascade
GO:0000228	CELLULAR	114	2	0.4656	1	nuclear chromosome
GO:0005198	FUNCTION	554	11	0.4666	1	structural molecule activity
GO:0033554	PROCESS	442	8	0.4678	1	cellular response to stress
GO:0032989	PROCESS	245	9	0.4698	1	cellular component morphogenesis
GO:0016568	PROCESS	246	5	0.4704	1	chromatin modification
GO:0010720	PROCESS	48	2	0.4706	1	positive regulation of cell development
GO:0019752	PROCESS	496	10	0.4716	1	carboxylic acid metabolic process
GO:0043436	PROCESS	496	10	0.4716	1	oxoacid metabolic process
GO:0007608	PROCESS	360	4	0.4718	1	sensory perception of smell
GO:0005840	CELLULAR	182	2	0.4720	1	ribosome
GO:0030902	PROCESS	41	2	0.4722	1	hindbrain development
GO:0016564	FUNCTION	262	6	0.4726	1	transcription repressor activity
GO:0006082	PROCESS	499	10	0.4748	1	organic acid metabolic process
GO:0019932	PROCESS	199	5	0.4760	1	second-messenger-mediated signaling
GO:0004984	FUNCTION	343	3	0.4766	1	olfactory receptor activity
GO:0016528	CELLULAR	33	2	0.4778	1	sarcoplasm
GO:0010605	PROCESS	588	12	0.4786	1	negative regulation of macromolecule metabolic process
GO:0008076	CELLULAR	80	3	0.4790	1	voltage-gated potassium channel complex
GO:0034705	CELLULAR	80	3	0.4790	1	potassium channel complex
GO:0031326	PROCESS	2636	46	0.4792	1	regulation of cellular biosynthetic process
GO:0030817	PROCESS	83	3	0.4808	1	regulation of cAMP biosynthetic process
GO:0000904	PROCESS	157	7	0.4808	1	cell morphogenesis involved in differentiation
GO:0015662	FUNCTION	44	2	0.4810	1	ATPase activity, coupled to transmembrane movement of ions, phosphorylative mechanism
GO:0034704	CELLULAR	26	2	0.4818	1	calcium channel complex
GO:0019867	CELLULAR	94	2	0.4822	1	outer membrane
GO:0031981	CELLULAR	1293	20	0.4828	1	nuclear lumen
GO:0005506	FUNCTION	277	6	0.4848	1	iron ion binding
GO:0003001	PROCESS	54	2	0.4860	1	generation of a signal involved in cell-cell signaling
GO:0007409	PROCESS	122	6	0.4860	1	axonogenesis
GO:0051171	PROCESS	2528	44	0.4868	1	regulation of nitrogen compound metabolic process
GO:0009889	PROCESS	2644	46	0.4870	1	regulation of biosynthetic process
GO:0005496	FUNCTION	112	3	0.4874	1	steroid binding
GO:0006875	PROCESS	136	4	0.4892	1	cellular metal ion homeostasis
GO:0006937	PROCESS	54	2	0.4906	1	regulation of muscle contraction
GO:0003682	FUNCTION	104	2	0.4924	1	chromatin binding
GO:0006665	PROCESS	67	2	0.4932	1	sphingolipid metabolic process
GO:0045941	PROCESS	440	10	0.4938	1	positive regulation of transcription
GO:0042180	PROCESS	506	10	0.4944	1	cellular ketone metabolic process
GO:0007517	PROCESS	175	5	0.4968	1	muscle organ development
GO:0006873	PROCESS	274	7	0.4970	1	cellular ion homeostasis
GO:0034703	CELLULAR	123	5	0.4974	1	cation channel complex
GO:0004713	FUNCTION	147	5	0.4982	1	protein tyrosine kinase activity
GO:0003713	FUNCTION	195	4	0.4992	1	transcription coactivator activity
GO:0048583	PROCESS	332	7	0.5014	1	regulation of response to stimulus
GO:0019904	FUNCTION	253	7	0.5020	1	protein domain specific binding
GO:0007167	PROCESS	283	9	0.5024	1	enzyme linked receptor protein signaling pathway
GO:0030234	FUNCTION	755	20	0.5026	1	enzyme regulator activity
GO:0008285	PROCESS	289	7	0.5034	1	negative regulation of cell proliferation
GO:0003676	FUNCTION	2951	46	0.5036	1	nucleic acid binding
GO:0005925	CELLULAR	89	3	0.5038	1	focal adhesion
GO:0043406	PROCESS	82	2	0.5040	1	positive regulation of MAP kinase activity
GO:0051276	PROCESS	422	7	0.5048	1	chromosome organization
GO:0043176	FUNCTION	80	2	0.5050	1	amine binding
GO:0008236	FUNCTION	175	4	0.5052	1	serine-type peptidase activity
GO:0050776	PROCESS	151	3	0.5066	1	regulation of immune response
GO:0031420	FUNCTION	222	8	0.5066	1	alkali metal ion binding
GO:0030814	PROCESS	86	3	0.5088	1	regulation of cAMP metabolic process
GO:0006812	PROCESS	494	16	0.5102	1	cation transport
GO:0048870	PROCESS	223	7	0.5106	1	cell motility
GO:0017171	FUNCTION	177	4	0.5114	1	serine hydrolase activity
GO:0009617	PROCESS	124	2	0.5116	1	response to bacterium

GO:0016323	CELLULAR	150	5	0.5118	1	basolateral plasma membrane
GO:0030529	CELLULAR	442	5	0.5130	1	ribonucleoprotein complex
GO:0001505	PROCESS	52	2	0.5132	1	regulation of neurotransmitter levels
GO:0031967	CELLULAR	519	8	0.5138	1	organelle envelope
GO:0055082	PROCESS	280	7	0.5140	1	cellular chemical homeostasis
GO:0007420	PROCESS	184	6	0.5154	1	brain development
GO:0051249	PROCESS	102	2	0.5164	1	regulation of lymphocyte activation
GO:0048646	PROCESS	245	7	0.5166	1	anatomical structure formation involved in morphogenesis
GO:0031975	CELLULAR	521	8	0.5178	1	envelope
GO:0000166	FUNCTION	2036	45	0.5182	1	nucleotide binding
GO:0035108	PROCESS	63	2	0.5200	1	limb morphogenesis
GO:0035107	PROCESS	63	2	0.5200	1	appendage morphogenesis
GO:0055065	PROCESS	143	4	0.5216	1	metal ion homeostasis
GO:0006886	PROCESS	323	6	0.5224	1	intracellular protein transport
GO:0034621	PROCESS	298	4	0.5226	1	cellular macromolecular complex subunit organization
GO:0048584	PROCESS	167	4	0.5228	1	positive regulation of response to stimulus
GO:0031347	PROCESS	93	2	0.5250	1	regulation of defense response
GO:0005887	CELLULAR	1096	31	0.5256	1	integral to plasma membrane
GO:0031667	PROCESS	102	2	0.5290	1	response to nutrient levels
GO:0031410	CELLULAR	544	12	0.5300	1	cytoplasmic vesicle
GO:0009725	PROCESS	185	4	0.5308	1	response to hormone stimulus
GO:0045449	PROCESS	2335	39	0.5310	1	regulation of transcription
GO:0005886	CELLULAR	3366	87	0.5312	1	plasma membrane
GO:0006643	PROCESS	73	2	0.5330	1	membrane lipid metabolic process
GO:0031225	CELLULAR	209	7	0.5336	1	anchored to membrane
GO:0048666	PROCESS	210	8	0.5360	1	neuron development
GO:0006508	PROCESS	927	17	0.5362	1	proteolysis
GO:0009894	PROCESS	92	2	0.5372	1	regulation of catabolic process
GO:0010556	PROCESS	2537	42	0.5374	1	regulation of macromolecule biosynthetic process
GO:0015077	FUNCTION	88	2	0.5378	1	monovalent inorganic cation transmembrane transporter activity
GO:0060173	PROCESS	65	2	0.5392	1	limb development
GO:0048736	PROCESS	65	2	0.5392	1	appendage development
GO:0044427	CELLULAR	314	4	0.5416	1	chromosomal part
GO:0030198	PROCESS	69	2	0.5418	1	extracellular matrix organization
GO:0044446	CELLULAR	3679	61	0.5424	1	intracellular organelle part
GO:0030163	PROCESS	937	17	0.5428	1	protein catabolic process
GO:0005654	CELLULAR	777	12	0.5428	1	nucleoplasm
GO:0008022	FUNCTION	114	3	0.5430	1	protein C-terminus binding
GO:0042625	FUNCTION	61	2	0.5434	1	ATPase activity, coupled to transmembrane movement of ions
GO:0046903	PROCESS	216	6	0.5444	1	secretion
GO:0048522	PROCESS	1405	29	0.5470	1	positive regulation of cellular process
GO:0005924	CELLULAR	93	3	0.5476	1	cell-substrate adherens junction
GO:0044057	PROCESS	190	6	0.5520	1	regulation of system process
GO:0017038	PROCESS	108	2	0.5526	1	protein import
GO:0043066	PROCESS	271	5	0.5532	1	negative regulation of apoptosis
GO:0000902	PROCESS	217	8	0.5542	1	cell morphogenesis
GO:0003707	FUNCTION	47	2	0.5552	1	steroid hormone receptor activity
GO:0016829	FUNCTION	139	3	0.5554	1	lyase activity
GO:0043566	FUNCTION	104	2	0.5576	1	structure-specific DNA binding
GO:0051241	PROCESS	100	2	0.5582	1	negative regulation of multicellular organismal process
GO:0045211	CELLULAR	118	6	0.5584	1	postsynaptic membrane
GO:0010033	PROCESS	416	9	0.5624	1	response to organic substance
GO:0043062	PROCESS	104	3	0.5628	1	extracellular structure organization
GO:0016567	PROCESS	96	2	0.5630	1	protein ubiquitination
GO:0005739	CELLULAR	948	14	0.5636	1	mitochondrion
GO:0042221	PROCESS	866	18	0.5640	1	response to chemical stimulus
GO:0006813	PROCESS	146	5	0.5644	1	potassium ion transport
GO:0005875	CELLULAR	86	2	0.5654	1	microtubule associated complex
GO:0030055	CELLULAR	96	3	0.5676	1	cell-substrate junction
GO:0019216	PROCESS	85	2	0.5718	1	regulation of lipid metabolic process
GO:0005231	FUNCTION	46	2	0.5720	1	excitatory extracellular ligand-gated ion channel activity
GO:0006091	PROCESS	267	4	0.5734	1	generation of precursor metabolites and energy
GO:0019220	PROCESS	386	8	0.5742	1	regulation of phosphate metabolic process
GO:0051174	PROCESS	386	8	0.5742	1	regulation of phosphorus metabolic process
GO:0016879	FUNCTION	203	4	0.5748	1	ligase activity, forming carbon-nitrogen bonds
GO:0044087	PROCESS	108	2	0.5748	1	regulation of cellular component biogenesis
GO:0051704	PROCESS	548	9	0.5752	1	multi-organism process
GO:0008037	PROCESS	47	3	0.5784	1	cell recognition
GO:0008083	FUNCTION	151	3	0.5824	1	growth factor activity
GO:0045937	PROCESS	85	2	0.5826	1	positive regulation of phosphate metabolic process
GO:0010562	PROCESS	85	2	0.5826	1	positive regulation of phosphorus metabolic process

GO:0006470	PROCESS	119	4	0.5838	1	protein amino acid dephosphorylation
GO:0031226	CELLULAR	1120	31	0.5846	1	intrinsic to plasma membrane
GO:0043069	PROCESS	277	5	0.5870	1	negative regulation of programmed cell death
GO:0060548	PROCESS	277	5	0.5870	1	negative regulation of cell death
GO:0005792	CELLULAR	174	3	0.5874	1	microsome
GO:0004930	FUNCTION	740	11	0.5874	1	G-protein coupled receptor activity
GO:0006357	PROCESS	593	11	0.5876	1	regulation of transcription from RNA polymerase II promoter
GO:0016021	CELLULAR	4828	108	0.5882	1	integral to membrane
GO:0007204	PROCESS	76	2	0.5884	1	elevation of cytosolic calcium ion concentration
GO:0045944	PROCESS	273	6	0.5908	1	positive regulation of transcription from RNA polymerase II promoter
GO:0033365	PROCESS	119	2	0.5918	1	protein localization in organelle
GO:0005730	CELLULAR	627	9	0.5922	1	nucleolus
GO:0032446	PROCESS	107	2	0.5926	1	protein modification by small protein conjugation
GO:0005267	FUNCTION	124	4	0.5932	1	potassium channel activity
GO:0007268	PROCESS	235	8	0.5940	1	synaptic transmission
GO:0030141	CELLULAR	139	3	0.5954	1	secretory granule
GO:0008092	FUNCTION	431	12	0.5964	1	cytoskeletal protein binding
GO:0019725	PROCESS	356	8	0.5968	1	cellular homeostasis
GO:0046983	FUNCTION	421	10	0.5972	1	protein dimerization activity
GO:0002520	PROCESS	194	4	0.5986	1	immune system development
GO:0051056	PROCESS	226	7	0.6000	1	regulation of small GTPase mediated signal transduction
GO:0010628	PROCESS	455	10	0.6006	1	positive regulation of gene expression
GO:0051480	PROCESS	80	2	0.6018	1	cytosolic calcium ion homeostasis
GO:0009607	PROCESS	301	4	0.6032	1	response to biotic stimulus
GO:0008283	PROCESS	302	6	0.6038	1	cell proliferation
GO:0008081	FUNCTION	81	3	0.6048	1	phosphoric diester hydrolase activity
GO:0042692	PROCESS	66	2	0.6054	1	muscle cell differentiation
GO:0019717	CELLULAR	48	2	0.6060	1	synaptosome
GO:0032870	PROCESS	95	2	0.6064	1	cellular response to hormone stimulus
GO:0016044	PROCESS	313	7	0.6064	1	membrane organization
GO:0016705	FUNCTION	119	2	0.6086	1	oxidoreductase activity, acting on paired donors, with incorporation or reduction of molecular oxygen
GO:0003774	FUNCTION	135	4	0.6108	1	motor activity
GO:0004879	FUNCTION	57	2	0.6110	1	ligand-dependent nuclear receptor activity
GO:0032940	PROCESS	147	4	0.6116	1	secretion by cell
GO:0003712	FUNCTION	325	6	0.6128	1	transcription cofactor activity
GO:0030030	PROCESS	238	9	0.6132	1	cell projection organization
GO:0007600	PROCESS	700	11	0.6156	1	sensory perception
GO:0034613	PROCESS	355	6	0.6184	1	cellular protein localization
GO:0042598	CELLULAR	179	3	0.6188	1	vesicular fraction
GO:0065008	PROCESS	1108	24	0.6190	1	regulation of biological quality
GO:0006325	PROCESS	331	5	0.6190	1	chromatin organization
GO:0005624	CELLULAR	611	15	0.6202	1	membrane fraction
GO:0016071	PROCESS	334	5	0.6204	1	mRNA metabolic process
GO:0009605	PROCESS	677	13	0.6210	1	response to external stimulus
GO:0030182	PROCESS	282	9	0.6222	1	neuron differentiation
GO:0005578	CELLULAR	290	9	0.6230	1	proteinaceous extracellular matrix
GO:0045893	PROCESS	378	8	0.6254	1	positive regulation of transcription, DNA-dependent
GO:0080090	PROCESS	2937	49	0.6260	1	regulation of primary metabolic process
GO:0019318	PROCESS	133	2	0.6296	1	hexose metabolic process
GO:0034702	CELLULAR	187	6	0.6304	1	ion channel complex
GO:0009897	CELLULAR	114	4	0.6332	1	external side of plasma membrane
GO:0051254	PROCESS	381	8	0.6336	1	positive regulation of RNA metabolic process
GO:0030900	PROCESS	88	2	0.6336	1	forebrain development
GO:0032991	CELLULAR	2668	45	0.6348	1	macromolecular complex
GO:0012506	CELLULAR	120	3	0.6368	1	vesicle membrane
GO:0005976	PROCESS	97	2	0.6374	1	polysaccharide metabolic process
GO:0070727	PROCESS	358	6	0.6378	1	cellular macromolecule localization
GO:0046483	PROCESS	280	6	0.6396	1	heterocycle metabolic process
GO:0016311	PROCESS	134	4	0.6396	1	dephosphorylation
GO:0008238	FUNCTION	72	2	0.6402	1	exopeptidase activity
GO:0045296	FUNCTION	13	2	0.6402	1	cadherin binding
GO:0055114	PROCESS	569	10	0.6408	1	oxidation reduction
GO:0007611	PROCESS	63	2	0.6412	1	learning or memory
GO:0022402	PROCESS	505	7	0.6424	1	cell cycle process
GO:0007267	PROCESS	497	12	0.6450	1	cell-cell signaling
GO:0048513	PROCESS	1318	30	0.6452	1	organ development
GO:0031966	CELLULAR	325	4	0.6452	1	mitochondrial membrane
GO:0020037	FUNCTION	111	2	0.6454	1	heme binding
GO:0045597	PROCESS	169	4	0.6456	1	positive regulation of cell differentiation

GO:0030955	FUNCTION	124	4	0.6464	1	potassium ion binding
GO:0048731	PROCESS	1802	44	0.6468	1	system development
GO:0048514	PROCESS	153	4	0.6494	1	blood vessel morphogenesis
GO:0007601	PROCESS	205	5	0.6500	1	visual perception
GO:0050953	PROCESS	205	5	0.6500	1	sensory perception of light stimulus
GO:0009790	PROCESS	405	8	0.6506	1	embryonic development
GO:0008270	FUNCTION	2119	38	0.6532	1	zinc ion binding
GO:0005126	FUNCTION	171	2	0.6538	1	cytokine receptor binding
GO:0048037	FUNCTION	213	4	0.6542	1	cofactor binding
GO:0042325	PROCESS	372	7	0.6568	1	regulation of phosphorylation
GO:0010740	PROCESS	145	2	0.6612	1	positive regulation of protein kinase cascade
GO:0051239	PROCESS	689	15	0.6614	1	regulation of multicellular organismal process
GO:0031323	PROCESS	3075	51	0.6626	1	regulation of cellular metabolic process
GO:0050878	PROCESS	109	2	0.6642	1	regulation of body fluid levels
GO:0000267	CELLULAR	794	18	0.6646	1	cell fraction
GO:0006397	PROCESS	291	4	0.6650	1	mRNA processing
GO:0016881	FUNCTION	176	3	0.6660	1	acid-amino acid ligase activity
GO:0050662	FUNCTION	153	3	0.6662	1	coenzyme binding
GO:0007346	PROCESS	138	2	0.6666	1	regulation of mitotic cell cycle
GO:0005201	FUNCTION	76	2	0.6682	1	extracellular matrix structural constituent
GO:0019898	CELLULAR	460	12	0.6684	1	extrinsic to membrane
GO:0010468	PROCESS	2571	41	0.6696	1	regulation of gene expression
GO:0006631	PROCESS	179	3	0.6698	1	fatty acid metabolic process
GO:0050890	PROCESS	757	12	0.6714	1	cognition
GO:0031401	PROCESS	101	2	0.6738	1	positive regulation of protein modification process
GO:0065003	PROCESS	461	7	0.6744	1	macromolecular complex assembly
GO:0040008	PROCESS	243	4	0.6746	1	regulation of growth
GO:0004721	FUNCTION	150	4	0.6748	1	phosphoprotein phosphatase activity
GO:0019901	FUNCTION	104	2	0.6752	1	protein kinase binding
GO:0003008	PROCESS	1263	25	0.6766	1	system process
GO:0031012	CELLULAR	308	9	0.6766	1	extracellular matrix
GO:0070161	CELLULAR	149	5	0.6778	1	anchoring junction
GO:0006807	PROCESS	1878	30	0.6786	1	nitrogen compound metabolic process
GO:0050839	FUNCTION	21	2	0.6816	1	cell adhesion molecule binding
GO:0042127	PROCESS	604	11	0.6834	1	regulation of cell proliferation
GO:0005626	CELLULAR	626	15	0.6836	1	insoluble fraction
GO:0043933	PROCESS	508	8	0.6852	1	macromolecular complex subunit organization
GO:0005740	CELLULAR	346	4	0.6870	1	mitochondrial envelope
GO:0046873	FUNCTION	296	10	0.6874	1	metal ion transmembrane transporter activity
GO:0008361	PROCESS	131	2	0.6880	1	regulation of cell size
GO:0007186	PROCESS	945	14	0.6882	1	G-protein coupled receptor protein signaling pathway
GO:0070647	PROCESS	132	2	0.6916	1	protein modification by small protein conjugation or removal
GO:0042626	FUNCTION	93	3	0.6946	1	ATPase activity, coupled to transmembrane movement of substances
GO:0042623	FUNCTION	238	5	0.6946	1	ATPase activity, coupled
GO:0019899	FUNCTION	404	9	0.6950	1	enzyme binding
GO:0031988	CELLULAR	466	9	0.6964	1	membrane-bounded vesicle
GO:0043492	FUNCTION	94	3	0.6986	1	ATPase activity, coupled to movement of substances
GO:0050804	PROCESS	65	2	0.6996	1	regulation of synaptic transmission
GO:0046906	FUNCTION	119	2	0.6996	1	tetrapyrrole binding
GO:0032269	PROCESS	125	2	0.7000	1	negative regulation of cellular protein metabolic process
GO:0003002	PROCESS	146	2	0.7000	1	regionalization
GO:0016820	FUNCTION	95	3	0.7002	1	hydrolase activity, acting on acid anhydrides, catalyzing transmembrane movement of substances
GO:0015629	CELLULAR	224	6	0.7010	1	actin cytoskeleton
GO:0008544	PROCESS	153	2	0.7030	1	epidermis development
GO:0030097	PROCESS	163	3	0.7030	1	hemopoiesis
GO:0046943	FUNCTION	80	2	0.7030	1	carboxylic acid transmembrane transporter activity
GO:0001568	PROCESS	172	4	0.7036	1	blood vessel development
GO:0016477	PROCESS	196	5	0.7038	1	cell migration
GO:0005342	FUNCTION	81	2	0.7046	1	organic acid transmembrane transporter activity
GO:0045596	PROCESS	157	3	0.7050	1	negative regulation of cell differentiation
GO:0030001	PROCESS	410	13	0.7060	1	metal ion transport
GO:0004252	FUNCTION	151	2	0.7070	1	serine-type endopeptidase activity
GO:0006355	PROCESS	1567	24	0.7084	1	regulation of transcription, DNA-dependent
GO:0034984	PROCESS	299	4	0.7092	1	cellular response to DNA damage stimulus
GO:0015405	FUNCTION	104	3	0.7104	1	P-P-bond-hydrolysis-driven transmembrane transporter activity
GO:0015399	FUNCTION	104	3	0.7104	1	primary active transmembrane transporter activity
GO:0001944	PROCESS	177	4	0.7130	1	vasculature development
GO:0007156	PROCESS	131	4	0.7132	1	homophilic cell adhesion
GO:0043169	FUNCTION	3870	83	0.7134	1	cation binding

GO:0019935	PROCESS	110	2	0.7136	1	cyclic-nucleotide-mediated signaling
GO:0050877	PROCESS	1007	19	0.7160	1	neurological system process
GO:0006259	PROCESS	443	6	0.7160	1	DNA metabolic process
GO:0046914	FUNCTION	2555	46	0.7162	1	transition metal ion binding
GO:0019838	FUNCTION	89	2	0.7164	1	growth factor binding
GO:0051969	PROCESS	72	2	0.7180	1	regulation of transmission of nerve impulse
GO:0009719	PROCESS	199	4	0.7204	1	response to endogenous stimulus
GO:0001701	PROCESS	115	2	0.7204	1	in utero embryonic development
GO:0043009	PROCESS	229	4	0.7206	1	chordate embryonic development
GO:0008380	PROCESS	255	3	0.7220	1	RNA splicing
GO:0040012	PROCESS	174	4	0.7222	1	regulation of locomotion
GO:0031644	PROCESS	75	2	0.7238	1	regulation of neurological system process
GO:0004842	FUNCTION	127	2	0.7238	1	ubiquitin-protein ligase activity
GO:0001653	FUNCTION	102	2	0.7242	1	peptide receptor activity
GO:0009792	PROCESS	231	4	0.7250	1	embryonic development ending in birth or egg hatching
GO:0007049	PROCESS	696	9	0.7278	1	cell cycle
GO:0017124	FUNCTION	83	2	0.7300	1	SH3 domain binding
GO:0018193	PROCESS	121	2	0.7304	1	peptidyl-amino acid modification
GO:0046872	FUNCTION	3836	82	0.7306	1	metal ion binding
GO:0003779	FUNCTION	288	8	0.7326	1	actin binding
GO:0005529	FUNCTION	170	3	0.7342	1	sugar binding
GO:0051128	PROCESS	361	7	0.7342	1	regulation of cellular component organization
GO:0007389	PROCESS	195	3	0.7346	1	pattern specification process
GO:0042391	PROCESS	89	2	0.7354	1	regulation of membrane potential
GO:0005230	FUNCTION	66	2	0.7360	1	extracellular ligand-gated ion channel activity
GO:0040007	PROCESS	121	2	0.7368	1	growth
GO:0060255	PROCESS	2924	46	0.7378	1	regulation of macromolecule metabolic process
GO:0051252	PROCESS	1598	24	0.7380	1	regulation of RNA metabolic process
GO:0034660	PROCESS	217	2	0.7388	1	ncRNA metabolic process
GO:0007160	PROCESS	79	2	0.7418	1	cell-matrix adhesion
GO:0048856	PROCESS	1938	45	0.7430	1	anatomical structure development
GO:0007398	PROCESS	167	2	0.7434	1	ectoderm development
GO:0051248	PROCESS	131	2	0.7450	1	negative regulation of protein metabolic process
GO:0005509	FUNCTION	864	26	0.7458	1	calcium ion binding
GO:0006520	PROCESS	213	3	0.7466	1	cellular amino acid metabolic process
GO:0044106	PROCESS	213	3	0.7466	1	cellular amine metabolic process
GO:0031399	PROCESS	178	3	0.7468	1	regulation of protein modification process
GO:0005099	FUNCTION	79	2	0.7468	1	Ras GTPase activator activity
GO:0040011	PROCESS	333	7	0.7480	1	locomotion
GO:0019226	PROCESS	276	8	0.7494	1	transmission of nerve impulse
GO:0019222	PROCESS	3211	52	0.7522	1	regulation of metabolic process
GO:0051246	PROCESS	466	7	0.7532	1	regulation of protein metabolic process
GO:0019787	FUNCTION	141	2	0.7534	1	small conjugating protein ligase activity
GO:0043233	CELLULAR	1633	22	0.7538	1	organelle lumen
GO:0032268	PROCESS	407	6	0.7542	1	regulation of cellular protein metabolic process
GO:0015276	FUNCTION	112	4	0.7548	1	ligand-gated ion channel activity
GO:0022834	FUNCTION	112	4	0.7548	1	ligand-gated channel activity
GO:0006974	PROCESS	329	4	0.7564	1	response to DNA damage stimulus
GO:0048534	PROCESS	183	3	0.7572	1	hemopoietic or lymphoid organ development
GO:0005912	CELLULAR	130	4	0.7582	1	adherens junction
GO:0044433	CELLULAR	154	3	0.7606	1	cytoplasmic vesicle part
GO:0005261	FUNCTION	252	8	0.7622	1	cation channel activity
GO:0010817	PROCESS	111	2	0.7636	1	regulation of hormone levels
GO:0016491	FUNCTION	623	10	0.7648	1	oxidoreductase activity
GO:0051020	FUNCTION	77	2	0.7654	1	GTPase binding
GO:0014069	CELLULAR	43	2	0.7660	1	postsynaptic density
GO:0010926	PROCESS	836	15	0.7676	1	anatomical structure formation
GO:0045121	CELLULAR	82	2	0.7676	1	membrane raft
GO:0006790	PROCESS	94	2	0.7676	1	sulfur metabolic process
GO:0044456	CELLULAR	181	7	0.7678	1	synapse part
GO:0010646	PROCESS	800	17	0.7680	1	regulation of cell communication
GO:0044463	CELLULAR	134	3	0.7710	1	cell projection part
GO:0043565	FUNCTION	523	8	0.7732	1	sequence-specific DNA binding
GO:0043167	FUNCTION	3920	83	0.7734	1	ion binding
GO:0031974	CELLULAR	1664	22	0.7744	1	membrane-enclosed lumen
GO:0006281	PROCESS	257	3	0.7756	1	DNA repair
GO:0070013	CELLULAR	1597	21	0.7766	1	intracellular organelle lumen
GO:0019900	FUNCTION	125	2	0.7770	1	kinase binding
GO:0022607	PROCESS	635	10	0.7784	1	cellular component assembly
GO:0003824	FUNCTION	4750	90	0.7856	1	catalytic activity
GO:0044451	CELLULAR	468	6	0.7860	1	nucleoplasm part

GO:0001558	PROCESS	133	2	0.7866	1	regulation of cell growth
GO:0005625	CELLULAR	199	3	0.7878	1	soluble fraction
GO:0005856	CELLULAR	1184	25	0.7878	1	cytoskeleton
GO:0043085	PROCESS	431	7	0.7886	1	positive regulation of catalytic activity
GO:0016887	FUNCTION	299	6	0.7896	1	ATPase activity
GO:0006461	PROCESS	323	5	0.7928	1	protein complex assembly
GO:0070271	PROCESS	323	5	0.7928	1	protein complex biogenesis
GO:0001932	PROCESS	136	2	0.7942	1	regulation of protein amino acid phosphorylation
GO:0005096	FUNCTION	202	5	0.7946	1	GTPase activator activity
GO:0005249	FUNCTION	94	2	0.7958	1	voltage-gated potassium channel activity
GO:0090066	PROCESS	222	3	0.7964	1	regulation of anatomical structure size
GO:0031589	PROCESS	89	2	0.7968	1	cell-substrate adhesion
GO:0005539	FUNCTION	122	2	0.7992	1	glycosaminoglycan binding
GO:0070887	PROCESS	196	3	0.8008	1	cellular response to chemical stimulus
GO:0034641	PROCESS	224	3	0.8010	1	cellular nitrogen compound metabolic process
GO:0001775	PROCESS	201	3	0.8020	1	cell activation
GO:0007605	PROCESS	80	2	0.8030	1	sensory perception of sound
GO:0030136	CELLULAR	101	2	0.8036	1	clathrin-coated vesicle
GO:0006350	PROCESS	267	3	0.8040	1	transcription
GO:0008415	FUNCTION	177	2	0.8056	1	acyltransferase activity
GO:0045860	PROCESS	171	3	0.8072	1	positive regulation of protein kinase activity
GO:0004714	FUNCTION	64	2	0.8080	1	transmembrane receptor protein tyrosine kinase activity
GO:0016023	CELLULAR	460	8	0.8084	1	cytoplasmic membrane-bounded vesicle
GO:0033674	PROCESS	172	3	0.8094	1	positive regulation of kinase activity
GO:0006366	PROCESS	175	2	0.8154	1	transcription from RNA polymerase II promoter
GO:0016747	FUNCTION	179	2	0.8160	1	transferase activity, transferring acyl groups other than amino-acyl groups
GO:0042578	FUNCTION	313	7	0.8198	1	phosphoric ester hydrolase activity
GO:0042277	FUNCTION	169	3	0.8200	1	peptide binding
GO:0005743	CELLULAR	242	2	0.8218	1	mitochondrial inner membrane
GO:0030659	CELLULAR	112	2	0.8218	1	cytoplasmic vesicle membrane
GO:0051345	PROCESS	154	2	0.8218	1	positive regulation of hydrolase activity
GO:0005057	FUNCTION	153	3	0.8226	1	receptor signaling protein activity
GO:0016746	FUNCTION	187	2	0.8260	1	transferase activity, transferring acyl groups
GO:0009966	PROCESS	715	14	0.8260	1	regulation of signal transduction
GO:0030247	FUNCTION	130	2	0.8262	1	polysaccharide binding
GO:0001871	FUNCTION	130	2	0.8262	1	pattern binding
GO:0030334	PROCESS	159	3	0.8284	1	regulation of cell migration
GO:0051347	PROCESS	179	3	0.8284	1	positive regulation of transferase activity
GO:0015630	CELLULAR	465	7	0.8306	1	microtubule cytoskeleton
GO:0006811	PROCESS	689	18	0.8344	1	ion transport
GO:0043234	CELLULAR	2171	37	0.8370	1	protein complex
GO:0008610	PROCESS	290	4	0.8372	1	lipid biosynthetic process
GO:0050954	PROCESS	84	2	0.8374	1	sensory perception of mechanical stimulus
GO:0009308	PROCESS	350	5	0.8378	1	amine metabolic process
GO:0031252	CELLULAR	108	2	0.8478	1	cell leading edge
GO:0050790	PROCESS	710	11	0.8486	1	regulation of catalytic activity
GO:0030135	CELLULAR	126	2	0.8498	1	coated vesicle
GO:0019866	CELLULAR	258	2	0.8504	1	organelle inner membrane
GO:0044429	CELLULAR	516	5	0.8516	1	mitochondrial part
GO:0044085	PROCESS	738	10	0.8550	1	cellular component biogenesis
GO:0032535	PROCESS	184	2	0.8550	1	regulation of cellular component size
GO:0030054	CELLULAR	461	14	0.8584	1	cell junction
GO:0044459	CELLULAR	1960	51	0.8588	1	plasma membrane part
GO:0008284	PROCESS	306	4	0.8632	1	positive regulation of cell proliferation
GO:0022843	FUNCTION	134	3	0.8636	1	voltage-gated cation channel activity
GO:0016791	FUNCTION	231	4	0.8676	1	phosphatase activity
GO:0005911	CELLULAR	156	3	0.8678	1	cell-cell junction
GO:0060341	PROCESS	174	2	0.8698	1	regulation of cellular localization
GO:0001525	PROCESS	110	2	0.8710	1	angiogenesis
GO:0045202	CELLULAR	265	9	0.8726	1	synapse
GO:0044093	PROCESS	479	7	0.8728	1	positive regulation of molecular function
GO:0032561	FUNCTION	340	4	0.8760	1	guanyl ribonucleotide binding
GO:0019001	FUNCTION	340	4	0.8760	1	guanyl nucleotide binding
GO:0006396	PROCESS	504	5	0.8770	1	RNA processing
GO:0022838	FUNCTION	364	10	0.8788	1	substrate-specific channel activity
GO:0019199	FUNCTION	80	2	0.8796	1	transmembrane receptor protein kinase activity
GO:0008134	FUNCTION	450	6	0.8806	1	transcription factor binding
GO:0016070	PROCESS	825	9	0.8808	1	RNA metabolic process
GO:0051270	PROCESS	175	3	0.8820	1	regulation of cell motion
GO:0015267	FUNCTION	372	10	0.8830	1	channel activity

GO:0022803	FUNCTION	372	10	0.8830	1	passive transmembrane transporter activity
GO:0022836	FUNCTION	283	8	0.8836	1	gated channel activity
GO:0008047	FUNCTION	302	6	0.8842	1	enzyme activator activity
GO:0051049	PROCESS	318	5	0.8880	1	regulation of transport
GO:0005244	FUNCTION	177	4	0.8896	1	voltage-gated ion channel activity
GO:0022832	FUNCTION	177	4	0.8896	1	voltage-gated channel activity
GO:0006351	PROCESS	225	2	0.8898	1	transcription, DNA-dependent
GO:0048471	CELLULAR	220	3	0.8914	1	perinuclear region of cytoplasm
GO:0032774	PROCESS	229	2	0.8928	1	RNA biosynthetic process
GO:0005813	CELLULAR	191	2	0.8966	1	centrosome
GO:0010627	PROCESS	225	2	0.8980	1	regulation of protein kinase cascade
GO:0007155	PROCESS	647	19	0.9026	1	cell adhesion
GO:0022610	PROCESS	648	19	0.9026	1	biological adhesion
GO:0042995	CELLULAR	464	11	0.9034	1	cell projection
GO:0065009	PROCESS	811	12	0.9056	1	regulation of molecular function
GO:0004725	FUNCTION	92	2	0.9074	1	protein tyrosine phosphatase activity
GO:0044430	CELLULAR	795	12	0.9130	1	cytoskeletal part
GO:0009898	CELLULAR	291	4	0.9164	1	internal side of plasma membrane
GO:0004175	FUNCTION	355	4	0.9172	1	endopeptidase activity
GO:0008509	FUNCTION	131	2	0.9178	1	anion transmembrane transporter activity
GO:0032879	PROCESS	486	8	0.9190	1	regulation of localization
GO:0030246	FUNCTION	301	4	0.9204	1	carbohydrate binding
GO:0005525	FUNCTION	331	3	0.9224	1	GTP binding
GO:0006816	PROCESS	112	3	0.9230	1	calcium ion transport
GO:0009986	CELLULAR	247	5	0.9236	1	cell surface
GO:0070838	PROCESS	113	3	0.9238	1	divalent metal ion transport
GO:0005216	FUNCTION	354	9	0.9260	1	ion channel activity
GO:0070011	FUNCTION	515	7	0.9274	1	peptidase activity, acting on L-amino acid peptides
GO:0005815	CELLULAR	213	2	0.9298	1	microtubule organizing center
GO:0005262	FUNCTION	73	2	0.9320	1	calcium channel activity
GO:0008233	FUNCTION	531	7	0.9337	1	peptidase activity
GO:0006519	PROCESS	315	3	0.9409	1	cellular amino acid and derivative metabolic process
GO:0015674	PROCESS	141	3	0.9421	1	di-, tri-valent inorganic cation transport
GO:0044419	PROCESS	264	2	0.9427	1	interspecies interaction between organisms
GO:0016788	FUNCTION	638	9	0.9443	1	hydrolase activity, acting on ester bonds
GO:0005516	FUNCTION	129	2	0.9527	1	calmodulin binding
GO:0016462	FUNCTION	680	10	0.9545	1	pyrophosphatase activity
GO:0009967	PROCESS	241	2	0.9549	1	positive regulation of signal transduction
GO:0016818	FUNCTION	683	10	0.9567	1	hydrolase activity, acting on acid anhydrides, in phosphorus-containing anhydrides
GO:0008237	FUNCTION	172	2	0.9573	1	metallopeptidase activity
GO:0016817	FUNCTION	687	10	0.9583	1	hydrolase activity, acting on acid anhydrides
GO:0017111	FUNCTION	653	9	0.9597	1	nucleoside-triphosphatase activity
GO:0007626	PROCESS	216	2	0.9681	1	locomotory behavior
GO:0010647	PROCESS	253	2	0.9719	1	positive regulation of cell communication
GO:0007610	PROCESS	348	4	0.9731	1	behavior
GO:0001501	PROCESS	243	2	0.9733	1	skeletal system development
GO:0043005	CELLULAR	171	3	0.9767	1	neuron projection
GO:0051336	PROCESS	287	2	0.9827	1	regulation of hydrolase activity
GO:0003723	FUNCTION	653	5	0.9845	1	RNA binding
GO:0016787	FUNCTION	2089	31	0.9905	1	hydrolase activity
GO:0016337	PROCESS	256	4	0.9907	1	cell-cell adhesion
