# Supplementary material for: Genetic Evidence Implicates the Immune System and Cholesterol Metabolism in the Aetiology of Alzheimer's Disease
Source: PLoS One. 2010 Nov 15;5(11):e13950. doi: 10.1371/journal.pone.0013950 (PMC2981526; doi:10.1371/journal.pone.0013950)
Supplement: Table S3 — List of KEGG categories significantly (p<0.05) enriched in both GWAS. “Joint p” is the probability of observing by chance at least one category among the entire set of categories tested with joint enrichment (defined as the product of enrichment p-values from the two GWAS) at least as extreme as that observed in the real data. This corrects for the multiple non-independent GO categories being tested. (0.00 MB PDF) [file pone.0013950.s003.pdf]

Table S3 List of KEGG categories significantly ($p<0.05$) enriched in both GWAS

KEGG pathway	p-val (Harold)	p-val (Lambert)	Joint p	Function
5310	0.0010	0.0026	0.0016	Asthma
4640	0.0060	0.0044	0.0068	Hematopoietic cell lineage
5332	0.0074	0.0106	0.0158	Graft-versus-host disease
5330	0.0078	0.0126	0.0189	Allograft rejection
5320	0.0246	0.0042	0.0197	Autoimmune thyroid disease
4940	0.0304	0.0072	0.0319	Type I diabetes mellitus

“Joint p” is the probability of observing by chance at least one category among the entire set of categories tested with joint enrichment (defined as the product of enrichment p-values from the two GWAS) at least as extreme as that observed in the real data. This corrects for the multiple non-independent GO categories being tested.
