# Supplementary material for: Genetic Evidence Implicates the Immune System and Cholesterol Metabolism in the Aetiology of Alzheimer's Disease
Source: PLoS One. 2010 Nov 15;5(11):e13950. doi: 10.1371/journal.pone.0013950 (PMC2981526; doi:10.1371/journal.pone.0013950)
Supplement: Table S4 — All genes in the KEGG immune-related categories in Table S3. “Best p (corrected)” is the significance of the best single-SNP p-value corrected for testing multiple SNPs in a gene (allowing for LD between SNPs). “Set based p” refers to a test of whether the average single-SNP chi-squared (allelic) association statistic is significantly high (again allowing for LD between SNPs). (0.01 MB PDF) [file pone.0013950.s004.pdf]

Table S4 All genes in the KEGG immune-related categories in Table S3

“Best p (corrected)” is the significance of the best single-SNP p-value corrected for testing multiple SNPs in a gene (allowing for LD between SNPs). “Set based p” refers to a test of whether the average single-SNP chi-squared (allelic) association statistic is significantly high (again allowing for LD between SNPs).

Gene ID number	Gene Symbol	Chr	No. of SNPs (Harold)	Most Sig Assoc p-value (Harold)	No. of SNPs (Lambert)	Most Sig Assoc p-value (Lambert)	Corrected best p (Harold)	Set-based p (Harold)	Simes-corrected p (Lambert)
1378	CR1	1	29	0.00000832	29	0.00000103	0.0002	0.0003	<0.0001
2206	MS4A2	11	11	0.00005739	10	0.04515877	0.0006	0.0010	0.2109
3123	HLA-DRB1	6	18	0.00015490	12	0.00012917	0.0028	0.0010	0.0011
3122	HLA-DRA	6	50	0.00039210	45	0.00046277	0.0190	0.0420	0.0208
3002	GZMB	14	20	0.00049510	20	0.04058881	0.0099	0.4565	0.2168
1380	CR2	1	21	0.00052180	21	0.00020979	0.0083	0.0114	0.0044
3112	HLA-DOB	6	75	0.00056360	64	0.00202924	0.0210	0.1399	0.1299
3676	ITGA4	2	26	0.00064550	25	0.01336532	0.0166	0.3487	0.1856
3674	ITGA2B	17	3	0.00083120	3	0.13378723	0.0025	0.0020	0.1831
3117	HLA-DQA1	6	24	0.00093190	14	0.00662362	0.0150	0.0669	0.0927
290	ANPEP	15	19	0.00098920	20	0.02098184	0.0186	0.2607	0.3402
4311	MME	3	28	0.00136100	28	0.00659825	0.0360	0.1648	0.1363
5799	PTPRN2	7	234	0.00187800	225	0.00587385	0.3559	0.5325	0.5941
356	FASLG	1	10	0.00190300	10	0.28340265	0.0170	0.0060	0.9338
1604	CD55	1	4	0.00197100	4	0.04281225	0.0079	0.0729	0.1285
2815	GP9	3	2	0.00263700	2	0.67754722	0.0053	0.0010	0.7216
929	CD14	5	14	0.00279200	14	0.06106207	0.0220	0.0260	0.3346
948	CD36	7	15	0.00293200	14	0.27579400	0.0431	0.2348	0.9163
3119	HLA-DQB1	6	19	0.00319500	12	0.00610550	0.0400	0.0190	0.0733
4254	KITLG	12	22	0.00410900	22	0.26134846	0.0866	0.2138	0.9571
945	CD33	19	13	0.00513400	10	0.00124969	0.0490	0.0459	0.0051
3111	HLA-DOA	6	68	0.00518200	67	0.00097250	0.1858	0.5375	0.0362
3821	KLRC1	12	5	0.00535200	3	0.55735761	0.0190	0.0450	0.6561
3815	KIT	4	31	0.00564200	29	0.01679184	0.1609	0.7083	0.4870
3655	ITGA6	2	46	0.00579500	48	0.10714733	0.2346	0.0509	0.9577
3690	ITGB3	17	28	0.00592600	28	0.00912907	0.0999	0.0320	0.2406
7038	TG	8	94	0.00693800	91	0.02051889	0.4803	0.7562	0.9224
1436	CSF1R	5	47	0.00714200	47	0.09129442	0.1988	0.1479	0.9626
3108	HLA-DMA	6	42	0.00797000	39	0.01987774	0.2008	0.4006	0.7752
3382	ICA1	7	71	0.00836100	65	0.01378867	0.3367	0.1928	0.6065
3566	IL4R	16	28	0.00844000	27	0.09647925	0.1309	0.1049	0.8491
7173	TPO	2	53	0.00896400	52	0.01305678	0.3157	0.3876	0.4740
3574	IL7	8	19	0.00927200	18	0.03908199	0.0999	0.1329	0.7035
3568	IL5RA	3	50	0.00975200	49	0.00269213	0.3457	0.5914	0.1319
926	CD8B	2	8	0.01035000	6	0.45551199	0.0599	0.0320	0.7079
7253	TSHR	14	59	0.01100000	55	0.01926499	0.4066	0.6853	0.7070
2322	FLT3	13	25	0.01187000	24	0.02856898	0.2398	0.0549	0.6857
920	CD4	12	29	0.01408000	28	0.09531122	0.2547	0.3986	0.8686
960	CD44	11	59	0.01440000	52	0.00167258	0.5750	0.4256	0.0870
1791	DNTT	10	16	0.01554000	16	0.00337235	0.2217	0.1119	0.0242
3105	HLA-A	6	35	0.01581000	27	0.05961366	0.3506	0.7253	0.6613
942	CD86	3	24	0.01587000	23	0.05430495	0.2587	0.3077	0.5917
3569	IL6	7	17	0.01614000	17	0.03573722	0.1768	0.2048	0.3321
952	CD38	4	15	0.01636000	16	0.01198153	0.2192	0.5944	0.1211
3134	HLA-F	6	34	0.01683000	31	0.01194847	0.4385	0.7203	0.1548
3630	INS	11	9	0.01925000	9	0.21881893	0.1605	0.4725	0.9321
940	CD28	2	10	0.01928000	10	0.42676902	0.1079	0.0540	0.8937
3586	IL10	1	18	0.02042000	17	0.05432774	0.2567	0.2627	0.4084
915	CD3D	11	12	0.02044000	12	0.37091404	0.1608	0.1349	0.9535
917	CD3G	11	11	0.02044000	11	0.46522278	0.1598	0.1209	0.9535
3118	HLA-DQA2	6	45	0.02065000	39	0.09014961	0.2977	0.1439	0.9681
3672	ITGA1	5	82	0.02133000	81	0.01919898	0.6583	0.2887	0.8522

3127	HLA-DRB5	6	6	0.02194000	1	0.37595102	0.0919	0.1159	0.3760
921	CD5	11	16	0.02348000	15	0.10718791	0.2078	0.1528	0.8414
3578	IL9	5	20	0.02453000	21	0.00915057	0.3447	0.3816	0.1922
3106	HLA-B	6	49	0.02488000	35	0.01262295	0.5135	0.5025	0.3970
933	CD22	19	16	0.02507000	14	0.09611820	0.2667	0.2767	0.7340
909	CD1A	1	3	0.02652000	4	0.55040061	0.0775	0.5105	0.7124
3553	IL1B	2	11	0.02861000	10	0.14206944	0.2368	0.3896	0.9601
1441	CSF3R	1	18	0.02901000	21	0.00482631	0.4113	0.5714	0.1014
947	CD34	1	16	0.03003000	15	0.07140601	0.2967	0.1518	0.5246
966	CD59	11	26	0.03075000	26	0.00702604	0.3676	0.2318	0.1591
3567	IL5	5	7	0.03096000	7	0.31342208	0.1419	0.1658	0.7352
3559	IL2RA	10	55	0.03149000	57	0.02786500	0.6723	0.3457	0.5878
3109	HLA-DMB	6	42	0.03249000	39	0.01987774	0.7502	0.7373	0.7752
924	CD7	17	4	0.03317000	5	0.04539241	0.1039	0.1668	0.2270
2993	GYPA	4	8	0.03457000	7	0.20945345	0.1648	0.3177	0.7069
2208	FCER2	19	16	0.03468000	16	0.06860438	0.3716	0.2328	0.7179
3824	KLRD1	12	9	0.03523000	10	0.29028481	0.1908	0.2907	0.9409
6356	CCL11	17	13	0.03606000	14	0.11793748	0.2498	0.1109	0.9057
1493	CTLA4	2	7	0.03837000	7	0.31128871	0.1908	0.3247	0.7491
2057	EPOR	19	6	0.03869000	6	0.33823758	0.1608	0.0989	0.7172
5553	PRG2	11	7	0.04488000	8	0.00577245	0.1948	0.1329	0.0215
3458	IFNG	12	11	0.04749000	11	0.54338610	0.3027	0.2927	0.9920
2207	FCER1G	1	16	0.04880000	16	0.01237873	0.4086	0.3906	0.1981
3673	ITGA2	5	42	0.04960000	35	0.00962238	0.7333	0.3576	0.3368
1363	CPE	4	42	0.05574000	41	0.03624798	N/A	N/A	0.9691
2323	FLT3LG	19	6	0.05633000	7	0.04162714	N/A	N/A	0.1923
4049	LTA	6	31	0.05746000	32	0.00281555	N/A	N/A	0.0901
7124	TNF	6	30	0.05746000	30	0.01436683	N/A	N/A	0.3325
5551	PRF1	10	17	0.05896000	17	0.07987220	N/A	N/A	0.9920
3113	HLA-DPA1	6	45	0.06027000	41	0.00065736	0.7273	0.9391	0.0264
7037	TFRC	3	13	0.06187000	13	0.25401241	N/A	N/A	0.9920
3442	IFNA5	9	17	0.06325000	16	0.02064280	N/A	N/A	0.2038
3440	IFNA2	9	8	0.06513000	8	0.01933898	N/A	N/A	0.1547
3447	IFNA13	9	6	0.06513000	6	0.01933898	N/A	N/A	0.1160
3448	IFNA14	9	6	0.06615000	5	0.00086300	N/A	N/A	0.0026
3444	IFNA7	9	8	0.06615000	7	0.00493911	N/A	N/A	0.0310
3446	IFNA10	9	8	0.06615000	7	0.00493911	N/A	N/A	0.0310
3449	IFNA16	9	5	0.06615000	4	0.00493911	N/A	N/A	0.0177
3451	IFNA17	9	4	0.06615000	2	0.00885124	N/A	N/A	0.0177
6037	RNASE3	14	8	0.06885000	8	0.20545031	N/A	N/A	0.4905
3592	IL12A	3	18	0.06952000	17	0.06628503	N/A	N/A	0.6174
3554	IL1R1	2	43	0.07186000	43	0.02888423	N/A	N/A	0.4956
355	FAS	10	25	0.07276000	24	0.26920372	0.7493	0.8142	0.8927
1435	CSF1	1	19	0.07353000	19	0.13740343	N/A	N/A	0.8911
3107	HLA-C	6	57	0.07459000	44	0.06388657	0.8881	0.8452	0.9013
914	CD2	1	9	0.07875000	9	0.07729881	N/A	N/A	0.5336
2205	FCER1A	1	11	0.08126000	13	0.05865897	N/A	N/A	0.5376
2571	GAD1	2	18	0.08493000	16	0.00050333	N/A	N/A	0.0081
7066	THPO	3	5	0.08517000	5	0.08875561	N/A	N/A	0.4438
3329	HSPD1	2	3	0.08637000	3	0.38364166	0.2374	0.6194	0.6804
931	MS4A1	11	24	0.08650000	23	0.12154489	N/A	N/A	0.8264
930	CD19	16	2	0.08982000	4	0.10640629	N/A	N/A	0.4256
959	CD40LG	23	3	0.09183000	0	N/A	N/A	N/A	N/A
3593	IL12B	5	13	0.09580000	13	0.11838941	N/A	N/A	0.6010
913	CD1E	1	7	0.09867000	6	0.32966840	N/A	N/A	0.8234
7850	IL1R2	2	59	0.10030000	56	0.20282707	N/A	N/A	0.6313
3590	IL11RA	9	7	0.10160000	7	0.46757543	N/A	N/A	0.9188
3570	IL6R	1	11	0.10220000	11	0.34415290	N/A	N/A	0.9805
3135	HLA-G	6	55	0.10430000	53	0.01735612	0.8761	0.9351	0.6223
928	CD9	12	14	0.11410000	15	0.06722542	N/A	N/A	0.2943
2572	GAD2	10	20	0.11510000	19	0.15447453	N/A	N/A	0.9423
5798	PTPRN	2	11	0.11590000	10	0.08729293	N/A	N/A	0.5463

912	CD1D	1	13	0.11680000	9	0.22921918	0.8010	0.7293	0.8915
3675	ITGA3	17	12	0.11730000	14	0.14216198	N/A	N/A	0.9018
2812	GP1BB	22	7	0.11820000	8	0.05363252	N/A	N/A	0.4291
3115	HLA-DPB1	6	46	0.11850000	45	0.09173278	0.9101	0.9970	0.9528
951	CD37	19	1	0.12140000	2	0.38552809	N/A	N/A	0.7711
8288	EPX	17	13	0.13460000	14	0.02932384	N/A	N/A	0.4105
1081	CGA	6	13	0.14150000	13	0.12184838	N/A	N/A	0.6930
2811	GP1BA	17	6	0.14260000	6	0.00034829	N/A	N/A	0.0021
3678	ITGA5	12	7	0.15340000	7	0.10310150	N/A	N/A	0.7217
3452	IFNA21	9	7	0.15820000	7	0.00876988	N/A	N/A	0.0614
958	CD40	20	17	0.15979999	17	0.09267406	N/A	N/A	0.7753
3804	KIR2DL3	19	2	0.16150001	1	0.20415477	N/A	N/A	0.2042
3575	IL7R	5	14	0.16400000	11	0.22960608	N/A	N/A	0.8440
3589	IL11	19	9	0.16410001	9	0.01685509	N/A	N/A	0.1473
911	CD1C	1	3	0.17090000	3	0.64598477	0.4301	0.9071	0.8040
3133	HLA-E	6	20	0.17410000	18	0.00322623	0.9051	0.9830	0.0581
941	CD80	3	28	0.17500000	26	0.00641026	N/A	N/A	0.1667
3552	IL1A	2	11	0.17980000	11	0.29459554	N/A	N/A	1.0000
1440	CSF3	17	5	0.18179999	4	0.09773626	N/A	N/A	0.3909
3445	IFNA8	9	10	0.19690000	9	0.00425091	N/A	N/A	0.0206
2814	GP5	3	5	0.19810000	5	0.00222208	N/A	N/A	0.0072
1437	CSF2	5	6	0.20160000	6	0.50366765	N/A	N/A	0.9761
3562	IL3	5	4	0.20160000	4	0.50366765	N/A	N/A	0.9761
916	CD3E	11	6	0.20800000	6	0.37091404	N/A	N/A	0.7497
7252	TSHB	1	8	0.24640000	8	0.35953981	N/A	N/A	0.7611
3812	KIR3DL2	19	7	0.26230001	6	0.73844284	N/A	N/A	0.9528
925	CD8A	2	5	0.27030000	6	0.52926630	N/A	N/A	0.6926
3684	ITGAM	16	13	0.28830001	12	0.00024742	N/A	N/A	0.0030
910	CD1B	1	3	0.28990000	2	0.33460322	0.5425	0.6244	0.6144
3439	IFNA1	9	6	0.30800000	8	0.00900719	N/A	N/A	0.0721
3565	IL4	5	8	0.33350000	8	0.18047601	N/A	N/A	0.8975
3596	IL13	5	6	0.33350000	6	0.18047601	N/A	N/A	0.8732
3811	KIR3DL1	19	3	0.41630000	3	0.87536788	N/A	N/A	0.9287
3441	IFNA4	9	9	0.42120001	9	0.00493911	N/A	N/A	0.0395
2056	EPO	7	1	0.47380000	1	0.65327185	N/A	N/A	0.6533
3443	IFNA6	9	4	0.47870001	5	0.01933898	N/A	N/A	0.0519
3558	IL2	4	3	0.53250003	3	0.01913758	N/A	N/A	0.0574
3581	IL9R	23	0	N/A	0	N/A	N/A	N/A	N/A
1438	CSF2RA	23	0	N/A	0	N/A	N/A	N/A	N/A
3563	IL3RA	23	0	N/A	0	N/A	N/A	N/A	N/A
3802	KIR2DL1	19	0	N/A	0	N/A	N/A	N/A	N/A
57292	KIR2DL5A	19	0	N/A	0	N/A	N/A	N/A	N/A
2209	FCGR1A	1	0	N/A	0	N/A	N/A	N/A	N/A
