# Supplementary material for: Genetic Evidence Implicates the Immune System and Cholesterol Metabolism in the Aetiology of Alzheimer's Disease
Source: PLoS One. 2010 Nov 15;5(11):e13950. doi: 10.1371/journal.pone.0013950 (PMC2981526; doi:10.1371/journal.pone.0013950)
Supplement: Table S5 — MGI mouse phenotypes identified by ALIGATOR analysis of the AD GWA data of Harold and colleagues. The 589 genes identified as having GWAS SNP signals p<0·001 were used: APOE was included in the gene list. In this analysis one SNP was not allowed to add more than one gene to any phenotype. “Study-wide p-value” is the probability of obtaining by chance at least one mouse phenotype with a phenotype-specific enrichment p-value at least as significant as that observed. (0.08 MB PDF) [file pone.0013950.s005.pdf]

Table S5 MGI mouse phenotypes identified by ALIGATOR analysis of the AD GWA data of Harold and colleagues (7)

The 589 genes identified as having GWAS SNP signals p < 0·001 were used: APOE was included in the gene list. In this analysis one SNP was not allowed to add more than one gene to any phenotype. "Study-wide p-value" is the probability of obtaining by chance at least one mouse phenotype with a phenotype-specific enrichment p-value at least as significant as that observed.

Phenotype number	total genes in pathway	#genes on list	expected #genes on list	p-value	Study-wide corrected p-value	Function
6084	12	6	0.53	<0.0001	0.323	abnormal circulating phospholipid level
5339	11	4	0.59	0.0006	0.652	increased susceptibility to atherosclerosis
4777	25	6	1.18	0.0008	0.716	abnormal phospholipid level
3980	6	3	0.18	0.0010	0.763	increased circulating phospholipid level
6303	9	3	0.24	0.0010	0.763	abnormal retinal nerve fiber layer morphology
5340	18	4	0.72	0.0012	0.802	altered susceptibility to atherosclerosis
3976	5	2	0.06	0.0012	0.802	decreased circulating VLDL triglyceride level
2442	777	38	23.36	0.0020	0.902	abnormal leukocyte physiology
3009	347	19	9.4	0.0022	0.919	abnormal cytokine secretion
6207	589	30	18.19	0.0022	0.919	embryonic lethality during organogenesis
2421	794	38	23.77	0.0024	0.929	abnormal cell-mediated immunity
2420	798	38	23.87	0.0024	0.929	abnormal adaptive immunity
1819	793	38	23.74	0.0024	0.929	abnormal immune cell physiology
5338	20	4	0.75	0.0024	0.929	atherosclerotic lesions
8704	68	6	1.5	0.0032	0.964	abnormal interleukin-6 secretion
3991	22	4	0.82	0.0034	0.971	arteriosclerosis
4801	7	2	0.09	0.0034	0.971	increased susceptibility to systemic lupus erythematosus
5014	95	8	2.61	0.0038	0.977	increased B cell number
8477	5	2	0.12	0.0038	0.977	decreased spleen red pulp amount
8568	213	13	5.56	0.0040	0.980	abnormal interleukin secretion
2723	553	27	15.44	0.0040	0.980	abnormal immune serum protein physiology
2080	1262	52	37.17	0.0054	0.993	prenatal lethality
3842	4	2	0.15	0.0060	0.996	abnormal metopic suture morphology
4810	18	3	0.45	0.0064	0.996	decreased hematopoietic stem cell number
4808	35	4	0.76	0.0066	0.997	abnormal hematopoietic stem cell morphology
5144	32	4	0.79	0.0070	0.997	abnormal circulating VLDL cholesterol level
4939	306	18	9.73	0.0078	0.998	abnormal B cell morphology
2452	506	25	15	0.0080	0.998	abnormal antigen presenting cell physiology
180	162	12	5.67	0.0082	0.998	abnormal circulating cholesterol level
3053	5	2	0.18	0.0086	0.998	delayed tooth eruption
5178	93	8	2.94	0.0088	0.999	increased circulating cholesterol level
4762	32	4	0.84	0.0088	0.999	increased anti-double stranded DNA antibody level
8781	17	3	0.49	0.0090	0.999	abnormal B cell apoptosis
8246	880	39	26.63	0.0096	0.999	abnormal leukocyte morphology
1685	44	5	1.38	0.0096	0.999	abnormal endoderm development
716	886	39	26.72	0.0098	0.999	abnormal immune system cell morphology
8706	36	4	0.83	0.0098	0.999	decreased interleukin-6 secretion
2086	468	23	13.83	0.0104	1.000	abnormal extraembryonic tissue morphology
2459	322	17	9.29	0.0104	1.000	abnormal B cell physiology
1552	74	7	2.47	0.0104	1.000	increased circulating triglyceride level
5491	19	3	0.45	0.0104	1.000	pancreatic islet hyperplasia
184	64	7	2.63	0.0108	1.000	abnormal circulating HDL cholesterol level
5145	16	3	0.48	0.0110	1.000	increased circulating VLDL cholesterol level
239	15	3	0.52	0.0110	1.000	absent common myeloid progenitor cells
4557	7	2	0.17	0.0110	1.000	dilated allantois
5317	94	8	3.05	0.0114	1.000	increased triglyceride level
3945	563	26	16.39	0.0116	1.000	abnormal lymphocyte physiology
2458	237	15	8.07	0.0142	1.000	abnormal B cell number
2415	8	2	0.19	0.0142	1.000	abnormal neutrophil differentiation
4229	18	3	0.54	0.0146	1.000	abnormal embryonic erythropoiesis
5006	40	5	1.57	0.0148	1.000	abnormal osteoblast physiology
5325	137	10	4.54	0.0152	1.000	abnormal renal glomerulus morphology
3604	19	4	1.05	0.0156	1.000	single kidney
2190	29	4	1.11	0.0156	1.000	disorganized myocardium
175	17	3	0.61	0.0162	1.000	absent bone marrow cell
1289	15	3	0.56	0.0166	1.000	persistence of hyaloid capillary system
8705	30	3	0.59	0.0168	1.000	increased interleukin-6 secretion
2743	49	5	1.5	0.0170	1.000	glomerulonephritis
4794	43	4	1.01	0.0170	1.000	increased anti-nuclear antigen antibody level
1783	57	5	1.49	0.0170	1.000	decreased white adipose tissue amount
8907	8	2	0.22	0.0172	1.000	decreased total fat pad weight

2429	1137	47	34.54	0.0176	1.000	abnormal blood cell morphology/development
9112	20	3	0.6	0.0176	1.000	abnormal pancreatic beta cell mass
1802	31	4	1.1	0.0180	1.000	arrested B cell differentiation
4796	10	2	0.22	0.0182	1.000	increased anti-histone antibody level
5330	50	6	2.25	0.0184	1.000	cardiomyopathy
1602	9	2	0.24	0.0186	1.000	impaired myelopoiesis
183	19	3	0.62	0.0188	1.000	decreased circulating LDL cholesterol level
8560	42	4	1.02	0.0190	1.000	increased tumor necrosis factor secretion
2718	73	5	1.52	0.0192	1.000	abnormal inner cell mass
8556	92	7	2.79	0.0194	1.000	abnormal tumor necrosis factor secretion
6208	232	14	7.66	0.0202	1.000	lethality throughout fetal growth and development
3643	14	3	0.84	0.0202	1.000	spleen atrophy
2144	220	13	7.02	0.0204	1.000	abnormal B cell differentiation
3947	175	12	6.28	0.0206	1.000	abnormal cholesterol level
248	9	2	0.24	0.0208	1.000	macrocytosis
9814	10	2	0.25	0.0210	1.000	increased prostaglandin level
8209	40	4	1.1	0.0212	1.000	decreased pre-B cell number
8762	943	37	26.35	0.0218	1.000	embryonic lethality
2451	215	12	6.4	0.0218	1.000	abnormal macrophage physiology
1556	30	4	1.09	0.0218	1.000	increased circulating HDL cholesterol level
8195	315	17	9.97	0.0220	1.000	abnormal antigen presenting cell morphology
2123	1123	46	34.22	0.0222	1.000	abnormal hematopoiesis
5350	73	5	1.64	0.0222	1.000	increased susceptibility to autoimmune disorder
136	26	3	0.62	0.0234	1.000	abnormal microglial cell morphology
4111	15	3	0.66	0.0234	1.000	abnormal coronary artery morphology
5278	181	12	6.45	0.0236	1.000	abnormal cholesterol homeostasis
8813	21	3	0.64	0.0238	1.000	decreased common myeloid progenitor cell number
8247	785	33	23.12	0.0246	1.000	abnormal mononuclear leukocyte morphology
3884	46	4	1.15	0.0250	1.000	decreased macrophage cell number
5495	39	4	1.11	0.0250	1.000	abnormal macrophage recruitment
119	23	3	0.69	0.0252	1.000	abnormal tooth eruption
4771	12	2	0.26	0.0252	1.000	increased anti-single stranded DNA antibody level
521	162	11	5.64	0.0256	1.000	abnormal kidney cortex
3141	74	7	2.96	0.0258	1.000	cardiac fibrosis
3057	16	3	0.74	0.0262	1.000	abnormal epicardium morphology
181	38	4	1.17	0.0268	1.000	abnormal circulating LDL cholesterol level
2465	22	3	0.65	0.0268	1.000	abnormal eosinophil physiology
4368	8	2	0.28	0.0270	1.000	abnormal stria vascularis vasculature morphology
3982	106	8	3.56	0.0282	1.000	increased cholesterol level
872	36	6	2.52	0.0282	1.000	abnormal cerebellum external granule cell layer morphology
5496	26	3	0.67	0.0294	1.000	impaired macrophage recruitment
3020	4	2	0.56	0.0298	1.000	decreased circulating chloride level
1983	24	4	1.31	0.0304	1.000	abnormal olfactory system physiology
9171	27	3	0.72	0.0306	1.000	enlarged pancreatic islets
2414	512	23	15.33	0.0308	1.000	abnormal myeloblast morphology/development
2620	63	5	1.78	0.0310	1.000	abnormal monocyte morphology
576	4	2	0.36	0.0318	1.000	clubfoot
5243	6	2	0.33	0.0328	1.000	hemothorax
9113	9	2	0.29	0.0330	1.000	increased pancreatic beta cell mass
3949	238	13	7.45	0.0332	1.000	abnormal circulating lipid level
3725	53	4	1.27	0.0332	1.000	increased autoantibody level
2827	147	10	5.18	0.0348	1.000	abnormal renal corpuscle morphology
1614	544	27	18.84	0.0358	1.000	abnormal blood vessel morphology
2210	310	14	8.43	0.0362	1.000	abnormal sex determination
6135	9	2	0.31	0.0364	1.000	arterial stenosis
2837	10	2	0.32	0.0364	1.000	dystrophic cardiac calcinosis
3898	22	4	1.41	0.0380	1.000	abnormal QRS complex
2887	18	3	0.83	0.0380	1.000	decreased susceptibility to pharmacologically induced seizures
1378	8	2	0.35	0.0382	1.000	abnormal ejaculation
2425	155	8	3.83	0.0388	1.000	altered susceptibility to autoimmune disorder
1689	36	3	0.76	0.0392	1.000	incomplete somite formation
1360	23	4	1.45	0.0394	1.000	abnormal social investigation
6032	31	4	1.42	0.0396	1.000	abnormal ureteric bud morphology
186	36	4	1.43	0.0406	1.000	decreased circulating HDL cholesterol level
2818	10	2	0.34	0.0408	1.000	abnormal dentin morphology
1354	10	2	0.33	0.0414	1.000	increased aggression towards males
4985	28	3	0.78	0.0416	1.000	decreased osteoclast cell number
2191	219	12	6.95	0.0420	1.000	abnormal artery morphology
8210	46	4	1.32	0.0424	1.000	increased mature B cell number
5387	1591	60	48.49	0.0426	1.000	immune system phenotype
6382	37	4	1.4	0.0426	1.000	abnormal lung epithelium morphology

2065	162	13	7.89	0.0428	1.000	abnormal fear/anxiety-related behavior
5146	17	2	0.32	0.0444	1.000	decreased circulating VLDL cholesterol level
4957	91	5	2	0.0452	1.000	abnormal blastocyst morphology
8720	31	4	1.47	0.0454	1.000	impaired neutrophil migration
2419	304	15	9.39	0.0468	1.000	abnormal innate immunity
2127	890	41	31.7	0.0474	1.000	abnormal cardiovascular system morphology
6203	8	2	0.38	0.0476	1.000	eye hemorrhage
5412	13	2	0.36	0.0478	1.000	vascular stenosis
5460	501	22	15.12	0.0482	1.000	abnormal leukopoiesis
1844	190	9	4.68	0.0482	1.000	autoimmune response
4965	22	2	0.35	0.0482	1.000	inner cell mass degeneration
8782	13	2	0.39	0.0488	1.000	increased B cell apoptosis
8248	226	11	6.29	0.0496	1.000	abnormal mononuclear phagocyte morphology
1675	74	5	2.01	0.0498	1.000	abnormal ectoderm development
2333	10	2	0.37	0.0498	1.000	abnormal lung compliance
2444	363	16	10.3	0.0508	1.000	abnormal T cell physiology
523	72	5	2.04	0.0518	1.000	cortical renal glomerulopathies
1711	263	12	7.18	0.0520	1.000	abnormal placenta morphology
5005	191	9	4.75	0.0520	1.000	abnormal self tolerance
5000	194	9	4.81	0.0538	1.000	abnormal immune tolerance
4986	56	5	2.18	0.0540	1.000	abnormal osteoblast morphology
8181	15	2	0.38	0.0540	1.000	increased marginal zone B cell number
4264	15	2	0.41	0.0542	1.000	abnormal extraembryonic tissue physiology
2396	1276	49	39.36	0.0552	1.000	abnormal hematopoietic system morphology/development
9768	62	4	1.44	0.0552	1.000	impaired somite development
2445	471	21	14.49	0.0556	1.000	abnormal mononuclear leukocyte differentiation
4029	16	2	0.39	0.0556	1.000	spontaneous chromosome breakage
2461	144	8	4.13	0.0564	1.000	increased immunoglobulin level
8662	22	2	0.38	0.0564	1.000	abnormal interleukin-12 secretion
1726	59	4	1.5	0.0576	1.000	abnormal allantois morphology
8082	82	6	2.76	0.0578	1.000	increased single-positive T cell number
2909	19	3	0.95	0.0580	1.000	abnormal adrenal gland physiology
2024	16	2	0.41	0.0584	1.000	T cell derived lymphoma
1698	383	16	10.32	0.0590	1.000	decreased embryo size
1712	136	7	3.56	0.0592	1.000	abnormal placenta development
3722	10	2	0.41	0.0596	1.000	absent ureter
5397	1320	50	40.52	0.0602	1.000	hematopoietic system phenotype
3106	28	4	1.58	0.0606	1.000	abnormal fear-related response
217	681	29	21.57	0.0612	1.000	abnormal leukocyte cell number
48	33	4	1.56	0.0612	1.000	abnormal stria vascularis
5069	71	5	2.13	0.0614	1.000	abnormal NK cell physiology
237	494	23	16.48	0.0630	1.000	decreased blood cell number
2798	8	2	0.45	0.0638	1.000	abnormal active avoidance behavior
2403	55	4	1.53	0.0638	1.000	abnormal pre-B cell morphology
1984	19	3	0.97	0.0640	1.000	abnormal olfaction
2490	261	12	7.31	0.0646	1.000	abnormal immunoglobulin level
8081	184	10	5.74	0.0650	1.000	abnormal single-positive T cell number
5322	43	4	1.52	0.0658	1.000	abnormal serotonin level
2619	669	27	20.01	0.0662	1.000	abnormal lymphocyte morphology
8125	35	3	0.96	0.0666	1.000	abnormal dendritic cell number
5095	116	7	3.57	0.0668	1.000	decreased T cell proliferation
5179	79	6	3.02	0.0676	1.000	decreased circulating cholesterol level
8482	13	2	0.45	0.0680	1.000	decreased spleen germinal center number
1697	388	16	10.57	0.0684	1.000	abnormal embryo size
2118	349	16	10.93	0.0688	1.000	abnormal lipid homeostasis
8250	356	15	9.93	0.0696	1.000	abnormal myeloid leukocyte morphology
1547	312	15	10.06	0.0696	1.000	abnormal lipid level
2462	154	9	5.17	0.0696	1.000	abnormal granulocyte physiology
2492	36	3	0.96	0.0696	1.000	decreased IgE level
8559	134	7	3.63	0.0698	1.000	abnormal interferon-gamma secretion
1674	167	8	4.36	0.0700	1.000	abnormal triploblastic development
4057	16	2	0.45	0.0704	1.000	thin myocardial compact layer
4989	17	2	0.46	0.0708	1.000	decreased osteoblast cell number
5017	189	11	6.82	0.0710	1.000	decreased B cell number
2401	463	20	14.14	0.0730	1.000	abnormal lymphopoiesis
187	166	9	5.24	0.0734	1.000	abnormal triglyceride level
5584	110	7	3.72	0.0734	1.000	abnormal enzyme/coenzyme activity
1695	171	8	4.45	0.0734	1.000	abnormal gastrulation
2275	17	2	0.47	0.0740	1.000	abnormal type II pneumocyte morphology
2736	22	3	1	0.0750	1.000	abnormal nociception after inflammation
1718	188	10	5.97	0.0752	1.000	abnormal yolk sac morphology

685	1110	43	34.63	0.0754	1.000	abnormal immune system morphology
5090	52	4	1.61	0.0760	1.000	increased double-negative T cell number
8973	20	2	0.46	0.0768	1.000	decreased erythroid progenitor cell number
1781	119	6	2.95	0.0772	1.000	abnormal white adipose tissue amount
8215	68	5	2.33	0.0784	1.000	decreased immature B cell number
8469	306	14	9.37	0.0788	1.000	abnormal protein level
1672	1018	38	30.16	0.0794	1.000	abnormal embryogenesis/ development
9657	38	3	1.01	0.0794	1.000	failure of chorioallantoic fusion
5380	1018	38	30.16	0.0794	1.000	embryogenesis phenotype
2836	36	3	1.04	0.0806	1.000	abnormal chorion morphology
4987	35	3	1.04	0.0806	1.000	abnormal osteoblast cell number
3871	31	3	1.03	0.0806	1.000	abnormal myelin sheath morphology
260	218	12	7.67	0.0808	1.000	abnormal angiogenesis
249	197	11	7.02	0.0814	1.000	abnormal blood vessel physiology
8713	175	8	4.6	0.0824	1.000	abnormal cytokine level
8074	51	4	1.67	0.0832	1.000	increased CD4-positive T cell number
2572	252	18	12.76	0.0842	1.000	abnormal emotion/affect behavior
4567	11	2	0.54	0.0844	1.000	decreased myocardial fiber number
3137	49	5	2.4	0.0848	1.000	abnormal impulse conducting system conduction
1790	1233	46	37.77	0.0850	1.000	abnormal immune system physiology
3983	88	6	3.18	0.0850	1.000	decreased cholesterol level
6043	51	3	1.07	0.0852	1.000	decreased apoptosis
5456	35	3	1.05	0.0852	1.000	weight gain
2833	60	4	1.73	0.0854	1.000	increased heart weight
2211	286	12	7.84	0.0860	1.000	abnormal primary sex determination
1127	82	5	2.47	0.0860	1.000	small ovary
2966	6	2	0.67	0.0860	1.000	decreased circulating alkaline phosphatase level
8251	289	12	7.74	0.0862	1.000	abnormal phagocyte morphology
5022	93	6	3.11	0.0866	1.000	abnormal immature B cell morphology
528	12	2	0.53	0.0876	1.000	delayed kidney development
278	110	7	3.86	0.0880	1.000	abnormal myocardial fiber morphology
3396	38	3	1.08	0.0884	1.000	abnormal embryonic hematopoiesis
5385	1189	53	44.23	0.0888	1.000	cardiovascular system phenotype
3461	19	3	1.17	0.0888	1.000	abnormal response to novel object
2652	57	4	1.73	0.0892	1.000	thin myocardial wall
5646	16	3	1.2	0.0892	1.000	abnormal pituitary gland physiology
221	486	22	16.34	0.0894	1.000	decreased leukocyte cell number
4951	97	6	3.19	0.0894	1.000	abnormal spleen weight
5164	227	11	7.07	0.0904	1.000	abnormal response to injury
8561	49	4	1.78	0.0910	1.000	decreased tumor necrosis factor secretion
8037	495	20	14.61	0.0914	1.000	abnormal T cell morphology
8217	100	6	3.12	0.0916	1.000	abnormal B cell activation
8555	150	7	3.85	0.0918	1.000	abnormal interferon secretion
3191	11	2	0.55	0.0922	1.000	abnormal cellular cholesterol metabolism
2435	227	11	6.97	0.0924	1.000	abnormal effector T cell morphology
2398	694	27	20.77	0.0930	1.000	abnormal bone marrow cell morphology/development
3087	16	2	0.54	0.0930	1.000	absent allantois
2145	332	14	9.5	0.0936	1.000	abnormal T cell differentiation
351	44	3	1.08	0.0936	1.000	increased cell proliferation
2886	31	4	1.83	0.0938	1.000	abnormal glutamate-mediated receptor currents
8484	12	2	0.54	0.0938	1.000	decreased spleen germinal center size
4016	45	4	1.81	0.0940	1.000	decreased bone mass
8567	82	5	2.42	0.0942	1.000	decreased interferon-gamma secretion
2918	34	5	2.64	0.0946	1.000	abnormal paired-pulse facilitation
223	19	2	0.52	0.0952	1.000	decreased monocyte cell number
8414	25	3	1.12	0.0956	1.000	abnormal spatial reference memory
3881	219	12	7.94	0.0970	1.000	abnormal nephron morphology
9593	12	2	0.55	0.0970	1.000	absent chorion
1413	88	9	5.63	0.0974	1.000	abnormal response to new environment
1798	17	2	0.54	0.0974	1.000	impaired macrophage phagocytosis
321	26	2	0.53	0.0980	1.000	increased bone marrow cell number
8208	33	3	1.13	0.0982	1.000	decreased pro-B cell number
1417	54	6	3.36	0.0982	1.000	decreased exploration in new environment
4952	64	4	1.79	0.0992	1.000	increased spleen weight
8143	22	3	1.14	0.0994	1.000	abnormal dendrite morphology
4769	18	2	0.54	0.0996	1.000	abnormal synaptic vesicle morphology
527	60	5	2.53	0.1006	1.000	abnormal kidney development
2434	20	2	0.55	0.1006	1.000	abnormal T-helper 2 cell morphology
2397	22	3	1.22	0.1014	1.000	abnormal bone marrow morphology
9698	30	3	1.16	0.1014	1.000	heart hemorrhage
5374	1733	65	55.89	0.1018	1.000	lethality-prenatal/perinatal

2989	90	6	3.28	0.1018	1.000	small kidney
2100	103	6	3.23	0.1026	1.000	abnormal tooth morphology
8127	24	2	0.54	0.1032	1.000	decreased dendritic cell number
5030	16	2	0.56	0.1046	1.000	absent amnion
2407	79	5	2.52	0.1052	1.000	abnormal double-negative T cell morphology
3941	7	2	0.58	0.1060	1.000	abnormal skin development
5070	42	3	1.17	0.1066	1.000	impaired NK cell cytolysis
4091	9	2	0.61	0.1070	1.000	abnormal Z lines
8111	23	2	0.58	0.1084	1.000	abnormal granulocyte differentiation
2084	326	13	8.8	0.1086	1.000	abnormal developmental patterning
8552	42	3	1.2	0.1088	1.000	abnormal circulating tumor necrosis factor level
2270	125	8	4.86	0.1090	1.000	abnormal respiratory alveoli morphology
3179	61	4	1.85	0.1092	1.000	decreased platelet cell number
2446	171	8	4.84	0.1094	1.000	abnormal macrophage morphology
6414	24	2	0.57	0.1096	1.000	decreased T cell apoptosis
3085	27	2	0.57	0.1098	1.000	abnormal egg cylinder morphology
9748	54	4	1.86	0.1098	1.000	abnormal behavioral response to addictive substance
182	20	2	0.56	0.1104	1.000	increased circulating LDL cholesterol level
4076	46	4	1.86	0.1116	1.000	abnormal vitelline vascular remodeling
109	43	4	1.93	0.1120	1.000	abnormal parietal bone morphology
8073	128	7	4.11	0.1128	1.000	abnormal CD4-positive T cell number
4086	11	2	0.62	0.1130	1.000	absent heartbeat
172	94	5	2.58	0.1140	1.000	abnormal bone marrow cell number
8835	179	8	4.94	0.1154	1.000	abnormal intercellular signaling peptide or protein level
5463	100	5	2.62	0.1158	1.000	abnormal CD4-positive T cell physiology
5015	181	9	5.71	0.1176	1.000	increased T cell number
2088	527	20	15.06	0.1184	1.000	abnormal embryonic growth/weight/body size
2953	22	2	0.59	0.1184	1.000	thick ventricular wall
1800	288	12	8.22	0.1220	1.000	abnormal humoral immune response
162	22	2	0.61	0.1220	1.000	lordosis
8701	23	2	0.6	0.1224	1.000	abnormal interleukin-5 secretion
4321	22	2	0.64	0.1234	1.000	short sternum
9085	9	2	0.74	0.1244	1.000	abnormal uterine horn morphology
2970	133	6	3.4	0.1246	1.000	abnormal white adipose tissue morphology
333	63	4	1.91	0.1246	1.000	decreased bone marrow cell number
4853	91	5	2.74	0.1256	1.000	abnormal ovary size
5345	46	3	1.26	0.1266	1.000	abnormal circulating corticosterone level
2328	7	2	0.73	0.1268	1.000	abnormal airway resistance
717	516	21	16.13	0.1270	1.000	abnormal lymphocyte cell number
5461	47	3	1.24	0.1280	1.000	abnormal dendritic cell morphology
3944	24	2	0.64	0.1282	1.000	abnormal T cell subpopulation ratio
4608	26	2	0.64	0.1286	1.000	abnormal cervical axis morphology
8698	67	4	1.91	0.1294	1.000	abnormal interleukin-4 secretion
1942	18	2	0.64	0.1294	1.000	abnormal lung capacity
8700	37	3	1.26	0.1300	1.000	decreased interleukin-4 secretion
3909	76	4	1.95	0.1304	1.000	increased eating behavior
3107	96	9	5.98	0.1308	1.000	abnormal response to novelty
5416	243	11	7.56	0.1310	1.000	abnormal circulating protein level
2493	96	5	2.71	0.1314	1.000	increased IgG level
5448	105	5	2.7	0.1316	1.000	abnormal energy balance
2738	7	2	0.68	0.1316	1.000	hyperresponsive to tactile stimuli
5266	307	14	10.07	0.1336	1.000	abnormal metabolism
5007	117	7	4.35	0.1336	1.000	abnormal bone density
8554	20	2	0.69	0.1340	1.000	decreased circulating tumor necrosis factor level
5565	66	5	2.82	0.1356	1.000	increased blood urea nitrogen level
2576	20	2	0.67	0.1390	1.000	abnormal enamel morphology
2824	49	3	1.28	0.1392	1.000	abnormal chorioallantoic fusion
298	11	2	0.69	0.1392	1.000	absent endocardial cushion
8078	42	3	1.31	0.1398	1.000	increased CD8-positive T cell number
3222	21	2	0.69	0.1404	1.000	increased cardiomyocyte apoptosis
1634	169	9	5.98	0.1408	1.000	internal hemorrhage
3460	21	3	1.39	0.1412	1.000	decreased fear-related response
2085	623	23	18.25	0.1416	1.000	abnormal embryonic tissue morphology
1415	21	3	1.4	0.1434	1.000	increased exploration in new environment
1859	85	5	2.82	0.1434	1.000	kidney inflammation
6264	22	2	0.68	0.1436	1.000	decreased systolic blood pressure
2295	46	4	2.09	0.1438	1.000	abnormal pulmonary circulation
2885	12	2	0.69	0.1440	1.000	abnormal AMPA-mediated synaptic currents
5013	260	11	7.69	0.1462	1.000	increased lymphocyte cell number
8168	17	2	0.67	0.1464	1.000	decreased B-1a cell number
2432	165	8	5.23	0.1472	1.000	abnormal CD4-positive T cell morphology

2494	65	4	2.08	0.1484	1.000	increased IgM level
1745	26	2	0.7	0.1486	1.000	increased circulating corticosterone level
1258	106	6	3.63	0.1502	1.000	decreased body length
6354	14	2	0.7	0.1506	1.000	abnormal fourth branchial arch artery morphology
3942	70	5	2.86	0.1512	1.000	abnormal urinary system development
9811	21	2	0.73	0.1512	1.000	abnormal prostaglandin level
3018	10	2	0.81	0.1516	1.000	abnormal circulating chloride level
2699	31	3	1.39	0.1518	1.000	abnormal vitreous body
6340	15	2	0.71	0.1530	1.000	abnormal fourth branchial arch morphology
2286	23	2	0.71	0.1534	1.000	cryptorchism
1353	20	2	0.71	0.1536	1.000	increased aggression towards mice
4890	28	2	0.7	0.1540	1.000	decreased energy expenditure
3229	132	7	4.42	0.1546	1.000	abnormal vitelline vasculature
4917	19	2	0.69	0.1546	1.000	abnormal T cell selection
8719	36	3	1.42	0.1566	1.000	impaired neutrophil recruitment
8752	66	4	2.16	0.1568	1.000	abnormal tumor necrosis factor level
6387	365	15	11.34	0.1584	1.000	abnormal T cell number
255	29	2	0.72	0.1586	1.000	vasculature congestion
8288	25	2	0.71	0.1600	1.000	abnormal adrenal cortex morphology
4720	98	5	2.88	0.1600	1.000	abnormal platelet morphology
1691	24	2	0.72	0.1612	1.000	abnormal somite shape
1077	76	6	3.71	0.1622	1.000	abnormal spinal nerve morphology
564	49	4	2.15	0.1622	1.000	syndactyly
3690	28	2	0.73	0.1634	1.000	abnormal glial cell physiology
1544	795	36	30.55	0.1656	1.000	abnormal cardiovascular system physiology
3894	6	2	0.76	0.1656	1.000	abnormal Purkinje cell innervation
228	100	5	2.92	0.1664	1.000	abnormal thrombopoiesis
222	40	2	0.72	0.1668	1.000	decreased neutrophil cell number
5025	332	12	8.82	0.1672	1.000	abnormal response to infection
6204	106	4	2.17	0.1682	1.000	embryonic lethality before implantation
5634	7	2	0.84	0.1682	1.000	decreased circulating sodium level
3484	21	3	1.45	0.1698	1.000	abnormal channel response
3408	25	2	0.75	0.1698	1.000	increased width of hypertrophic chondrocyte zone
2022	87	5	3.11	0.1702	1.000	lymphoma
1899	18	3	1.49	0.1712	1.000	absent long term depression
1722	56	3	1.42	0.1718	1.000	pale yolk sac
218	395	15	11.5	0.1724	1.000	increased leukocyte cell number
2151	308	12	8.83	0.1732	1.000	abnormal neural tube morphology/development
5166	58	3	1.45	0.1732	1.000	decreased susceptibility to injury
8172	26	2	0.75	0.1750	1.000	abnormal follicular B cell morphology
3221	27	2	0.78	0.1756	1.000	abnormal cardiomyocyte apoptosis
2799	29	3	1.5	0.1760	1.000	abnormal passive avoidance behavior
2063	320	22	18.05	0.1780	1.000	abnormal learning/memory/conditioning
2828	26	2	0.77	0.1784	1.000	abnormal renal glomerular capsule
8866	56	3	1.5	0.1790	1.000	chromosomal instability
2439	33	2	0.77	0.1798	1.000	abnormal plasma cell morphology
5294	298	15	11.69	0.1808	1.000	abnormal heart ventricle morphology
4978	35	3	1.48	0.1812	1.000	decreased B-1 B cell number
3974	19	2	0.79	0.1812	1.000	abnormal endocardium morphology
1980	22	2	0.78	0.1814	1.000	abnormal chemically-elicited antinociception
8722	26	2	0.77	0.1814	1.000	abnormal chemokine secretion
3963	51	3	1.46	0.1818	1.000	abnormal corticosterone level
520	39	4	2.3	0.1832	1.000	absent kidney
1764	1713	63	56.59	0.1838	1.000	abnormal homeostasis
5016	408	17	13.41	0.1848	1.000	decreased lymphocyte cell number
5018	286	12	9.06	0.1850	1.000	decreased T cell number
5265	77	5	3.1	0.1860	1.000	abnormal blood urea nitrogen level
8588	88	4	2.26	0.1862	1.000	abnormal circulating interleukin level
2412	152	6	3.89	0.1862	1.000	increased susceptibility to bacterial infection
2687	123	6	3.89	0.1864	1.000	oligozoospermia
133	25	2	0.8	0.1866	1.000	abnormal long bone metaphysis morphology
5587	27	2	0.78	0.1870	1.000	abnormal Meckel's cartilage morphology
3355	21	2	0.8	0.1870	1.000	decreased ovulation frequency
5264	23	2	0.81	0.1878	1.000	glomerulosclerosis
3930	25	2	0.8	0.1884	1.000	abnormal tooth hard tissue morphology
929	126	6	3.9	0.1888	1.000	open neural tube
2273	24	2	0.8	0.1890	1.000	abnormal respiratory alveolar epithelial cell morphology
9170	48	3	1.54	0.1898	1.000	abnormal pancreatic islet size
4856	22	2	0.81	0.1906	1.000	decreased ovary weight
8164	21	2	0.8	0.1914	1.000	abnormal B-1a B cell morphology
3956	1322	58	51.81	0.1918	1.000	abnormal body size

6410	50	3	1.52	0.1932	1.000	abnormal common myeloid progenitor cell morphology
1286	129	6	3.93	0.1938	1.000	abnormal eye development
6009	57	5	3.11	0.1938	1.000	abnormal neuronal migration
5329	238	12	9.18	0.1960	1.000	abnormal cardiac muscle morphology
5394	60	4	2.33	0.1974	1.000	taste/olfaction phenotype
9858	34	2	0.82	0.1978	1.000	abnormal cellular extravasation
4056	25	2	0.83	0.1984	1.000	abnormal myocardial compact layer morphology
8854	7	2	0.84	0.2026	1.000	bleb
4854	25	2	0.84	0.2038	1.000	abnormal ovary weight
691	223	10	7.41	0.2038	1.000	enlarged spleen
2182	52	3	1.58	0.2040	1.000	abnormal astrocyte morphology
5376	2005	73	66.6	0.2054	1.000	homeostasis/metabolism phenotype
5608	24	2	0.86	0.2056	1.000	cardiac interstitial fibrosis
1625	89	6	4.02	0.2060	1.000	cardiac hypertrophy
1489	37	5	3.25	0.2064	1.000	decreased startle reflex
3795	261	12	9.21	0.2076	1.000	abnormal bone structure
259	267	12	9.18	0.2080	1.000	abnormal vascular development
2792	45	3	1.6	0.2080	1.000	abnormal retinal vasculature morphology
2148	101	4	2.4	0.2088	1.000	hypersensitivity
1256	120	6	4.01	0.2092	1.000	abnormal body length
9749	27	2	0.86	0.2094	1.000	enhanced behavioral response to addictive substance
1739	15	2	0.87	0.2114	1.000	abnormal adrenal gland secretion
2919	17	3	1.74	0.2116	1.000	enhanced paired-pulse facilitation
1116	275	10	7.48	0.2136	1.000	small gonad
8515	47	3	1.61	0.2136	1.000	thin retinal outer nuclear layer
702	79	4	2.39	0.2158	1.000	enlarged lymph nodes
715	83	4	2.38	0.2162	1.000	decreased thymocyte number
5154	32	2	0.87	0.2164	1.000	increased B cell proliferation
8688	38	3	1.61	0.2168	1.000	decreased interleukin-2 secretion
3674	31	2	0.89	0.2190	1.000	oxidative stress
445	66	4	2.43	0.2214	1.000	short snout
5238	18	2	0.95	0.2214	1.000	increased brain size
4983	53	3	1.67	0.2258	1.000	abnormal osteoclast cell number
4180	33	2	0.92	0.2272	1.000	failure of initiation of embryo turning
3059	54	3	1.66	0.2292	1.000	decreased insulin secretion
1449	269	18	14.94	0.2296	1.000	abnormal learning/ memory
1719	51	3	1.66	0.2304	1.000	absent vitelline blood vessels
2314	42	3	1.69	0.2308	1.000	abnormal respiratory mechanics
5094	187	7	5	0.2324	1.000	abnormal T cell proliferation
5079	27	2	0.9	0.2326	1.000	defective cytotoxic T cell cytolysis
2972	113	7	5.03	0.2346	1.000	abnormal cardiac muscle contractility
2835	34	2	0.92	0.2346	1.000	abnormal cranial suture morphology
343	45	3	1.7	0.2350	1.000	altered response to myocardial infarction
2998	90	5	3.34	0.2354	1.000	abnormal bone remodeling
4509	33	3	1.7	0.2368	1.000	abnormal pelvic girdle bone morphology
5092	78	4	2.51	0.2370	1.000	decreased double-positive T cell number
4883	35	2	0.93	0.2414	1.000	abnormal blood vessel healing
1314	26	2	0.97	0.2418	1.000	corneal opacity
4787	54	3	1.71	0.2436	1.000	abnormal dorsal aorta morphology
62	34	2	0.95	0.2438	1.000	increased bone density
5466	37	2	0.96	0.2448	1.000	abnormal T-helper 2 physiology
2406	257	9	6.91	0.2468	1.000	increased susceptibility to infection
5293	113	6	4.23	0.2470	1.000	impaired glucose tolerance
1869	42	2	0.95	0.2478	1.000	pancreas inflammation
149	24	2	0.96	0.2494	1.000	abnormal scapula morphology
1601	59	3	1.73	0.2504	1.000	abnormal myelopoiesis
5450	99	4	2.55	0.2506	1.000	abnormal energy expenditure
2169	1089	39	34.97	0.2514	1.000	no abnormal phenotype detected
3131	21	2	1	0.2514	1.000	increased erythrocyte cell number
1462	50	4	2.59	0.2516	1.000	abnormal avoidance learning behavior
8750	49	2	0.94	0.2520	1.000	abnormal interferon level
1636	32	3	1.79	0.2520	1.000	irregular heartbeat
790	17	2	1	0.2522	1.000	abnormal stratification in cerebral cortex
5358	57	3	1.73	0.2530	1.000	abnormal incisor morphology
5616	42	2	0.96	0.2530	1.000	decreased susceptibility to type IV hypersensitivity reaction
1957	16	2	0.99	0.2552	1.000	apnea
4196	634	21	17.85	0.2588	1.000	abnormal prenatal growth/weight/body size
2463	104	5	3.48	0.2588	1.000	abnormal neutrophil physiology
266	631	26	22.69	0.2592	1.000	abnormal heart morphology
8472	60	3	1.77	0.2604	1.000	abnormal spleen secondary B follicle morphology
8488	27	2	0.99	0.2604	1.000	abnormal semicircular canal ampulla morphology

8171	137	7	5.17	0.2612	1.000	abnormal mature B cell morphology
2224	344	14	11.52	0.2612	1.000	abnormal spleen size
1486	85	8	6.2	0.2616	1.000	abnormal startle reflex
3075	39	2	1	0.2616	1.000	altered response to CNS ischemic injury
4976	45	3	1.79	0.2628	1.000	abnormal B-1 B cell number
4940	45	3	1.79	0.2628	1.000	abnormal B-1 B cell morphology
9357	106	8	6.14	0.2630	1.000	abnormal seizure response to inducing agent
1716	110	5	3.46	0.2636	1.000	abnormal placenta labyrinth morphology
516	428	19	16.26	0.2640	1.000	abnormal urinary system morphology
8076	34	2	1.01	0.2640	1.000	abnormal CD4-positive T cell differentiation
63	84	5	3.52	0.2642	1.000	decreased bone density
2427	38	3	1.81	0.2652	1.000	disproportionate dwarf
3186	38	2	1.02	0.2660	1.000	abnormal redox activity
5407	30	3	1.8	0.2664	1.000	hyperalgesia
1265	1208	53	48.61	0.2670	1.000	decreased body size
501	26	2	1.01	0.2672	1.000	abnormal digestive secretion
1828	199	7	5.28	0.2678	1.000	abnormal T cell activation
3911	49	3	1.79	0.2680	1.000	increased drinking behavior
2062	157	11	8.9	0.2684	1.000	abnormal conditioning behavior
2873	1093	39	35.25	0.2688	1.000	normal phenotype
5244	26	2	1.01	0.2688	1.000	hemopericardium
8788	14	2	1.02	0.2688	1.000	abnormal fetal cardiomyocyte morphology
3410	80	4	2.63	0.2698	1.000	abnormal artery development
2531	37	2	1.01	0.2710	1.000	type I hypersensitivity reaction
4046	49	2	1.01	0.2716	1.000	abnormal mitosis
3231	81	4	2.64	0.2740	1.000	abnormal placenta vasculature
8188	24	2	1.04	0.2742	1.000	abnormal transitional stage B cell morphology
3809	30	2	1.03	0.2746	1.000	abnormal hair shaft morphology
4956	22	2	1.08	0.2748	1.000	decreased thymus weight
5465	37	2	1.04	0.2750	1.000	abnormal T-helper 1 physiology
738	128	8	6.25	0.2752	1.000	impaired muscle contractility
2327	54	4	2.72	0.2752	1.000	abnormal respiratory function
5432	52	3	1.82	0.2770	1.000	abnormal pro-B cell morphology
639	67	4	2.68	0.2784	1.000	abnormal adrenal gland morphology
952	109	5	3.56	0.2790	1.000	abnormal CNS glial cell morphology
854	68	7	5.37	0.2792	1.000	abnormal cerebellum development
1488	25	2	1.06	0.2802	1.000	increased startle reflex
2135	399	18	15.46	0.2806	1.000	abnormal kidney morphology
5667	106	4	2.69	0.2818	1.000	abnormal circulating leptin level
1496	9	2	1.09	0.2830	1.000	audiogenic seizures
8500	41	2	1.04	0.2836	1.000	increased IgG2a level
2633	31	3	1.87	0.2856	1.000	persistent truncus arteriosis
5150	69	5	3.57	0.2856	1.000	cachexia
4000	13	2	1.07	0.2860	1.000	impaired passive avoidance behavior
3446	27	2	1.07	0.2872	1.000	renal hypoplasia
1146	345	12	9.99	0.2880	1.000	abnormal testis morphology
1208	20	2	1.06	0.2894	1.000	blistering
709	37	2	1.08	0.2898	1.000	enlarged thymus
948	23	2	1.07	0.2904	1.000	nonconvulsive seizures
6104	26	2	1.09	0.2904	1.000	abnormal tectum morphology
6103	26	2	1.09	0.2904	1.000	abnormal midbrain roof plate morphology
899	26	2	1.09	0.2904	1.000	abnormal corpora quadrigemina morphology
3921	107	6	4.49	0.2910	1.000	abnormal heart left ventricle morphology
8595	46	2	1.07	0.2912	1.000	abnormal circulating interleukin-6 level
1219	43	2	1.1	0.2922	1.000	thickened epidermis
3054	38	2	1.06	0.2934	1.000	spina bifida
2417	120	5	3.62	0.2960	1.000	abnormal megakaryocyte morphology
5655	22	2	1.1	0.2970	1.000	increased aggression
4090	23	2	1.1	0.2978	1.000	abnormal sarcomere morphology
4392	65	3	1.94	0.2990	1.000	abnormal CD8-positive T cell physiology
2557	180	10	8.18	0.2994	1.000	abnormal social/conspecific interaction
5602	45	2	1.11	0.3004	1.000	decreased angiogenesis
5140	87	5	3.64	0.3008	1.000	decreased cardiac muscle contractility
1688	136	5	3.67	0.3018	1.000	abnormal somite development
6361	64	3	1.94	0.3020	1.000	abnormal female germ cell morphology
1125	64	3	1.94	0.3020	1.000	abnormal oocyte morphology
5165	48	3	1.92	0.3034	1.000	increased susceptibility to injury
1362	140	8	6.43	0.3034	1.000	abnormal anxiety-related response
116	37	2	1.1	0.3034	1.000	abnormal tooth development
1410	28	3	1.91	0.3036	1.000	head bobbing
1730	170	6	4.56	0.3062	1.000	embryonic growth arrest

4936	22	2	1.11	0.3068	1.000	abnormal ureteric bud branching morphogenesis
5010	136	6	4.6	0.3076	1.000	abnormal CD8-positive T cell morphology
565	23	2	1.11	0.3080	1.000	oligodactyly
4028	44	2	1.12	0.3090	1.000	chromosome breakage
2673	213	7	5.51	0.3092	1.000	abnormal sperm number
3733	37	3	1.97	0.3096	1.000	abnormal retinal inner nuclear layer morphology
272	140	6	4.59	0.3100	1.000	abnormal aorta morphology
3049	54	2	1.12	0.3104	1.000	abnormal lumbar vertebrae morphology
1700	106	4	2.85	0.3144	1.000	abnormal embryo turning
4874	37	2	1.14	0.3156	1.000	abnormal timing of postnatal eyelid opening
8801	42	2	1.12	0.3176	1.000	abnormal erythroid progenitor cell morphology
2183	60	3	1.98	0.3184	1.000	gliosis
6138	34	2	1.14	0.3198	1.000	congestive heart failure
2644	65	3	2	0.3200	1.000	decreased circulating triglyceride level
3720	176	7	5.59	0.3212	1.000	abnormal neural tube closure
5379	935	31	28.46	0.3226	1.000	endocrine/exocrine gland phenotype
4231	52	3	2	0.3226	1.000	abnormal calcium ion homeostasis
350	225	7	5.55	0.3244	1.000	abnormal cell proliferation
8770	119	6	4.69	0.3258	1.000	decreased survivor rate
8497	35	2	1.17	0.3262	1.000	decreased IgG2b level
5378	1961	74	70.24	0.3270	1.000	growth/size phenotype
2413	124	5	3.77	0.3274	1.000	abnormal megakaryocyte progenitor cell morphology
4816	33	2	1.17	0.3276	1.000	abnormal class switch recombination
8572	22	3	2.02	0.3282	1.000	abnormal Purkinje cell dendrite morphology
245	310	12	10.25	0.3284	1.000	abnormal erythropoiesis
2423	33	2	1.17	0.3292	1.000	abnormal mast cell physiology
2212	49	2	1.17	0.3292	1.000	abnormal secondary sex determination
1683	49	2	1.19	0.3300	1.000	absent mesoderm
9644	82	5	3.79	0.3314	1.000	uremia
5153	90	4	2.9	0.3314	1.000	abnormal B cell proliferation
2499	40	2	1.18	0.3314	1.000	chronic inflammation
4508	34	2	1.18	0.3316	1.000	abnormal pectoral girdle morphology
2064	211	12	10.3	0.3318	1.000	seizures
2497	45	2	1.18	0.3326	1.000	increased IgE level
5431	39	2	1.21	0.3338	1.000	decreased oocyte cell number
2124	280	11	9.4	0.3358	1.000	abnormal red blood cell
3634	159	7	5.68	0.3368	1.000	abnormal glial cell morphology
2441	201	7	5.69	0.3390	1.000	abnormal granulocyte morphology
8751	120	4	2.94	0.3398	1.000	abnormal interleukin level
288	177	8	6.59	0.3398	1.000	abnormal pericardium morphology
3984	287	10	8.44	0.3422	1.000	embryonic growth retardation
5217	64	3	2.08	0.3424	1.000	abnormal pancreatic beta cell morphology
9745	175	10	8.63	0.3426	1.000	abnormal behavioral response to xenobiotic
1212	48	2	1.21	0.3430	1.000	skin lesions
2230	43	2	1.24	0.3474	1.000	abnormal primitive streak formation
2625	36	3	2.08	0.3482	1.000	heart left ventricle hypertrophy
8043	47	2	1.23	0.3494	1.000	abnormal NK cell number
3675	57	4	3	0.3500	1.000	kidney cysts
76	130	6	4.8	0.3504	1.000	abnormal calvaria morphology
689	473	17	15.21	0.3508	1.000	abnormal spleen morphology
8077	117	5	3.89	0.3510	1.000	abnormal CD8-positive T cell number
220	39	2	1.23	0.3512	1.000	increased monocyte cell number
5184	30	2	1.22	0.3524	1.000	abnormal circulating progesterone level
1845	553	18	16.2	0.3532	1.000	abnormal inflammatory response
274	187	9	7.66	0.3534	1.000	enlarged heart
8071	34	2	1.22	0.3540	1.000	absent B cells
2795	30	2	1.23	0.3552	1.000	dilated cardiomyopathy
3149	25	2	1.25	0.3554	1.000	abnormal tectorial membrane morphology
8874	96	5	3.95	0.3566	1.000	decreased physiological sensitivity to xenobiotic
4945	25	2	1.25	0.3570	1.000	abnormal bone resorption
2534	57	2	1.24	0.3582	1.000	type IV hypersensitivity reaction
1906	21	2	1.27	0.3612	1.000	increased dopamine level
2061	48	3	2.14	0.3640	1.000	abnormal aggression-related behavior
3731	66	3	2.16	0.3642	1.000	abnormal retinal outer nuclear layer morphology
653	462	15	13.4	0.3648	1.000	abnormal sex gland morphology
2672	33	2	1.27	0.3654	1.000	abnormal branchial arch artery morphology
8415	118	6	4.91	0.3656	1.000	abnormal neurite morphology
432	397	15	13.5	0.3658	1.000	abnormal head morphology
4007	28	2	1.27	0.3658	1.000	abnormal lung vasculature morphology
5277	24	2	1.26	0.3658	1.000	abnormal brainstem morphology
1242	48	2	1.25	0.3660	1.000	hyperkeratosis

1898	47	4	3.06	0.3670	1.000	abnormal long term depression
3743	306	12	10.62	0.3682	1.000	abnormal facial morphology
9642	1171	40	37.76	0.3726	1.000	abnormal blood homeostasis
2651	27	2	1.27	0.3732	1.000	abnormal sciatic nerve
1776	17	2	1.29	0.3738	1.000	abnormal circulating sodium level
692	136	6	4.97	0.3744	1.000	small spleen
5319	132	6	4.98	0.3776	1.000	abnormal enzyme/ coenzyme level
5029	40	2	1.29	0.3786	1.000	abnormal amnion morphology
5377	420	22	20.34	0.3792	1.000	hearing/vestibular/ear phenotype
3953	503	19	17.5	0.3796	1.000	abnormal hormone level
1147	247	8	6.88	0.3806	1.000	small testis
334	81	3	2.24	0.3818	1.000	decreased granulocyte number
6319	42	2	1.32	0.3822	1.000	abnormal epididymal fat pad morphology
6395	113	5	4.05	0.3830	1.000	abnormal epiphyseal plate morphology
2602	55	2	1.31	0.3832	1.000	abnormal eosinophil cell number
2447	148	6	4.99	0.3832	1.000	abnormal erythrocyte morphology
4857	87	4	3.16	0.3836	1.000	abnormal heart weight
5620	172	10	8.8	0.3838	1.000	abnormal muscle contractility
5599	21	2	1.3	0.3860	1.000	increased cardiac muscle contractility
5591	17	2	1.3	0.3866	1.000	decreased vasodilation
2164	307	12	10.76	0.3868	1.000	abnormal gland physiology
1363	93	5	4.12	0.3870	1.000	increased anxiety-related response
5384	990	31	29.16	0.3874	1.000	cellular phenotype
1293	40	2	1.33	0.3882	1.000	anophthalmia
2089	1522	60	57.69	0.3902	1.000	abnormal postnatal growth/weight/body size
950	81	5	4.07	0.3904	1.000	abnormal seizure response to pharmacological agent
3140	35	2	1.34	0.3942	1.000	dilated atria
9746	39	2	1.35	0.3946	1.000	enhanced behavioral response to xenobiotic
1765	100	5	4.13	0.3946	1.000	abnormal ion homeostasis
2757	61	3	2.23	0.3946	1.000	decreased vertical activity
8566	59	2	1.35	0.3950	1.000	increased interferon-gamma secretion
2356	59	3	2.25	0.3962	1.000	abnormal spleen red pulp morphology
1778	47	2	1.36	0.3972	1.000	abnormal brown adipose tissue amount
5382	562	21	19.54	0.3984	1.000	craniofacial phenotype
428	562	21	19.54	0.3984	1.000	abnormal craniofacial morphology
1364	48	3	2.27	0.3984	1.000	decreased anxiety-related response
208	91	4	3.2	0.3986	1.000	decreased hematocrit
1259	1030	43	41.15	0.3998	1.000	abnormal body weight
726	62	3	2.28	0.3998	1.000	absent lymphocyte
4901	221	7	6.03	0.4008	1.000	decreased male germ cell number
2221	454	16	14.77	0.4020	1.000	abnormal lymph organ size
5031	87	3	2.26	0.4034	1.000	abnormal trophoblast layer morphology
358	200	6	5.1	0.4050	1.000	abnormal cell content/ morphology
443	89	4	3.23	0.4058	1.000	abnormal nose/snout morphology
5404	93	4	3.22	0.4068	1.000	abnormal axon morphology
1260	164	6	5.14	0.4076	1.000	increased body weight
8686	68	3	2.32	0.4086	1.000	abnormal interleukin-2 secretion
4322	37	2	1.36	0.4098	1.000	abnormal sternebra morphology
3564	74	3	2.3	0.4102	1.000	abnormal insulin secretion
5674	39	3	2.32	0.4112	1.000	abnormal outflow tract septation
1126	188	7	6.13	0.4128	1.000	abnormal ovary morphology
2416	67	3	2.32	0.4130	1.000	abnormal proerythroblast morphology
5291	178	7	6.13	0.4146	1.000	abnormal glucose tolerance
752	20	2	1.39	0.4152	1.000	dystrophic muscle
1928	56	3	2.32	0.4174	1.000	abnormal ovulation
3796	55	3	2.34	0.4178	1.000	abnormal cortical bone morphology
2706	141	6	5.2	0.4190	1.000	abnormal kidney size
5087	59	2	1.42	0.4192	1.000	decreased acute inflammation
5659	56	2	1.43	0.4196	1.000	increased resistance to diet-induced obesity
3938	67	3	2.34	0.4200	1.000	abnormal ear development
5241	50	3	2.33	0.4206	1.000	abnormal retinal ganglion layer morphology
5331	72	3	2.32	0.4208	1.000	insulin resistance
5464	80	3	2.37	0.4218	1.000	abnormal platelet physiology
1793	306	9	8.09	0.4220	1.000	altered susceptibility to infection
2408	104	4	3.34	0.4226	1.000	abnormal double-positive T cell morphology
9358	13	2	1.42	0.4228	1.000	environmentally induced seizures
1932	50	2	1.44	0.4230	1.000	abnormal spermiogenesis
8428	20	2	1.41	0.4232	1.000	abnormal spatial working memory
8947	44	2	1.42	0.4242	1.000	increased neuron number
4953	36	2	1.42	0.4250	1.000	decreased spleen weight
3797	58	3	2.37	0.4252	1.000	abnormal compact bone morphology

5312	42	2	1.44	0.4258	1.000	pericardial effusion
4272	25	2	1.46	0.4260	1.000	abnormal basement membrane morphology
4889	58	2	1.45	0.4262	1.000	increased energy expenditure
1264	167	6	5.25	0.4264	1.000	increased body size
165	70	3	2.4	0.4264	1.000	abnormal long bone hypertrophic chondrocyte zone
192	124	5	4.26	0.4284	1.000	abnormal mineral level
5061	61	2	1.45	0.4294	1.000	abnormal eosinophil morphology
5668	54	2	1.47	0.4302	1.000	decreased circulating leptin level
2925	432	17	15.98	0.4318	1.000	abnormal cardiovascular development
1119	322	12	11.05	0.4324	1.000	abnormal female reproductive system morphology
2081	721	28	26.9	0.4348	1.000	perinatal lethality
4201	54	2	1.46	0.4348	1.000	fetal growth retardation
3866	35	2	1.46	0.4352	1.000	abnormal defecation
2668	22	2	1.44	0.4352	1.000	abnormal circulating potassium level
5669	55	2	1.47	0.4360	1.000	increased circulating leptin level
2804	19	2	1.44	0.4360	1.000	abnormal motor learning
4859	27	2	1.47	0.4360	1.000	abnormal synaptic plasticity
202	28	2	1.45	0.4370	1.000	abnormal circulating alkaline phosphatase level
694	78	3	2.41	0.4374	1.000	spleen hypoplasia
4954	34	2	1.46	0.4382	1.000	abnormal thymus weight
3935	210	8	7.18	0.4384	1.000	abnormal craniofacial development
1322	44	2	1.48	0.4384	1.000	abnormal iris morphology
1577	186	7	6.31	0.4390	1.000	anemia
3008	35	3	2.42	0.4390	1.000	enhanced long term potentiation
2111	164	6	5.33	0.4392	1.000	abnormal tail morphology
2693	142	5	4.32	0.4398	1.000	abnormal pancreas physiology
2906	63	4	3.36	0.4410	1.000	increased susceptibility to pharmacologically induced seizures
5621	890	28	26.96	0.4424	1.000	abnormal cell physiology
2207	128	8	7.31	0.4446	1.000	abnormal long term potentiation
322	123	4	3.41	0.4458	1.000	increased granulocyte number
9764	48	2	1.5	0.4498	1.000	decreased sensitivity to induced morbidity/mortality
2753	52	2	1.49	0.4500	1.000	dilated heart left ventricle
6357	89	4	3.44	0.4506	1.000	abnormal circulating mineral level
1502	50	2	1.5	0.4510	1.000	abnormal circadian rhythm
2110	143	6	5.4	0.4516	1.000	abnormal digit morphology
1182	30	2	1.48	0.4526	1.000	lung hemorrhage
9205	392	12	11.23	0.4532	1.000	abnormal internal male genitalia morphology
8180	45	2	1.54	0.4538	1.000	abnormal marginal zone B cell morphology
5068	59	2	1.52	0.4540	1.000	abnormal NK cell morphology
2069	365	14	13.27	0.4590	1.000	abnormal eating/drinking behavior
8496	47	2	1.52	0.4596	1.000	decreased IgG2a level
1072	17	2	1.52	0.4598	1.000	abnormal vestibulocochlear nerve morphology
5351	96	3	2.52	0.4604	1.000	decreased susceptibility to autoimmune disorder
1431	312	12	11.36	0.4620	1.000	abnormal eating behavior
2703	120	5	4.46	0.4620	1.000	abnormal renal tubule morphology
5333	57	4	3.48	0.4632	1.000	decreased heart rate
8211	110	5	4.44	0.4642	1.000	decreased mature B cell number
1405	203	13	12.26	0.4654	1.000	impaired coordination
440	44	2	1.55	0.4656	1.000	domed skull
5636	131	5	4.46	0.4666	1.000	abnormal mineral homeostasis
2784	39	2	1.55	0.4668	1.000	abnormal Sertoli cell morphology
4200	100	3	2.53	0.4670	1.000	decreased fetal size
1243	58	2	1.56	0.4670	1.000	abnormal dermal layer morphology
3111	75	2	1.55	0.4672	1.000	abnormal nucleus morphology
9209	248	9	8.37	0.4676	1.000	abnormal internal female genitalia morphology
934	70	3	2.53	0.4678	1.000	abnormal telencephalon development
1216	153	5	4.49	0.4682	1.000	abnormal epidermal layer morphology
4994	32	2	1.58	0.4682	1.000	abnormal brain wave pattern
2073	134	6	5.45	0.4688	1.000	abnormal hair growth
1968	161	8	7.41	0.4694	1.000	abnormal touch/ nociception
130	105	4	3.52	0.4694	1.000	abnormal cancellous bone morphology
8083	139	5	4.49	0.4700	1.000	decreased single-positive T cell number
88	44	2	1.56	0.4700	1.000	short mandible
3156	55	2	1.56	0.4708	1.000	abnormal leukocyte migration
1806	56	2	1.58	0.4716	1.000	decreased IgM level
5194	48	2	1.58	0.4728	1.000	abnormal anterior uvea morphology
701	116	4	3.49	0.4732	1.000	abnormal lymph node size
2023	26	2	1.47	0.4736	1.000	B cell derived lymphoma
3562	82	3	2.55	0.4762	1.000	abnormal pancreatic beta cell physiology
2269	65	3	2.57	0.4768	1.000	muscular atrophy
2059	61	2	1.59	0.4770	1.000	abnormal seminal vesicle morphology

4271	174	7	6.54	0.4770	1.000	abnormal paw/hand/foot morphology
1297	88	3	2.55	0.4772	1.000	microphthalmia
2551	153	5	4.54	0.4790	1.000	abnormal blood coagulation
9676	153	5	4.54	0.4790	1.000	abnormal hemostasis
1876	56	2	1.59	0.4796	1.000	decreased inflammatory response
1648	268	8	7.53	0.4800	1.000	abnormal apoptosis
74	164	7	6.52	0.4814	1.000	abnormal neurocranium morphology
6412	52	2	1.6	0.4816	1.000	abnormal T cell apoptosis
1183	30	2	1.58	0.4824	1.000	overexpanded respiratory alveoli
3651	32	2	1.61	0.4824	1.000	abnormal axon outgrowth
2694	84	3	2.57	0.4826	1.000	abnormal pancreas secretion
1663	406	15	14.48	0.4842	1.000	abnormal digestive system physiology
1926	183	6	5.51	0.4844	1.000	female infertility
8535	32	2	1.6	0.4854	1.000	enlarged lateral ventricles
609	201	6	5.6	0.4866	1.000	abnormal liver physiology
1931	84	3	2.59	0.4866	1.000	abnormal oogenesis
3	350	12	11.52	0.4872	1.000	abnormal adipose tissue morphology
5375	352	12	11.54	0.4886	1.000	adipose tissue phenotype
2746	37	2	1.63	0.4902	1.000	abnormal semilunar valve morphology
1422	78	3	2.6	0.4906	1.000	abnormal drinking behavior
2460	166	5	4.62	0.4912	1.000	decreased immunoglobulin level
9198	405	12	11.53	0.4920	1.000	abnormal male genitalia morphology
1145	405	12	11.53	0.4920	1.000	abnormal male reproductive system morphology
778	92	5	4.58	0.4920	1.000	abnormal tract
3077	136	3	2.64	0.4924	1.000	abnormal cell cycle
9208	253	9	8.53	0.4930	1.000	abnormal female genitalia morphology
2115	270	10	9.63	0.4940	1.000	abnormal skeleton extremities morphology
4849	264	8	7.6	0.4950	1.000	abnormal testis size
5318	86	3	2.65	0.4956	1.000	decreased triglyceride level
9538	62	3	2.6	0.4958	1.000	abnormal synapse morphology
2082	774	32	31.6	0.4960	1.000	postnatal lethality
2357	149	5	4.62	0.4962	1.000	abnormal spleen white pulp morphology
8470	81	3	2.62	0.4968	1.000	abnormal spleen B cell follicle morphology
3711	40	2	1.63	0.4992	1.000	pathological neovascularization
1262	907	38	37.53	0.4994	1.000	decreased body weight
5332	58	2	1.67	0.5010	1.000	abnormal amino acid level
4982	82	3	2.67	0.5032	1.000	abnormal osteoclast morphology
857	43	5	4.6	0.5034	1.000	abnormal cerebellar foliation
1680	107	3	2.68	0.5060	1.000	abnormal mesoderm development
2745	34	2	1.67	0.5064	1.000	abnormal atrioventricular valve morphology
1065	27	2	1.64	0.5094	1.000	abnormal trigeminal nerve morphology
4198	107	3	2.71	0.5130	1.000	abnormal fetal size
9964	45	5	4.65	0.5138	1.000	abnormal cerebellum lobule morphology
1570	92	4	3.72	0.5176	1.000	abnormal circulating enzyme level
8056	32	2	1.67	0.5186	1.000	abnormal retinal ganglion cell morphology
2697	91	3	2.72	0.5190	1.000	abnormal eye size
2102	285	12	11.76	0.5198	1.000	abnormal ear morphology
830	174	6	5.76	0.5202	1.000	abnormal diencephalon morphology
5078	53	2	1.7	0.5202	1.000	abnormal cytotoxic T cell physiology
2566	61	3	2.73	0.5254	1.000	abnormal sexual interaction
2362	58	2	1.75	0.5260	1.000	abnormal spleen marginal zone morphology
3878	290	16	15.95	0.5266	1.000	abnormal ear physiology
1124	255	7	6.81	0.5266	1.000	abnormal gametes
5023	57	2	1.73	0.5274	1.000	abnormal wound healing
2596	107	4	3.77	0.5280	1.000	abnormal hematocrit
2209	252	7	6.81	0.5284	1.000	decreased germ cell number
8075	86	3	2.76	0.5286	1.000	decreased CD4-positive T cell number
5334	128	4	3.78	0.5288	1.000	abnormal fat pad morphology
703	237	9	8.83	0.5302	1.000	abnormal thymus morphology
2359	58	2	1.73	0.5316	1.000	abnormal spleen germinal center morphology
313	422	12	11.92	0.5318	1.000	abnormal cell death
1429	58	2	1.74	0.5334	1.000	dehydration
137	230	8	7.79	0.5342	1.000	abnormal vertebrae morphology
458	93	3	2.8	0.5352	1.000	abnormal mandible morphology
5169	88	2	1.78	0.5354	1.000	abnormal male meiosis
1915	56	2	1.77	0.5360	1.000	intracranial hemorrhage
9250	283	10	9.93	0.5368	1.000	abnormal appendicular skeleton morphology
281	127	5	4.82	0.5372	1.000	abnormal ventricular septum morphology
1545	152	5	4.85	0.5372	1.000	abnormal hematopoietic system physiology
8803	57	2	1.76	0.5384	1.000	abnormal placental labyrinth vasculature morphology
34	62	3	2.8	0.5394	1.000	abnormal vestibule morphology

2168	81	4	3.82	0.5430	1.000	other aberrant phenotype
1153	51	2	1.74	0.5432	1.000	small seminiferous tubules
5065	111	3	2.84	0.5436	1.000	abnormal neutrophil morphology
2136	250	8	7.97	0.5440	1.000	abnormal kidney physiology
693	59	2	1.8	0.5448	1.000	spleen hyperplasia
267	254	10	9.94	0.5454	1.000	abnormal heart development
3055	105	4	3.84	0.5474	1.000	abnormal long bone epiphyseal plate morphology
1515	64	3	2.84	0.5476	1.000	abnormal grip strength
3048	87	3	2.8	0.5478	1.000	abnormal cervical vertebrae morphology
5502	330	11	11.06	0.5480	1.000	abnormal urinary system physiology
1586	100	4	3.85	0.5484	1.000	abnormal erythrocyte cell number
8284	25	2	1.77	0.5486	1.000	abnormal hippocampus pyramidal cell layer
2199	79	4	3.87	0.5488	1.000	abnormal brain commissure morphology
1051	59	3	2.83	0.5510	1.000	abnormal somatic motor system morphology
6307	52	2	1.77	0.5530	1.000	abnormal seminiferous tubule size
2722	625	20	20.18	0.5534	1.000	abnormal immune system organ morphology
1846	426	13	13.08	0.5542	1.000	increased inflammatory response
545	465	16	16.18	0.5548	1.000	abnormal limbs/digits/tail morphology
5371	465	16	16.18	0.5548	1.000	limbs/digits/tail phenotype
1053	42	2	1.8	0.5558	1.000	abnormal neuromuscular synapse morphology
2428	48	2	1.82	0.5560	1.000	abnormal semicircular canal
812	41	2	1.8	0.5560	1.000	abnormal dentate gyrus morphology
352	163	4	3.96	0.5564	1.000	decreased cell proliferation
2364	177	6	6	0.5592	1.000	abnormal thymus size
4087	195	8	8.02	0.5592	1.000	abnormal muscle fiber morphology
5606	58	2	1.84	0.5602	1.000	increased bleeding time
2160	705	21	21.25	0.5620	1.000	abnormal reproductive system morphology
788	140	6	5.99	0.5636	1.000	abnormal cerebral cortex morphology
5392	212	9	9.1	0.5640	1.000	touch/vibrissae phenotype
5373	804	32	32.48	0.5640	1.000	lethality-postnatal
102	42	2	1.83	0.5642	1.000	abnormal nasal bone morphology
9937	147	6	6.02	0.5646	1.000	abnormal neuron differentiation
706	141	5	4.99	0.5648	1.000	small thymus
5369	748	29	29.43	0.5656	1.000	muscle phenotype
1523	66	4	3.87	0.5666	1.000	impaired righting response
3169	141	7	7.04	0.5668	1.000	abnormal scala media morphology
3936	137	5	4.97	0.5668	1.000	abnormal reproductive system development
2675	99	3	2.94	0.5678	1.000	asthenozoospermia
5598	45	2	1.85	0.5688	1.000	decreased ventricle muscle contractility
1914	298	10	10.22	0.5694	1.000	hemorrhage
5425	60	2	1.88	0.5696	1.000	increased macrophage cell number
1925	322	10	10.15	0.5702	1.000	male infertility
2108	497	18	18.42	0.5712	1.000	abnormal muscle morphology
1463	122	7	7.05	0.5716	1.000	abnormal spatial learning
1402	183	7	7.08	0.5716	1.000	hypoactivity
6205	241	6	6.1	0.5752	1.000	embryonic lethality before somite formation
5418	438	14	14.31	0.5752	1.000	abnormal circulating hormone level
134	41	2	1.86	0.5768	1.000	abnormal cortical bone thickness
2189	93	4	4	0.5774	1.000	abnormal myocardial trabeculae morphology
1240	69	2	1.89	0.5778	1.000	abnormal epidermis stratum corneum morphology
534	42	2	1.87	0.5780	1.000	abnormal ureter morphology
2971	64	2	1.93	0.5788	1.000	abnormal brown adipose tissue morphology
4408	31	2	1.88	0.5788	1.000	decreased cochlear hair cell number
2729	51	2	1.89	0.5804	1.000	abnormal inner ear canal morphology
2139	221	6	6.2	0.5814	1.000	abnormal liver/biliary system physiology
2797	24	2	1.84	0.5828	1.000	increased thigmotaxis
5578	100	3	3.01	0.5846	1.000	teratozoospermia
2106	466	19	19.53	0.5880	1.000	abnormal muscle physiology
2083	602	25	25.52	0.5884	1.000	premature death
2339	178	5	5.11	0.5900	1.000	abnormal lymph node morphology
1613	44	2	1.92	0.5914	1.000	abnormal vasodilation
1326	56	2	1.94	0.5916	1.000	retinal degeneration
1386	80	4	4.06	0.5926	1.000	abnormal maternal nurturing
3861	610	23	23.71	0.5958	1.000	abnormal nervous system development
9939	29	2	1.91	0.5968	1.000	abnormal hippocampus neuron morphology
240	74	2	1.99	0.5978	1.000	extramedullary hematopoiesis
4197	150	4	4.12	0.5984	1.000	abnormal fetal growth/weight/body size
3920	102	4	4.09	0.5990	1.000	abnormal heart right ventricle morphology
1923	168	7	7.29	0.6008	1.000	reduced female fertility
875	102	6	6.22	0.6020	1.000	abnormal cerebellar Purkinje cell layer
5306	48	2	1.98	0.6036	1.000	abnormal phalanx morphology

780	59	3	3.05	0.6040	1.000	abnormal corpus callosum morphology
60	71	2	2.01	0.6052	1.000	delayed bone ossification
2843	57	2	2.03	0.6068	1.000	decreased blood pressure
8495	67	2	2.02	0.6072	1.000	decreased IgG1 level
4599	61	2	1.99	0.6082	1.000	abnormal vertebral arch morphology
6113	137	5	5.2	0.6112	1.000	abnormal heart septum morphology
6206	93	2	2.07	0.6120	1.000	embryonic lethality before turning of embryo
1436	84	3	3.1	0.6128	1.000	abnormal suckling behavior
8892	57	2	2.01	0.6132	1.000	abnormal sperm flagellum morphology
4937	107	4	4.16	0.6138	1.000	dilated heart
2113	338	12	12.59	0.6148	1.000	abnormal skeleton development
6004	64	3	3.06	0.6170	1.000	abnormal interneuron morphology
8219	62	3	3.11	0.6178	1.000	abnormal dorsal telencephalic commissure morphology
26	183	8	8.44	0.6196	1.000	abnormal inner ear morphology
740	22	2	1.93	0.6210	1.000	impaired smooth muscle contractility
4686	112	4	4.21	0.6226	1.000	decreased length of long bones
1588	96	3	3.12	0.6226	1.000	abnormal hemoglobin
1823	98	3	3.19	0.6230	1.000	thymus hypoplasia
913	381	14	14.74	0.6230	1.000	abnormal brain development
1575	140	5	5.28	0.6246	1.000	cyanosis
2128	377	13	13.72	0.6248	1.000	abnormal blood circulation
5201	58	2	2.06	0.6264	1.000	abnormal retinal pigment epithelium morphology
4852	127	3	3.2	0.6286	1.000	decreased testis weight
885	18	2	1.97	0.6318	1.000	ectopic Purkinje cell
31	154	7	7.49	0.6320	1.000	abnormal cochlea morphology
2884	93	3	3.19	0.6322	1.000	abnormal branchial arch morphology
2912	48	3	3.17	0.6336	1.000	abnormal excitatory postsynaptic potential
2409	93	2	2.14	0.6346	1.000	decreased susceptibility to infection
2376	84	2	2.15	0.6370	1.000	abnormal dendritic cell physiology
8779	84	4	4.29	0.6388	1.000	abnormal maternal behavior
5449	217	7	7.58	0.6392	1.000	abnormal food intake
5316	52	3	3.2	0.6398	1.000	abnormal response to tactile stimuli
2915	57	4	4.22	0.6402	1.000	abnormal synaptic depression
1756	70	3	3.19	0.6420	1.000	abnormal urination
2855	68	3	3.23	0.6424	1.000	abnormal cochlear ganglion morphology
628	82	3	3.24	0.6438	1.000	abnormal mammary gland development
5367	570	20	21.25	0.6448	1.000	renal/urinary system phenotype
1664	68	3	3.23	0.6478	1.000	abnormal digestion
2862	76	4	4.29	0.6480	1.000	altered righting response
5215	99	3	3.27	0.6480	1.000	abnormal pancreatic islet morphology
1943	320	13	13.83	0.6504	1.000	abnormal respiration
9165	100	3	3.29	0.6526	1.000	abnormal endocrine pancreas morphology
1944	141	4	4.4	0.6530	1.000	abnormal pancreas morphology
3635	351	21	22.22	0.6544	1.000	abnormal synaptic transmission
1951	169	7	7.59	0.6566	1.000	abnormal breathing
3699	420	14	15	0.6576	1.000	abnormal female reproductive system physiology
9674	46	2	2.15	0.6582	1.000	decreased birth weight
5200	64	2	2.17	0.6586	1.000	abnormal pigment epithelium of the eye
819	73	3	3.29	0.6588	1.000	abnormal olfactory bulb morphology
9850	312	7	7.73	0.6592	1.000	embryonic lethality between implantation and placentation
5406	257	9	9.81	0.6608	1.000	abnormal heart size
2628	104	3	3.36	0.6626	1.000	hepatic steatosis
4406	44	2	2.18	0.6630	1.000	abnormal cochlear hair cell number
6069	158	6	6.63	0.6636	1.000	abnormal retinal neuronal layer morphology
5452	277	8	8.83	0.6652	1.000	abnormal adipose tissue amount
2231	79	2	2.22	0.6658	1.000	abnormal primitive streak morphology
2315	173	7	7.69	0.6682	1.000	abnormal respiratory signs/symptoms
9819	48	2	2.17	0.6700	1.000	abnormal circulating androgen level
5180	48	2	2.17	0.6700	1.000	abnormal circulating testosterone level
2109	310	10	11.06	0.6702	1.000	abnormal limb morphology
2498	99	2	2.29	0.6714	1.000	abnormal acute inflammation
9672	47	2	2.22	0.6726	1.000	abnormal birth weight
3744	251	8	8.84	0.6728	1.000	abnormal orofacial morphology
1177	50	2	2.23	0.6736	1.000	atelectasis
1120	107	4	4.48	0.6740	1.000	abnormal uterus morphology
1312	79	2	2.27	0.6748	1.000	abnormal cornea morphology
849	186	9	9.93	0.6760	1.000	abnormal cerebellum morphology
1930	101	2	2.31	0.6774	1.000	abnormal meiosis
2058	507	18	19.43	0.6776	1.000	neonatal lethality
3115	76	3	3.38	0.6792	1.000	abnormal respiratory system development
9944	78	3	3.41	0.6794	1.000	abnormal olfactory lobe morphology

2980	82	4	4.46	0.6798	1.000	abnormal postural reflex
2945	40	2	2.21	0.6814	1.000	abnormal inhibitory postsynaptic currents
5370	597	16	17.5	0.6816	1.000	liver/biliary system phenotype
6325	113	5	5.63	0.6830	1.000	impaired hearing
8026	99	4	4.57	0.6840	1.000	abnormal brain white matter morphology
592	61	2	2.25	0.6840	1.000	short tail
436	67	3	3.44	0.6846	1.000	abnormal head movements
2272	172	9	10.03	0.6854	1.000	abnormal nervous system electrophysiology
1175	272	9	10.09	0.6868	1.000	abnormal lung morphology
2371	34	2	2.23	0.6870	1.000	abnormal thymus cortex morphology
1970	131	5	5.67	0.6884	1.000	abnormal pain threshold
1340	133	4	4.62	0.6890	1.000	abnormal eyelid morphology
8872	254	10	11.08	0.6898	1.000	abnormal physiological response to xenobiotic
847	190	9	10.08	0.6904	1.000	abnormal metencephalon morphology
1967	75	3	3.49	0.6906	1.000	deafness
5595	75	3	3.44	0.6908	1.000	abnormal vascular smooth muscle physiology
3723	212	7	7.92	0.6910	1.000	abnormal long bone morphology
1330	50	2	2.29	0.6930	1.000	abnormal optic nerve morphology
2068	90	4	4.59	0.6936	1.000	abnormal parental behavior
3633	975	44	46.7	0.6948	1.000	abnormal nervous system physiology
5395	116	4	4.67	0.6950	1.000	other phenotype
4811	136	7	7.9	0.6954	1.000	abnormal neuron physiology
599	107	3	3.51	0.6956	1.000	enlarged liver
1533	218	7	7.92	0.6962	1.000	abnormal skeleton physiology
627	94	3	3.5	0.6966	1.000	abnormal mammary gland morphology
9931	267	7	8.02	0.6966	1.000	abnormal skin appearance
1905	49	2	2.29	0.6978	1.000	abnormal dopamine level
3232	102	3	3.5	0.6984	1.000	abnormal forebrain development
4425	51	2	2.32	0.6992	1.000	abnormal otolith organ
5563	81	2	2.36	0.6992	1.000	abnormal hemoglobin content
807	145	7	7.92	0.7010	1.000	abnormal hippocampus morphology
4848	213	6	6.9	0.7012	1.000	abnormal liver size
2734	62	3	3.51	0.7034	1.000	abnormal mechanical nociception
5202	66	2	2.4	0.7042	1.000	lethargy
2674	112	3	3.55	0.7056	1.000	abnormal sperm motility
877	93	5	5.75	0.7066	1.000	abnormal Purkinje cell morphology
6126	81	3	3.6	0.7090	1.000	abnormal outflow tract development
163	203	7	8.06	0.7098	1.000	abnormal cartilage morphology
920	92	3	3.56	0.7100	1.000	abnormal myelination
2896	77	3	3.52	0.7106	1.000	abnormal bone mineralization
601	111	3	3.57	0.7118	1.000	small liver
9956	122	6	6.91	0.7132	1.000	abnormal cerebellar layer morphology
4036	31	2	2.23	0.7134	1.000	abnormal muscle relaxation
1454	48	3	3.5	0.7154	1.000	abnormal cued conditioning behavior
2891	77	2	2.45	0.7176	1.000	increased insulin sensitivity
897	77	3	3.51	0.7188	1.000	abnormal midbrain morphology
4085	129	5	5.91	0.7196	1.000	abnormal heartbeat
280	63	2	2.42	0.7212	1.000	thin ventricular wall
1176	60	2	2.46	0.7226	1.000	abnormal lung development
1963	181	8	9.23	0.7240	1.000	abnormal hearing physiology
3631	1762	69	73.16	0.7256	1.000	nervous system phenotype
813	32	2	2.38	0.7256	1.000	abnormal hippocampus layer morphology
9661	88	3	3.63	0.7260	1.000	abnormal pregnancy
1924	465	13	14.79	0.7268	1.000	infertility
5559	113	3	3.74	0.7290	1.000	increased circulating glucose level
4021	48	2	2.45	0.7290	1.000	abnormal rod electrophysiology
771	104	4	4.83	0.7308	1.000	abnormal brain size
297	44	2	2.42	0.7314	1.000	abnormal endocardial cushion morphology
1574	158	5	5.95	0.7318	1.000	abnormal oxygen level
2910	48	4	4.69	0.7318	1.000	abnormal excitatory postsynaptic currents
3105	106	4	4.87	0.7324	1.000	abnormal heart atrium morphology
8489	74	2	2.54	0.7330	1.000	postnatal slow weight gain
1805	134	3	3.77	0.7332	1.000	decreased IgG level
6362	302	7	8.33	0.7338	1.000	abnormal male germ cell morphology
5389	1078	33	35.94	0.7344	1.000	reproductive system phenotype
556	140	4	4.93	0.7348	1.000	abnormal hindlimb morphology
889	32	2	2.5	0.7372	1.000	abnormal cerebellar molecular layer
961	73	2	2.55	0.7374	1.000	abnormal dorsal root ganglion morphology
633	76	2	2.53	0.7400	1.000	abnormal pituitary gland morphology
5274	178	5	6.05	0.7406	1.000	abnormal viscerocranium morphology
5197	74	2	2.58	0.7412	1.000	abnormal uvea morphology

1510	108	3	3.78	0.7416	1.000	abnormal coat appearance
2208	347	8	9.54	0.7416	1.000	abnormal germ cell morphology
801	150	7	8.27	0.7444	1.000	abnormal temporal lobe morphology
2078	382	11	12.87	0.7478	1.000	abnormal glucose homeostasis
2177	75	2	2.64	0.7490	1.000	abnormal outer ear morphology
4850	137	3	3.82	0.7524	1.000	abnormal testis weight
1473	69	3	3.81	0.7526	1.000	reduced long term potentiation
2163	816	22	24.57	0.7564	1.000	abnormal gland morphology
4097	143	7	8.4	0.7566	1.000	abnormal cerebellar cortex morphology
947	89	4	5	0.7608	1.000	convulsive seizures
285	69	2	2.72	0.7608	1.000	abnormal cardiac valve morphology
2705	55	2	2.61	0.7614	1.000	dilated renal tubules
1388	111	5	6.16	0.7626	1.000	abnormal stationary movement
2206	296	17	19.36	0.7650	1.000	abnormal CNS synaptic transmission
2133	459	16	18.4	0.7652	1.000	abnormal respiratory system physiology
4543	130	3	3.94	0.7654	1.000	abnormal sperm physiology
823	69	2	2.67	0.7658	1.000	abnormal lateral ventricle morphology
189	64	2	2.64	0.7684	1.000	hypoglycemia
8022	73	2	2.68	0.7690	1.000	dilated heart ventricles
960	155	5	6.29	0.7714	1.000	abnormal sensory ganglion morphology
631	81	2	2.73	0.7790	1.000	abnormal neuroendocrine gland morphology
2200	149	4	5.26	0.7818	1.000	abnormal brain ventricle/choroid plexus morphology
1324	83	2	2.78	0.7860	1.000	abnormal eye pigmentation
5508	772	24	27.41	0.7866	1.000	abnormal skeleton morphology
452	246	7	8.7	0.7882	1.000	abnormal mouth morphology
5386	1478	57	62.02	0.7886	1.000	behavior/neurological phenotype
4924	1478	57	62.02	0.7886	1.000	abnormal behavior
1469	68	3	4.02	0.7902	1.000	abnormal contextual conditioning behavior
8079	84	2	2.87	0.7914	1.000	decreased CD8-positive T cell number
5195	293	10	12.16	0.7916	1.000	abnormal posterior eye segment morphology
1629	104	4	5.29	0.7920	1.000	abnormal heart rate
558	72	2	2.9	0.7968	1.000	abnormal tibia morphology
6042	163	4	5.24	0.7972	1.000	increased apoptosis
4847	88	2	2.87	0.7992	1.000	abnormal liver weight
3727	189	6	7.74	0.7996	1.000	abnormal retinal layer morphology
5372	892	31	34.94	0.8012	1.000	life span-post-weaning/aging
4166	176	7	8.82	0.8026	1.000	abnormal limbic system morphology
1263	184	6	7.68	0.8034	1.000	weight loss
6335	142	6	7.72	0.8046	1.000	abnormal hearing electrophysiology
9389	87	2	2.9	0.8056	1.000	abnormal extracutaneous pigmentation
2405	102	2	2.97	0.8058	1.000	respiratory system inflammation
559	80	2	2.98	0.8068	1.000	abnormal femur morphology
5390	824	25	28.82	0.8070	1.000	skeleton phenotype
3698	469	13	15.62	0.8096	1.000	abnormal male reproductive system physiology
841	232	10	12.39	0.8124	1.000	abnormal hindbrain morphology
438	295	9	11.28	0.8148	1.000	abnormal skull morphology
2067	415	19	22.32	0.8152	1.000	abnormal sensory capabilities/reflexes/nociception
188	241	6	7.95	0.8168	1.000	abnormal circulating glucose level
5442	46	2	2.85	0.8178	1.000	abnormal pyramidal neuron morphology
4262	123	4	5.51	0.8182	1.000	abnormal physical strength
1468	76	3	4.25	0.8196	1.000	abnormal temporal memory
1961	265	13	15.66	0.8200	1.000	abnormal reflex
1408	101	4	5.41	0.8230	1.000	stereotypic behavior
1056	126	5	6.63	0.8240	1.000	abnormal cranial nerve morphology
1156	339	7	9.24	0.8260	1.000	abnormal spermatogenesis
5560	130	3	4.34	0.8286	1.000	decreased circulating glucose level
1522	52	3	4.09	0.8304	1.000	impaired swimming
1407	26	2	2.68	0.8304	1.000	short stride length
2367	48	2	2.94	0.8316	1.000	abnormal thymus lobule morphology
164	101	3	4.33	0.8322	1.000	abnormal cartilage development
1399	163	7	9.18	0.8328	1.000	hyperactivity
2727	112	2	3.17	0.8338	1.000	decreased circulating insulin level
1657	268	7	9.28	0.8346	1.000	abnormal induced morbidity/mortality
3632	1399	50	56.06	0.8360	1.000	abnormal nervous system morphology
2116	306	9	11.55	0.8360	1.000	abnormal craniofacial bone morphology
414	64	2	2.94	0.8412	1.000	alopecia
3313	476	17	20.71	0.8422	1.000	abnormal locomotor activation
5454	92	2	3.19	0.8430	1.000	decreased adipose tissue amount
454	139	3	4.54	0.8480	1.000	abnormal jaw morphology
787	376	13	16.27	0.8486	1.000	abnormal telencephalon morphology
1784	306	7	9.59	0.8510	1.000	abnormal fluid regulation

2132	371	10	13.12	0.8532	1.000	abnormal respiratory system morphology
4765	97	3	4.61	0.8536	1.000	decreased brainstem auditory evoked potential
1560	201	4	5.93	0.8540	1.000	abnormal circulating insulin level
8873	117	3	4.53	0.8558	1.000	increased physiological sensitivity to xenobiotic
550	90	2	3.34	0.8584	1.000	abnormal forelimb morphology
5388	644	20	24.44	0.8598	1.000	respiratory system phenotype
157	126	3	4.66	0.8608	1.000	abnormal sternum morphology
1325	265	8	10.8	0.8624	1.000	abnormal retina morphology
2060	451	10	13.39	0.8632	1.000	abnormal skin morphology
774	82	2	3.33	0.8638	1.000	decreased brain size
783	499	16	20.15	0.8682	1.000	abnormal forebrain morphology
4742	105	4	5.89	0.8688	1.000	abnormal vestibular system physiology
5391	650	19	23.49	0.8722	1.000	vision/eye phenotype
966	100	3	4.77	0.8726	1.000	decreased sensory neuron number
5273	91	2	3.43	0.8752	1.000	abnormal cranial base morphology
9643	203	4	6.15	0.8760	1.000	abnormal urine homeostasis
2216	192	4	6.15	0.8764	1.000	abnormal seminiferous tubule morphology
2092	594	17	21.37	0.8778	1.000	abnormal eye morphology
5551	123	4	6.04	0.8784	1.000	abnormal eye electrophysiology
2864	272	8	11.03	0.8786	1.000	abnormal ocular fundus morphology
9765	78	2	3.42	0.8794	1.000	abnormal xenobiotic induced morbidity/mortality
9763	204	5	7.4	0.8796	1.000	increased sensitivity to induced morbidity/mortality
5253	219	6	8.75	0.8812	1.000	abnormal eye physiology
914	122	2	3.57	0.8820	1.000	exencephaly
2152	820	28	33.67	0.8828	1.000	abnormal brain morphology
4738	127	4	6.24	0.8830	1.000	abnormal brainstem auditory evoked potential
2979	93	3	4.73	0.8836	1.000	abnormal vestibular response
2733	80	2	3.53	0.8848	1.000	abnormal thermal nociception
1929	414	8	11.35	0.8852	1.000	abnormal gametogenesis
3492	360	15	19.05	0.8854	1.000	abnormal involuntary movement
5193	195	4	6.35	0.8882	1.000	abnormal anterior eye segment morphology
3308	120	4	6.22	0.8898	1.000	abnormal cochlear sensory epithelium morphology
1731	500	14	18.21	0.8900	1.000	abnormal postnatal growth
1732	485	13	17.15	0.8906	1.000	postnatal growth retardation
1954	128	4	6.24	0.8908	1.000	respiratory distress
3717	124	2	3.7	0.8914	1.000	pallor
886	49	2	3.53	0.8918	1.000	abnormal cerebellar granule layer
42	121	4	6.26	0.8922	1.000	abnormal organ of Corti
1303	108	2	3.76	0.8960	1.000	abnormal lens morphology
45	123	4	6.36	0.8980	1.000	abnormal hair cell morphology
4703	326	9	12.55	0.8980	1.000	abnormal vertebral column
852	64	3	4.96	0.9004	1.000	small cerebellum
1935	195	4	6.32	0.9012	1.000	decreased litter size
5501	137	2	3.81	0.9040	1.000	abnormal skin physiology
1081	111	3	5.08	0.9086	1.000	abnormal cranial ganglia morphology
1785	211	4	6.74	0.9094	1.000	edema
937	92	2	3.8	0.9100	1.000	abnormal motor neuron morphology
1933	203	4	6.55	0.9142	1.000	abnormal litter size
1919	893	24	30.22	0.9144	1.000	abnormal reproductive system physiology
5535	104	2	3.9	0.9194	1.000	abnormal body temperature
9703	85	2	3.94	0.9196	1.000	decreased birth body size
1516	445	18	23.32	0.9204	1.000	abnormal motor coordination/ balance
8540	262	9	12.94	0.9220	1.000	abnormal cerebrum morphology
4174	145	4	6.79	0.9234	1.000	abnormal spine curvature
822	111	2	4.06	0.9240	1.000	abnormal brain ventricle morphology
972	136	4	6.84	0.9260	1.000	abnormal mechanoreceptor morphology
2020	267	5	8.2	0.9276	1.000	increased tumor incidence
9701	87	2	4.09	0.9284	1.000	abnormal birth body size
367	297	7	10.86	0.9310	1.000	abnormal coat/ hair morphology
1131	125	2	4.13	0.9316	1.000	abnormal ovarian follicle morphology
598	476	10	14.85	0.9320	1.000	abnormal liver morphology
5402	68	2	4.05	0.9320	1.000	abnormal action potential
1394	70	2	3.88	0.9330	1.000	circling
57	156	3	5.66	0.9357	1.000	abnormal osteogenesis
2114	525	14	19.39	0.9365	1.000	abnormal axial skeleton morphology
8503	115	2	4.31	0.9367	1.000	abnormal spinal cord grey matter morphology
4624	213	4	7.28	0.9381	1.000	abnormal thoracic cage
2184	148	5	8.36	0.9389	1.000	abnormal innervation
3948	245	5	8.41	0.9397	1.000	abnormal gas homeostasis
2138	491	10	15.17	0.9415	1.000	abnormal liver/biliary system morphology
8271	127	2	4.39	0.9441	1.000	abnormal bone ossification

2166	344	6	10	0.9447	1.000	altered tumor susceptibility
2006	344	6	10	0.9447	1.000	tumorigenesis
1392	684	24	31.18	0.9449	1.000	abnormal locomotor activity
1186	185	3	5.81	0.9453	1.000	pigmentation phenotype
1513	85	3	5.81	0.9493	1.000	limb grasping
2161	788	20	27.27	0.9523	1.000	abnormal fertility/fecundity
8946	170	4	7.53	0.9541	1.000	abnormal neuron number
2019	302	5	8.97	0.9563	1.000	abnormal tumor incidence
745	147	4	7.45	0.9567	1.000	tremors
5555	143	2	4.8	0.9575	1.000	abnormal kidney excretion
2622	116	3	6.12	0.9579	1.000	abnormal cochlear hair cell morphology
1921	353	9	14.39	0.9591	1.000	reduced fertility
160	100	2	4.61	0.9603	1.000	kyphosis
2066	970	34	43.38	0.9609	1.000	abnormal motor capabilities/coordination/movement
2752	509	15	21.69	0.9609	1.000	abnormal somatic nervous system morphology
5381	784	19	26.68	0.9635	1.000	digestive/alimentary phenotype
150	158	2	4.83	0.9649	1.000	abnormal rib morphology
5552	132	2	4.91	0.9649	1.000	abnormal orbital bone morphology
8948	142	3	6.46	0.9657	1.000	decreased neuron number
2882	768	24	32.68	0.9683	1.000	abnormal neuron morphology
1525	143	4	7.81	0.9689	1.000	impaired balance
3491	737	24	32.65	0.9691	1.000	abnormal voluntary movement
1953	108	2	4.9	0.9691	1.000	respiratory failure
4399	91	2	5.06	0.9705	1.000	abnormal cochlear outer hair cell morphology
1393	152	5	9.36	0.9741	1.000	ataxia
955	175	3	6.86	0.9761	1.000	abnormal spinal cord morphology
5393	631	13	20.45	0.9771	1.000	skin/coat/nails phenotype
3312	327	11	18.46	0.9867	1.000	abnormal locomotor coordination
230	137	2	5.81	0.9889	1.000	abnormal blood pressure
1922	145	2	6.3	0.9919	1.000	reduced male fertility
1406	181	5	11.31	0.9923	1.000	abnormal gait
959	378	8	15.42	0.9925	1.000	abnormal somatic sensory system morphology
477	220	2	7.27	0.9963	1.000	abnormal intestine morphology
965	299	6	13.77	0.9967	1.000	abnormal sensory neuron morphology
759	196	2	7.69	0.9979	1.000	abnormal skeletal muscle morphology
462	572	8	19.23	0.9995	1.000	abnormal digestive system morphology
3224	186	2	9.51	0.9997	1.000	neuron degeneration
2229	224	2	10.59	0.9999	1.000	neurodegeneration
