# Supplementary material for: Genetic Evidence Implicates the Immune System and Cholesterol Metabolism in the Aetiology of Alzheimer's Disease
Source: PLoS One. 2010 Nov 15;5(11):e13950. doi: 10.1371/journal.pone.0013950 (PMC2981526; doi:10.1371/journal.pone.0013950)
Supplement: Table S7 — All genes in the immune-related categories in Table 6. “Best p (corrected)” is the significance of the best single-SNP p-value corrected for testing multiple SNPs in a gene (allowing for LD between SNPs). “Set based p” refers to a test of whether the average single-SNP chi-squared (allelic) association statistic is significantly high (again allowing for LD between SNPs). (0.04 MB PDF) [file pone.0013950.s007.pdf]

Table S7 All genes in the immune-related categories in Table 6

"Best p (corrected)" is the significance of the best single-SNP p-value corrected for testing multiple SNPs in a gene (allowing for LD between SNPs). "Set based p" refers to a test of whether the average single-SNP chi-squared (allelic) association statistic is significantly high (again allowing for LD between SNPs).

Gene ID number	Gene Symbol	No. of SNPs (Harold[1])	Most Sig Assoc p-value (Harold[1])	No. of SNPs (Lambert[2])	Most Sig Assoc p-value (Lambert[2])	Corrected best p (Harold[1])	Set-based p (Harold[1])	Simes-corrected p (Lambert)
602	BCL3	6	<1.00E-10	6	1.90E-09	<0.0001	<0.0001	<0.0001
1191	CLU	15	1.40E-09	14	5.19E-08	<0.0001	0.0001	<0.0001
1378	CR1	29	8.32E-06	29	1.03E-06	0.0002	0.0003	<0.0001
3556	IL1RAP	50	1.26E-05	49	9.41E-03	0.0006	0.3207	0.4613
2206	MS4A2	11	5.74E-05	10	4.52E-02	0.0006	0.0010	0.2109
3123	HLA-DRB1	18	1.55E-04	12	1.29E-04	0.0028	0.0010	0.0011
116449	MIST	62	2.72E-04	55	8.72E-02	0.0167	0.4525	0.9470
151888	BTLA	12	3.67E-04	11	6.85E-02	0.0044	0.0010	0.5064
3122	HLA-DRA	50	3.92E-04	45	4.63E-04	0.0190	0.0420	0.0208
8807	IL18RAP	15	3.94E-04	15	1.61E-02	0.0037	0.0004	0.1480
6318	SERPINB4	5	5.05E-04	5	6.44E-01	0.0025	0.0939	0.9348
1380	CR2	21	5.22E-04	21	2.10E-04	0.0083	0.0114	0.0044
8809	IL18R1	16	5.42E-04	16	1.74E-02	0.0048	0.0005	0.2787
9934	P2RY14	14	5.47E-04	13	1.16E-01	0.0076	0.0040	0.7665
3112	HLA-DOB	75	5.64E-04	64	2.03E-03	0.0210	0.1399	0.1299
6891	TAP2	97	5.64E-04	83	6.50E-03	0.0380	0.0709	0.2606
3426	CFI	18	5.85E-04	18	1.01E-01	0.0105	0.0370	0.8677
9156	EXO1	16	6.52E-04	19	9.65E-02	0.0104	0.0400	0.7243
11314	CD300A	14	7.23E-04	13	2.20E-01	0.0080	0.0040	0.9508
6387	CXCL12	18	7.41E-04	17	7.51E-03	0.0090	0.0030	0.1120
735	C9	28	7.53E-04	27	3.38E-02	0.0170	0.0929	0.4701
2590	GALNT2	83	7.60E-04	79	1.02E-02	0.0440	0.3516	0.7473
337	APOA4	4	7.62E-04	3	3.00E-01	0.0030	0.0140	0.4672
23308	ICOSLG	21	8.36E-04	19	2.21E-01	0.0174	0.0050	0.8863
9173	IL1RL1	22	9.02E-04	22	1.74E-02	0.0133	0.0070	0.2551
3117	HLA-DQA1	24	9.32E-04	14	6.62E-03	0.0150	0.0669	0.0927
716	C1S	6	9.73E-04	6	4.55E-03	0.0051	0.0074	0.0195
23705	CADM1	71	1.01E-03	69	7.10E-02	0.0691	0.4875	0.7522
10060	ABCC9	75	1.05E-03	72	1.82E-02	0.0330	0.1249	0.3879
976	CD97	13	1.06E-03	11	6.25E-02	0.0137	0.1568	0.6873
708	C1QBP	8	1.29E-03	8	5.28E-03	0.0060	0.0330	0.0422
353514	LILRA5	12	1.37E-03	11	8.09E-03	0.0110	0.0090	0.0890
11026	LILRA3	10	1.37E-03	9	2.53E-02	0.0110	0.0080	0.2279
6868	ADAM17	11	1.44E-03	11	3.73E-02	0.0100	0.1009	0.4099
4851	NOTCH1	16	1.48E-03	14	1.41E-01	0.0233	0.0170	0.9652
8942	KYNU	41	1.71E-03	40	6.91E-02	0.0679	0.5445	0.6268
356	FASLG	10	1.90E-03	10	2.83E-01	0.0170	0.0060	0.9338
27159	CHIA	33	1.92E-03	32	8.98E-03	0.0459	0.0380	0.2307
1604	CD55	4	1.97E-03	4	4.28E-02	0.0079	0.0729	0.1285
10225	CD96	18	2.01E-03	17	5.67E-02	0.0356	0.2827	0.6884
5336	PLCG2	103	2.03E-03	100	3.68E-03	0.1718	0.0390	0.1937
9567	GTPBP1	6	2.03E-03	6	4.72E-01	0.0080	0.0190	0.9761
3981	LIG4	11	2.10E-03	11	2.03E-02	0.0228	0.0250	0.2055
55824	PAG1	57	2.41E-03	56	7.20E-04	0.1286	0.5295	0.0403
1394	CRHR1	18	2.46E-03	17	1.87E-02	0.0430	0.0659	0.1917
730	C7	40	2.47E-03	42	2.68E-03	0.0759	0.6993	0.1126
6368	CCL23	10	2.64E-03	9	4.64E-03	0.0170	0.0130	0.0417
929	CD14	14	2.79E-03	14	6.11E-02	0.0220	0.0260	0.3346
3550	IK	13	2.79E-03	13	9.28E-02	0.0250	0.0140	0.6215
29760	BLNK	25	2.88E-03	27	1.35E-02	0.0549	0.1289	0.3638
5818	PVRL1	23	3.15E-03	24	1.64E-01	0.0440	0.1838	0.9887
3119	HLA-DQB1	19	3.20E-03	12	6.11E-03	0.0400	0.0190	0.0733

4091	SMAD6	34	3.23E-03	37	2.44E-01	0.1042	0.1768	0.8997
715	C1R	13	3.56E-03	13	4.55E-03	0.0453	0.0949	0.0591
282618	IL29	7	4.10E-03	6	1.24E-01	0.0240	0.0430	0.5366
340061	TMEM173	3	4.24E-03	3	2.91E-01	0.0120	0.0110	0.8744
596	BCL2	74	4.48E-03	72	3.09E-03	0.2058	0.2208	0.2222
868	CBLB	48	4.54E-03	46	2.78E-02	0.0929	0.0769	0.9005
55904	MLL5	13	4.69E-03	12	2.64E-02	0.0593	0.0729	0.1176
5788	PTPRC	23	4.99E-03	22	1.03E-02	0.0709	0.0190	0.2264
5971	RELB	6	5.06E-03	6	1.06E-02	0.0310	0.1069	0.0635
3111	HLA-DOA	68	5.18E-03	67	9.73E-04	0.1858	0.5375	0.0362
6957	TRB@	141	5.28E-03	134	2.74E-02	0.5258	0.5964	0.9445
8477	GPR65	11	5.53E-03	11	9.08E-02	0.0350	0.0270	0.5357
27178	IL1F7	13	5.91E-03	12	1.40E-01	0.0742	0.4366	0.6671
157869	RPESP	14	5.97E-03	13	2.16E-01	0.0804	0.0869	0.9920
4155	MBP	90	6.06E-03	88	6.37E-03	0.3137	0.1199	0.2534
1667	DEFA1	10	6.14E-03	9	2.12E-01	0.0450	0.0170	0.9314
10859	LILRB1	15	6.25E-03	13	3.98E-02	0.0819	0.1239	0.1864
1147	CHUK	8	6.46E-03	7	9.00E-05	0.0499	0.0979	0.0006
10563	CXCL13	18	6.68E-03	17	4.43E-02	0.1136	0.0899	0.3731
51279	C1RL	15	6.93E-03	15	4.42E-02	0.0991	0.1489	0.2037
3635	INPP5D	57	6.93E-03	51	2.63E-02	0.2488	0.0609	0.4253
10344	CCL26	8	7.10E-03	8	2.64E-01	0.0479	0.2757	0.5946
6369	CCL24	6	7.10E-03	6	2.47E-01	0.0390	0.0360	0.7441
23495	TNFRSF13B	25	7.15E-03	19	2.10E-03	0.1249	0.2867	0.0399
4065	LY75	28	7.16E-03	28	2.07E-02	0.1329	0.0849	0.2488
3394	IRF8	39	7.22E-03	38	8.94E-04	0.2461	0.1888	0.0271
729	C6	26	7.74E-03	26	2.19E-01	0.1518	0.3546	0.9445
56259	CTNNBL1	21	7.79E-03	21	1.84E-01	0.0759	0.0669	0.9296
100128009	LOC100128(	36	7.81E-03	33	2.82E-02	0.5295	0.2947	0.3230
440786	LOC440786	22	7.81E-03	20	2.94E-02	0.1585	0.1988	0.2936
84659	RNASE7	18	7.82E-03	18	4.30E-02	0.1318	0.7702	0.5291
3108	HLA-DMA	42	7.97E-03	39	1.99E-02	0.2008	0.4006	0.7752
5698	PSMB9	62	8.05E-03	55	6.50E-03	0.2967	0.0829	0.1539
5696	PSMB8	81	8.05E-03	71	6.50E-03	0.3716	0.0559	0.2129
6890	TAP1	83	8.05E-03	71	6.50E-03	0.3606	0.0520	0.1987
10695	CNPY3	11	8.05E-03	11	1.78E-02	0.0659	0.0450	0.1732
56477	CCL28	9	8.17E-03	9	6.60E-02	0.0679	0.1698	0.4306
56253	CRTAM	21	8.22E-03	21	1.41E-01	0.1099	0.2667	0.7140
170482	CLEC4C	13	8.36E-03	12	5.72E-02	0.0809	0.1499	0.2608
311	ANXA11	24	8.38E-03	23	7.45E-02	0.1239	0.0639	1.0000
8876	VNN1	26	8.42E-03	25	9.31E-03	0.1973	0.2757	0.2328
3566	IL4R	28	8.44E-03	27	9.65E-02	0.1309	0.1049	0.8491
8600	TNFSF11	17	8.62E-03	18	5.41E-02	0.0919	0.1389	0.5119
10346	TRIM22	30	8.66E-03	28	3.55E-02	0.2297	0.1548	0.3222
10321	CRISP3	12	8.92E-03	11	6.50E-02	0.0699	0.0220	0.3419
9547	CXCL14	9	9.07E-03	9	2.89E-01	0.0788	0.0859	0.6731
5763	PTMS	12	9.18E-03	12	1.06E-01	0.1047	0.4066	0.9556
3574	IL7	19	9.27E-03	18	3.91E-02	0.0999	0.1329	0.7035
27179	IL1F6	7	9.45E-03	7	3.05E-02	0.0643	0.4196	0.1450
27177	IL1F8	14	9.45E-03	14	6.22E-02	0.1245	0.6044	0.4343
326	AIRE	18	9.52E-03	15	4.27E-03	0.1359	0.2118	0.0401
8878	SQSTM1	11	9.67E-03	12	2.17E-01	0.0859	0.3746	0.8212
2533	FYB	35	9.69E-03	35	2.64E-02	0.1788	0.2937	0.5339
22904	SBNO2	17	1.01E-02	13	7.51E-02	0.1289	0.0929	0.5525
79679	VTCN1	24	1.02E-02	22	6.38E-02	0.1588	0.5804	0.8000
54900	LAX1	7	1.02E-02	7	2.06E-02	0.0499	0.1139	0.1047
926	CD8B	8	1.04E-02	6	4.56E-01	0.0599	0.0320	0.7079
353376	TICAM2	15	1.06E-02	14	1.01E-02	0.0889	0.0759	0.1414
7409	VAV1	35	1.14E-02	34	5.67E-02	0.3308	0.2058	0.8857
1672	DEFB1	34	1.19E-02	32	1.88E-01	0.2278	0.0230	0.9570
93978	CLEC6A	5	1.21E-02	4	1.61E-01	0.0588	0.7672	0.2823
4437	MSH3	51	1.22E-02	49	1.34E-02	0.2567	0.3666	0.4369

1521	CTSW	4	1.23E-02	3	2.84E-01	0.0484	0.2867	0.4517
5079	PAX5	100	1.23E-02	101	2.89E-02	0.5674	0.5155	0.8226
7157	TP53	8	1.27E-02	8	8.99E-02	0.0759	0.3746	0.4565
7099	TLR4	14	1.28E-02	13	1.62E-02	0.1129	0.4166	0.2109
10241	CALCOCO2	15	1.30E-02	15	6.26E-02	0.0949	0.0470	0.2536
149233	IL23R	32	1.30E-02	32	3.94E-02	0.1968	0.0559	0.3865
3605	IL17A	21	1.32E-02	21	5.55E-04	0.1578	0.1369	0.0116
1520	CTSS	4	1.33E-02	4	1.04E-01	0.0360	0.0649	0.4151
710	SERPING1	13	1.34E-02	12	5.92E-02	0.1069	0.0490	0.6632
8174	MADCAM1	7	1.36E-02	7	2.94E-02	0.0849	0.1169	0.1455
28454	IGHV2-70	8	1.36E-02	9	3.25E-02	0.0699	0.0599	0.2929
220972	MARCH_8	24	1.39E-02	23	4.29E-02	0.2078	0.0929	0.4579
58191	CXCL16	17	1.40E-02	18	1.30E-01	0.2127	0.2517	0.9789
920	CD4	29	1.41E-02	28	9.53E-02	0.2547	0.3986	0.8686
23547	LILRA4	26	1.44E-02	24	2.36E-03	0.2208	0.2657	0.0565
3493	IGHA1	1	1.44E-02	0	N/A	0.0140	0.0140	N/A
3500	IGHG1	1	1.44E-02	0	N/A	0.0140	0.0140	N/A
1755	DMBT1	8	1.46E-02	7	1.59E-01	0.0959	0.1538	0.6570
608	TNFRSF17	10	1.47E-02	10	1.47E-01	0.1374	0.2178	0.6789
10410	IFITM3	7	1.48E-02	6	8.65E-02	0.0990	0.4426	0.2755
57379	AICDA	8	1.50E-02	7	2.11E-01	0.1139	0.0599	0.9563
3105	HLA-A	35	1.58E-02	27	5.96E-02	0.3506	0.7253	0.6613
3075	CFH	17	1.59E-02	13	2.31E-03	0.2068	0.2048	0.0300
942	CD86	24	1.59E-02	23	5.43E-02	0.2587	0.3077	0.5917
64127	NOD2	12	1.61E-02	12	1.09E-02	0.1768	0.2997	0.0834
3569	IL6	17	1.61E-02	17	3.57E-02	0.1768	0.2048	0.3321
1511	CTSG	7	1.64E-02	7	1.16E-01	0.1094	0.0999	0.8100
8698	EDG6	8	1.64E-02	8	5.67E-02	0.1089	0.1009	0.3386
79465	ULBP3	19	1.67E-02	17	2.84E-02	0.1958	0.1019	0.1276
3134	HLA-F	34	1.68E-02	31	1.19E-02	0.4385	0.7203	0.1548
974	CD79B	8	1.70E-02	8	2.42E-01	0.1089	0.3267	0.7322
1075	CTSC	24	1.70E-02	24	1.64E-01	0.2418	0.0829	0.9465
3606	IL18	10	1.71E-02	10	4.30E-03	0.1129	0.0899	0.0298
23601	CLEC5A	7	1.72E-02	7	5.92E-01	0.1146	0.1898	0.9805
5450	POU2AF1	14	1.73E-02	17	1.56E-01	0.1868	0.2448	1.0000
4277	MICB	76	1.74E-02	59	6.88E-03	0.6693	0.3187	0.2075
721	C4B	16	1.74E-02	16	2.73E-02	0.2455	0.7113	0.2953
717	C2	35	1.74E-02	32	1.01E-02	0.2455	0.8861	0.3227
629	CFB	32	1.74E-02	31	1.01E-02	0.4307	0.8282	0.3126
1880	EBI2	10	1.77E-02	9	7.46E-02	0.1633	0.8162	0.4135
7518	XRCC4	26	1.77E-02	29	4.11E-02	0.2647	0.1119	0.5366
5806	PTX3	13	1.77E-02	13	1.90E-01	0.2073	0.3337	0.7754
366	AQP9	32	1.82E-02	32	8.34E-02	0.3546	0.1249	0.7510
7097	TLR2	14	1.83E-02	14	1.15E-01	0.2148	0.1958	0.9777
7049	TGFBR3	78	1.85E-02	76	9.27E-03	0.7666	0.7882	0.3373
11006	LILRB4	22	1.87E-02	21	2.82E-04	0.3397	0.2488	0.0041
6364	CCL20	11	1.88E-02	11	9.36E-02	0.1883	0.1419	0.6239
2920	CXCL2	3	1.89E-02	3	4.24E-01	0.0557	0.6404	0.4985
5648	MASP1	50	1.90E-02	50	5.05E-02	0.4386	0.4006	0.9385
940	CD28	10	1.93E-02	10	4.27E-01	0.1079	0.0540	0.8937
6348	CCL3	14	1.94E-02	13	9.30E-02	0.1439	0.0599	0.4860
6351	CCL4	10	1.94E-02	11	9.30E-02	0.1429	0.1319	0.5874
3683	ITGAL	14	1.94E-02	14	9.13E-03	0.2278	0.4046	0.1279
57115	PGLYRP4	15	1.97E-02	14	1.53E-01	0.1688	0.1768	0.8157
3480	IGF1R	106	1.97E-02	105	8.82E-03	0.8252	0.1718	0.4619
6932	TCF7	13	1.98E-02	12	9.73E-02	0.2284	0.2747	0.4900
148022	TICAM1	7	2.02E-02	7	5.98E-02	0.1329	0.3227	0.4183
9450	LY86	50	2.02E-02	49	3.07E-02	0.4845	0.4755	0.5672
3586	IL10	18	2.04E-02	17	5.43E-02	0.2567	0.2627	0.4084
4542	MYO1F	15	2.06E-02	15	1.33E-02	0.2678	0.2218	0.1706
3118	HLA-DQA2	45	2.06E-02	39	9.01E-02	0.2977	0.1439	0.9681
3140	MR1	13	2.09E-02	15	6.32E-02	0.2397	0.4795	0.9096

665	BNIP3L	17	2.11E-02	18	8.11E-02	0.3036	0.9950	0.5937
2113	ETS1	39	2.14E-02	39	6.39E-03	0.3906	0.5604	0.2493
29949	IL19	24	2.15E-02	24	5.01E-02	0.4063	0.3896	0.4547
3127	HLA-DRB5	6	2.19E-02	1	3.76E-01	0.0919	0.1159	0.3760
8518	IKBKAP	34	2.20E-02	34	9.76E-03	0.3367	0.5854	0.2789
6672	SP100	50	2.21E-02	48	5.23E-03	0.4835	0.0879	0.1427
7293	TNFRSF4	2	2.22E-02	4	2.15E-01	0.0400	0.0170	0.7624
10462	CLEC10A	13	2.23E-02	13	4.39E-01	0.2168	0.1648	0.9887
720	C4A	8	2.30E-02	7	2.52E-03	0.1399	0.2597	0.0177
6360	CCL16	14	2.31E-02	13	1.57E-03	0.2138	0.0699	0.0204
6358	CCL14	12	2.31E-02	11	1.57E-03	0.1828	0.0540	0.0173
3274	HRH2	10	2.34E-02	9	1.77E-03	0.1518	0.2777	0.0159
1238	CCBP2	16	2.37E-02	15	1.23E-02	0.2118	0.4675	0.1847
4261	CIITA	10	2.39E-02	9	7.94E-03	0.2150	0.8811	0.0715
1233	CCR4	6	2.41E-02	6	7.97E-02	0.1079	0.0939	0.3654
6359	CCL15	9	2.41E-02	8	1.57E-03	0.1369	0.0669	0.0126
8808	IL1RL2	33	2.41E-02	34	1.14E-01	0.5535	0.4515	0.7122
4939	OAS2	20	2.42E-02	19	1.88E-02	0.3868	0.5664	0.2118
6846	XCL2	11	2.43E-02	10	2.63E-02	0.2369	0.9880	0.1151
3578	IL9	20	2.45E-02	21	9.15E-03	0.3447	0.3816	0.1922
3106	HLA-B	49	2.49E-02	35	1.26E-02	0.5135	0.5025	0.3970
567	B2M	6	2.49E-02	6	3.65E-02	0.1189	0.1359	0.2190
114771	PGLYRP3	8	2.50E-02	7	5.04E-01	0.1299	0.1289	0.9606
3600	IL15	14	2.50E-02	12	1.62E-01	0.2827	0.1788	0.6036
7852	CXCR4	8	2.51E-02	8	1.17E-01	0.1648	0.1469	0.7892
8631	SKAP1	32	2.52E-02	33	3.41E-01	0.3337	0.0719	0.9774
7942	TFEB	22	2.53E-02	23	7.27E-02	0.3417	0.4116	0.8683
5008	OSM	14	2.55E-02	13	2.30E-03	0.3035	0.9251	0.0168
8530	CST7	6	2.57E-02	6	2.23E-01	0.1447	0.8292	0.9652
8915	BCL10	16	2.58E-02	16	8.83E-02	0.2298	0.1409	0.5790
4860	NP	13	2.59E-02	13	1.14E-01	0.2597	0.5075	0.8349
7098	TLR3	15	2.60E-02	16	1.39E-01	0.2587	0.3137	0.8794
563	AZGP1	6	2.62E-02	6	5.94E-02	0.1473	0.2398	0.1803
909	CD1A	3	2.65E-02	4	5.50E-01	0.0775	0.5105	0.7124
83417	FCRL4	11	2.68E-02	11	2.24E-01	0.1489	0.1169	0.9388
718	C3	20	2.71E-02	21	6.13E-02	0.4233	0.9670	0.8712
3848	KRT1	17	2.76E-02	19	1.79E-01	0.3696	0.7712	0.9920
5996	RGS1	20	2.79E-02	19	1.66E-01	0.4316	0.3976	0.9535
56241	SUSD2	4	2.80E-02	3	2.97E-01	0.1074	0.2657	0.5185
386653	IL31	9	2.80E-02	9	2.82E-01	0.1748	0.2827	0.8273
11027	LILRA2	8	2.81E-02	9	8.13E-05	0.2038	0.4436	0.0007
3553	IL1B	11	2.86E-02	10	1.42E-01	0.2368	0.3896	0.9601
10417	SPON2	7	2.88E-02	5	9.15E-02	0.1850	0.4296	0.2630
2634	GBP2	13	2.90E-02	11	5.99E-01	0.3180	0.5055	0.9822
4973	OLR1	17	2.93E-02	16	1.70E-01	0.2637	0.2687	0.8573
84876	ORAI1	7	2.93E-02	6	1.93E-01	0.1059	0.2757	0.6339
81035	COLEC12	109	2.94E-02	105	1.75E-02	0.9041	0.9321	0.5783
2669	GEM	17	2.96E-02	17	9.31E-02	0.4004	0.3157	0.8828
9755	TBKBP1	6	3.01E-02	6	2.17E-02	0.1349	0.0410	0.0790
3567	IL5	7	3.10E-02	7	3.13E-01	0.1419	0.1658	0.7352
8764	TNFRSF14	7	3.11E-02	6	2.96E-02	0.1329	0.1379	0.1081
8542	APOL1	19	3.12E-02	19	1.17E-01	0.3576	0.6583	0.4540
27343	POLL	5	3.13E-02	5	4.25E-02	0.1469	0.2917	0.2126
6357	CCL13	9	3.14E-02	8	9.72E-02	0.1918	0.3297	0.7192
6346	CCL1	11	3.14E-02	10	9.72E-02	0.2338	0.4256	0.7684
3559	IL2RA	55	3.15E-02	57	2.79E-02	0.6723	0.3457	0.5878
7441	VPREB1	12	3.16E-02	11	1.56E-01	0.2058	0.3976	0.7868
10814	CPLX2	48	3.18E-02	45	4.39E-04	0.5524	0.6134	0.0109
2213	FCGR2B	9	3.18E-02	9	1.89E-01	0.2524	0.3616	0.7577
1401	CRP	6	3.22E-02	6	2.14E-01	0.1449	0.1049	0.6561
55122	C6orf166	18	3.23E-02	20	3.58E-03	0.4466	0.7353	0.0610
64421	DCLRE1C	12	3.24E-02	11	2.79E-01	0.2118	0.1049	0.6509

3109	HLA-DMB	42	3.25E-02	39	1.99E-02	0.7502	0.7373	0.7752
924	CD7	4	3.32E-02	5	4.54E-02	0.1039	0.1668	0.2270
54	ACP5	8	3.42E-02	8	9.15E-02	0.2431	0.3457	0.4344
939	CD27	17	3.53E-02	18	1.04E-01	0.3866	0.7173	0.9822
1584	CYP11B1	9	3.55E-02	8	1.31E-01	0.2774	0.6673	0.2675
84955	NUDCD1	7	3.55E-02	5	2.51E-02	0.2236	0.6813	0.1257
91543	RSAD2	10	3.57E-02	9	1.30E-01	0.3046	0.1489	0.9522
3636	INPPL1	8	3.59E-02	6	1.73E-02	0.1908	0.2038	0.1036
6355	CCL8	15	3.61E-02	16	1.27E-01	0.2727	0.1888	0.7911
6356	CCL11	13	3.61E-02	14	1.18E-01	0.2498	0.1109	0.9057
10333	TLR6	4	3.62E-02	6	5.16E-01	0.1039	0.0599	0.7712
6668	SP2	8	3.64E-02	8	4.44E-01	0.1888	0.2048	0.7868
3001	GZMA	13	3.67E-02	11	3.27E-01	0.3853	0.5854	0.8810
56300	IL1F9	9	3.68E-02	8	3.05E-02	0.2863	0.5345	0.1736
1535	CYBA	9	3.70E-02	10	4.39E-02	0.2737	0.2038	0.2728
2204	FCAR	8	3.71E-02	9	6.25E-01	0.1958	0.4376	0.9528
657	BMPR1A	26	3.72E-02	28	5.01E-02	0.3846	0.3896	0.4195
127544	RNF19B	11	3.74E-02	10	1.84E-01	0.3422	0.9560	0.4911
50852	TRAT1	20	3.75E-02	20	2.17E-01	0.4346	0.6793	0.9439
26253	CLEC4E	15	3.76E-02	15	3.77E-02	0.4370	0.2947	0.5660
10332	CLEC4M	21	3.77E-02	19	1.76E-03	0.5536	0.7752	0.0334
7040	TGFB1	8	3.78E-02	8	1.10E-01	0.2547	0.6004	0.4499
8741	TNFSF13	11	3.82E-02	11	2.58E-01	0.2198	0.0889	0.9617
8742	TNFSF12	10	3.82E-02	10	5.35E-02	0.2048	0.1059	0.5349
1493	CTLA4	7	3.84E-02	7	3.11E-01	0.1908	0.3247	0.7491
5393	EXOSC9	8	3.88E-02	7	4.87E-01	0.2328	0.3536	0.8734
3588	IL10RB	18	3.89E-02	18	5.26E-02	0.4426	0.6763	0.6722
6480	ST6GAL1	66	3.91E-02	67	9.56E-03	0.8052	0.8262	0.3834
51752	ERAP1	38	3.95E-02	38	2.90E-02	0.7840	0.9820	0.8533
80380	PDCD1LG2	26	3.95E-02	25	2.02E-02	0.5005	0.8691	0.1755
353091	RAET1G	8	3.98E-02	7	1.98E-02	0.2773	0.1149	0.1389
80328	ULBP2	7	3.98E-02	8	1.98E-02	0.2468	0.4206	0.1588
28577	TRBV12-3	12	4.00E-02	12	4.82E-02	0.3870	0.5175	0.5779
3681	ITGAD	9	4.00E-02	9	1.20E-01	0.2028	0.1728	0.9157
2643	GCH1	10	4.00E-02	10	1.47E-02	0.2637	0.1439	0.1471
8993	PGLYRP1	12	4.03E-02	10	1.65E-01	0.3337	0.5435	0.6088
3576	IL8	4	4.09E-02	4	4.05E-01	0.1539	0.2537	0.7883
60489	APOBEC3G	7	4.16E-02	6	6.59E-02	0.2572	0.6643	0.3955
683	BST1	31	4.17E-02	31	7.15E-02	0.7328	0.5724	0.9106
6938	TCF12	52	4.22E-02	45	2.84E-03	0.5245	0.2817	0.1278
4843	NOS2A	21	4.23E-02	19	1.17E-02	0.5967	0.4765	0.2218
54878	DPP8	10	4.24E-02	7	1.74E-01	0.2488	0.2018	0.7287
6814	STXBP3	19	4.25E-02	19	2.20E-03	0.3596	0.1698	0.0250
6370	CCL25	19	4.28E-02	17	1.50E-02	0.4006	0.6833	0.2558
1241	LTB4R	12	4.30E-02	13	6.55E-03	0.3596	0.4995	0.0852
5468	PPARG	35	4.40E-02	34	6.12E-03	0.5385	0.8851	0.2080
6935	ZEB1	13	4.45E-02	16	1.02E-01	0.3177	0.1089	0.7613
5553	PRG2	7	4.49E-02	8	5.77E-03	0.1978	0.1558	0.0215
10394	PRG3	8	4.49E-02	9	7.47E-03	0.2198	0.1678	0.0363
6354	CCL7	12	4.50E-02	12	1.18E-01	0.3017	0.1928	0.8225
4282	MIF	18	4.54E-02	18	3.17E-01	0.4056	0.8302	0.8947
6362	CCL18	12	4.56E-02	11	8.99E-02	0.2747	0.0619	0.3564
944	TNFSF8	20	4.57E-02	18	2.30E-01	0.6072	0.7303	0.7482
3903	LAIR1	24	4.59E-02	21	2.36E-03	0.5744	0.6284	0.0495
50856	CLEC4A	14	4.64E-02	14	8.57E-03	0.3027	0.3686	0.1200
10512	SEMA3C	37	4.65E-02	38	1.48E-02	0.6394	0.3896	0.2850
10164	CHST4	13	4.70E-02	12	3.02E-04	0.3976	0.5475	0.0036
3603	IL16	29	4.71E-02	27	8.47E-02	0.5425	0.3566	0.8219
159296	NKX2-3	5	4.71E-02	5	1.72E-01	0.2144	0.5654	0.3533
6363	CCL19	7	4.80E-02	7	2.28E-01	0.2567	0.2108	0.9761
6366	CCL21	6	4.80E-02	6	3.26E-02	0.2188	0.2218	0.1956
6965	TRG@	57	4.82E-02	57	4.34E-02	0.8332	0.9131	1.0000

2207	FCER1G	16	4.88E-02	16	1.24E-02	0.4086	0.3906	0.1981
6347	CCL2	9	4.96E-02	9	1.97E-01	0.2777	0.2478	0.8225
27040	LAT	8	5.02E-02	11	1.43E-01	NS	NS	0.7661
4179	CD46	10	5.04E-02	9	3.11E-01	NS	NS	0.7575
3077	HFE	16	5.06E-02	17	3.19E-01	NS	NS	0.9552
28639	TRBC1	8	5.06E-02	8	3.09E-01	NS	NS	0.7662
3557	IL1RN	11	5.06E-02	10	3.11E-01	NS	NS	0.7483
1675	CFD	11	5.23E-02	9	3.99E-02	NS	NS	0.3592
387357	C6orf190	49	5.26E-02	45	2.67E-02	NS	NS	0.6446
2537	IFI6	5	5.27E-02	5	3.54E-01	NS	NS	0.8818
135250	RAET1E	7	5.34E-02	7	3.03E-01	NS	NS	0.7088
27240	SIT1	11	5.34E-02	11	6.25E-03	NS	NS	0.0687
731	C8A	21	5.35E-02	21	2.59E-01	NS	NS	0.8884
201294	UNC13D	8	5.39E-02	7	2.08E-01	NS	NS	0.5564
57506	VISA	16	5.43E-02	16	7.29E-02	NS	NS	0.5537
6375	XCL1	13	5.47E-02	9	3.17E-01	NS	NS	0.9805
4057	LTF	10	5.56E-02	10	2.25E-01	NS	NS	0.5083
10288	LILRB2	9	5.60E-02	7	2.75E-01	NS	NS	0.6971
2219	FCN1	17	5.61E-02	16	1.95E-02	NS	NS	0.1573
7100	TLR5	5	5.65E-02	5	3.29E-01	NS	NS	0.6300
1240	CMKLR1	21	5.66E-02	23	2.10E-03	NS	NS	0.0298
722	C4BPA	13	5.69E-02	13	8.46E-02	NS	NS	0.5627
11151	CORO1A	1	5.72E-02	1	7.35E-01	NS	NS	0.7347
6441	SFTPD	18	5.72E-02	17	1.43E-01	NS	NS	0.5823
4049	LTA	31	5.75E-02	32	2.82E-03	NS	NS	0.0901
7124	TNF	30	5.75E-02	30	1.44E-02	NS	NS	0.3325
4783	NFIL3	13	5.83E-02	13	3.21E-02	NS	NS	0.4169
2956	MSH6	14	5.83E-02	14	6.99E-02	NS	NS	0.3649
10148	EBI3	8	5.84E-02	7	3.39E-01	NS	NS	0.9920
8909	P11	9	5.87E-02	9	1.09E-01	NS	NS	0.8428
23075	SWAP70	17	6.02E-02	17	7.79E-02	NS	NS	0.5102
3113	HLA-DPA1	45	6.03E-02	41	6.57E-04	NS	NS	0.0264
8995	TNFSF18	3	6.07E-02	3	2.19E-01	NS	NS	0.5092
7837	PXDN	41	6.13E-02	40	4.41E-03	NS	NS	0.1764
2921	CXCL3	3	6.16E-02	3	4.42E-01	NS	NS	0.8423
5167	ENPP1	30	6.21E-02	30	1.37E-02	NS	NS	0.4105
2220	FCN2	20	6.26E-02	19	1.95E-02	NS	NS	0.3698
3929	LBP	17	6.36E-02	19	2.21E-02	NS	NS	0.4198
10261	IGSF6	6	6.53E-02	5	4.36E-02	NS	NS	0.1034
1235	CCR6	38	6.53E-02	36	6.94E-02	NS	NS	0.5929
5724	PTAFR	7	6.57E-02	7	3.12E-01	NS	NS	0.6586
4683	NBN	15	6.68E-02	15	1.42E-01	NS	NS	0.7060
725	C4BPB	11	6.70E-02	11	8.46E-02	NS	NS	0.4761
154064	RAET1L	7	6.75E-02	6	1.98E-02	NS	NS	0.1186
2821	GPI	4	6.76E-02	4	1.12E-01	NS	NS	0.1757
3494	IGHA2	7	6.79E-02	4	3.16E-01	NS	NS	0.7010
3497	IGHE	5	6.79E-02	4	3.16E-01	NS	NS	0.7010
654	BMP6	54	6.90E-02	54	6.75E-02	NS	NS	0.7881
3592	IL12A	18	6.95E-02	17	6.63E-02	NS	NS	0.6174
5169	ENPP3	25	6.95E-02	27	1.15E-02	NS	NS	0.2571
25939	SAMHD1	14	7.04E-02	13	8.78E-02	NS	NS	0.8518
4688	NCF2	10	7.06E-02	10	1.39E-02	NS	NS	0.1338
733	C8G	5	7.08E-02	4	3.05E-01	NS	NS	0.6544
7292	TNFSF4	12	7.11E-02	12	5.05E-02	NS	NS	0.6061
84639	IL1F10	25	7.14E-02	24	1.21E-02	NS	NS	0.2903
7433	VIPR1	14	7.16E-02	16	1.39E-01	NS	NS	0.9620
9966	TNFSF15	11	7.18E-02	10	1.06E-01	NS	NS	0.9691
3554	IL1R1	43	7.19E-02	43	2.89E-02	NS	NS	0.4956
9235	IL32	10	7.19E-02	7	3.20E-01	NS	NS	0.7758
727	C5	32	7.25E-02	29	1.13E-02	NS	NS	0.2459
355	FAS	25	7.28E-02	24	2.69E-01	NS	NS	0.8927
2635	GBP3	14	7.40E-02	14	4.46E-01	NS	NS	0.9652

4938	OAS1	10	7.44E-02	9	5.60E-02	NS	NS	0.2880
3107	HLA-C	57	7.46E-02	44	6.39E-02	NS	NS	0.9013
6278	S100A7	9	7.57E-02	9	3.60E-01	NS	NS	0.9502
3503	IGHG4	2	7.60E-02	2	4.91E-01	NS	NS	0.5312
64581	CLEC7A	15	7.64E-02	15	1.01E-01	NS	NS	0.5499
4940	OAS3	15	7.67E-02	15	5.60E-02	NS	NS	0.3385
3543	IGLL1	15	7.74E-02	14	1.49E-01	NS	NS	0.6412
54941	RNF125	18	7.75E-02	15	2.93E-02	NS	NS	0.3877
8740	TNFSF14	11	7.80E-02	11	6.13E-02	NS	NS	0.6744
2161	F12	4	7.88E-02	4	5.85E-02	NS	NS	0.2340
10673	TNFSF13B	13	7.98E-02	14	2.20E-01	NS	NS	0.9887
6372	CXCL6	3	8.09E-02	3	1.43E-01	NS	NS	0.4303
5197	PF4V1	8	8.09E-02	8	2.86E-02	NS	NS	0.2274
2919	CXCL1	12	8.09E-02	12	2.86E-02	NS	NS	0.2274
2633	GBP1	13	8.15E-02	13	4.26E-01	NS	NS	0.9652
83953	FCAMR	10	8.20E-02	10	1.74E-01	NS	NS	0.7324
26525	IL1F5	21	8.23E-02	21	1.21E-02	NS	NS	0.2540
5196	PF4	6	8.58E-02	5	5.34E-02	NS	NS	0.2159
5473	PPBP	7	8.58E-02	6	2.72E-03	NS	NS	0.0163
3329	HSPD1	3	8.64E-02	3	3.84E-01	NS	NS	0.6804
1230	CCR1	4	8.65E-02	6	1.39E-01	NS	NS	0.6415
931	MS4A1	24	8.65E-02	23	1.22E-01	NS	NS	0.8264
338339	CLEC4D	11	8.66E-02	11	3.77E-02	NS	NS	0.4151
3937	LCP2	19	8.66E-02	20	1.54E-01	NS	NS	0.9056
1118	CHIT1	25	8.70E-02	25	6.37E-02	NS	NS	0.8251
359710	C20orf185	18	8.76E-02	18	8.15E-02	NS	NS	0.7996
100	ADA	12	8.78E-02	10	2.73E-02	NS	NS	0.2729
732	C8B	13	8.88E-02	13	1.58E-01	NS	NS	0.5897
10964	IFI44L	10	8.95E-02	10	1.03E-01	NS	NS	0.4552
9308	CD83	12	9.02E-02	11	9.43E-02	NS	NS	0.6732
10544	PROCR	6	9.02E-02	5	1.88E-01	NS	NS	0.3384
10803	CCR9	13	9.05E-02	13	1.19E-01	NS	NS	0.7823
342510	CD300E	15	9.08E-02	15	9.82E-02	NS	NS	0.8057
5319	PLA2G1B	7	9.14E-02	7	1.85E-01	NS	NS	0.4581
345611	IRGM	7	9.17E-02	8	1.37E-01	NS	NS	0.3791
10087	COL4A3BP	10	9.22E-02	9	2.20E-01	NS	NS	0.7694
259197	NCR3	27	9.34E-02	26	1.44E-02	NS	NS	0.3106
4050	LTB	31	9.34E-02	30	1.44E-02	NS	NS	0.3325
7940	LST1	28	9.34E-02	27	1.44E-02	NS	NS	0.3225
203068	TUBB	33	9.39E-02	37	3.57E-02	NS	NS	1.0000
29126	CD274	23	9.47E-02	21	6.60E-02	NS	NS	0.9453
54210	TREM1	12	9.52E-02	13	1.69E-02	NS	NS	0.1380
4689	NCF4	26	9.52E-02	25	3.71E-02	NS	NS	0.9267
3593	IL12B	13	9.58E-02	13	1.18E-01	NS	NS	0.6010
64167	ERAP2	16	9.61E-02	16	2.01E-01	NS	NS	0.9311
11251	GPR44	8	9.75E-02	8	1.40E-01	NS	NS	0.5455
6622	SNCA	27	9.75E-02	23	4.79E-02	NS	NS	0.5530
114770	PGLYRP2	7	9.78E-02	7	6.42E-02	NS	NS	0.4496
29110	TBK1	12	9.82E-02	11	3.67E-01	NS	NS	0.9242
913	CD1E	7	9.87E-02	6	3.30E-01	NS	NS	0.8234
7850	IL1R2	59	1.00E-01	56	2.03E-01	NS	NS	0.6313
10747	MASP2	7	1.01E-01	7	8.65E-02	NS	NS	0.5419
81494	CFHR5	7	1.01E-01	6	1.48E-01	NS	NS	0.5609
23545	ATP6V0A2	13	1.01E-01	12	3.94E-01	NS	NS	1.0000
10850	CCL27	7	1.02E-01	7	4.68E-01	NS	NS	0.9188
3570	IL6R	11	1.02E-01	11	3.44E-01	NS	NS	0.9805
3135	HLA-G	55	1.04E-01	53	1.74E-02	NS	NS	0.6223
2152	F3	10	1.05E-01	10	2.61E-01	NS	NS	0.9662
51297	PLUNC	7	1.05E-01	8	1.27E-01	NS	NS	0.9521
8638	OASL	16	1.05E-01	16	1.43E-01	NS	NS	0.8213
100131439	LOC1001314	16	1.05E-01	16	9.82E-02	NS	NS	0.5449
6374	CXCL5	8	1.05E-01	7	2.72E-03	NS	NS	0.0191

970	CD70	12	1.06E-01	12	1.10E-01	NS	NS	0.7738
1051	CEBPB	4	1.08E-01	4	3.36E-01	NS	NS	0.7966
81793	TLR10	14	1.08E-01	15	1.66E-01	NS	NS	1.0000
54472	TOLLIP	10	1.09E-01	10	9.73E-02	NS	NS	0.8812
4985	OPRD1	15	1.11E-01	15	4.46E-03	NS	NS	0.0669
79168	LILRA6	8	1.12E-01	11	3.18E-01	NS	NS	0.8678
7535	ZAP70	16	1.12E-01	15	9.78E-02	NS	NS	0.5872
11025	LILRB3	6	1.12E-01	11	1.22E-01	NS	NS	0.9321
114609	TIRAP	13	1.13E-01	13	3.50E-03	NS	NS	0.0455
57823	SLAMF7	6	1.13E-01	6	8.30E-03	NS	NS	0.0498
9641	IKBKE	16	1.13E-01	14	1.20E-01	NS	NS	0.7326
11024	LILRA1	8	1.14E-01	9	8.13E-05	NS	NS	0.0007
5897	RAG2	5	1.15E-01	5	1.15E-02	NS	NS	0.0575
163351	GBP6	14	1.15E-01	12	1.33E-01	NS	NS	0.6179
29851	ICOS	10	1.15E-01	12	1.57E-01	NS	NS	0.9748
54923	LIME1	7	1.16E-01	4	5.42E-01	NS	NS	0.9117
146894	CD300LG	4	1.16E-01	4	3.56E-01	NS	NS	0.6393
59307	SIGIRR	10	1.16E-01	4	6.53E-01	NS	NS	1.0000
51441	YTHDF2	7	1.17E-01	6	1.94E-02	NS	NS	0.1164
3115	HLA-DPB1	46	1.19E-01	45	9.17E-02	NS	NS	0.9528
133396	IL31RA	20	1.20E-01	21	1.57E-01	NS	NS	0.8489
51295	ECSIT	5	1.20E-01	5	1.78E-01	NS	NS	0.6308
6813	STXBP2	4	1.20E-01	3	6.04E-02	NS	NS	0.1811
4063	LY9	12	1.23E-01	12	1.30E-02	NS	NS	0.0668
6376	CX3CL1	11	1.25E-01	11	1.65E-01	NS	NS	0.7096
6367	CCL22	11	1.25E-01	12	1.65E-01	NS	NS	0.6429
3764	KCNJ8	1	1.25E-01	1	3.79E-02	NS	NS	0.0379
6398	SECTM1	4	1.25E-01	5	6.39E-01	NS	NS	0.8731
6892	TAPBP	9	1.26E-01	9	7.64E-02	NS	NS	0.6874
23643	LY96	11	1.29E-01	11	3.08E-02	NS	NS	0.2139
8482	SEMA7A	13	1.32E-01	12	2.21E-02	NS	NS	0.2500
9466	IL27RA	4	1.35E-01	4	5.82E-01	NS	NS	0.8824
8547	FCN3	6	1.36E-01	6	6.68E-01	NS	NS	0.9283
4064	CD180	10	1.37E-01	12	2.73E-01	NS	NS	0.9403
23586	DDX58	23	1.38E-01	20	1.47E-02	NS	NS	0.2943
3495	IGHD	1	1.39E-01	0	N/A	NS	NS	N/A
3507	IGHM	1	1.39E-01	0	N/A	NS	NS	N/A
1088	CEACAM8	1	1.42E-01	1	3.12E-01	NS	NS	0.3118
3627	CXCL10	8	1.44E-01	7	4.15E-03	NS	NS	0.0103
4283	CXCL9	6	1.44E-01	5	6.35E-01	NS	NS	0.9248
8744	TNFSF9	8	1.44E-01	7	1.61E-01	NS	NS	0.5918
2794	GNL1	20	1.46E-01	25	1.84E-03	NS	NS	0.0113
51371	POMP	13	1.47E-01	13	6.30E-02	NS	NS	0.6798
1594	CYP27B1	8	1.48E-01	7	6.61E-01	NS	NS	0.9283
353219	KAAG1	8	1.49E-01	8	3.21E-01	NS	NS	0.9571
200316	APOBEC3F	5	1.54E-01	4	1.57E-01	NS	NS	0.2608
54106	TLR9	7	1.54E-01	7	2.38E-01	NS	NS	0.6024
3383	ICAM1	11	1.55E-01	13	1.53E-01	NS	NS	0.8141
79671	NLRX1	10	1.55E-01	10	5.20E-02	NS	NS	0.3651
57817	HAMP	7	1.56E-01	7	6.27E-02	NS	NS	0.4389
115362	GBP5	9	1.60E-01	9	5.92E-02	NS	NS	0.3383
3804	KIR2DL3	2	1.62E-01	1	2.04E-01	NS	NS	0.2042
8743	TNFSF10	19	1.63E-01	18	1.56E-02	NS	NS	0.1494
30835	CD209	18	1.63E-01	16	1.76E-03	NS	NS	0.0282
3575	IL7R	14	1.64E-01	11	2.30E-01	NS	NS	0.8440
3078	CFHR1	2	1.64E-01	1	6.49E-01	NS	NS	0.6487
146722	CD300LF	7	1.69E-01	7	5.34E-01	NS	NS	0.9372
10990	LILRB5	7	1.69E-01	8	3.18E-01	NS	NS	0.8889
972	CD74	7	1.70E-01	7	2.06E-01	NS	NS	0.4098
124599	CD300LB	10	1.71E-01	10	7.14E-01	NS	NS	1.0000
911	CD1C	3	1.71E-01	3	6.46E-01	NS	NS	0.8040
3133	HLA-E	20	1.74E-01	18	3.23E-03	NS	NS	0.0581

397	ARHGDIB	20	1.77E-01	21	1.44E-02	NS	NS	0.1865
27166	PRELID1	5	1.77E-01	5	1.08E-01	NS	NS	0.5385
10175	CNIH	9	1.78E-01	8	4.19E-02	NS	NS	0.3352
1054	CEBPG	13	1.78E-01	11	1.42E-03	NS	NS	0.0157
3552	IL1A	11	1.80E-01	11	2.95E-01	NS	NS	1.0000
1440	CSF3	5	1.82E-01	4	9.77E-02	NS	NS	0.3909
7096	TLR1	7	1.84E-01	10	4.60E-01	NS	NS	0.9887
3539	IGLC3	9	1.84E-01	9	7.26E-02	NS	NS	0.5451
684	BST2	7	1.86E-01	7	5.22E-01	NS	NS	0.9920
23098	SARM1	10	1.87E-01	10	9.10E-02	NS	NS	0.5922
4986	OPRK1	25	1.90E-01	25	6.44E-03	NS	NS	0.1610
9447	AIM2	2	1.93E-01	2	2.33E-01	NS	NS	0.4655
1673	DEFB4	1	1.95E-01	0	N/A	NS	NS	N/A
1130	LYST	18	1.98E-01	16	3.14E-01	NS	NS	0.9586
140850	DEFB127	10	1.98E-01	10	1.08E-01	NS	NS	1.0000
1437	CSF2	6	2.02E-01	6	5.04E-01	NS	NS	0.9761
3562	IL3	4	2.02E-01	4	5.04E-01	NS	NS	0.9761
10581	IFITM2	6	2.03E-01	4	9.18E-02	NS	NS	0.3674
54795	TRPM4	15	2.04E-01	15	8.91E-02	NS	NS	0.6078
5871	MAP4K2	6	2.04E-01	5	1.20E-01	NS	NS	0.3017
728	C5AR1	3	2.06E-01	3	7.22E-02	NS	NS	0.1200
10507	SEMA4D	26	2.06E-01	26	1.59E-03	NS	NS	0.0413
1234	CCR5	2	2.08E-01	2	4.49E-01	NS	NS	0.5831
3976	LIF	9	2.09E-01	8	2.30E-03	NS	NS	0.0103
7126	TNFAIP1	8	2.13E-01	8	2.46E-01	NS	NS	0.8444
10871	CD300C	10	2.19E-01	10	6.67E-01	NS	NS	1.0000
7448	VTN	4	2.21E-01	4	2.46E-01	NS	NS	0.5392
3512	IGJ	7	2.24E-01	8	4.32E-01	NS	NS	0.8219
973	CD79A	4	2.30E-01	4	5.08E-01	NS	NS	0.9150
28776	IGLV7-43	11	2.30E-01	11	1.73E-01	NS	NS	0.7184
57824	HMHB1	13	2.32E-01	13	1.71E-01	NS	NS	0.7254
4436	MSH2	19	2.40E-01	17	2.27E-03	NS	NS	0.0387
64135	IFIH1	6	2.42E-01	4	5.25E-01	NS	NS	0.8092
388646	GBP7	9	2.49E-01	9	4.27E-01	NS	NS	0.9774
8575	PRKRA	7	2.52E-01	7	3.31E-01	NS	NS	0.7709
3110	MNX1	2	2.57E-01	3	2.05E-01	NS	NS	0.6143
115361	GBP4	9	2.61E-01	9	4.27E-01	NS	NS	0.9774
9214	FAIM3	7	2.67E-01	5	4.27E-01	NS	NS	0.7884
925	CD8A	5	2.70E-01	6	5.29E-01	NS	NS	0.6926
57105	CYSLTR2	6	2.72E-01	6	9.93E-03	NS	NS	0.0596
117285	DEFB118	5	2.73E-01	5	5.85E-05	NS	NS	0.0003
7462	LAT2	2	2.75E-01	2	1.36E-01	NS	NS	0.2628
2495	FTH1	12	2.79E-01	12	8.39E-02	NS	NS	0.6827
84174	SLA2	6	2.81E-01	6	3.11E-01	NS	NS	0.8712
712	C1QA	7	2.87E-01	6	1.12E-01	NS	NS	0.6742
714	C1QC	9	2.87E-01	8	1.12E-01	NS	NS	0.8990
910	CD1B	3	2.90E-01	2	3.35E-01	NS	NS	0.6144
2217	FCGRT	4	2.94E-01	5	4.16E-02	NS	NS	0.2081
6976	TRGV3	7	2.98E-01	7	2.90E-01	NS	NS	0.9176
55611	OTUB1	4	3.04E-01	5	6.29E-02	NS	NS	0.1556
7057	THBS1	9	3.05E-01	7	4.46E-01	NS	NS	0.9671
8763	CD164	6	3.15E-01	5	5.17E-02	NS	NS	0.1824
4153	MBL2	28	3.24E-01	27	6.05E-02	NS	NS	0.9020
27190	IL17B	4	3.29E-01	4	5.58E-02	NS	NS	0.1347
3565	IL4	8	3.33E-01	8	1.80E-01	NS	NS	0.8975
3596	IL13	6	3.33E-01	6	1.80E-01	NS	NS	0.8732
4791	NFKB2	6	3.36E-01	6	2.64E-01	NS	NS	0.4837
664	BNIP3	4	3.44E-01	3	9.29E-03	NS	NS	0.0279
2215	FCGR3B	2	3.46E-01	3	5.29E-02	NS	NS	0.1588
2214	FCGR3A	5	3.46E-01	4	5.29E-02	NS	NS	0.2117
713	C1QB	9	3.51E-01	8	2.36E-01	NS	NS	0.8632
6352	CCL5	3	3.53E-01	3	7.64E-01	NS	NS	0.9211

28988	DBNL	4	3.70E-01	4	4.83E-01	NS	NS	0.7452
51561	IL23A	2	3.71E-01	2	6.67E-02	NS	NS	0.0747
3430	IFI35	3	3.76E-01	3	2.80E-01	NS	NS	0.8393
80329	ULBP1	5	3.81E-01	8	2.68E-01	NS	NS	0.8803
340205	TREML1	3	3.85E-01	3	3.08E-01	NS	NS	0.9238
54209	TREM2	4	3.85E-01	4	1.68E-01	NS	NS	0.4612
5699	PSMB10	2	3.85E-01	2	1.81E-01	NS	NS	0.3629
79626	TNFAIP8L2	6	3.91E-01	6	5.56E-01	NS	NS	0.9748
6361	CCL17	10	4.13E-01	12	1.52E-01	NS	NS	0.9234
28442	IGHV3-23	1	4.14E-01	0	N/A	NS	NS	N/A
3811	KIR3DL1	3	4.16E-01	3	8.75E-01	NS	NS	0.9287
79132	DHX58	5	4.17E-01	3	3.96E-01	NS	NS	0.7905
2302	FOXJ1	4	4.26E-01	4	4.84E-01	NS	NS	0.7269
7494	XBP1	4	4.30E-01	4	9.80E-02	NS	NS	0.1921
246778	IL27	3	4.54E-01	3	1.27E-03	NS	NS	0.0038
51554	CCRL1	1	4.63E-01	1	8.55E-01	NS	NS	0.8552
5452	POU2F2	4	4.84E-01	5	2.83E-01	NS	NS	0.7142
4615	MYD88	5	4.86E-01	5	1.91E-01	NS	NS	0.4291
10437	IFI30	5	5.13E-01	5	1.55E-01	NS	NS	0.7762
5970	RELA	6	5.20E-01	6	4.74E-01	NS	NS	0.7798
3558	IL2	3	5.33E-01	3	1.91E-02	NS	NS	0.0574
5734	PTGER4	3	5.39E-01	3	9.63E-02	NS	NS	0.2889
260434	PYDC1	1	5.46E-01	1	1.47E-01	NS	NS	0.1470
6373	CXCL11	4	5.62E-01	4	4.15E-03	NS	NS	0.0059
5989	RFX1	3	5.81E-01	3	3.70E-01	NS	NS	0.7378
729230	FLJ78302	1	6.07E-01	1	4.49E-01	NS	NS	0.4493
10068	IL18BP	2	6.18E-01	2	2.23E-01	NS	NS	0.4451
3514	IGKC	1	6.50E-01	0	N/A	NS	NS	N/A
1510	CTSE	1	6.75E-01	1	2.35E-01	NS	NS	0.2348
1237	CCR8	2	6.77E-01	5	7.38E-02	NS	NS	0.1394
5896	RAG1	4	7.33E-01	4	3.92E-02	NS	NS	0.1570
28912	IGKV3-20	1	7.84E-01	0	N/A	NS	NS	N/A
10383	TUBB2C	1	9.90E-01	1	4.70E-01	NS	NS	0.4703
